# A titanium redox-switch enables reversible C–C bond forming and splitting reactions

**DOI:** 10.1039/d5sc04824a

**Published:** 2025-08-22

**Authors:** Mehrafshan G. Jafari, Dominik Fehn, Christian Sandoval-Pauker, Michael R. Gau, Karsten Meyer, Balazs Pinter, Daniel J. Mindiola, Anders Reinholdt

**Affiliations:** a Department of Chemistry, University of Pennsylvania 231 South 34th Street Philadelphia PA 19104 USA mindiola@sas.upenn.edu; b Department of Chemistry and Pharmacy, Inorganic Chemistry, Friedrich-Alexander-Universität (FAU) Erlangen-Nürnberg Erlangen 91058 Germany karsten.meyer@fau.de; c Department of Chemistry and Biochemistry, University of Texas at El Paso El Paso Texas 79968 USA pinter.balazs@gmail.com; d Centre for Analysis and Synthesis, Department of Chemistry, Lund University Naturvetarvägen 22 Lund 22100 Sweden anders.reinholdt@chem.lu.se

## Abstract

Using an Earth-abundant transition metal to mediate formation and splitting of C–C σ-bonds, in response to electrical stimuli, constitutes a promising strategy to construct complex organic skeletons. Here, we showcase how [^*n*^Bu_4_N][N_3_] reacts with an isocyanide adduct of a tetrahedral and high-spin Ti^II^ complex, [(Tp^*t*Bu,Me^)TiCl] (1), to enact N-atom transfer, C–N bond formation, and C–C coupling, to form a dinuclear complex, [(Tp^*t*Bu,Me^)Ti{AdN(N)C–C(N)NAd}Ti(Tp^*t*Bu,Me^)] (3), with two Ti^III^ ions bridged by a disubstituted oxalimidamide ligand (^*n*^Bu = *n*-butyl, Tp^*t*Bu,Me^ = hydrotris(3-*tert*-butyl-5-methylpyrazol-1-yl)borate, Ad = 1-adamantyl). Magnetic and computational studies reveal two magnetically isolated d^1^ Ti^III^ ions, and electrochemical studies unravel a reversible two-electron oxidation at −0.87 V *vs.* [FeCp_2_]^0/+^. Despite these observations, chemical oxidation of 3, ultimately, leads to rupture of the oxalimidamide moiety with C–C bond splitting to form [(Tp^*t*Bu,Me^)Ti{1,3-μ_2_-AdNCN}_2_Ti(Tp^*t*Bu,Me^)][B(C_6_F_5_)_4_]_2_ (4), which displays an antiferromagnetically coupled Ti_2_^III,III^ configuration, mediated by superexchange through its bridging carbodiimide ligands. A comparative reactivity study of isocyanide toward a transient vanadium nitride [(Tp^*t*Bu,Me^)V≡N(THF)] (5) gives further insight into the structure of putative intermediates involved in the coupling sequence.

## Abstract 2

A dinuclear titanium complex remains in a trivalent valence state, during redox-driven formation and breakage of a C–C σ-bond.

## Graphical abstract

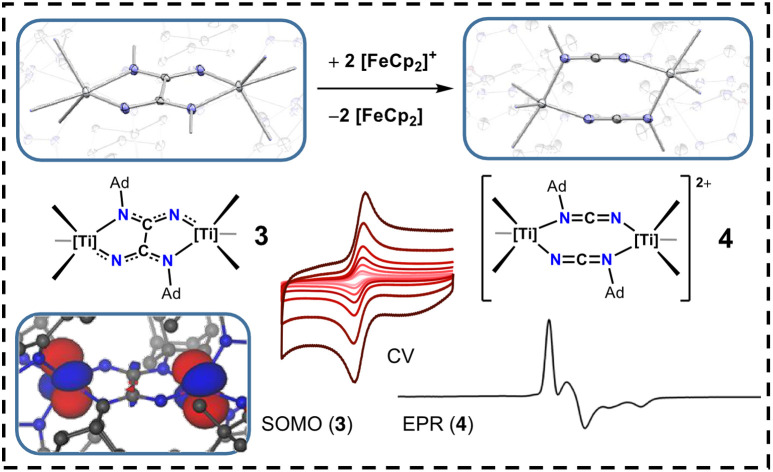

## Introduction

**Chart 1:**
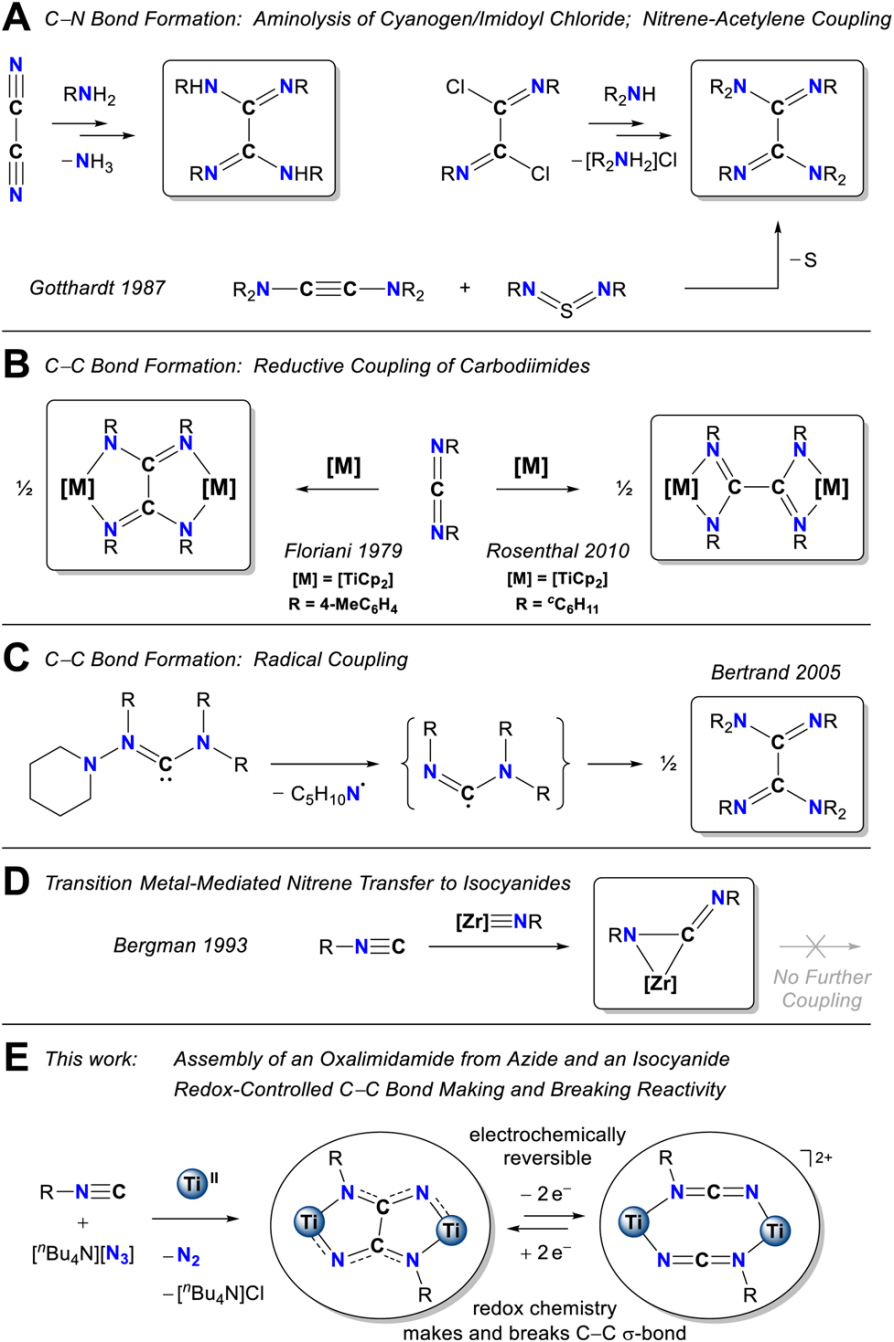
Synthesis of sterically congested oxalimidamides. (A) Aminolysis of cyanogen and oxalimidoyl chlorides; nitrene transfer to a diaminoacetylene. (B) Metal-mediated reductive coupling of carbodiimides. (C) C–C coupling of aminoimidoyl radicals. (D) Coupling of isocyanide and a zirconium imido does not lead to further coupling steps. (E) This work showing Ti^II^-mediated coupling of isocyanide and azide to yield a disubstituted oxalimidamide (R = Ad).

The development of synthetic methods that simultaneously assemble a manifold of carbon–carbon and carbon–nitrogen bonds is a central step toward converting simple chemical precursors to complex organic skeletons in few synthetic steps. Oxalimidamides are derivatives of the ubiquitous oxalic acid, H_2_C_2_O_4_, in which all oxygen atoms are replaced by nitrogen. The {C_2_N_4_} cores in these unsaturated fragments are ideally suited for constructing challenging nitrogen-rich motifs, for example polycyclic N-heterocycles^1^ and tetraaminoethylenes.^2^ Synthetic strategies toward oxalimidamides have historically relied on the formation of carbon–nitrogen bonds. Examples include aminolysis of carboxylic acid derivatives such as cyanogen^3^ and oxalimidoyl chlorides.^4^ Likewise, Gotthardt and co-workers generated an oxalimidamide upon nitrene transfer from a sulfur(iv) diimide to a diaminoacetylene (Chart 1A).^5^ More recently, strategies toward oxalimidamide have focused on assembling the central carbon–carbon σ-bond: Akin to the reductive coupling of heterocumulenes such as CO_2_ or CS_2_ into oxalate^6^ or tetrathiooxalate^7^ motifs, respectively, Chart 1B shows how reducing metal complexes convert carbodiimides (RNCNR) into two distinct oxalimidamide archetypes,^8^ having either 5-membered (Floriani^9^ and co-workers) or 4-membered chelate rings (Rosenthal^10^ and co-workers). While these products evidently form *via* disparate reaction pathways, and conceivably could involve radical intermediates, very little mechanistic information is available about the metal-promoted couplings. Interestingly, Bertrand and co-workers showed that, in the absence of a metal reductant, a transient aminoimidoyl radical underwent C–C coupling to form a hexasubstituted oxalimidamide (Chart 1C).^11^ A central limitation of the synthetic strategies outlined in Chart 1A–C is that they exclusively lead to sterically congested oxalimidamides, bearing four or more substituents. Because sparsely substituted {C_2_N_4_} cores could offer unique access to extended π-conjugated systems, we sought strategies to construct these motifs in a modular fashion. Interestingly, early transition metal multiple-bonds, {M≡E}, are prone to couple with isocyanides to afford various {M(ECNR)} heterocumulenic products (E = C,^12^ N,^13^ NR,^14^ PR,^15^ O^16^). However, despite possessing reducing metal nodes that should render the central carbon atoms reactive sites, carbodiimide complexes produced in this fashion (Chart 1D) have hitherto proven to be stagnant for subsequent C–C coupling. Realizing the reduced steric constraints associated with a nitride-based coupling scheme, we examined a three-component deazotation strategy in which an early transition metal complex, an isocyanide, and an azide would combine to offer unique access to a disubstituted {C_2_N_4_} core.

Herein, we report how a tetrahedral and high-spin Ti^II^ complex binds an isocyanide to afford a five-coordinate adduct, which undergoes halide exchange with azide salt [^*n*^Bu_4_N][N_3_] to form a transient Ti^II^ azide-isocyanide intermediate. A manifold of N_2_-extrusion, C–N bond formation, and C–C coupling steps lead to a paramagnetic bimetallic complex bearing disubstituted oxalimidamide (Chart 1E). Using a combination of X-band EPR, solid state magnetometry, and theoretical studies, we demonstrate that the two unpaired electrons in the Ti_2_^III,III^ core are housed in two metallocentric molecular orbitals having δ and δ* parentage, resulting in each Ti^III^ center being electronically isolated by the bridging oxalimidamide ligand. In addition, our electrochemical studies show that the new Ti_2_^III,III^ species undergoes a fully reversible two-electron oxidation at low potential (−0.87 V *vs.* ferrocene/ferrocenium). Although the oxalimidamide complex possesses metallo-radical character, and in spite of its electrochemical redox reversibility, chemical oxidation results in scission of its central C–C bond to form a pair of linear, bridging, and monosubstituted carbodiimide ligands {Ti(1,3-μ_2_-NCNR)_2_Ti}, while the Ti_2_^III,III^ oxidation state remains unchanged. Finally, to elucidate the geometry of potential coupling intermediates, we show how a structurally analogous, tetrahedral and high-spin V^II^ complex brings about distinctly different coupling reactivity, resulting in a new C–N bond but avoiding a C–C bond formation step, when treated with isocyanide and azide.

## Results and discussion

### A Ti^II^ center reductive couples an isocyanide and an azide ion to form an oxalimidamide scaffold (3)

**Fig. 1:**
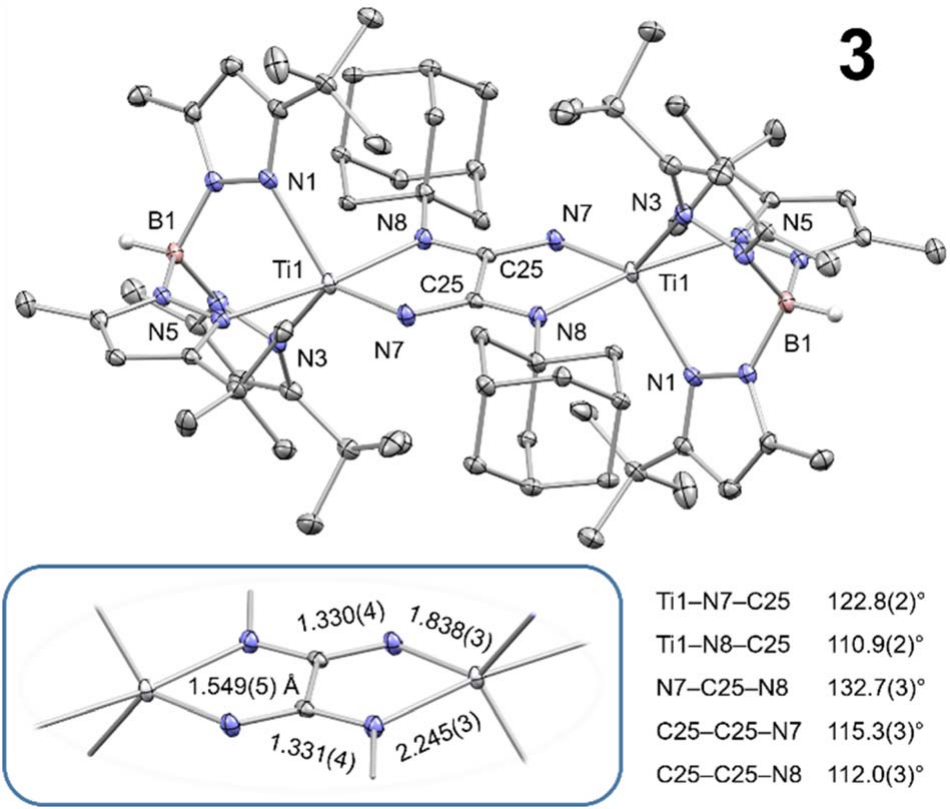
Thermal ellipsoid plot of 3 (50% probability level). Co-crystallized diethyl ether, hydrogen atoms (except B–H), and disorder of Ad groups are omitted for clarity purposes.

**Scheme 1:**
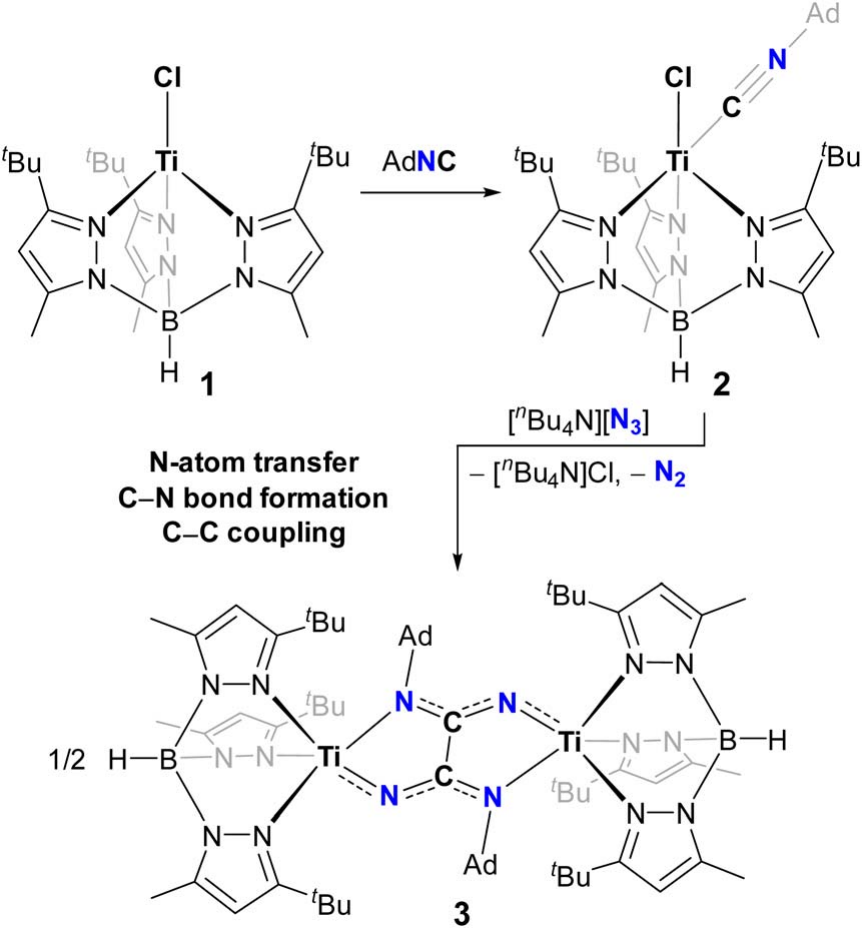
Treatment of tetrahedral Ti^II^ complex 1 with AdNC to form adduct 2, followed by N-atom transfer from N_3_^−^ to afford dinuclear oxalimidamide complex 3.

We recently reported how a high-spin and tetrahedral Ti^II^ complex, [(Tp^*t*Bu,Me^)TiCl] (1) reacts with the isocyanide AdNC to form a paramagnetic and five-coordinate adduct, [(Tp^*t*Bu,Me^)TiCl(CNAd)] (2).^17^ Upon reaction with pnictogen transfer reagents, Na(OCPn), the activated isocyanide ligand in 2 forms a bond with the Pn-atom (Pn = P, As) and displaces CO to afford mononuclear complexes having η^3^-bonded cyanophosphide or cyanoarsenide frameworks, [(Tp^*t*Bu,Me^)Ti(η^3^-PnCNAd)].^18^ In contrast, reaction of 2 with AdN_3_ proceeds instead with substitution of the isocyanide ligand to afford a metastable azide adduct, [(Tp^*t*Bu,Me^)TiCl(N_3_Ad)], which allowed us to mechanistically probe the conversion of an azide to an imide.^19^ In view of these divergent reactions, we inquired whether an azide ion, being sterically less hindered than AdN_3_, might deliver an N-atom to the isocyanide akin to how the OCPn^−^ ions did. Under an Ar atmosphere, treatment of a toluene solution of 2 with [^*n*^Bu_4_N][N_3_] resulted in effervescence over 1 minute accompanied with a color change from maroon to dark green. In the course of minutes, dark green crystals of a bimetallic complex, [(Tp^*t*Bu,Me^)Ti{AdN(N)C–C(N)NAd}Ti(Tp^*t*Bu,Me^)] (3, *cf.*Fig. 1 and Scheme 1), began forming along with colorless crystals of [^*n*^Bu_4_N]Cl. The two crystalline products could be separated by washing away [^*n*^Bu_4_N]Cl with acetonitrile to afford pure 3 in 30% isolated yield. The dark green color of 3 is qualitatively reminiscent of cyanophosphide complex [(Tp^*t*Bu,Me^)Ti(η^3^-PCNAd)].^18^ UV-vis spectral characterization revealed electronic absorptions, which are more intense than would be expected for pure d–d transitions in a Werner-type complex (*λ*_max_ = 601, 432, 281, 223 nm; *ε* = 990, 8800, 34 600, 43 400 M^−1^ cm^−1^, respectively, see Fig. S9), indicating significant metal–ligand covalency in complex 3. Moreover, ^1^H NMR spectroscopic characterization revealed two sets of paramagnetically shifted pyrazole resonances integrating in a 1 : 2 ratio (−0.4 to 29.3 ppm), indicative of a local *C*_s_ symmetric structure about the titanium ion. Whereas the ^1^H NMR spectral resonances from 3 fall in the range expected for a [(Tp^*t*Bu,Me^)Ti^III^] complex (*cf.* [(Tp^*t*Bu,Me^)TiCl_2_] at 1.9 to 25.6 ppm),^17^ the solubility of 3 is very low in organic solvents such as THF, diethyl ether, toluene, benzene, and DMSO (<1 mg mL^−1^), hampering determination of a solution-state magnetic moment by Evans' method. Moreover, complex 3 readily decomposes in CH_2_Cl_2_ (instant decolorization), likely as a result of this solvent acting as a chlorinating agent toward the early transition metal.^20^

### Molecular structure of oxalimidamide complex 3

Given its limited solubility, complex 3 crystallizes readily from organic solvents such as THF, diethyl ether, toluene, and benzene. X-ray crystallographic studies identified various solvates of 3, in which the bimetallic complex displays essentially identical metrics; Fig. 1 depicts a thermal ellipsoid plot of 3 as its diethyl ether solvate (space group *P*1̄). The two titanium centers are related by inversion and adopt a distorted five-coordinate structure, resembling an idealized trigonal bipyramidal geometry more closely than a square pyramidal geometry (*τ*_5_ = 0.71).^21^ The most conspicuous structural motif in 3 is the oxalimidamide ligand, {AdN(N)CC(N)NAd}^*n*−^. The central {C_2_N_4_} fragment defines a plane (angle sum around C25 and N8 equal 360°), which also contains the titanium centers, both BH groups, the quaternary carbons from the adamantyl groups, and one pyrazolyl ring from each Tp^*t*Bu,Me^ moiety. As opposed to the more strained four-membered rings shown in Chart 1B (right), the oxalimidamide ligand in 3 forms a five-membered chelate ring with each titanium center. While the Ti–N_pyrazole_ bond distances in 3 are overall quite uniform (2.228(3)–2.307(3) Å), there is a dramatic decrease (>0.40 Å) between the Ti–N bond distances for the monosubstituted (N8, 2.245(3) Å) and the unsubstituted (N7, 1.838(3) Å) nitrogen atoms. This difference is attributed to steric effects and variations in Ti–N π-bonding between the Ti–NAd and Ti=N fragments (*vide infra*). It should be noted that the C–N bond distances within the {C_2_N_4_} fragment are statistically indistinguishable. Finally, the most informative geometric feature of 3 is the C25–C25 bond distance at 1.549(5) Å (Fig. 1), which falls in the typical range for carbon–carbon single bonds.^22^ This assignment invites an isoelectronic analogy with the oxalate ion, {C_2_O_4_}^2−^, which in turn renders 3 best described as two Ti^III^ centers bridged by a tetraanionic {AdN(N)C–C(N)NAd}^4−^ ligand, in accord with spectroscopic data (*vide supra*).

### Mechanism for N-atom transfer and C–C coupling to form oxalimidamide complex 3

**Scheme 2:**
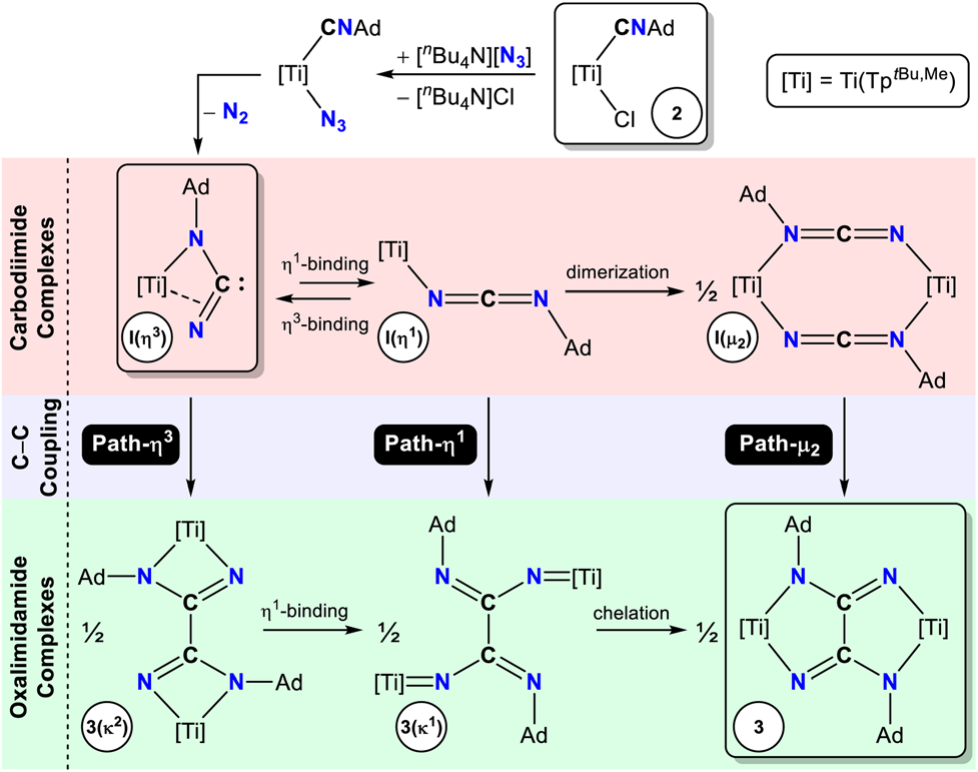
Conceivable mechanistic pathways leading from isocyanide complex 2 to oxalimidamide complex 3.

While Pn-atom transfer to isocyanide complex 2 was previously shown to generate monometallic cyanophosphide or cyanoarsenide complexes, [(Tp^*t*Bu,Me^)Ti(η^3^-PnCNAd)],^18^ a similar N-atom transfer strategy shown here afforded instead a bimetallic oxalimidamide complex 3. This striking difference in reactivity prompted us to glean mechanistic information with the aid of solution-state DFT (TPSSh-D3/def2-TZVP), using non-truncated structures in the simulations and employing the Broken Symmetry (BS) approach to describe open-shell wavefunctions associated with antiferromagnetically coupled states. The formation of the key intermediate [(Tp^*t*Bu,Me^)Ti(NCNAd)] is envisioned to follow a pathway akin to that leading to the cyanophosphide analog [(Tp^*t*Bu,Me^)Ti(η^3^-PCNAd)],^18^ beginning with exchange of the chloride ligand in 2 for an azide, to yield an azide intermediate [(Tp^*t*Bu,Me^)Ti(N_3_)(CNAd)]. Then, N-atom transfer from the azide to the isocyanide plausibly proceeds in a concerted fashion, such that the N⋯NN bond-breaking and N⋯CNAd bond-making steps proceed synchronously to produce a dinitrogen intermediate [(Tp^*t*Bu,Me^)Ti(N_2_)(NCNAd)]. Subsequent release of N_2_ would yield intermediate [(Tp^*t*Bu,Me^)Ti(NCNAd)]. The latter Ti^II^ complex predominantly exists as a triplet species bearing a monodentate and linear carbodiimide fragment, [(Tp^*t*Bu,Me^)Ti(η^1^-NCNAd)], I(η^1^), Scheme 2), which lies 30.8 kcal mol^−1^ lower in energy than the isomeric singlet species with chelating carbodiimide coordination, [(Tp^*t*Bu,Me^)Ti(η^3^-NCNAd)], I(η^3^).

The stability of the monodentate carbodiimide in I(η^1^) differs strikingly from a recent study by Tonks, which showed coordination of a carbodiimide to a [Cp_2_Ti^II^] system to form an isolable metallacyclic carbene.^23^ The finding is also in sharp contrast to the stability pattern observed for the heavier pnictogen analogs, which exist as stable singlet species [(Tp^*t*Bu,Me^)Ti(η^3^-PnCNAd)], in preference to triplet and/or dimerized isomerization/coupling products.^18^ From a molecular orbital standpoint, the distinct coordination modes of the cumulenic ligands, AdN=C=N^−^ and AdN=C=P^−^, reflects their disparate π-manifolds. The carbodiimide, having three 2p_*x*_ and three 2p_*y*_ orbitals oriented perpendicular to the internuclear *z*-axis, possesses a very strong π-bonded framework, which is intrinsically resistant to bending, thus favoring η^1^ binding. Contrarily, the heavier cyanophosphide congener possesses a 3p node, and the less efficient π-orbital overlap within this cumulene mitigates the energetic penalty for bending the AdN=C=P^−^ fragment into an η^3^ binding mode. These structural and electronic differences provide the driving force for the subsequent dimerization of the carbodiimide intermediate, I(η^1^), into the oxalimidamide complex 3.

**Fig. 2:**
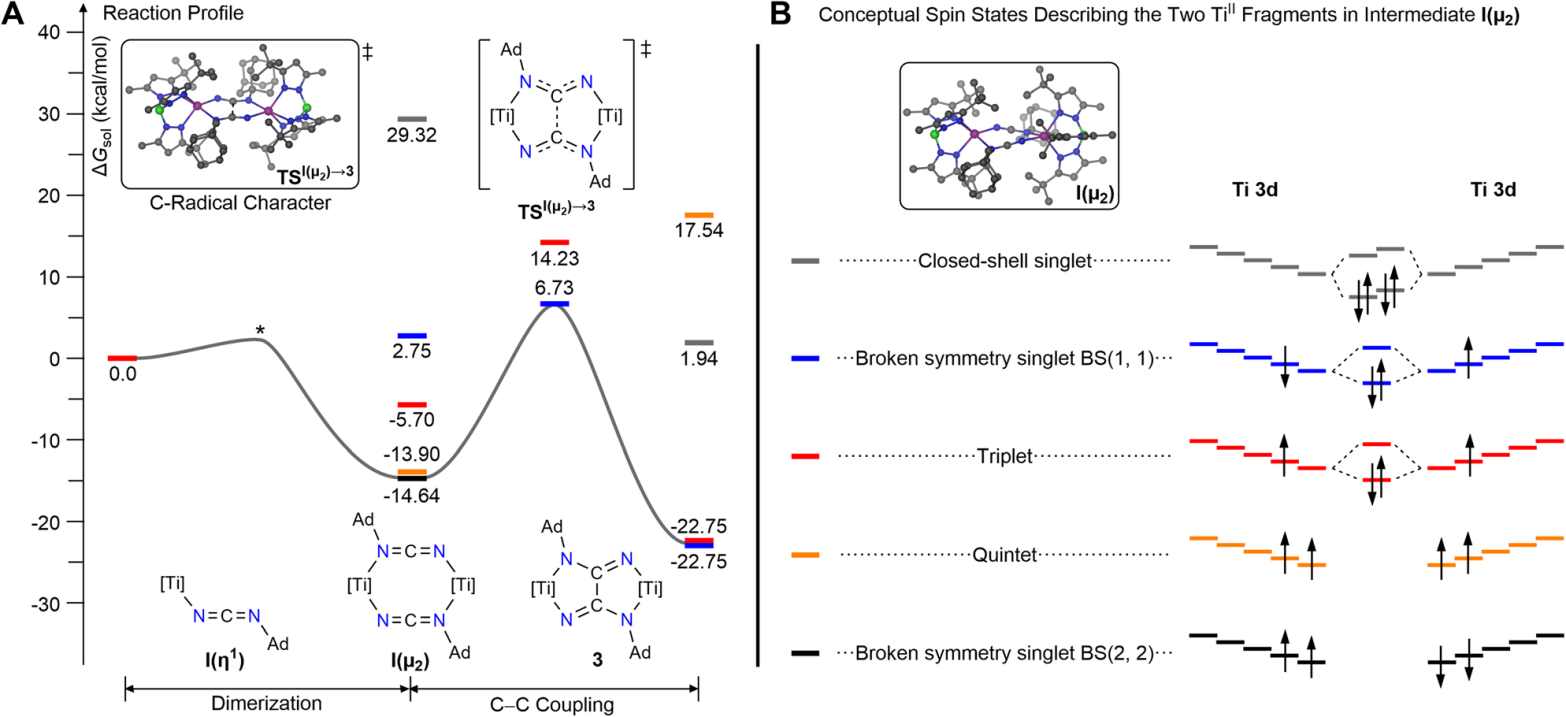
(A) Reaction profile for most likely pathway forming the C–C bond of the oxalimidamide, based on computational studies (TPSSh-D3/def2-TZVP). An energetically shallow transition state (*) converged to I(μ_2_); this transition state was not located on the PES. (B) Conceptual spin states describing I(μ_2_). For unsymmetrical wavefunctions, representing antiferromagnetic coupling of the unpaired electrons, we describe I(μ_2_) as consisting of two titanium fragments. We use the broken symmetry notation, BS(*n*,*m*), to indicate *n* unpaired spin-up electrons on the first titanium fragment and *m* unpaired spin-down electrons on the second fragment.

Starting from I(η^1^) or I(η^3^), three distinct C–C bond-forming events could occur (Scheme 2). The first scenario (Path-η^3^) involves a C–C bond-forming step between two units of I(η^3^) to produce an isomer of 3 having four-membered chelate rings, 3(κ^2^); subsequent de-chelation of the strained ring would lead to coordinatively unsaturated intermediate 3(κ^1^), and re-chelation would ultimately complete the isomerization into 3. The C–C bond forming step associated with this reaction path has a prohibitively high activation barrier of ∼50 kcal mol^−1^ (see Fig. S20), on both the triplet and the broken-symmetry singlet manifolds, with respect to the most stable intermediate I(η^1^), therefore making Path-η^3^ unlikely. It is worth mentioning that a large portion of the activation barrier stems from the simple fact that intermediate I(η^3^) is thermodynamically unstable (by 30.8 kcal mol^−1^) with respect to I(η^1^). The second scenario (Path-η^1^) would proceed with a C–C bond-forming step directly between two units of I(η^1^) to produce intermediate 3(κ^1^); a chelation step would then lead to 3. This mechanism was also found to be non-operational given the large activation barriers (57 and 48 kcal mol^−1^) associated with the C–C bond formation step in the triplet and broken-symmetry singlet manifold. Finally, the third scenario (Path-μ_2_), involves the early dimerization of two units of I(η^1^) into a bimetallic Ti^II^ species having two bridging carbodiimide ligands, [(Tp^*t*Bu,Me^)Ti(1,3-μ_2_-NCNAd)_2_Ti(Tp^*t*Bu,Me^)], I(μ_2_), which undergoes C–C coupling to produce complex 3. The dimerization of I(η^1^) to I(μ_2_) was calculated to take place with a driving force of −13.9 or −14.7 kcal mol^−1^, depending on the resulting spin state (Fig. 2A). The quintet configuration of I(μ_2_) with two ferromagnetically coupled Ti^II^ centers is slightly less stable (by 0.8 kcal mol^−1^) than the weakly antiferromagnetically coupled singlet state, BS(2,2). Note that the label, BS(*n*,*m*), describes an antiferromagnetically coupled state of I(μ_2_), consisting of two titanium centers, having *n* spin-up electrons on the first titanium center and *m* spin-down electrons on the second center (Fig. 2B). Starting from I(μ_2_), we could locate the transition state (TS) corresponding to the C–C bond formation event in two spin states, namely the triplet state and the broken-symmetry singlet state with two Ti^III^ centers (BS(1,1)). The stabilization of these spin-states relative to the quintet implies that the transition state leading to C–C bond formation proceeds through carbon-centered radicals coupled in a strong antiferromagnetic fashion. At the same time, the metal-centered radicals are coupled either *via* weak antiferromagnetic (BS(1,1)) or ferromagnetic (BS(0,1), triplet) interactions. The broken-symmetry singlet transition state with weak antiferromagnetically coupled Ti^III^ (d^1^) centers is 7.5 kcal mol^−1^ more stable than the triplet TS; the barrier to cross this ^BS^TS^I(μ^_2_^)→3^ lies 21.4 kcal mol^−1^ above dimer I(μ_2_), which indicates rapid kinetics at room temperature, consistent with our experimental observations. Overall, the reaction involving I(η^1^) → 3 is exergonic overall by about −22.8 kcal mol^−1^ (Fig. 2).

### Electronic structure studies identify a Ti_2_^III,III^ configuration for oxalimidamide complex 3

**Fig. 3:**
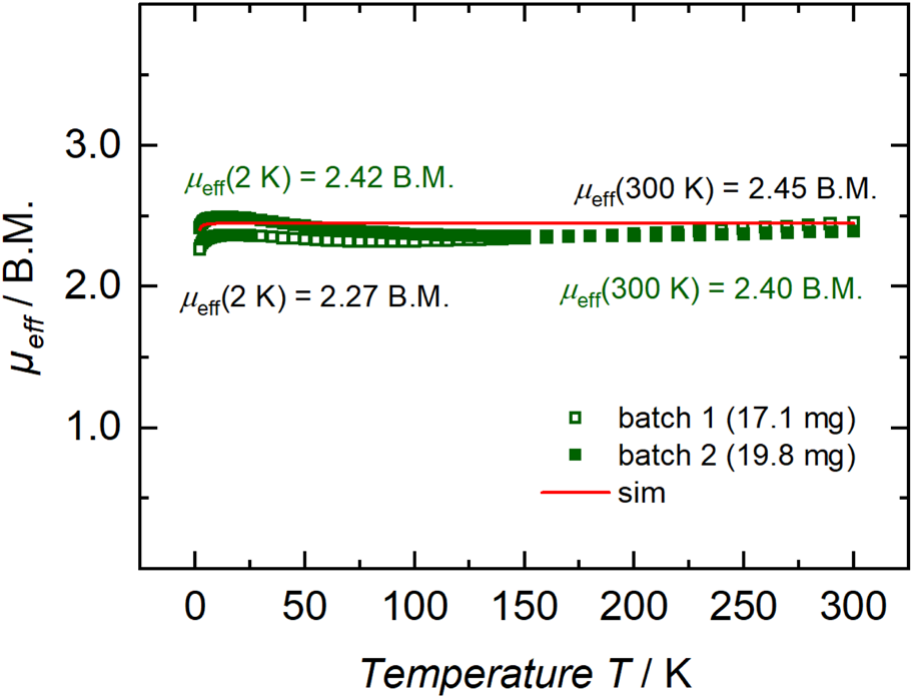
Temperature-dependent SQUID magnetization data of two independently synthesized batches of polycrystalline 3 (green squares) and a simulation (red trace), recorded with an applied magnetic field of 1 T (2–300 K), plotted as *μ*_eff_*vs. T*. Simulation parameters for an ideal uncoupled d^1^–d^1^ diradical system: *S*_1_ = *S*_2_ = 1/2, *J* = 0.00 cm^−1^, |*D*_1_| = |*D*_2_| = 0.00 cm^−1^, *E*/*D*_1_ = *E*/*D*_2_ = 0.00, *g*_avg,1_ = *g*_avg,2_ = 2.00. The simulation assumes a fully desolvated system; the presence of residual toluene in the crystal lattice (two C_7_H_8_ per formula unit of 3, 14% molar mass increase) readily explains the slight discrepancies in effective magnetic moment between the two analyzed batches of 3.

To corroborate the electronic structure of 3, we turned to experimental techniques that could shed light on its radical nature. The high sensitivity of the C–C coupled product toward oxygen and moisture, in tandem with its low solubility in inert solvents such as toluene and THF, prevented acquisition of reproducible EPR data. In contrast, SQUID magnetometry conducted on two independently synthesized, polycrystalline samples of 3 unequivocally and reproducibly revealed a paramagnetic system with a magnetic moment of 2.40 *μ*_B_ at 300 K (Fig. 3). This value is in close agreement with the expected value for two magnetically uncoupled d^1^ ions (*J* = 0.00 cm^−1^), each contributing to the total magnetic moment of the dinuclear system with the spin-only magnetic moment of an *S* = 1/2 system, [(1.73 *μ*_B_)^2^ + (1.73 *μ*_B_)^2^]^½^. On cooling, the magnetic moment of 3 remains practically constant, reaching 2.27 *μ*_B_ at 2 K. The final decrease in magnetic moment suggests the presence of very weak antiferromagnetic interactions between the two Ti^III^ ions in 3.

**Fig. 4:**
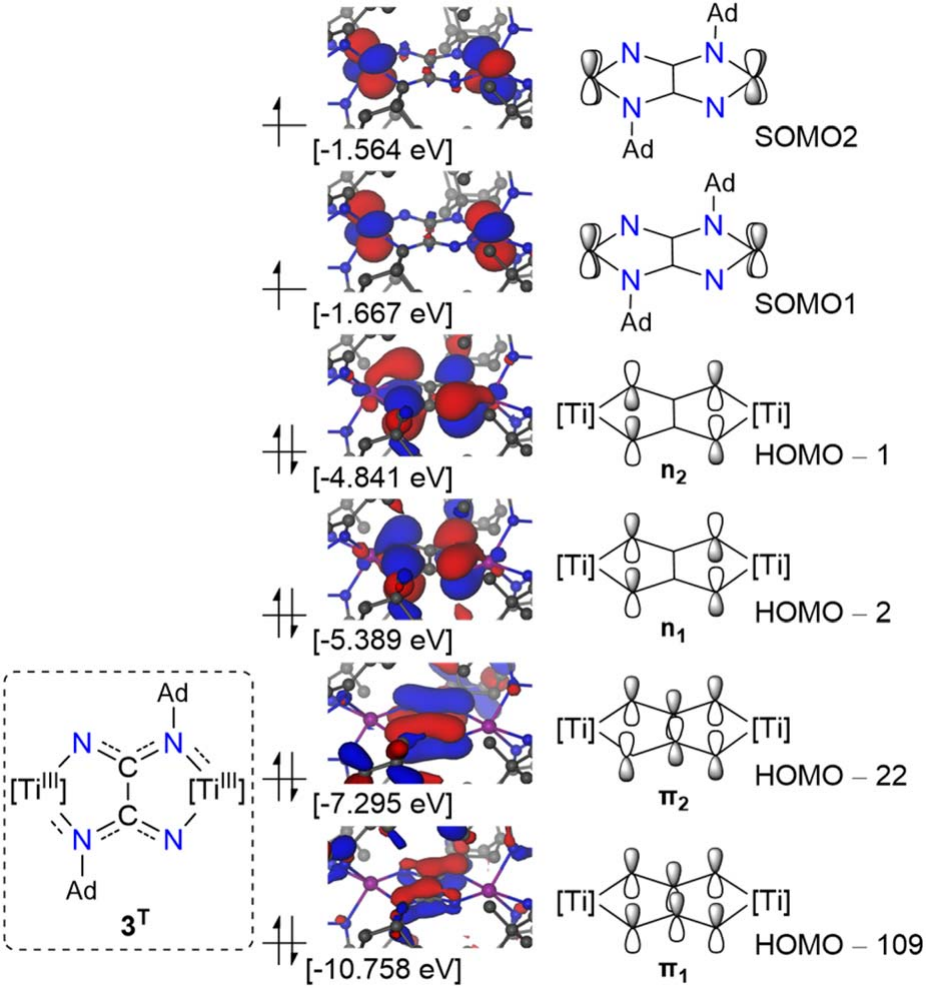
π-Type molecular orbitals for complex 3 (*S* = 1) with SOMO1 and SOMO2 representing metal δ and δ* character, respectively.

In line with these SQUID results, our DFT studies at the TPSSh-D3/def2-TZVP level of theory indicate that the triplet and broken-symmetry singlet states of 3 are essentially isoenergetic (Fig. S17), with the triplet state being only slightly more stable by a negligible margin (0.15 kcal mol^−1^). This feature also suggests that antiferromagnetic coupling within 3 must be very weak in accordance with the magnetometric data (Fig. 3, *vide supra*). In both spin states, the unpaired electrons are essentially metal-centric, describing a Ti_2_^III,III^ system (Fig. S18 and S19). The orbitals housing the two unpaired electrons are in-phase and out-of-phase Ti 3d linear combinations, having δ and δ* symmetry, respectively, characterized by SOMO1 and SOMO2 (Fig. 4). Notably, the unpaired electrons do not display delocalization onto the oxalimidamide. Considering the spatial orientation of HOMO−1 (n_2_) and HOMO−2 (n_1_) together with the difference in Ti–NAd and Ti=N Mayer bond orders (0.45 and 1.33, respectively), there is clear evidence that the unsubstituted nitrogen donor of the oxalimidamide ligand acts as a strong π-donor ligand, forcing the unpaired electrons on Ti^III^ into the set of non-bonding δ orbitals. The C–C bond order was found to be 0.9, *i.e.* a single bond, whereas the C–N bond orders are between 1.39 and 1.42, *i.e.* midway between single and double bonds. Finally, Fig. 4 depicts HOMO−22 and HOMO−109, showing two delocalized π-bonds along the N–C–N moieties in the oxalimidamide scaffold. According to these electronic structure analyses, complex 3 is best described as essentially two isolated Ti^III^ centers bridged by an oxalimidamide with a formal charge of −4.

### Electrochemical studies reveal a reversible oxidation for oxalimidamide complex 3

**Fig. 5:**
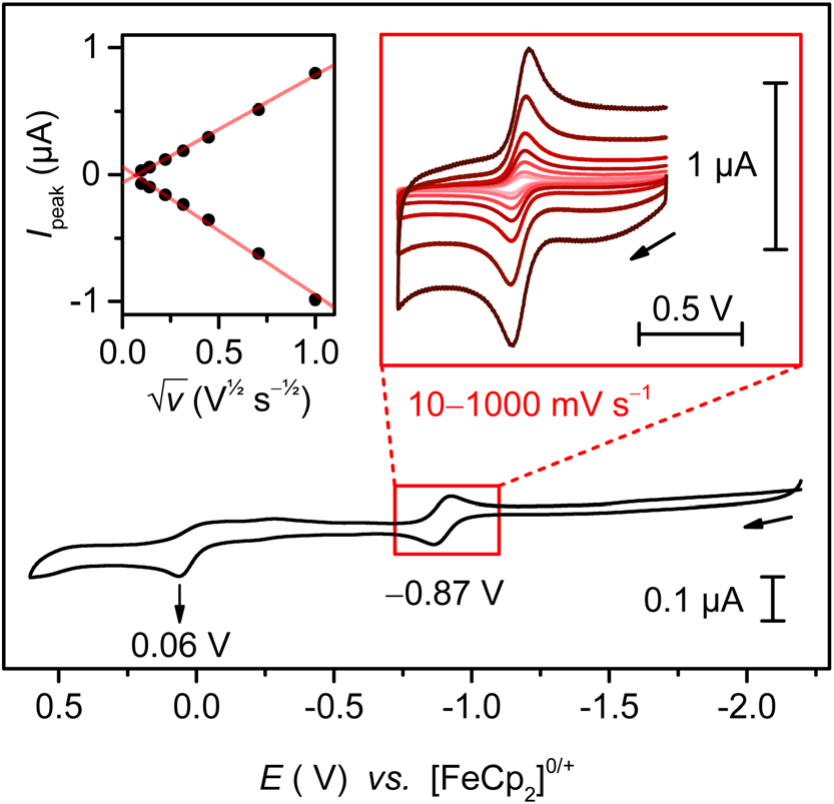
Cyclic voltammogram of 3 in 0.1 M [^*n*^Bu_4_N][PF_6_] in THF (10 mV s^−1^), referenced to [FeCp_2_]^0/+^ at 0.00 V. Insets show the reversible feature at −0.87 V at scan-rates from 10–1000 mV s^−1^ (right) and a plot of the anodic/cathodic peak currents *vs.* the square root of the scan rate (top left inset).

An oxalimidamide may undergo several redox transformations, including oxidation to two carbodiimides or reduction to a tetraaminoethylene fragment.^2^ In a complex such as 3, the metal centers (in our case, Ti_2_^III,III^) can furthermore assume various oxidation states. As such, oxalimidamide complexes have the potential to display rich metal/ligand-centered redox chemistry, but only a few studies have addressed this fundamental property. Based on IR and NMR spectroscopic studies, Floriani and co-workers reported that a Ti_2_^III,III^ oxalimidamide complex such as [Cp_2_Ti{(ArN)_2_C–C(NAr)_2_}TiCp_2_] (Ar = 4-CH_3_C_6_H_4_) could be oxidized by elemental iodine to afford what was proposed to be an isostructural Ti_2_^IV,IV^ derivative, but no X-ray crystallographic characterization was presented.^9*b*^ Given the predominant Ti 3d character of the SOMO1 and SOMO2 (Fig. 4, *vide supra*), our oxalimidamide complex 3 was expected to display a metal-centric oxidation with minimal structural rearrangement. Indeed, a cyclic voltammogram of 3 in THF, with 0.1 M of [^*n*^Bu_4_N][PF_6_] as electrolyte, revealed a reversible two-electron oxidation centered around −0.87 V and an additional irreversible anodic feature at +0.06 V (referenced to [FeCp_2_]^0/+^ at 0.00 V, Fig. 5). A plot of anodic/cathodic peak currents *vs.* the square root of the scan rate clearly shows the oxidation at lowest potential to be reversible under electrochemical conditions. The irreversibility of the second anodic wave at more positive potential suggests a hypothetical [3]^*n*+^ (*n* > 2) species to be unstable, particularly in the presence of the anions of the electrolyte.

Given the reversible nature of the first anodic event, we attempted to isolate this species, using an outer-sphere oxidant commensurate with the reduction potential. We chose an oxidant with a weakly coordinating anion in order to minimize the structural reorganization of the oxalimidamide ligand. Accordingly, when carrying out a chemical oxidation using solutions of dark green 3 and dark blue [FeCp_2_][B(C_6_F_5_)_4_] in THF, the colors of the reactants immediately faded to a lighter orange, from which orange crystals could be isolated in 90% yield. The same color change was also brought about when using a decamethylferrocenium oxidant, [FeCp*_2_][SO_3_CF_3_] in THF (−0.44 V *vs.* Fc^0/+^),^24^ ruling out that the oxidation product could correspond to the irreversible redox wave at +0.06 V.

### Chemical oxidation of oxalimidamide complex 3 leads to C–C bond splitting and formation of a carbodiimide complex (4)

**Scheme 3:**
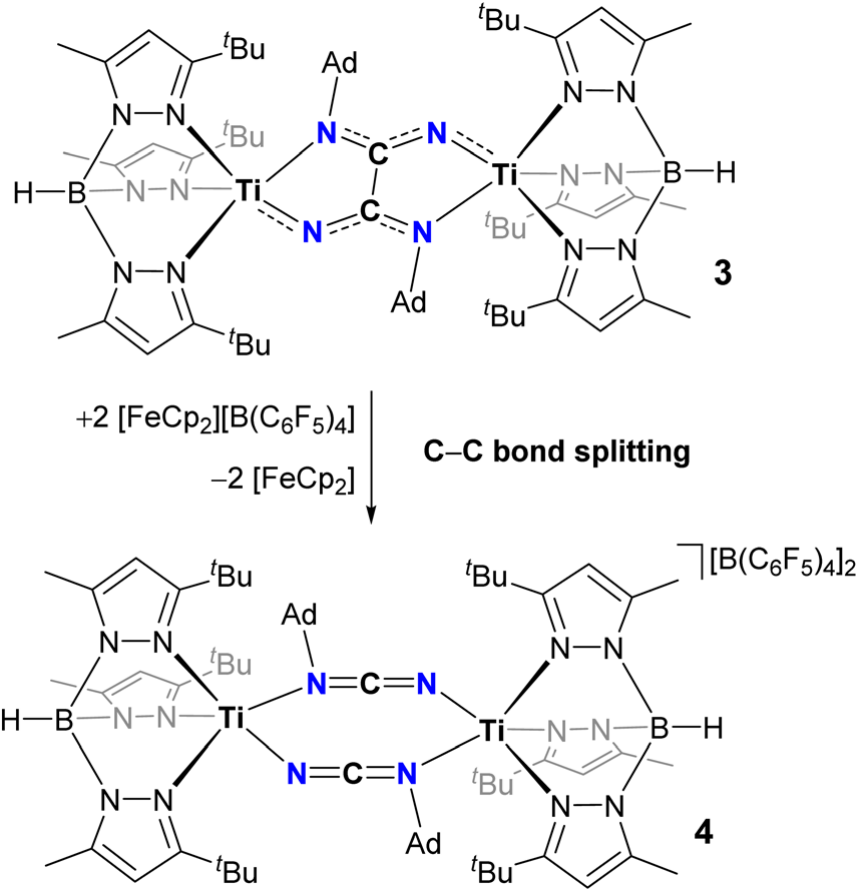
Two-electron oxidation of the oxalimidamide ligand in 3 to cleave the C–C bond and form carbodiimide complex 4.

**Fig. 6:**
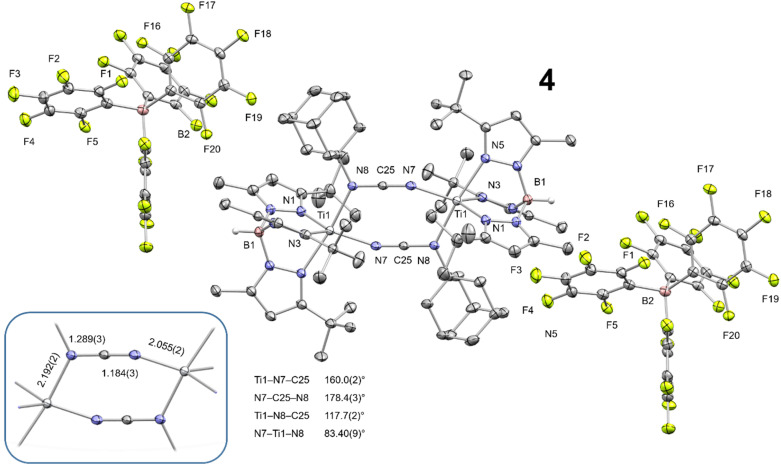
Thermal ellipsoid plot of 4 (50% probability level). Co-crystallized diethyl ether, hydrogen atoms (except B–H), and disorder of two ^*t*^Bu groups are omitted for clarity.

Contrary to expectation, the chemical oxidation of complex 3 did not generate a hypothetical “[3]^2+^” system consisting of two Ti^IV^ centers bridged by an intact and chelating oxalimidamide ligand. Instead, the two-electron oxidation produced a dinuclear complex bridged by two carbodiimide ligands, [(Tp^*t*Bu,Me^)Ti{1,3-μ_2_-NCNAd}_2_Ti(Tp^*t*Bu,Me^)][B(C_6_F_5_)_4_]_2_ (4), revealed by a single crystal X-ray diffraction structural study (Scheme 3 and Fig. 6). Complex 4 crystallizes in the centrosymmetric triclinic spacegroup (*P*1̄) with inversion-related titanium centers, each having a distorted five-coordinate geometry (*τ*_5_ = 0.77). All atoms that were confined to the plane defined by the central {C_2_N_4_} unit in 3 remain in a similar planar arrangement in the oxidized complex 4. The Ti–N_pyrazole_ bond distances in complex 4 (2.103(2)–2.213(2) Å) are on average ∼0.11 Å shorter than their counterparts in 3, in line with the higher effective charge of the Ti centers. On the other hand, both Ti–N_carbodiimide_ bond distances in 4 exceed 2.00 Å, therefore indicating negligible Ti–N π-bonding in the oxidized system. The bridging carbodiimide ligands display short C=N double bonds and a linear geometry about carbon, suggestive of a cumulenic resonance form as the result of oxidation. Taken together, these structural data indicate that 4, to a first approximation, can be viewed as two Ti^III^ centers bridged by two monoanionic AdN=C=N^−^ ligands. Our structural studies therefore showcase how the oxidation of a dinuclear Ti_2_^III,III^ oxalimidamide complex leaves invariant the oxidation state of the metals, in contrast to Floriani's conclusions about a related Cp-based Ti_2_^III,III^ oxalimidamide system.^9*b*^ Finally, the carbon atoms in the carbodiimide ligands display a separation, which lies well beyond a bonding interaction (2.821(4) Å) but within the sum of their van der Waals radii.^25^ These structural traits lend support to the proposal that 3 forms *via* the computationally identified Path-μ_2_ proceeding through a {Ti^II^(1,3-μ_2_-NCNAd)_2_Ti^II^} core structurally analogous to 4 (*vide supra*).

### Electronic structure studies show that carbodiimide complex 4 retains a Ti_2_^III,III^ configuration

**Fig. 7:**
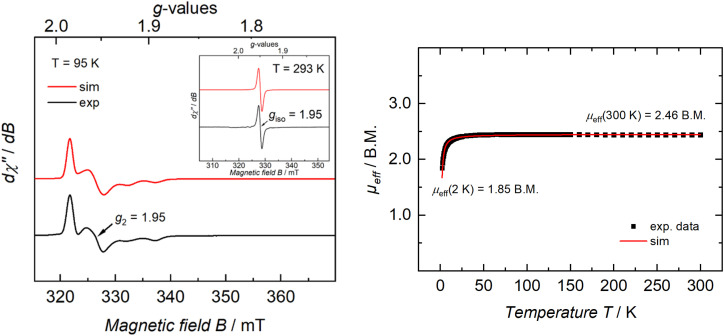
(Left) CW X-band EPR spectrum of a 1 mM solution of 4 in THF at 95 K (black trace), and its simulation (red trace). Simulation parameters: *g*_1_ = 1.98, *g*_2_ = 1.95, and *g*_3_ = 1.92, linewidths *W*_FWHM,1_ = 0.77 mT, *W*_FWHM,2_ = 2.00 mT, and *W*_FWHM,3_ = 2.17 mT, pseudo-Voigt lines used with ratios (Lorentz = 0, Gauss = 1) *V*_1_ = 1.00, *V*_2_ = *V*_3_ = 0.50. Hyperfine coupling to one ^14^N nucleus was determined to be *A*_3_ = 43.6 × 10^−4^ cm^−1^ (4.9 mT). (Inset) CW X-band EPR spectrum of a 1 mM solution of 4 in THF at 293 K (black trace), and its simulation (red trace). Simulation parameters: *g*_iso_ = 1.95, linewidth *W*_FWHM,iso_ = 1.31 × 10^−4^ cm^−1^ GHz^−1^, pseudo-Voigt lines used with ratio (Lorentz = 0, Gauss = 1) *V*_iso_ = 0.50. (Right) Temperature-dependent SQUID magnetization data of polycrystalline 4 (black squares) and its simulation (red trace) with an applied magnetic field of 1 T (2–300 K), plotted as *μ*_eff_*vs. T*. Simulation parameters: *S*_1_ = *S*_2_ = 1/2, *J* = −1.23 cm^−1^, TIP = 1184 × 10^−6^ emu, |*D*_1_| = |*D*_2_| = 0.00 cm^−1^, *E*/*D*_1_ = *E*/*D*_2_ = 0.00, *g*_avg,1_ = *g*_avg,2_ = 2.00.

We also studied the spectroscopic and magnetic properties of 4 (Fig. 7). CW X-band EPR spectroscopic studies on a THF solution of 4 at 95 K revealed only one rhombic species (*g*_1_ = 1.98, *g*_2_ = 1.95, *g*_3_ = 1.93), which proves that both metal centers are electromagnetically identical. This resonance could be well simulated by including superhyperfine coupling to one ^14^N nucleus (*A*_3_ = 43.6 × 10^−4^ cm^−1^). Importantly, the EPR spectrum unequivocally confirms the presence of a Ti_2_^III,III^ core in solution, implying that a C–C bond-reforming event producing a hypothetical bridging oxalimidamide Ti_2_^IV,IV^ system such as “[3]^2+^” does not predominate under these experimental conditions. An additional RT measurement shows an isotropic signal at *g*_iso_ = 1.95, which corresponds to *g*_avg_ = 1.95 from the rhombic spectrum at 95 K and, thus, confirms the determined simulation parameters.

From SQUID magnetometric measurements conducted on a polycrystalline sample of 4, a magnetic moment of 2.46 *μ*_B_ at 300 K was determined, which is in excellent agreement with the theoretical value for a dinuclear system composed of two identical *S* = 1/2 ions. When cooling the sample, the magnetic moment remains practically constant until 25 K, after which it decreases to reach a final value of 1.85 *μ*_B_ at 2 K. Modelling of such decrease of magnetic moment yielded a coupling constant of *J* = −1.23 cm^−1^, due to antiferromagnetic coupling of the two Ti^III^ centers. It is noteworthy that although the Ti_2_^III,III^ centers in 3 and 4 are located at nearly identical spatial separations (5.5737(9) and 5.5339(6) Å, respectively), the magnetic interactions in these systems are strikingly different. Despite being a highly conjugated system, the oxalimidamide ligand makes the Ti^III^ centers in 3 behave as essentially isolated d^1^ ions, whereas the cumulenic carbodiimide ligands in 4 mediate antiferromagnetic coupling. The magnetically insulating nature of the oxalimidamide ligand may be traced to the SOMO1 and SOMO2 of 3, which display near-zero amplitude at the {C_2_N_4_} fragment and essentially possess pure titanium 3d character. By contrast, the SOMO1 and SOMO2 of 4 have a sizeable admixture of the carbon 2p orbitals of the central carbodiimide moiety (Fig. S29), thus mediating antiferromagnetic coupling of the two d^1^ ions *via* magnetic superexchange.

### Origin of C–C bond splitting upon oxidation of oxalimidamide complex 3 to form carbodiimide complex 4

The two energetically highest positioned molecular orbitals of 3 are SOMO1 and SOMO2. Conventional understanding would suggest that removal of electrons should occur from these essentially metal-centered orbitals. Nonetheless, chemical oxidation of 3, ultimately, leads to oxidative breaking of the central C–C bond of the oxalimidamide ligand. To better understand this apparent conundrum, we evaluated the energetics of the two possible isomeric dications, [(Tp^*t*Bu,Me^)Ti{1,3-μ_2_-NCNAd}_2_Ti(Tp^*t*Bu,Me^)]^2+^ (present in 4, with *S* = 1) as well as the hypothetical Ti_2_^IV,IV^ oxalimidamide dication, “[3]^2+^” (Fig. S28). Interestingly, the former dication is about 3.8 kcal mol^−1^ more stable than “[3]^2+^”, in line with the experimental outcome. Taken together, these theoretical findings indicate that the overall conversion of 3 to 4 most likely proceeds *via* a composite reaction sequence, consisting of first an electron transfer step, followed then by a structural rearrangement with internal redox chemistry between the oxalimidamide and the titanium centers. The underlying driving force for the C–C bond splitting event, therefore, seems to be predicated on the tendency for the dinuclear titanium core to preserve its Ti_2_^III,III^ valence state.

### Reductive coupling of an isocyanide with a vanadium nitride identifies a possible coupling intermediate

**Scheme 4:**
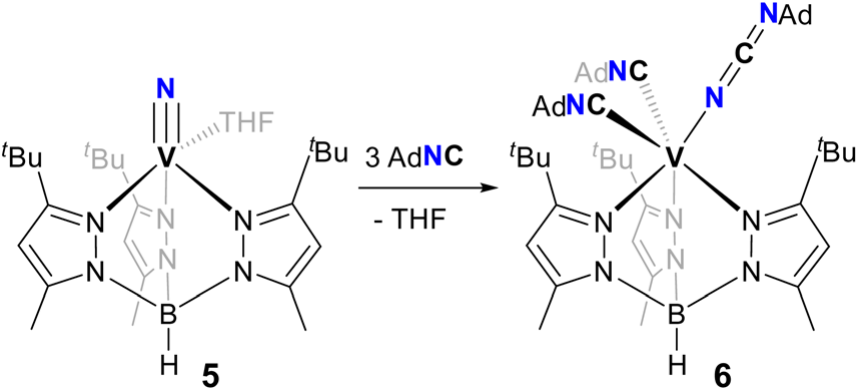
Synthesis of complex 6 upon addition of AdNC to the V^IV^ nitrido complex 5.

**Fig. 8:**
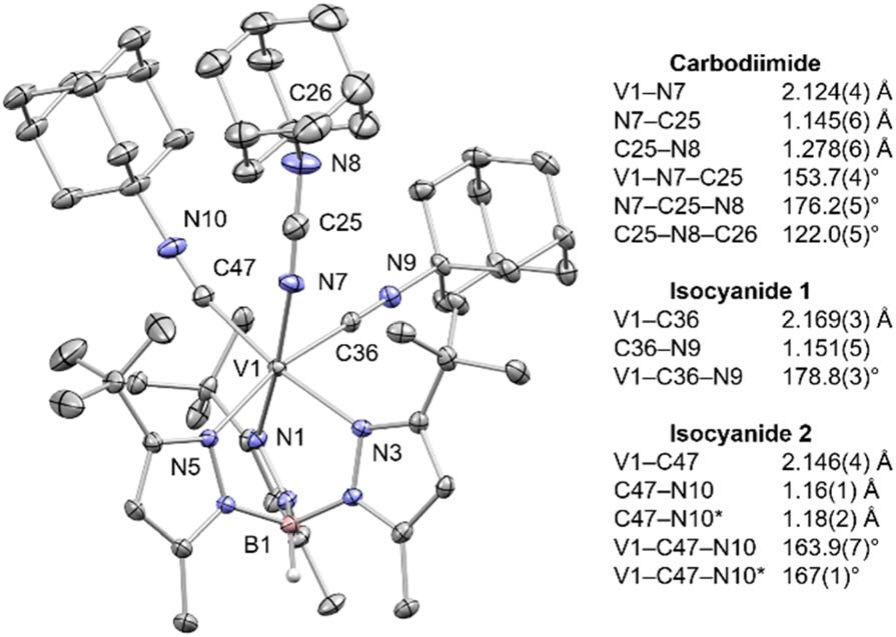
Thermal ellipsoid plot of 6 (50% probability). Disorder in one of the AdNC ligands is omitted for clarity.

To probe if structural analogs of the putative titanium(ii) intermediates, I(η^3^) or I(η^1^), could be trapped *via* azide deazotation and isocyanide coupling, starting from a less strongly reducing metal center such as vanadium(ii), we examined the *in situ* photolysis of the V^II^ azide precursor [(Tp^*t*Bu,Me^)V(μ-N_3_)_2_V(Tp^*t*Bu,Me^)] in THF (390 nm, 1 hour) to form the transient V^IV^ nitrido complex, [(Tp^*t*Bu,Me^)V≡N(THF)] (5).^23^ Subsequent treatment of 5 with >3 equiv. of AdNC led to optimized yields (69%) of a new paramagnetic species, identified to be the carbodiimide complex [(Tp^*t*Bu,Me^)V(η^1^-NCNAd)(CNAd)_2_] (6, Scheme 4). A solid-state structure of a single crystal of 6 confirmed the formation of a mononuclear V^II^ ion confined to a distorted octahedral geometry (Fig. 8). The most notable feature, however, is the presence of the linear carbodiimide (N7–C25–N8, 176.2(5)°) linkage resulting from the reductive coupling of AdNC with the nitrido ligand. Formation of a short N–C (1.145(6) Å) bond is accompanied by significant elongation of the former nitrido group, with the V–N distance increasing from 1.580(2) to 2.124(6) Å.^26^ Other metrical parameters are shown in Fig. 8. As expected, complex 6 is paramagnetic and a solution Evans magnetic susceptibility study indicates the presence of three unpaired electrons (*μ*_eff_ = 3.86 *μ*_B_, 25 °C, C_6_D_6_). Moreover, the solid-state IR spectrum showed the stretch of not only the B–H functionality (2572 cm^−1^), but also the isocyanide (2162 and 2142 cm^−1^) and carbodiimide moieties (2101 cm^−1^). While vanadium species 6 neither dimerizes to a structure akin to 4, nor undergoes C–C coupling to generate a vanadium analogue of 3, it provides structural support for the existence of the hypothesized, key intermediate, I(η^1^), predicted by simulations (Scheme 2, *vide supra*), given its analogous η^1^-binding mode and linear carbodiimide geometry. The lack of further coupling reactivity of 6 possibly stems from the protective auxiliary AdNC ligands that provide high kinetic stability to this vanadium derivative.

## Conclusions

In summary, we have shown how the combination of a tetrahedral Ti^II^ complex, an isocyanide, and an azide ion leads to a cascade of metal-coordination, C–N, and C–C bond-forming steps, which ultimately produce a disubstituted oxalimidamide ligand, {C_2_N_4_Ad_2_}^4−^, chelated between two Ti^III^ fragments (3). The ability of a dinuclear Ti_2_^II,II^ bis(carbodiimide) intermediate, I(μ_2_), to relay electrons from its metal centers and onto its ligand moieties was computationally identified to be a pivotal step in the radical coupling mechanism resulting in 3. The electronic structure of 3 comprises substantial π-donation from the unsubstituted oxalimidamide N-donors to each titanium center, which in turn confines the unpaired electrons to Ti 3d orbitals of δ symmetry in an essentially uncoupled magnetic ground state. Although electrochemical studies of 3 reveal a fully reversible two-electron redox wave, and in spite of the metallocentric character of its two SOMOs, chemical oxidation of this Ti_2_^III,III^ complex, ultimately, leads to rupture of the oxalimidamide, involving C–C bond scission and formation of two bridging carbodiimide {NCNAd}^−^ ligands. This oxidation product (4) retains a Ti_2_^III,III^ configuration and displays antiferromagnetic coupling due to magnetic superexchange mediated by the central C 2p orbitals of the carbodiimides. From a wider perspective, our studies showcase how a strongly reducing metal ion such as Ti^II^ converts small molecular precursors into a complex nitrogen-rich skeletal framework. The oxalimidamide product bears only two organic substituents, making it an enticing starting point for extended π-conjugated systems. Our coupling strategy depends critically on the identity of the metal; a tetrahedral V^II^ complex, thus, afforded a stable carbodiimide fragment, which did not react further. Finally, we note that the ability to make and break a covalent C–C σ-bond by the act of capturing or releasing electrons constitutes a means, not only of constructing complex organic skeletons, but also of storing electrical energy in kinetically stable chemical bonds.

## Disclaimer

The views expressed are purely those of the authors and may not in any circumstances be regarded as stating an official position of the ERCEA and the European Commission.

## Author contributions

MGJ and AR carried out the synthetic work. All authors contributed to the data collection and to writing the manuscript.

## Conflicts of interest

There are no conflicts to declare.

## Abbreviations

Ad: 1-Adamantyl
^*n*^Bu: Butyl
^*t*^Bu: *Tert*-butyl
Cp: Cyclopentadienyl
Cp*: Pentamethylcyclopentadienyl
Me: Methyl
Ph: Phenyl
Tp^*t*Bu,Me^: Hydrotris(3-*tert*-butyl-5-methylpyrazol-1-yl)borate

## Supplementary Material

SC-016-D5SC04824A-s001

SC-016-D5SC04824A-s002

## Data Availability

CCDC 2445536 (3), 2445537 (4) and 2445538 (6) contain the supplementary crystallographic data for this paper.^27–29^ All synthetic procedures, NMR, IR, UV-vis, EPR, magnetic, electrochemical, and crystallographic characterization data, as well as DFT studies are available in the SI. See DOI: https://doi.org/10.1039/d5sc04824a.
