# Supplementary material for: A titanium redox-switch enables reversible C–C bond forming and splitting reactions
Source: Chem Sci. 2025 Aug 22;16(38):17714–24. doi: 10.1039/d5sc04824a (PMC12406152; doi:10.1039/d5sc04824a)
Supplement: SC-016-D5SC04824A-s001 [file SC-016-D5SC04824A-s001.pdf]

Supplementary Information (SI) for Chemical Science.
This journal is © The Royal Society of Chemistry 2025

Supporting Information

for

A titanium redox-switch enables reversible C–C bond forming and splitting reactions

Mehrafshan G. Jafari,[a] Dominik Fehn,[b] Christian Sandoval-Pauker,[c] Michael R. Gau,[a] Karsten Meyer,[b],* Balazs Pinter,[c],[d],* Daniel J. Mindiola,[a],* Anders Reinholdt[a],[e],*

[a] *Department of Chemistry, University of Pennsylvania, 231 South 34th Street, Philadelphia, PA 19104 (USA). E-mail: mindiola@sas.upenn.edu*

[b] *Inorganic Chemistry, Department of Chemistry and Pharmacy, Friedrich-Alexander University (FAU) Erlangen-Nürnberg, 91058 Erlangen, Germany. Email: karsten.meyer@fau.de*

[c] *Department of Chemistry and Biochemistry, University of Texas at El Paso, El Paso, Texas 79968, USA. E-mail: pinter.balazs@gmail.com*

[d] *Current affiliation: European Research Council Executive Agency* #

[e] *Centre for Analysis and Synthesis, Department of Chemistry, Lund University, Naturvetarvägen 22, 22100 Lund, Sweden. E-mail: anders.reinholdt@chem.lu.se*

Disclaimer: The views expressed are purely those of the authors and may not in any circumstances be regarded as stating an official position of the ERCEA and the European Commission.

1 Contents

1 Contents **S2**

2 Materials and Methods **S3**

3 Synthetic Procedures **S6**

3.1 Synthesis of [(Tp^{tBu,Me})Ti{AdN(N)C–C(N)NAd}Ti(Tp^{tBu,Me})] (**3**) S6

3.2 Synthesis of [(Tp^{tBu,Me})Ti{1,3-μ_2-NCNAd}$_2$Ti(Tp^{tBu,Me})][B(C_6F_5)$_4$]$_2$ (**4**) S7

3.3 Synthesis of [(Tp^{tBu,Me})V(η^1-NCNAd)(CNAd)$_2$] (**6**) S8

4 NMR Spectroscopic Studies **S9**

4.1 NMR Spectral Data for [(Tp^{tBu,Me})Ti{AdN(N)C–C(N)NAd}Ti(Tp^{tBu,Me})] (**3**) S9

4.2 NMR Spectral Data for [(Tp^{tBu,Me})Ti{1,3-μ_2-NCNAd}$_2$Ti(Tp^{tBu,Me})][B(C_6F_5)$_4$]$_2$ (**4**) S10

4.3 NMR Spectral Data for [(Tp^{tBu,Me})V(η^1-NCNAd)(CNAd)$_2$] (**6**) S13

5 IR Spectroscopic Studies **S14**

5.1 IR Spectral Data for [(Tp^{tBu,Me})Ti{AdN(N)C–C(N)NAd}Ti(Tp^{tBu,Me})] (**3**) S14

5.2 IR Spectral Data for [(Tp^{tBu,Me})Ti{1,3-μ_2-NCNAd}$_2$Ti(Tp^{tBu,Me})][B(C_6F_5)$_4$]$_2$ (**4**) S15

5.3 IR Spectral Data for [(Tp^{tBu,Me})V(η^1-NCNAd)(CNAd)$_2$] (**6**) S16

6 UV-vis Spectroscopic Studies **S17**

6.1 UV-vis Spectral Data for [(Tp^{tBu,Me})Ti{AdN(N)C–C(N)NAd}Ti(Tp^{tBu,Me})] (**3**) S17

6.2 UV-vis Spectral Data for [(Tp^{tBu,Me})Ti{1,3-μ_2-NCNAd}$_2$Ti(Tp^{tBu,Me})][B(C_6F_5)$_4$]$_2$ (**4**) S18

6.3 UV-vis Spectral Data for [(Tp^{tBu,Me})V(η^1-NCNAd)(CNAd)$_2$] (**6**) S19

7 EPR Spectroscopic Studies **S20**

7.1 EPR Spectral Data for [(Tp^{tBu,Me})Ti{1,3-μ_2-NCNAd}$_2$Ti(Tp^{tBu,Me})][B(C_6F_5)$_4$]$_2$ (**4**) S20

8 SQUID Magnetometry **S22**

8.1 Magnetometric Data for [(Tp^{tBu,Me})Ti{AdN(N)C–C(N)NAd}Ti(Tp^{tBu,Me})] (**3**) S22

8.2 Magnetometric Data for [(Tp^{tBu,Me})Ti{1,3-μ_2-NCNAd}$_2$Ti(Tp^{tBu,Me})][B(C_6F_5)$_4$]$_2$ (**4**) S23

9 Electrochemical Studies **S24**

10 Crystallographic Data **S25**

11 Computational Studies **S26**

11.1 Computational Methodology S26

11.2 Computational Data S27

12 References **S155**

2 Materials and Methods

All synthetic operations were performed in M. Braun glove boxes under purified argon or nitrogen atmospheres (H_2O, O_2 < 1 ppm). Diethyl ether (Et_2O, Alfa Aesar), pentane (Fisher Scientific), toluene (Fisher Scientific), and tetrahydrofuran (THF, Fisher Scientific) were purchased from commercial vendors, thoroughly bubbled with argon, and made anhydrous by passage through columns of activated alumina in a Grubbs-type solvent system (JC Meyer). The anhydrous solvents were stored over 4 Å molecular sieves (Acros Organics). Benzene-d_6 and THF-d_8 (Cambridge Isotope Laboratories) were stored over potassium mirror overnight, sublimed/distilled by trap-to-trap transfer *in vacuo*, and degassed by freeze-pump-thaw cycles. Celite and 4 Å molecular sieves were activated under vacuum overnight at 200 °C. 1-adamantyl isonitrile (AdNC, TCI, >97.0%) and tetrabutylammonium azide ([^{n}Bu$_4$N][N$_3$], Fisher) were purchased from commercial vendors and used as received. [(Tp^{tBu,Me})TiCl] (**1**),[1] and [(Tp^{tBu,Me})V($\mu_{1,3}$-N$_3$)]$_2$,[2] were prepared according to published procedures.

The synthesis of [Fc][B(C_6F_5)] has been reported before;[3] in this study, a slightly modified procedure was employed: In a well ventilated fume hood, ferrocene ([Fc], 260 mg, 1400 mmol) was dissolved in 10 ml concentrated H_2SO_4, resulting in a dark blue solution due to the formation of ferrocenium (Fc^+) ion. The solution was added dropwise into 100 ml water and then filtered to remove an orange solid. In a separate container, K[B(C_6F_5)$_4$] (1.00 g, 1400 mmol, 1.0 eq.) was dissolved in 30 ml methanol, and added to the ferrocenium-containing solution, resulting in rapid separation of a blue precipitate of crude [Fc][B(C_6F_5)$_4$], which was collected by filtration and allowed to dry in the air overnight. The crude [Fc][B(C_6F_5)$_4$] was dissolved in CH_2Cl_2 (ca. 50 ml), filtered through a medium-porosity frit (removing a brown solid residue), and crystallized as dark blue needle crystals by adding hexanes (50–100 ml). The crystals were collected by filtration and dried *in vacuo* at 50 °C overnight. Yield of [Fc][B(C_6F_5)$_4$] · ⅛ CH_2Cl_2: 0.867 g, 990 µmol, 70.8% based on [Fc]. The presence of residual CH_2Cl_2 in the vacuum-dried crystals was certified by analyzing the samples by ^{1}H NMR spectroscopy. The residual CH_2Cl_2 was removed by transferring the sample into the glovebox, dissolving the [Fc][B(C_6F_5)$_4$] crystals in THF, recrystallizing by adding *ca.* 4 times the volume of pentane, washing with twice with pentane, and drying the sample *in vacuo*.

NMR spectroscopic studies were carried out using Bruker 400 MHz and 500 MHz spectrometers equipped with NMR tubes sealed with J. Young valves. ^{1}H chemical shifts are

referenced to residual solvent signals (C_6D_6: 7.16 ppm, THF-d_8: 1.72 & 3.58 ppm). For ^{11}B, 15% $BF_3{\cdot}Et_2O$ defines 0 ppm.

Solution-state magnetic susceptibility was measured by the Evans' method[4] using samples dissolved in C_6D_6 and with this deuterated solvent also being used as an external standard (kept in a capillary inside the NMR tube). Corrections for diamagnetism were made using tabulated Pascal's constants.[5]

$$\chi_M = 3\,\Delta\delta\,(4\pi\,c)^{-1} \qquad\qquad \mu_{eff} = (8\,\chi_M\,T)^{1/2}$$

χ_M is the molar susceptibility, $\Delta\delta$ is the change in chemical shift between benzene in the capillary and the NMR sample (ppm converted to 10^{-6}), and c is the concentration (cgs units). μ_{eff} is the effective magnetic moment, and T is the temperature.

IR spectroscopic studies were carried out using a Jasco FT/IR-4600 spectrometer with samples mounted between KBr windows. The sample chamber was flushed with Ar to prevent decomposition during measurements. Due to the high sensitivity of **3** toward air and moisture, this complex was suspended in NVH oil for the IR spectroscopic analysis.

UV-vis spectroscopic studies were carried out using a Cary 5000 Spectrometer equipped with 1 cm quartz cuvettes sealed with J. Young valves.

EPR spectroscopic studies were carried out using a JEOL continuous-wave spectrometer JES-FA200, equipped with an X-band Gunn diode oscillator bridge, a cylindric mode cavity, and a helium cryostat. If not stated otherwise, the samples were measured in the solid-state under nitrogen atmosphere in quartz glass EPR tubes at 293 and 95 K. The spectra shown were measured using the following parameters: microwave frequency = 8.943–8.945 GHz, modulation width = 1.0 mT, microwave power = 1.0 mW, modulation frequency = 100 kHz, time constant = 0.1 s. Data analysis and simulation was performed using the software "eview" and "esim", written by Dr. Eckhard Bill (MPI CEC, Mülheim/Ruhr), on the basis of a spin-Hamiltonian description of the electronic ground state:

$$\hat{H} = D\left(\hat{S}_z^2 - \frac{1}{3}S(S+1) + \frac{E}{D}\left(\hat{S}_x^2 - \hat{S}_y^2\right)\right) + \mu_B \underline{g}\vec{S}\ .$$

Here, S represents the total spin quantum number of the coupled system, D and E/D are the axial and rhombic zero-field parameters, respectively, and $\underline{g}$ is the g-matrix. Calculations are based on the S = 5/2 routines developed by Gaffney and Silverstone.[6] EPR linewidths, W, are given in units of mT or $\cdot\ 10^{-4}$ cm^{-1} GHz^{-1} at full width at half maximum (FWHM).

SQUID magnetometric studies were carried out on polycrystalline powdered samples (10.0–25.0 mg), loaded within a polycarbonate gel capsule inside a plastic straw or loaded and compressed into a quartz glass holder. The data were collected on a Quantum Design MPMS-3 SQUID magnetometer. The DC moment was recorded in the temperature range of 2–300 K with an applied DC field of 1 T. The DC moment was converted into molar magnetic susceptibility (χ_{M}) using the following formula (with H = magnetic field, n = moles of substance):

$$\chi_{\mathrm{M}} = \frac{DC\ moment}{H \cdot n}$$

Values of the magnetic susceptibility were corrected for core diamagnetism (χ_{dia}) of the sample, using tabulated Pascal's constants.[5] Effective magnetic moments (μ_{eff}) were calculated using the following formula (with temperature (T)):

$$\mu_{\mathrm{eff}} = 2.828 \cdot \sqrt{(\chi_{\mathrm{M}} - \chi_{\mathrm{dia}}) \cdot T}$$

For data simulation and analysis, the program "JulX2", written by Dr. Eckhard Bill (MPI CEC, Mülheim/Ruhr) was used.[1]

Electrochemical studies were carried out using a CH Instruments 620E Electrochemical Workstation equipped with a three-electrode system: a platinum working electrode, a platinum wire as counter electrode, and a silver wire as reference electrode. Measurements were carried out inside a N_2 filled glovebox. The potential was referenced to ferrocene, taking $[FeCp_2]^{0/+}$ as 0.0 V.

Crystallographic studies were carried out on single crystals, which were coated with NVH oil, mounted at the end of a cryoloop, and placed in the nitrogen cold stream of the diffractometer. Data were collected and processed using Rigaku XtaLAB Synergy-I,[7, 8] or Rigaku XtaLAB Synergy-S[8, 9] diffractometers (Mo K_{α} or Cu K_{α} radiation), operated through the manufacturer's software. The crystal structures were solved using SHELXT (intrinsic phasing) and refined using SHELXL-2018 (least squares),[10, 11] typically with data processing carried out in Olex2.[12]

Elemental analyses were carried out by Midwest Microlab, Inc (Indianapolis, Indiana, USA). According to our inquiry, analysis capsules were prepared in a glovebox with an O_2 level of *ca.* 40 ppm and then transferred outside for combustion. The extremely air- and moisture-sensitive low-valent, early transition metal complexes reported herein proved incompatible with the aforementioned analysis protocol, resulting in unsatisfactory CHN analyses.

3 Synthetic Procedures

3.1 Synthesis of [(Tp^{tBu,Me})Ti{AdN(N)C–C(N)NAd}Ti(Tp^{tBu,Me})] (3)

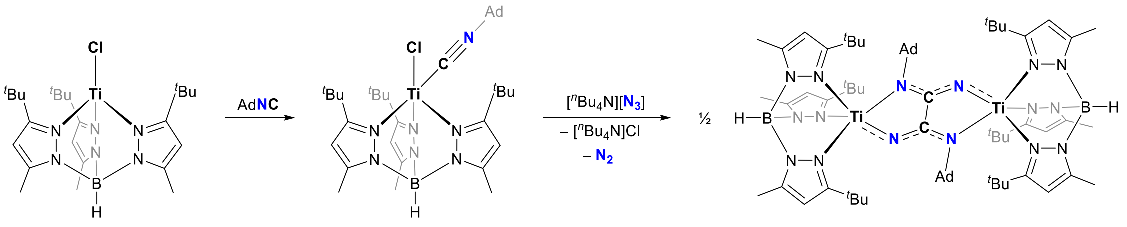

1 green **2** maroon **3** dark green

Under an Ar atmosphere, [(Tp^{tBu,Me})TiCl] (**1**, 59.7 mg, 118 μmol) and AdNC (19.0 mg, 118 μmol, 1.0 eq.) were dissolved in 0.5 ml toluene, resulting in an immediate color change due to the transformation of the green complex **1** to the maroon isocyanide complex [(Tp^{tBu,Me})TiCl(CNAd)] (**2**). The solution was chilled in the freezer of the glovebox (–35 °C), and then [^{n}Bu$_4$N][N$_3$] (33.9 mg, 119 μmol, 1.0 eq.) was added, resulting in effervescence and formation of a dark green solution. The reaction mixture was placed in the freezer at –35 °C over 2.5 hours, resulting in the formation of dark green needle-shaped crystals of [(Tp^{tBu,Me})Ti{AdN(N)C–C(N)NAd}Ti(Tp^{tBu,Me})] (**3**) as well as colorless, block-shaped crystals of [^{n}Bu$_4$N]Cl. The mother liquor was then pipetted off, and the crystals were washed with 2 × 0.5 ml toluene, and dried *in vacuo*. The mother liquor solution was also concentrated and layered with diethyl ether, which resulted in further precipitation of product. These combined crops were quickly washed with first with 1 ml diethyl ether, and then with 3 × 0.5 ml acetonitrile, and finally dried *in vacuo*, resulting in complete removal of the colorless crystals of [^{n}Bu$_4$N]Cl. Total yield of [(Tp^{tBu,Me})Ti{AdN(N)C–C(N)NAd}Ti(Tp^{tBu,Me})] (**3**): 28.1 mg, 21.7 μmol, 36.9% based on the amount of titanium in the monomeric starting material. **^{1}H NMR**, 500 MHz, THF-d_8, δ (ppm): 29.29 (FWHM = 1300 Hz), 26.27 (FWHM = 950 Hz), 5.28 (FWHM = 71 Hz), 4.79 (FWHM = 80 Hz), 0.71 (FWHM = 55 Hz), 0.27 (FWHM = 280 Hz), –0.40 (FWHM = 160 Hz). **IR**, solid, ν (cm^{-1}): 2546 (B–H).

3.2 Synthesis of [(Tp^{tBu,Me})Ti{1,3-μ$_2$-NCNAd}$_2$Ti(Tp^{tBu,Me})][B(C_6F_5)$_4$]$_2$ (4)

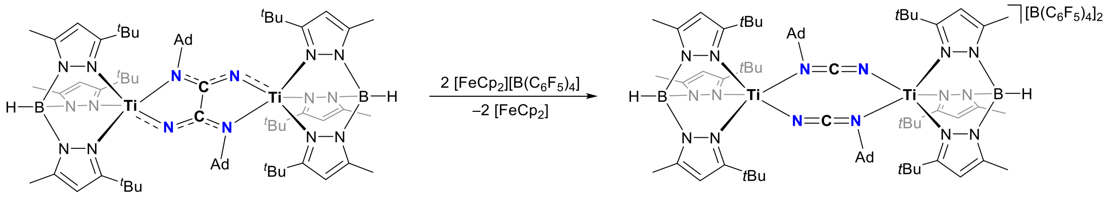

3 dark green **4** orange

Under a N_2 atmosphere, [(Tp^{tBu,Me})Ti(NCNAd)$_2$Ti(Tp^{tBu,Me})] (**3**, 13.0 mg, 10.1 μmol) was dissolved in *ca.* 2 ml THF. To the solution was added [Fc][B(C_6F_5)$_4$] (17.4 mg, 20.1 μmol, 2.0 eq.), upon which the solution immediately turned in color from green to orange. After 10 minutes of stirring the reaction mixture, and the volatiles were removed *in vacuo*. The orange residue was washed with pentane until the pentane extracts were colorless (initially orange due to dissolved ferrocene). The product was dissolved in THF and filtered through Celite. The solution was concentrated under vacuum, layered with pentane, and was left inside the freezer at –35 °C to allow large orange needle crystals of [(Tp^{tBu,Me})Ti{1,3-μ$_2$-NCNAd}$_2$Ti(Tp^{tBu,Me})][B(C_6F_5)$_4$]$_2$ (**4**) to grow. The mother liquor was pipetted off, and the product was dried *in vacuo*. A second crop of crystals was collected by further concentration of the mother liquor. Total yield of [(Tp^{tBu,Me})Ti{1,3-μ$_2$-NCNAd}$_2$Ti(Tp^{tBu,Me})][B(C_6F_5)$_4$]$_2$ (**4**): 24.0 mg, 9.05 μmol, 90.0% based on **3**. Crystals suitable for X-ray crystallography separated from a THF solution of **4** stored at –35 °C, using toluene as the sorbent. **^{1}H NMR**, 400 MHz, THF-d_8, δ (ppm): 18.15 (FWHM = 350 Hz), 14.45 (FWHM = 6 Hz), 11.99 (FWHM = 120 Hz), 2.54 (FWHM = 25 Hz), 2.19 (FWHM = 23 Hz), 2.09 (FWHM = 13 Hz), 1.68 (FWHM = 12 Hz), 1.39 (FWHM = 5 Hz), 0.19 (FWHM = 245 Hz). **^{11}B{^{1}H} NMR**, 128 MHz, THF-d_8, δ (ppm): –17.09. **^{19}F NMR**, 376 MHz, THF-d_8, δ (ppm): –132.67, –165.02, –168.46. **IR**, solid, ν (cm^{-1}): 2566 (B–H), 2243 (N=C=N), 2119 (N=C=N).

3.3 Synthesis of [(Tp^{tBu,Me})V(η^1-NCNAd)(CNAd)$_2$] (6)

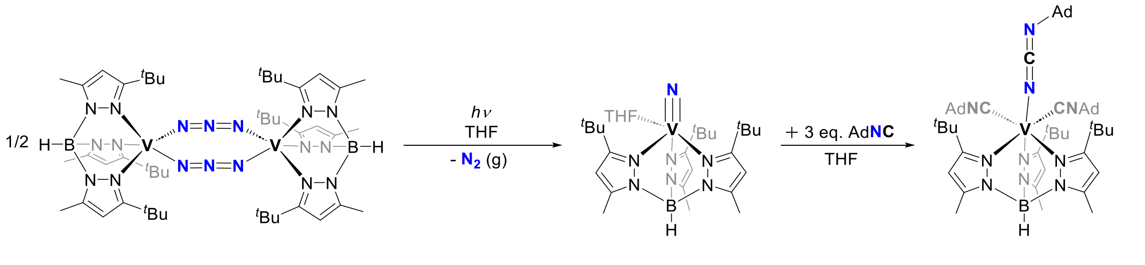

Under a N_2 atmosphere, [(Tp^{tBu,Me})V(μ$_{1,3}$-N_3)]$_2$ (34.2 mg, 33.1 μmol, ½ eq.) was dissolved in *ca.* 30 ml THF and transferred into a thick-walled pressure vessel. The pressure vessel was taken out of the glovebox and was photolyzed for 1 hour using a Kessil 390 nm lamp, upon which the green solution of the azide complex changed in color to afford an orange-colored solution of nitrido complex **5** *in situ*. Thereafter, the pressure vessel was transferred back into the glovebox, and to the solution of **5** in THF was added AdNC (33.7 mg, 209 μmol, 3.1 eq. isonitrile for each vanadium), upon which the solution immediately changed in color from orange to blue-green. After 15 minutes, the volatiles were removed *in vacuo*, and the product was washed with a small amount of cold pentane (*ca.* 1 ml). The product was then dissolved in diethyl ether (*ca.* 2 ml) and filtered through Celite and concentrated under vacuum (to *ca.* 1 ml), upon which blue crystal blocks of [(Tp^{tBu,Me})V(η^1-NCNAd)(CNAd)$_2$] (**6**) started to grow. The solution was left inside the freezer (–35 °C) for 4 hours, allowing the crystals to grow more, then the mother liquor was transferred to another vial and the crystals were washed with cold pentane (*ca.* 1 ml), resulting in the isolation of 40 mg of **6**. Thereafter, blue crystal plates were put under vacuum to remove volatiles, and one more crop of crystals was grown from the blue-green mother liquor solution. Total yield of [(Tp^{tBu,Me})V(η^1-NCNAd)(CNAd)$_2$] (**6**): 45.0 mg, 46.3 μmol, 69.9% based on the amount of vanadium in the dimeric starting material. Crystals suitable for X-ray crystallography were grown by concentrating a diethyl ether solution of **6** at –35 °C using toluene as a sorbent. **^{1}H NMR**, 400 MHz, C_6D_6, δ (ppm): 51.16 (FWHM = 3700 Hz), 39.36 (FWHM = 930 Hz), –4.10 (FWHM = 2100 Hz). **Magnetic moment** (Evans' method, C_6D_6, 300 K): 3.86 μ_B. **IR**, solid, ν (cm^{-1}): 2572 (B–H), 2162 (C≡N), 2142 (C≡N), 2101 (N=C=N).

4 NMR Spectroscopic Studies

4.1 NMR Spectral Data for [(Tp^{tBu,Me})Ti{AdN(N)C–C(N)NAd}Ti(Tp^{tBu,Me})] (3)

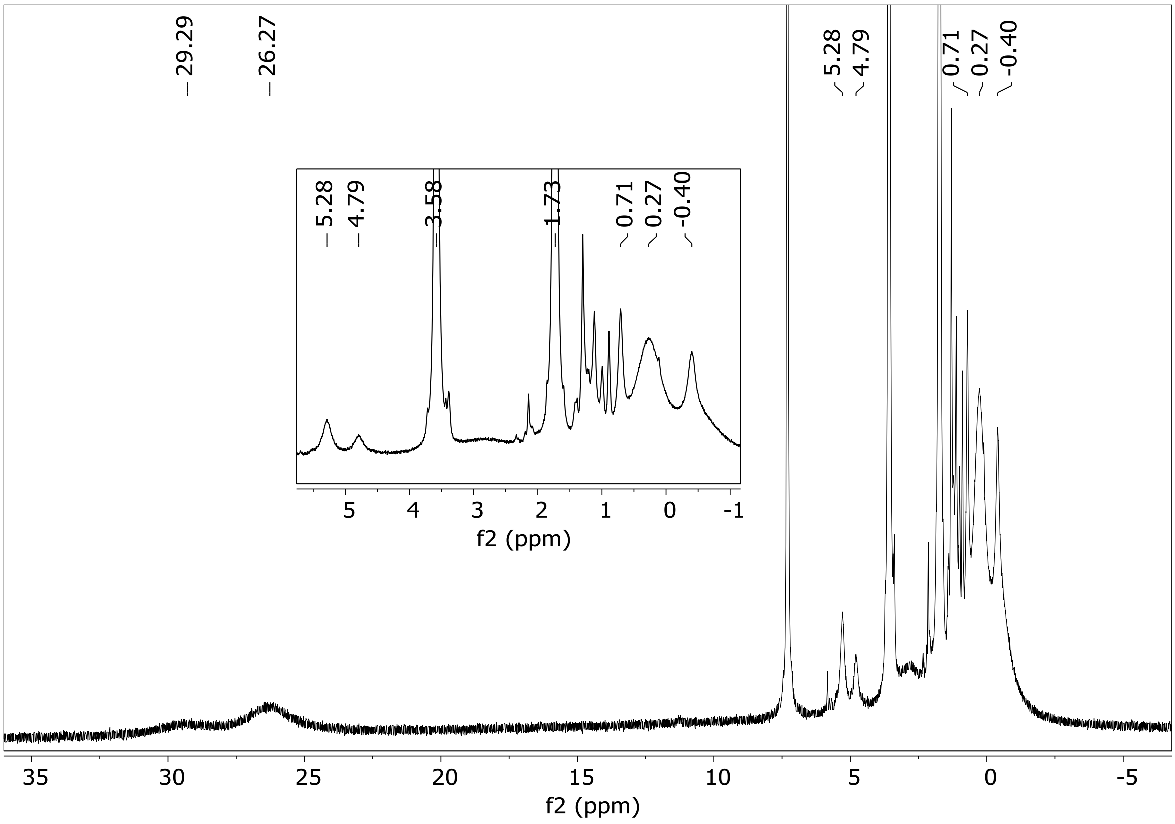

Figure S1. ^{1}H NMR spectrum of [(Tp^{tBu,Me})Ti{AdN(N)C–C(N)NAd}Ti(Tp^{tBu,Me})] (**3**) in THF-d_8 (residual peaks at 1.73 and 3.58 ppm). The relative integrals (1:2) of the resonances at 29.29 & 26.27 ppm, at 5.28 & 4.79 ppm, and at 0.27 & –0.40 ppm are indicative of local C_s symmetry about the titanium ion. The resonances between 0.89 ppm and 1.31 ppm are due to a trace of pentane.

4.2 NMR Spectral Data for [(Tp^{tBu,Me})Ti{1,3-μ_2-NCNAd}$_2$Ti(Tp^{tBu,Me})][B(C_6F_5)$_4$]$_2$ (4)

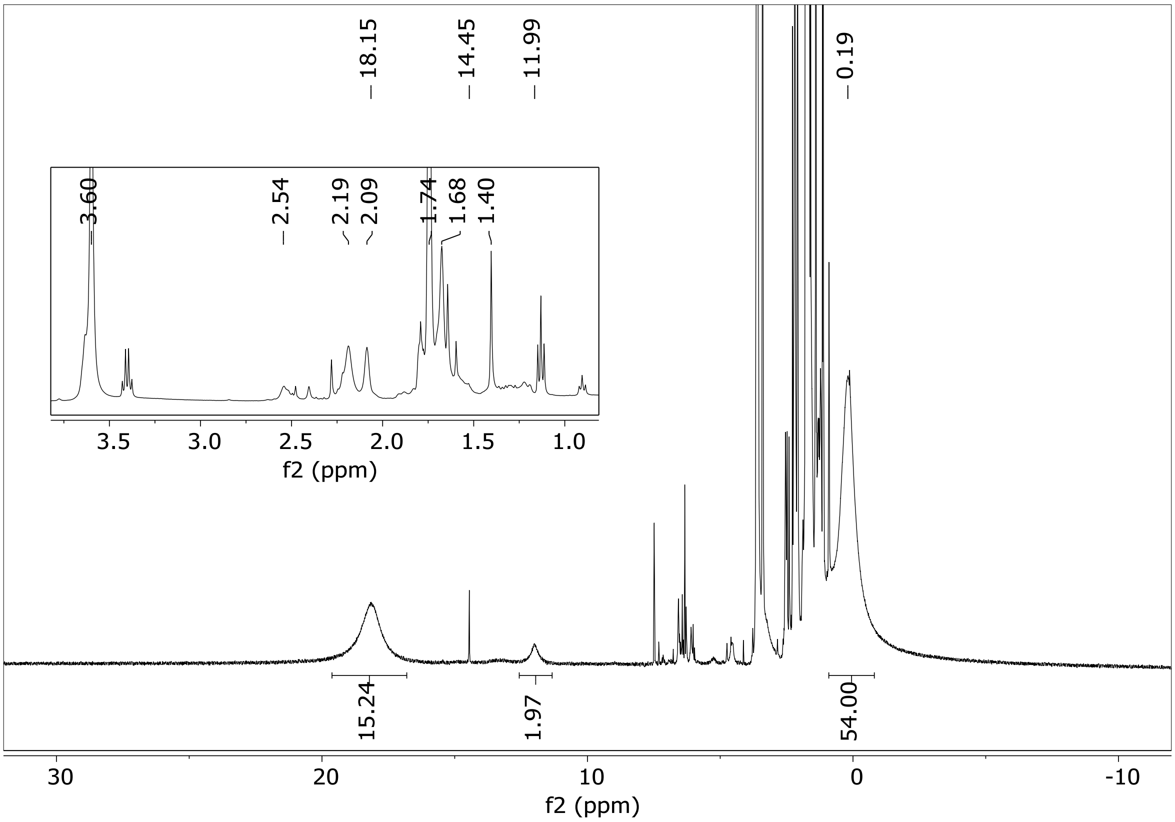

Figure S2. ^{1}H NMR spectrum of [(Tp^{tBu,Me})Ti{1,3-μ_2-NCNAd}$_2$Ti(Tp^{tBu,Me})][B(C_6F_5)$_4$]$_2$ (**4**) in THF-d_8 (residual peaks at 1.74 and 3.60 ppm). Based on their relative integrals, the resonances at 18.15 ppm (Me), 11.99 ppm (pyrazolyl-CH), and 0.19 ppm (^{t}Bu) can be assigned to the Tp^{tBu,Me} ligand. The resonances at 1.13 ppm and 3.41 ppm (diethyl ether) and at 0.90 ppm and 1.31 ppm (pentane) are due to traces of solvent.

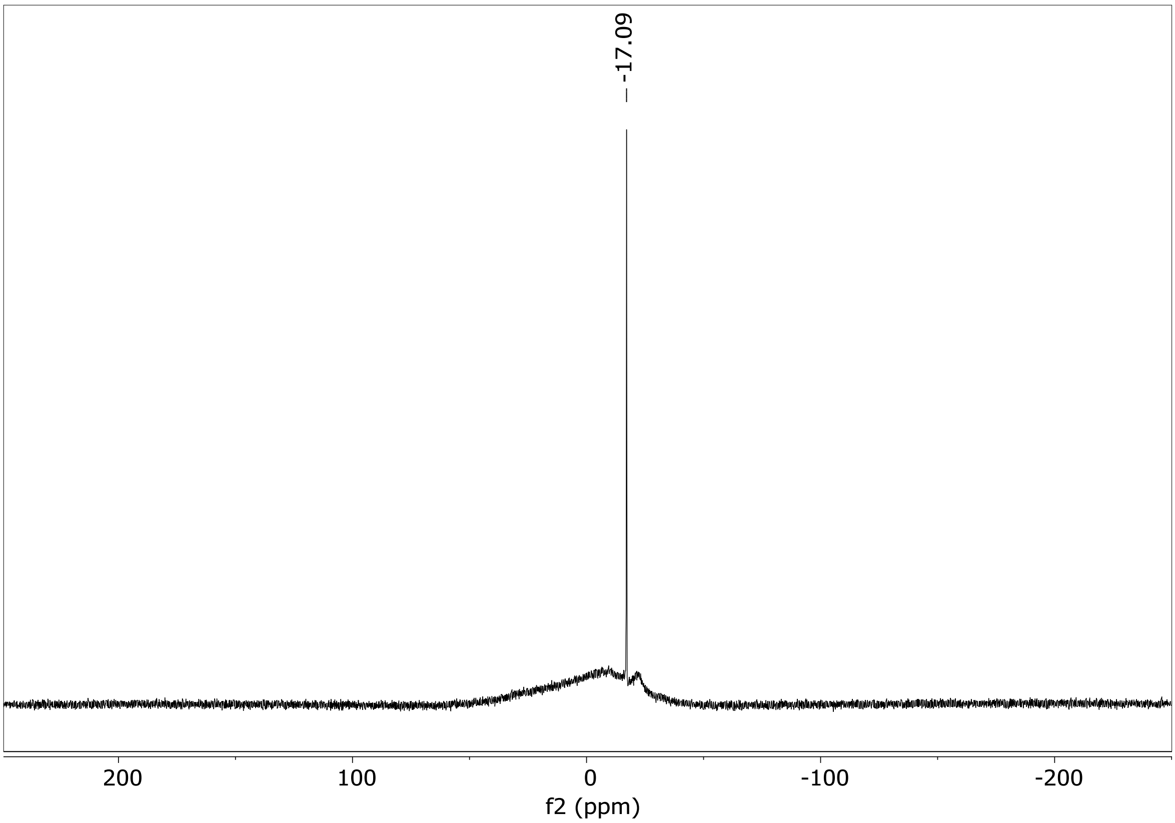

Figure S3. $^{11}B\{^{1}H\}$ NMR spectrum of $[(Tp^{tBu,Me})Ti\{1,3\text{-}\mu_2\text{-}NCNAd\}_2Ti(Tp^{tBu,Me})][B(C_6F_5)_4]_2$ (**4**) in THF-d_8.

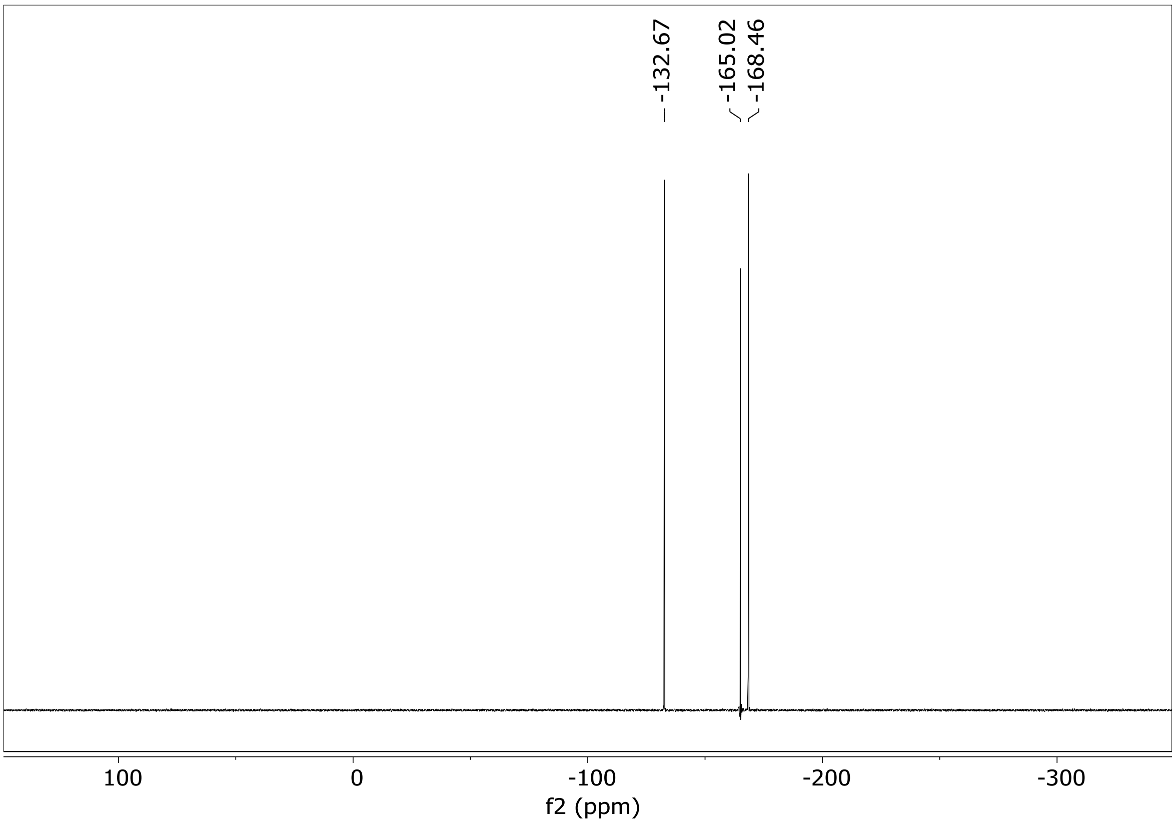

Figure S4. ^{19}F NMR spectrum of [(Tp^{tBu,Me})Ti{1,3-μ$_2$-NCNAd}$_2$Ti(Tp^{tBu,Me})][B(C$_6$F$_5$)$_4$]$_2$ (**4**) in THF-d_8.

4.3 NMR Spectral Data for [(Tp^{tBu,Me})V(η^1-NCNAd)(CNAd)$_2$] (6)

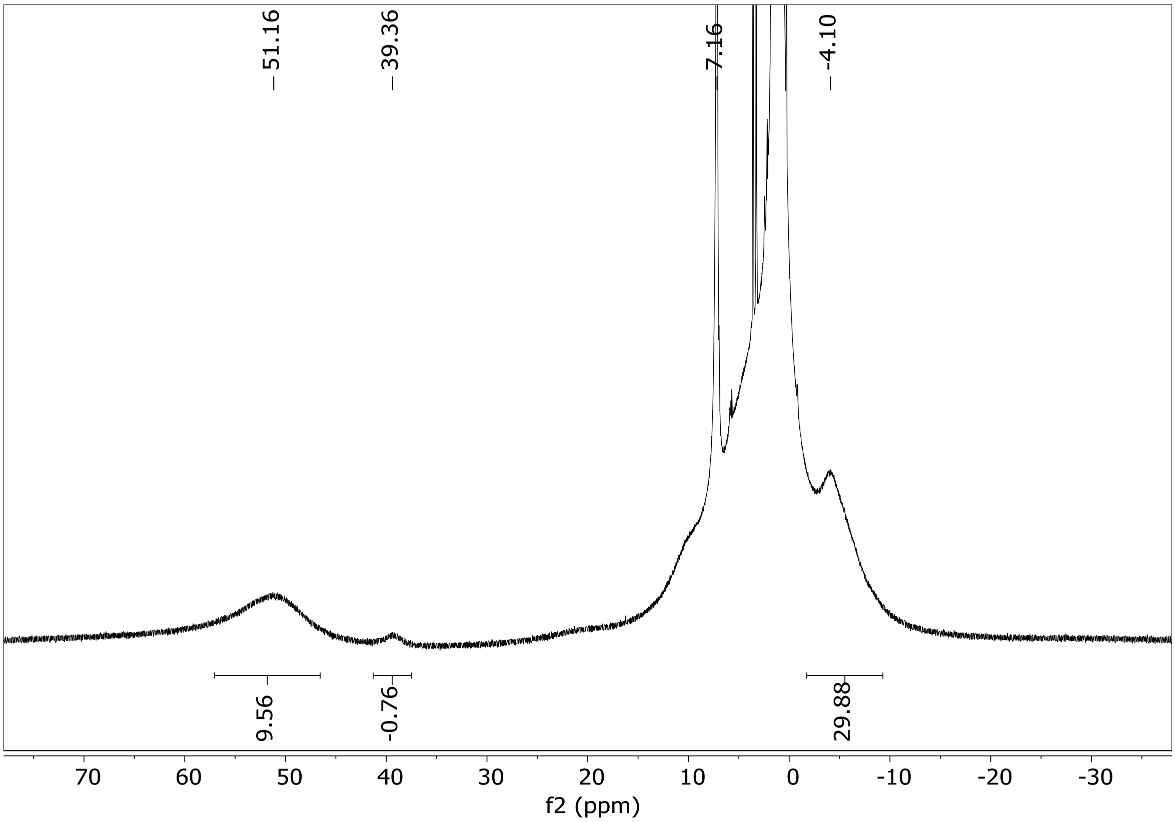

Figure S5. ^{1}H NMR spectrum of [(Tp^{tBu,Me})V(η^1-NCNAd)(CNAd)$_2$] (**6**) in C_6D_6 (residual peak at 7.16 ppm). Resonances that can be reliably assigned to the Tp^{tBu,Me} ligand are highlighted: Based on their relative integrals, 51.16 ppm (Me), 39.36 ppm (pyrazolyl-CH), and –4.10 ppm (^{t}Bu) can be assigned to the Tp^{tBu,Me} ligand.

5 IR Spectroscopic Studies

5.1 IR Spectral Data for [(Tp^{tBu,Me})Ti{AdN(N)C–C(N)NAd}Ti(Tp^{tBu,Me})] (3)

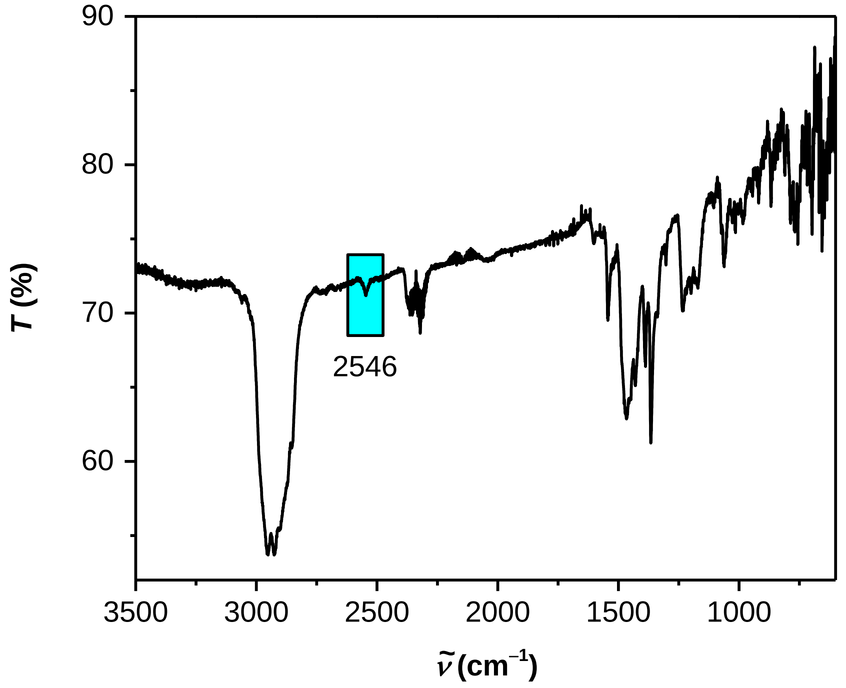

Figure S6. IR spectrum of solid [(Tp^{tBu,Me})Ti{AdN(N)C–C(N)NAd}Ti(Tp^{tBu,Me})] (**3**) suspended in NVH oil and pressed between KBr windows.

5.2 IR Spectral Data for [(Tp^{tBu,Me})Ti{1,3-μ$_2$-NCNAd}$_2$Ti(Tp^{tBu,Me})][B(C_6F_5)$_4$]$_2$ (4)

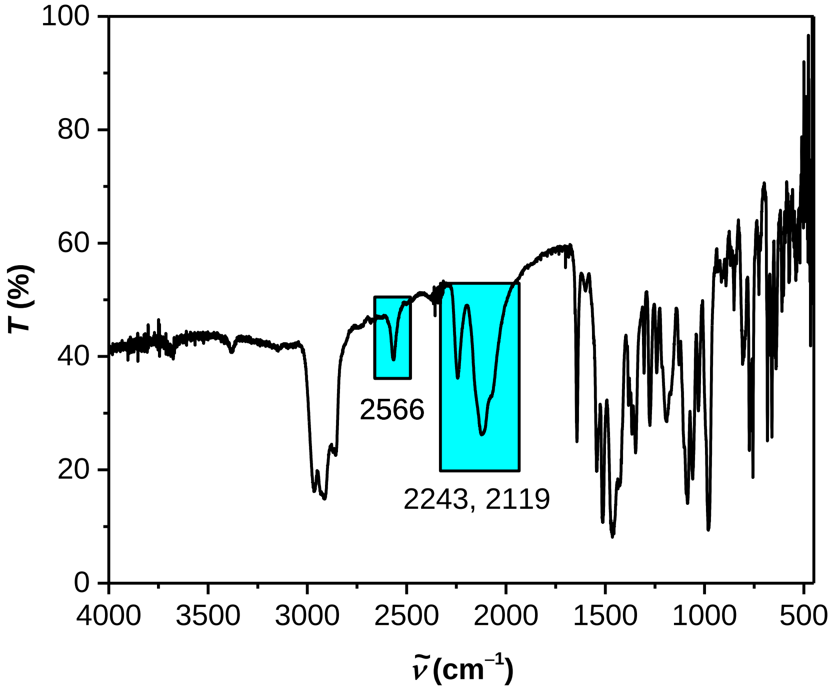

Figure S7. IR spectrum of solid [(Tp^{tBu,Me})Ti{1,3-μ$_2$-NCNAd}$_2$Ti(Tp^{tBu,Me})][B(C_6F_5)$_4$]$_2$ (**4**) pressed between KBr windows.

5.3 IR Spectral Data for [(Tp^{tBu,Me})V(η^1-NCNAd)(CNAd)$_2$] (6)

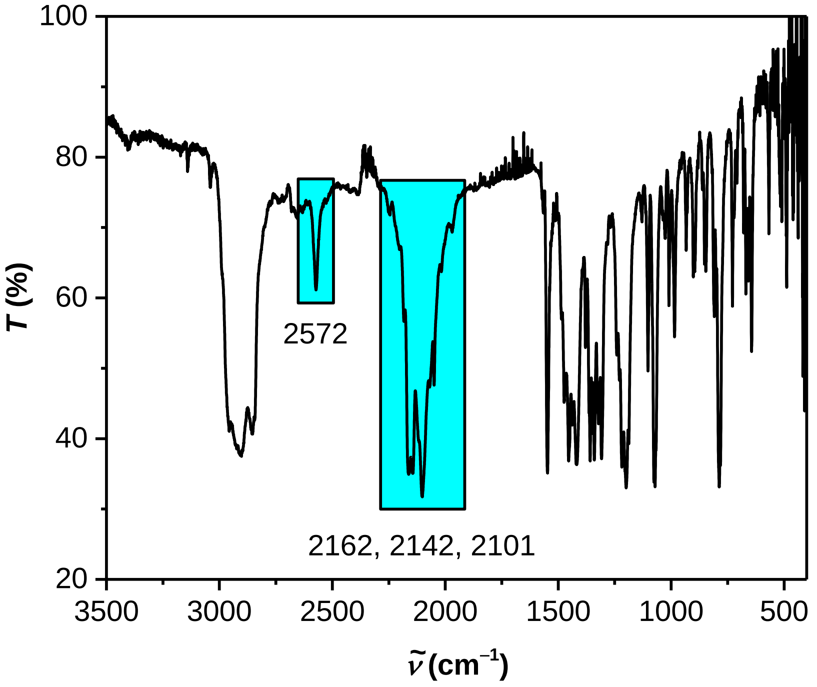

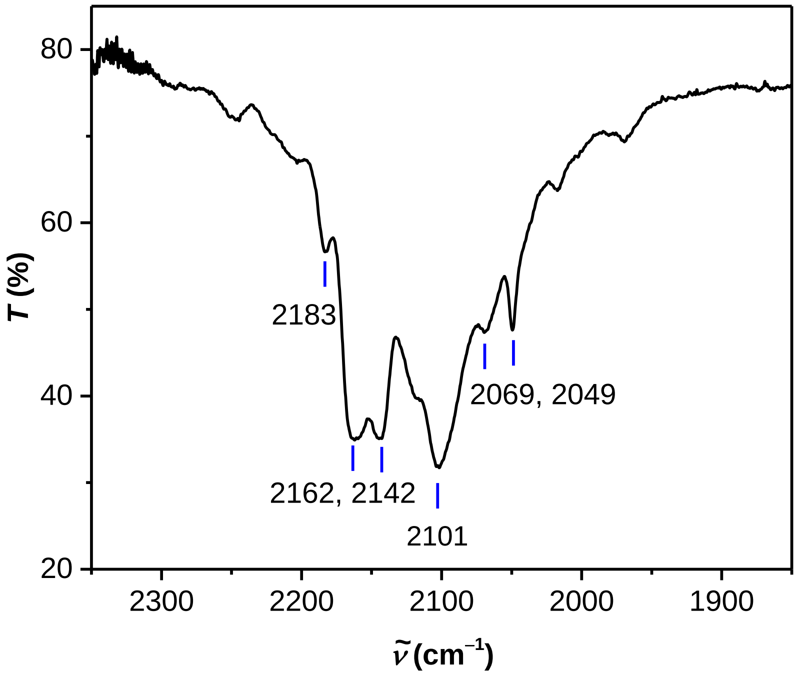

Figure S8. IR spectrum of solid [(Tp^{tBu,Me})V(η^1-NCNAd)(CNAd)$_2$] (**6**) pressed between KBr windows; full spectrum (*top*), zoom on the Ad**N≡C** and Ad**N=C=N**$^-$ stretching region (*bottom*).

6 UV-vis Spectroscopic Studies

6.1 UV-vis Spectral Data for [(Tp$^{t\text{Bu,Me}}$)Ti{AdN(N)C–C(N)NAd}Ti(Tp$^{t\text{Bu,Me}}$)] (3)

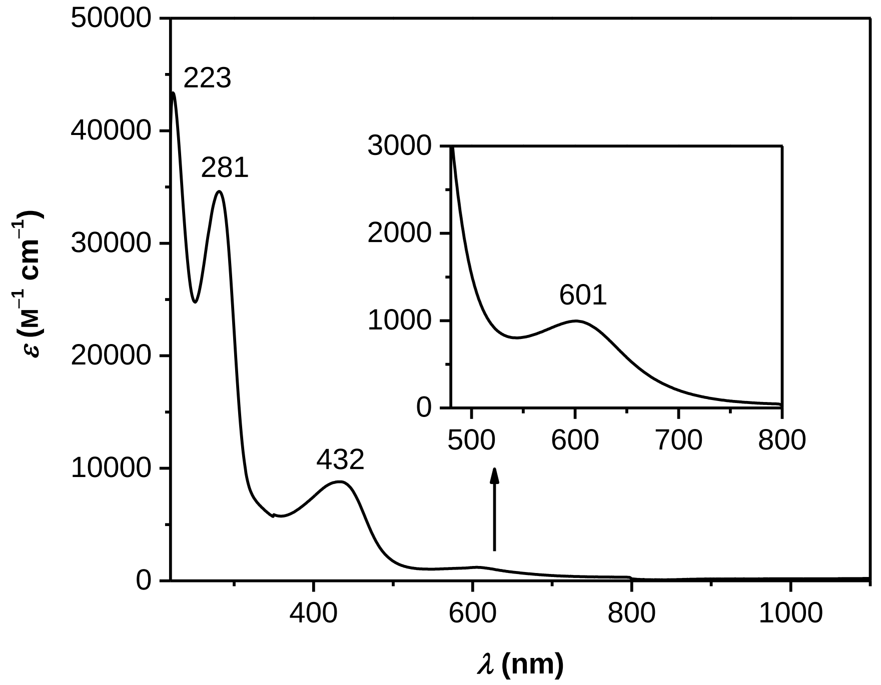

Figure S9. UV-vis spectrum of [(Tp$^{t\text{Bu,Me}}$)Ti{AdN(N)C–C(N)NAd}Ti(Tp$^{t\text{Bu,Me}}$)] (**3**) in THF. Lamp changeover at 350 nm, grating changeover at 800 nm. In order to best exploit the dynamic range of the UV-vis spectrometer, and to verify that **3** obeys Lambert-Beer's law, spectra were recorded at two concentrations. The full spectrum was recorded on a $4.1 \cdot 10^{-5}$ M solution, and the insert was recorded on a $3.2 \cdot 10^{-4}$ M solution of **3**.

6.2 UV-vis Spectral Data for [(Tp^{tBu,Me})Ti{1,3-μ_2-NCNAd}$_2$Ti(Tp^{tBu,Me})][B(C_6F_5)$_4$]$_2$ (4)

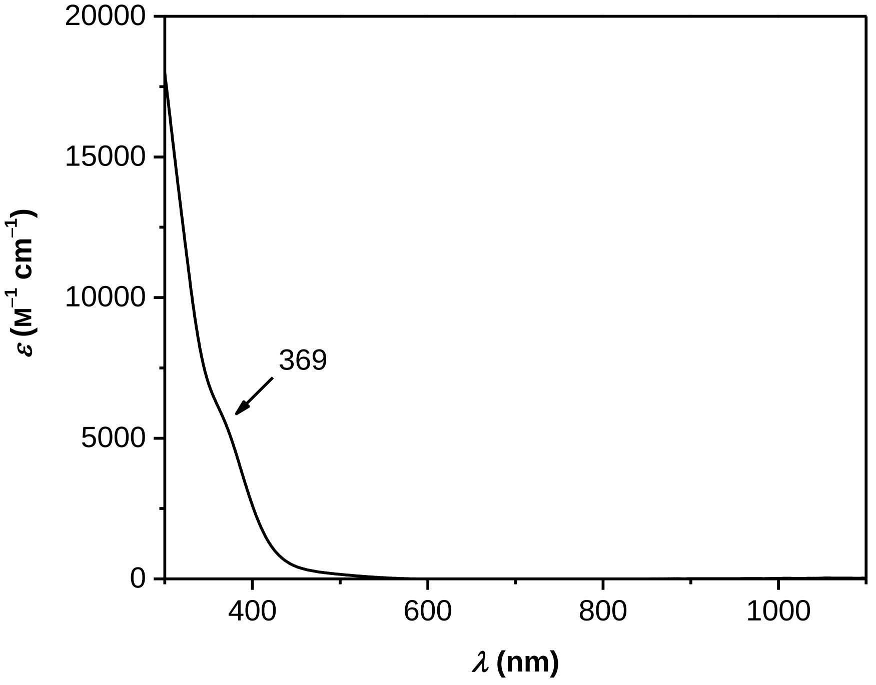

Figure S10. UV-vis spectrum of [(Tp^{tBu,Me})Ti{1,3-μ_2-NCNAd}$_2$Ti(Tp^{tBu,Me})][B(C_6F_5)$_4$]$_2$ (**4**) in THF. Lamp changeover at 350 nm, grating changeover at 800 nm. The full spectrum was recorded on a $1.02 \cdot 10^{-4}$ M solution of **6**.

6.3 UV-vis Spectral Data for [(Tp^{tBu,Me})V(η^1-NCNAd)(CNAd)$_2$] (6)

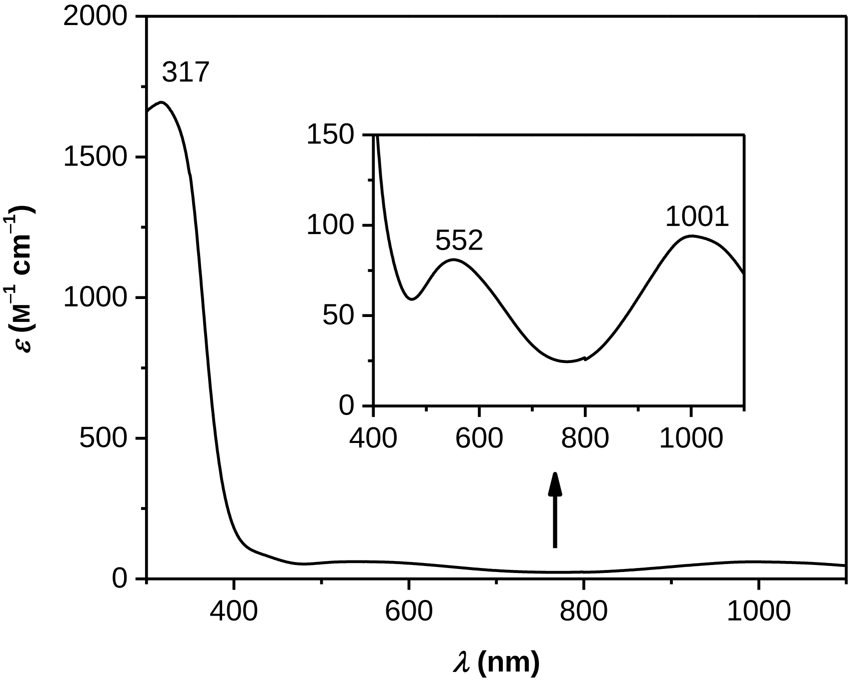

Figure S11. UV-vis spectrum of [(Tp^{tBu,Me})V(η^1-NCNAd)(CNAd)$_2$] (**6**) in toluene. Lamp changeover at 350 nm, grating changeover at 800 nm. In order to best exploit the dynamic range of the UV-vis spectrometer, and to verify that **6** obeys Lambert-Beer's law, spectra were recorded at two concentrations: The full spectrum was recorded on a $1.38 \cdot 10^{-3}$ M solution, and the insert was recorded on a $1.02 \cdot 10^{-2}$ M solution of **6**.

7 EPR Spectroscopic Studies

7.1 X-band EPR Spectral Data for [(Tp^{tBu,Me})Ti{1,3-μ$_2$-NCNAd}$_2$Ti(Tp^{tBu,Me})][B(C_6F_5)$_4$]$_2$ (4)

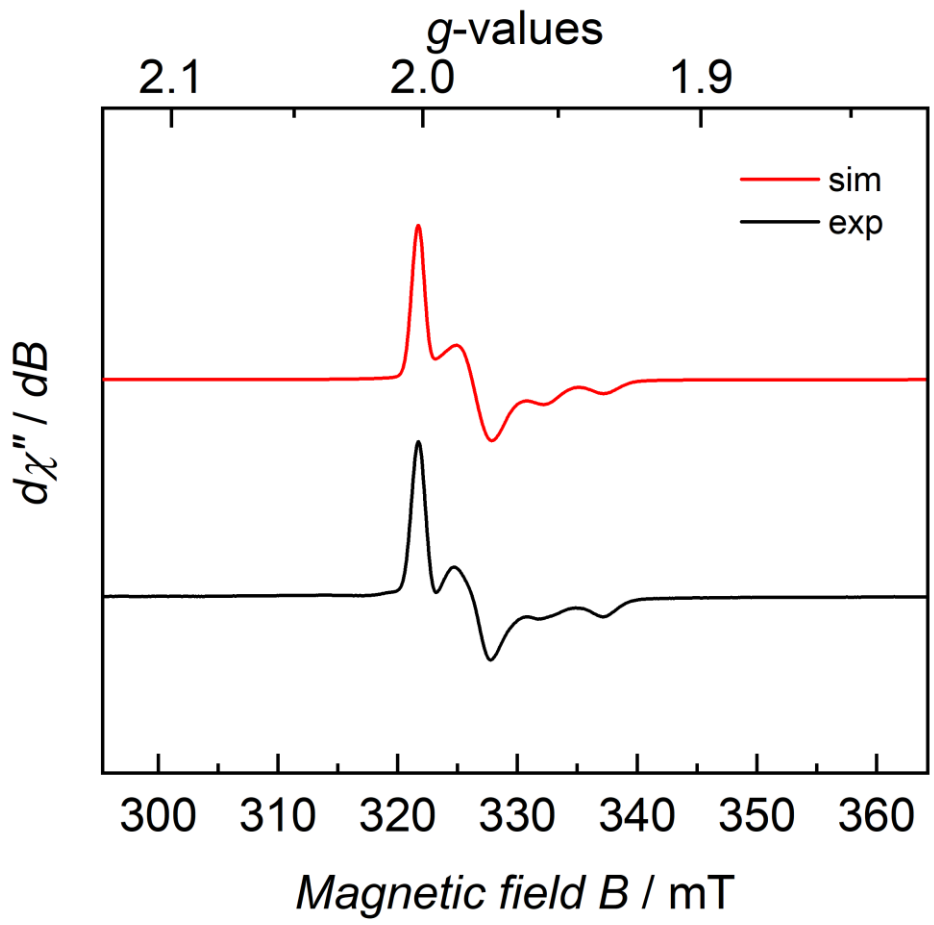

Figure S12: [(Tp^{tBu,Me})Ti{1,3-μ$_2$-NCNAd}$_2$Ti(Tp^{tBu,Me})][B(C_6F_5)$_4$]$_2$ (**4**) studied by CW X-band EPR spectroscopy as a 1 mM solution in THF at 95 K (black trace), and its simulation (red trace). Experimental conditions: microwave frequency ν = 8.945 GHz, modulation amplitude = 1.0 mT, microwave power = 1.0 mW, modulation frequency = 100 kHz, time constant = 0.1 s. Simulation parameters: effective g-values g_1 = 1.98, g_2 = 1.95, and g_3 = 1.92, linewidths $W_{FWHM,1}$ = 0.77 mT, $W_{FWHM,2}$ = 2.00 mT, and $W_{FWHM,3}$ = 2.17 mT, pseudo-Voigt lines used with ratios (Lorentz = 0, Gauss = 1) V_1 = 1.00, V_2 = V_3 = 0.50. Hyperfine coupling to one ^{14}N (I = 1, 99.6% natural abundance) nucleus was determined as A_3 = 43.6 · 10^{-4} cm^{-1} (4.9 mT).

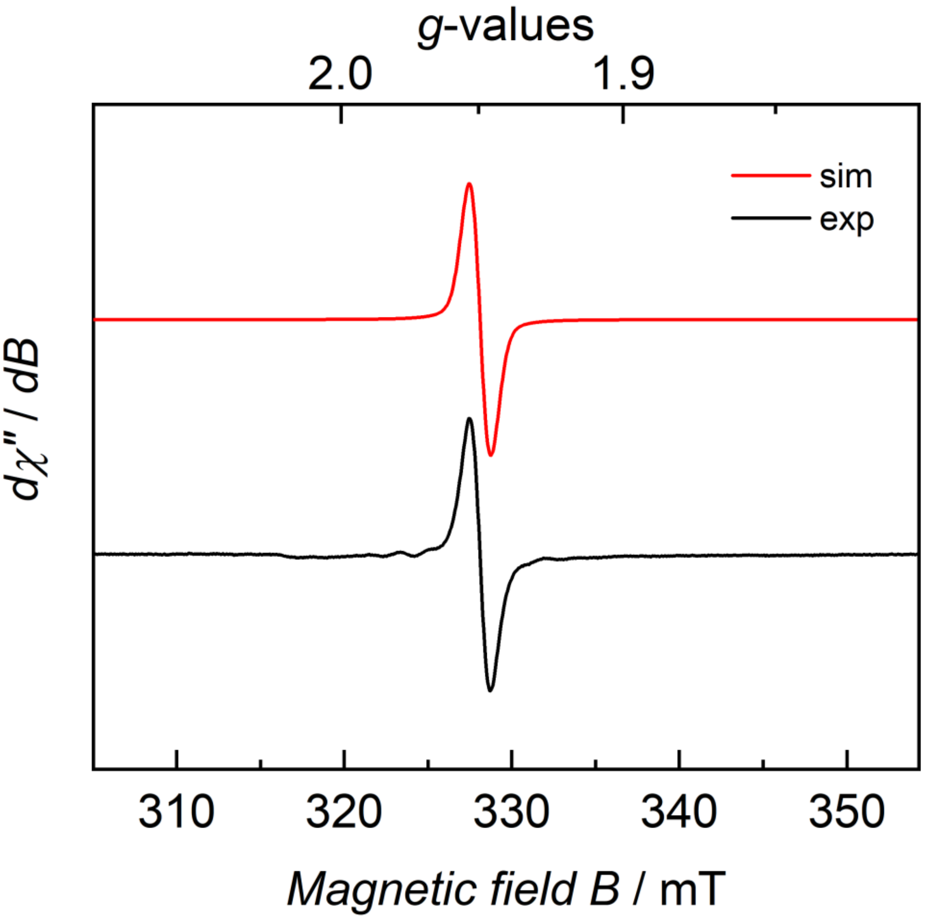

Figure S13: [(Tp^{tBu,Me})Ti{1,3-μ$_2$-NCNAd}$_2$Ti(Tp^{tBu,Me})][B(C_6F_5)$_4$]$_2$ (**4**) studied by CW X-band EPR spectroscopy as a 1 mM solution in THF at 293 K (black trace), and its simulation (red trace). Experimental conditions: microwave frequency ν = 8.943 GHz, modulation amplitude = 1.0 mT, microwave power = 1.0 mW, modulation frequency = 100 kHz, time constant = 0.1 s. Simulation parameters: effective g-value g_{iso} = 1.95, linewidth $W_{FWHM,iso}$ = 1.31 · 10^{-4} cm^{-1} GHz^{-1}, pseudo-Voigt lines used with ratio (Lorentz = 0, Gauss = 1) V_{iso} = 0.50.

8 SQUID Magnetometric Studies

8.1 Magnetometric Data for [(Tp^{tBu,Me})Ti{AdN(N)C–C(N)NAd}Ti(Tp^{tBu,Me})] (3)

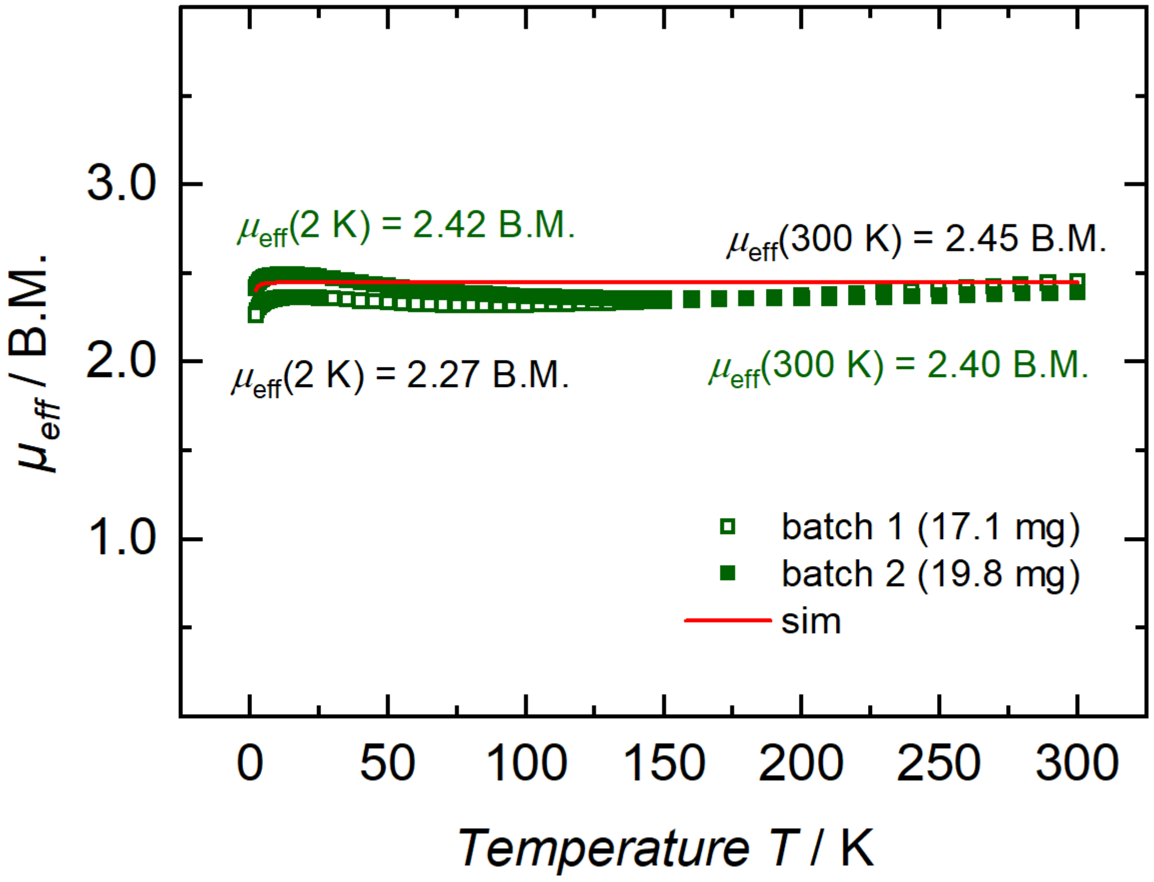

Figure S14. Temperature-dependent SQUID magnetization data of two independently synthesized batches of polycrystalline **3** (green squares) and a simulation (red trace), recorded with an applied magnetic field of 1 T (2–300 K), plotted as μ_{eff} *vs.* T. Simulation parameters for an idealized, uncoupled d^1-d^1 radical system: $S_1 = S_2 = 1/2$, $J = 0.00$ cm^{-1}, $|D_1| = |D_2| = 0.00$ cm^{-1}, $E/D_1 = E/D_2 = 0.00$, $g_{avg,1} = g_{avg,2} = 2.00$.

8.2 Magnetometric Data for [(Tp^{tBu,Me})Ti{1,3-μ$_2$-NCNAd}$_2$Ti(Tp^{tBu,Me})][B(C_6F_5)$_4$]$_2$ (4)

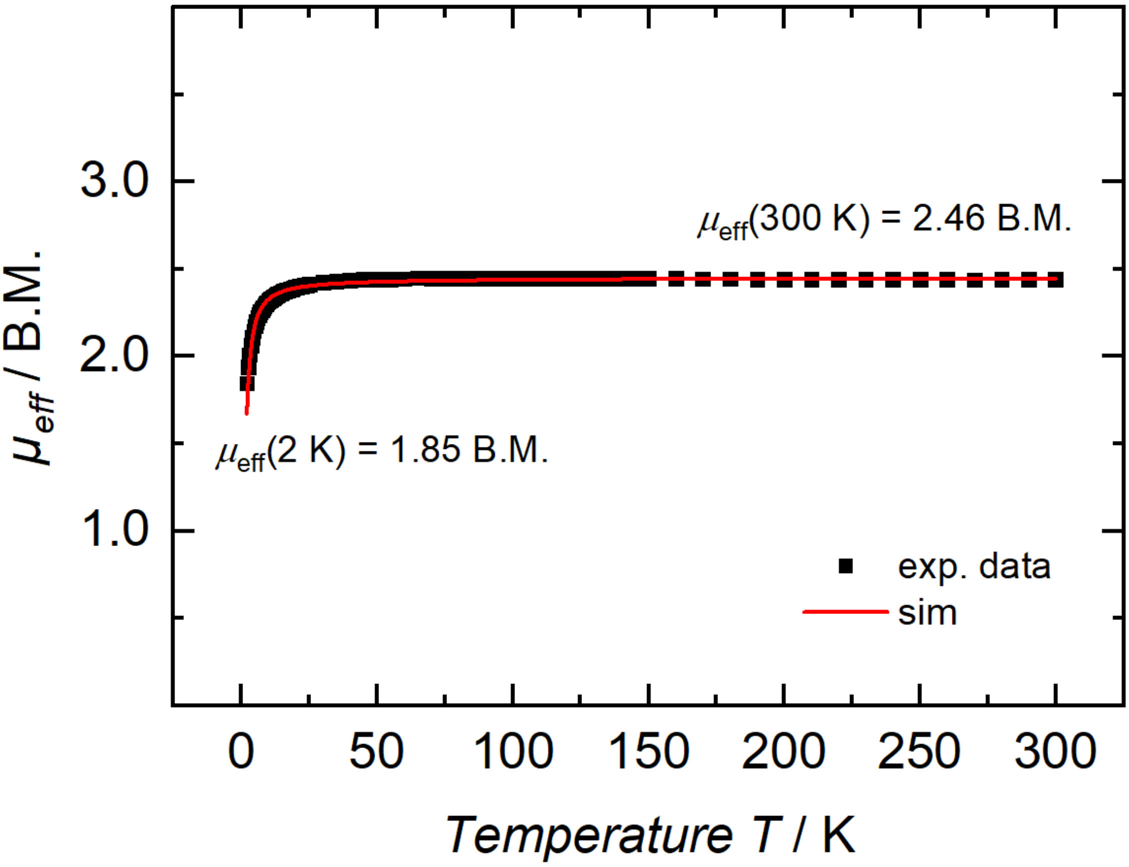

Figure S15. Temperature-dependent SQUID magnetization data of polycrystalline **4** (black squares) and its simulation (red trace) with an applied magnetic field of 1 T (2–300 K), plotted as μ_{eff} *vs*. T. Simulation parameters: $S_1 = S_2 = 1/2$, $J = -1.23$ cm^{-1}, $TIP = 1184 \cdot 10^{-6}$ emu, $|D_1| = |D_2| = 0.00$ cm^{-1}, $E/D_1 = E/D_2 = 0.00$, $g_{avg,1} = g_{avg,2} = 2.00$.

9 Electrochemical Studies

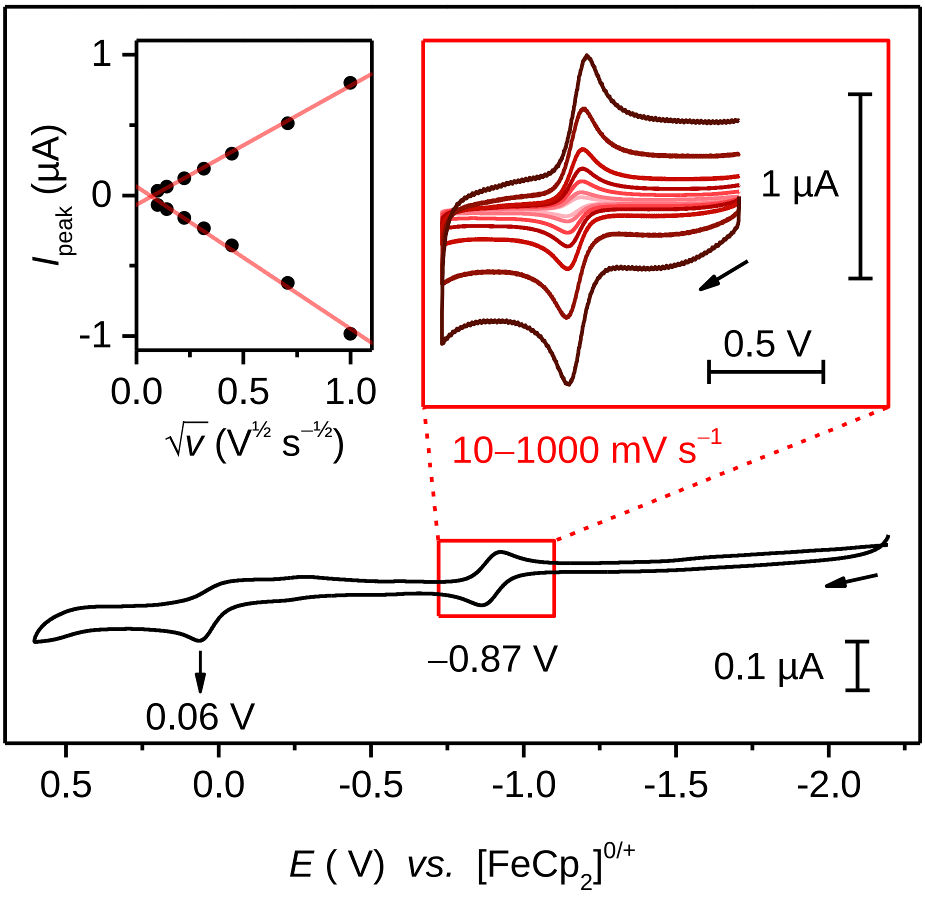

Figure S16. CV data on 1.4 · 10^{-4} M [(Tp^{tBu,Me})Ti{1,3-μ_2-NCNAd}$_2$Ti(Tp^{tBu,Me})][B(C_6F_5)$_4$]$_2$ (**4**) in THF containing 0.1 M [^{n}Bu$_4$N][PF_6] as electrolyte; referenced to [FeCp$_2$]$^{0/+}$ at *E* = 0. Inset: Dependence on scan rate of the reversible feature at –0.87 V [recorded at 10, 20, 50, 100, 200, 500, 1000 mV s^{-1}] along with a plot of cathodic/anodic peak currents *versus* square root of scan rate.

10 Crystallographic Data

Table S1. Crystallographic data for complexes **3** and **4**.

**Complex**<br>**CCDC entry**	$[(Tp^{tBu,Me})Ti\{AdN(N)C–C(N)NAd\}Ti(Tp^{tBu,Me})]$<br>**2445536 (3)**	$[(Tp^{tBu,Me})Ti\{1,3\text{-}\mu_2\text{-}NCNAd\}_2Ti(Tp^{tBu,Me})][B(C_6F_5)_4]_2$<br>**2445537 (4)**
Empirical formula	$C_{78}H_{130}B_2N_{16}O_2Ti_2$	$C_{134}H_{150}B_4F_{40}N_{16}O_4Ti_2$
Formula weight	1441.39	2947.73
Temperature / K	100(2)	100(2)
Crystal system	triclinic	triclinic
Spacegroup	*P*–1	*P*–1
a / Å	11.1596(5)	14.0910(2)
b / Å	11.4928(5)	14.8091(2)
c / Å	15.7244(7)	16.4769(2)
α / °	90.689(4)	99.9250(10)
β / °	97.872(4)	92.3010(10)
γ / °	90.376(4)	94.2010(10)
V / Å^3	1997.52(15)	3372.88(8)
Z	1	1
ρ_{calc} g/cm^3	1.198	1.451
μ / mm^{-1}	0.255	2.017
F(000)	780.0	1522.0
Crystal size / mm^3	0.12 × 0.1 × 0.01	0.42 × 0.08 × 0.05
Radiation (Å)	Mo K_α (λ = 0.71073)	Cu K_α (λ = 1.54184)
2θ range / °	4.378 – 56.564	5.452 – 148.972
Index ranges	$-14 \le h \le 14, -15 \le k \le 14, -20 \le l \le 20$	$-15 \le h \le 16, -18 \le k \le 17, -20 \le l \le 19$
Reflections collected	43229	72165
Independent reflections	9874 [R_{int} = 0.0971, R_{sigma} = 0.1009]	13264 [R_{int} = 0.0530, R_{sigma} = 0.0320]
Data / restraints / parameters	9874 / 300 / 546	13264 / 231 / 1013
Goodness-of-fit on F^2	1.119	1.043
Final *R* indexes [$I \ge 2\sigma(I)$]	R_1 = 0.0849, wR_2 = 0.1883	R_1 = 0.0614, wR_2 = 0.1714
Final *R* indexes [all data]	R_1 = 0.1392, wR_2 = 0.2065	R_1 = 0.0727, wR_2 = 0.1809
Largest diff. peak/hole / e Å^{-3}	0.80 / –0.56	0.84 / –0.83

Table S2. Crystallographic data for complex **6**.

**Complex**<br>**CCDC entry**	$[(Tp^{tBu,Me})V(\eta^1\text{-}NCNAd)(CNAd)_2]$<br>**2445538 (6)**
Empirical formula	$C_{57}H_{85}BN_{10}V$
Formula weight	972.09
Temperature / K	100(2)
Crystal system	triclinic
Spacegroup	*P*–1
a / Å	11.9108(2)
b / Å	12.6051(2)
c / Å	24.0379(5)
α / °	104.5687(18)
β / °	90.2187(18)
γ / °	118.154(2)
V / Å^3	3047.78(12)
Z	2
ρ_{calc} g/cm^3	1.059
μ / mm^{-1}	0.204
F(000)	1050.0
Crystal size / mm^3	0.33 × 0.23 × 0.03
Radiation (Å)	Mo K_α (λ = 0.71073)
2θ range / °	3.538 – 56.564
Index ranges	$-15 \le h \le 15, -16 \le k \le 16, -32 \le l \le 32$
Reflections collected	89248
Independent reflections	15113 [R_{int} = 0.0545, R_{sigma} = 0.0357]
Data / restraints / parameters	15113 / 374 / 733
Goodness-of-fit on F^2	1.099
Final *R* indexes [$I \ge 2\sigma(I)$]	R_1 = 0.0895, wR_2 = 0.2370
Final *R* indexes [all data]	R_1 = 0.0996, wR_2 = 0.2421
Largest diff. peak/hole / e Å^{-3}	1.24 / –0.70

11 Computational Studies

11.1 Computational Methodology

We performed density functional theory (DFT) calculations as implemented in the Orca software package v. 4.2.1.[13] Gas-phase geometry optimizations were carried out using the PBE0 functional[14] in combination with the def2-SV(p) basis[15] and the auxiliary basis set def2-J.[16] Electronic structure searches were accelerated via the resolution of the identity approximation for Coulomb and chain of spheres approximation for exchange interactions (RIJCOSX).[17] Grimme's D3 method and the Becke-Johnson (D3BJ) damping scheme accounted for the dispersion effects in all calculations.[18] A tight convergence of the wavefunction was requested on grid quality Grid4.

Analytical frequency calculations at the same level of theory as optimizations (PBE0-D3/def2-SV(p)/Grid4) were carried out to confirm that the resulting equilibrium structures corresponded to the minima of the potential energy surface. Thermodynamic corrections to the electronic energy were obtained within the ideal gas–rigid rotor–harmonic oscillator approximation at T=298.15 K. We performed subsequent single-point calculations to refine the electronic energies and wavefunctions of the calculated species. For these calculations, the meta GGA hybrid TPSSh functional[19–21] in combination with the def2-TZVP basis set was employed. Quasirestricted orbitals (QROs) for open-shell species and unrestricted Hartree-Fock (UHF) corresponding orbitals for broken symmetry species were generated via single-point calculations at the TPSSh-D3/Def2-TZVP level of theory.

The SMD implicit solvation model[22] was employed for the calculation of solvation energies of the species studied at the TPSSh-D3/Def2-TZVP level of theory. The GEPOL algorithm was used to generate the solvent cavity as a solvent-excluded surface (GEPOL-SES).[23–25] The atomic radii of the default method were adjusted to H (1.150 Å), C (1.900 Å), N (1.600 Å), B (2.042), P (2.074 Å), and Ti (2.999 Å). The radius of the solvent toluene was set at 2.76 Å. The gas phase Gibbs free energy values were corrected by solvation energies to obtain solution-state Gibbs free energies of the individual species.

11.2 Computational Data

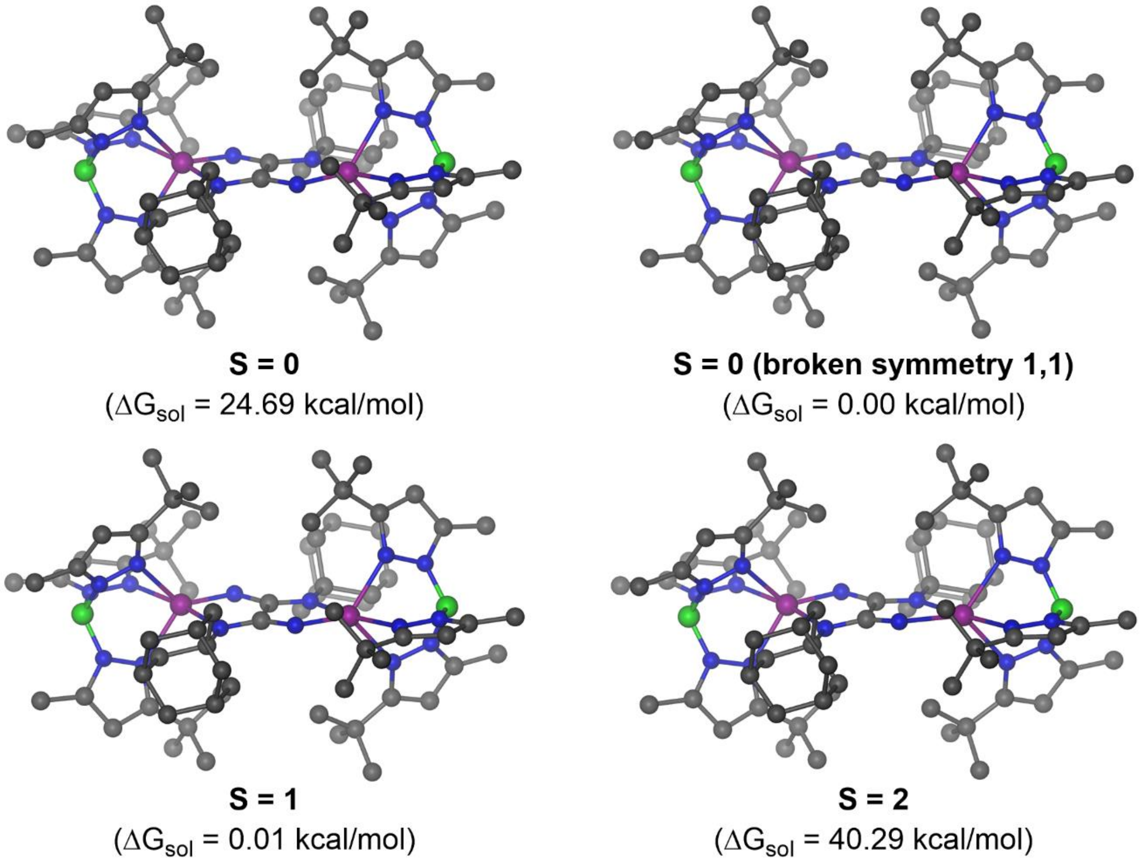

Figure S17. Calculated equilibrium structures (PBE0-D3/def2-SV(p)) of [(Tp^{tBu,Me})Ti{AdN(N)C–C(N)NAd}Ti(Tp^{tBu,Me})] (**3**) considering different spin states (S is the spin multiplicity, M=2S+1). Relative solution state (toluene) Gibbs free energies of these species calculated at the TPSSh-D3/def2-TZVP level of theory are provided in parenthesis.

Table S3. Calculated distances (Å) of [(Tp^{tBu,Me})Ti{AdN(N)C–C(N)NAd}Ti(Tp^{tBu,Me})] (**3**) at the PBE0-D3/def2-SV(p) level of theory. The unsigned difference between calculated and experimental structural XRD data is provided in parenthesis. Numbering is provided using the singlet (S = 0) equilibrium structure as a reference. Hydrogen atoms are omitted for clarity.

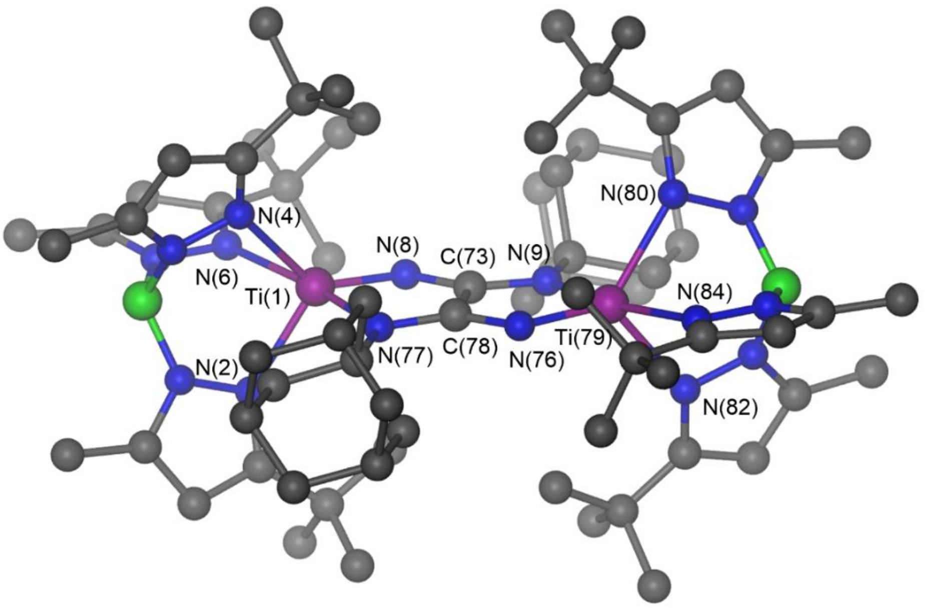

Bond	Multiplicity			
	Singlet	Brok. Symm. Singlet (1,1)	Triplet	Quintet
Ti(1)-N(2)	2.201 (0.036)	2.233 (0.004)	2.233 (0.004)	2.222 (0.015)
Ti(1)-N(4)	2.259 (0.031)	2.248 (0.020)	2.248 (0.020)	2.233 (0.005)
Ti(1)-N(6)	2.284 (0.023)	2.290 (0.017)	2.289 (0.018)	2.287 (0.020)
Ti(1)-N(8)	1.746 (0.092)	1.825 (0.013)	1.824 (0.014)	1.837 (0.000)
Ti(1)-N(77)	2.063 (0.182)	2.242 (0.003)	2.241 (0.004)	2.215 (0.030)
N(8)-C(73)	1.360 (0.030)	1.317 (0.013)	1.318 (0.012)	1.309 (0.022)
N(9)-C(73)	1.389 (0.059)	1.327 (0.003)	1.327 (0.002)	1.319 (0.011)
C(73)-C(78)	1.406 (0.144)	1.534 (0.016)	1.533 (0.017)	1.577 (0.027)
N(77)-C(78)	1.387 (0.057)	1.327 (0.003)	1.327 (0.003)	1.331 (0.001)
N(76)-C(78)	1.362 (0.032)	1.318 (0.012)	1.318 (0.012)	1.285 (0.045)
Ti(79)-N(76)	1.743 (0.095)	1.824 (0.014)	1.824 (0.014)	2.190 (0.352)
Ti(79)-N(9)	2.061 (0.184)	2.243 (0.002)	2.242 (0.003)	2.242 (0.004)
Ti(79)-N(80)	2.242 (0.005)	2.238 (0.001)	2.239 (0.002)	2.239 (0.002)
Ti(79)-N(82)	2.208 (0.020)	2.237 (0.009)	2.237 (0.009)	2.224 (0.004)
Ti(79)-N(84)	2.288 (0.019)	2.288 (0.019)	2.287 (0.020)	2.329 (0.022)

Table S4. Calculated Mayer bond orders for [(Tp^{tBu,Me})Ti{AdN(N)C–C(N)NAd}Ti(Tp^{tBu,Me})] (**3**) at the TPSSh-D3/def2-TZVP level of theory. Data is provided for the most stable triplet (S = 1) and broken symmetry singlet species (S = 0).

**Bond**	**Triplet**	**Brok. Symm. Singlet (1,1)**
Ti(1) – N (77)	0.4507	0.4483
Ti(1) – N (8)	1.3344	1.3278
Ti(79) – N (76)	1.3322	1.3260
Ti(79) – N (9)	0.4513	0.4489
N(77) – C(78)	1.4212	1.3725
N(8) – C (73)	1.3929	1.3968
N(76) – C(78)	1.4005	1.4044
N(9) – C(73)	1.4212	1.4204
C(73) – C(78)	0.9056	0.9033

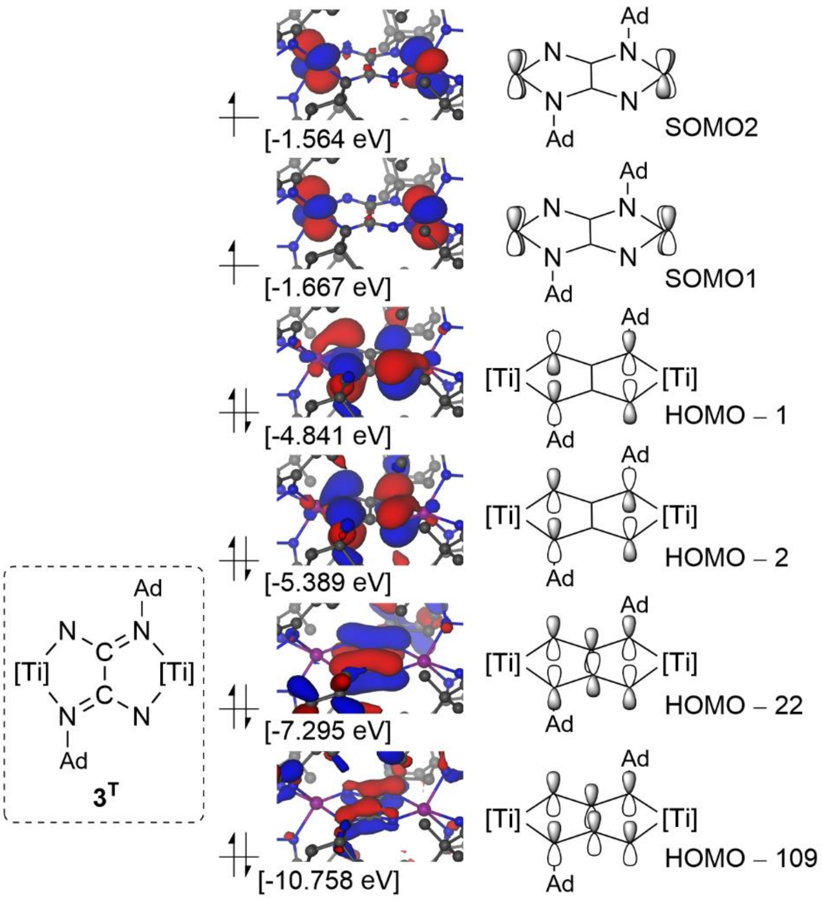

Figure S18. Most relevant quasi-restricted molecular orbitals (QROs) for **3**$^{\mathbf{T}}$ plotted at an isovalue of ± 0.03 a.u.

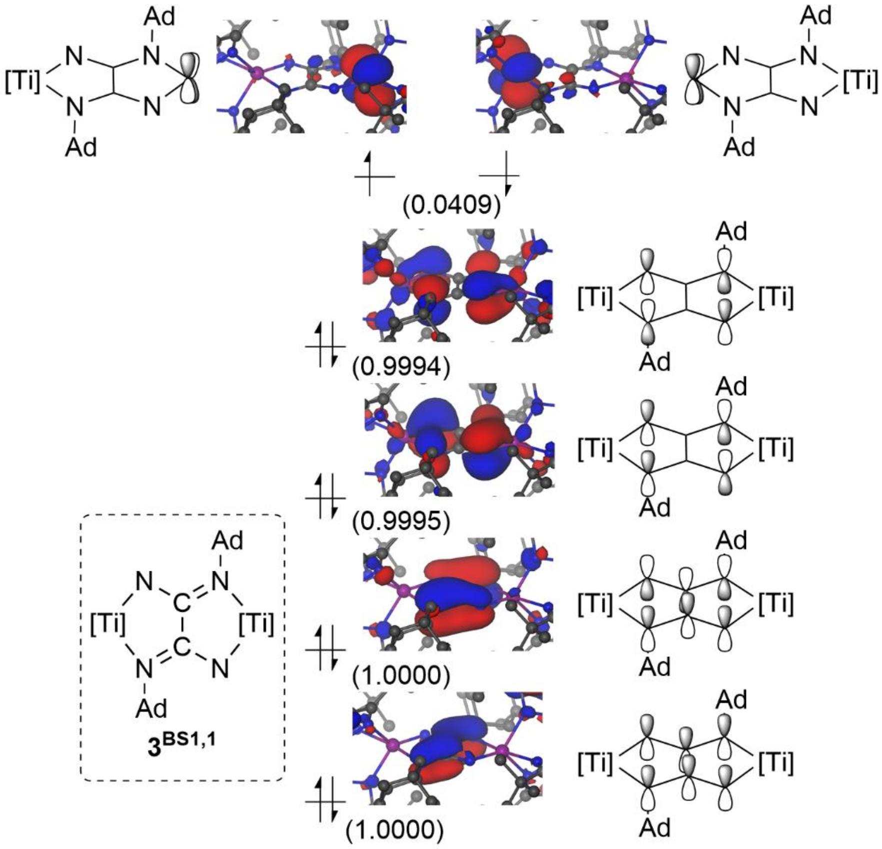

Figure S19. Most relevant unrestricted Hartree-Fock (UHF) corresponding orbitals for $\mathbf{3}^{\mathbf{BS(1,1)}}$ plotted at an isovalue of ± 0.03 a.u. The overlap between the corresponding orbitals is provided in parenthesis.

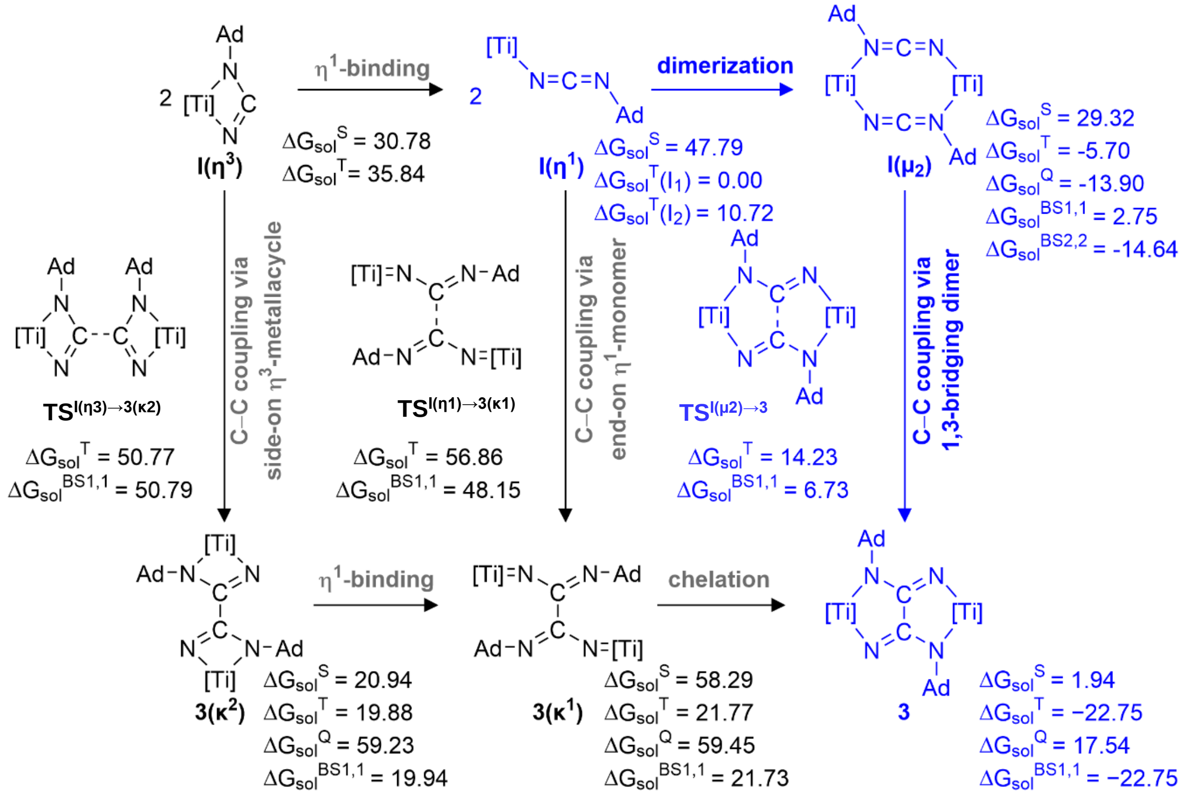

Figure S20. Calculated thermodynamics (kcal/mol) of the proposed mechanistic pathways for the formation of complex **3**. Relative solution state (toluene) Gibbs free energies (ΔG_{sol}) are computed at the TPSSh-D3/def2-TZVP level and referenced to the most stable **I(η¹)** species. The most likely pathway to C−C bond formation is highlighted in blue. Superscripts represent the multiplicity of the species: S = singlet, T = triplet, Q = quintet, and BS = broken symmetry singlet.

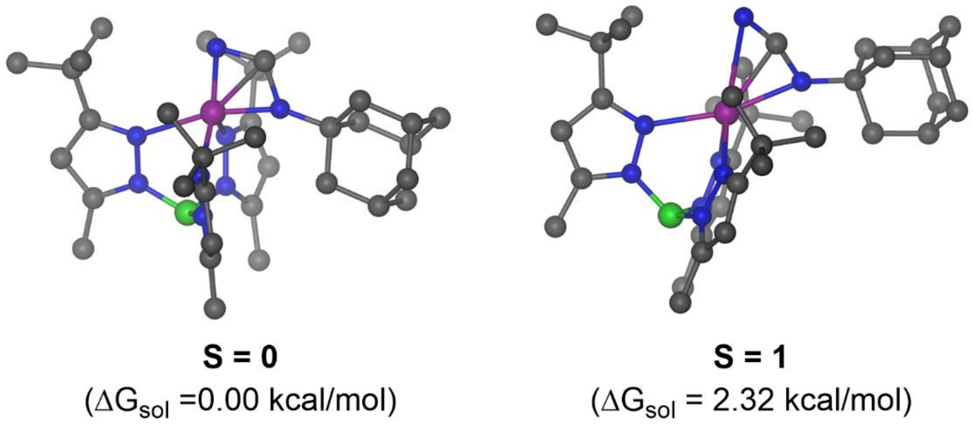

Figure S21. Calculated equilibrium structures (PBE0-D3/def2-SV(p)) of [(Tp^{tBu,Me})Ti(η^3-NCNAd)] (**I(η³)**) considering different spin states (S is the spin multiplicity, M=2S+1). Relative solution state (toluene) Gibbs free energies of these species calculated at the TPSSh-D3/def2-TZVP level of theory are provided in parenthesis.

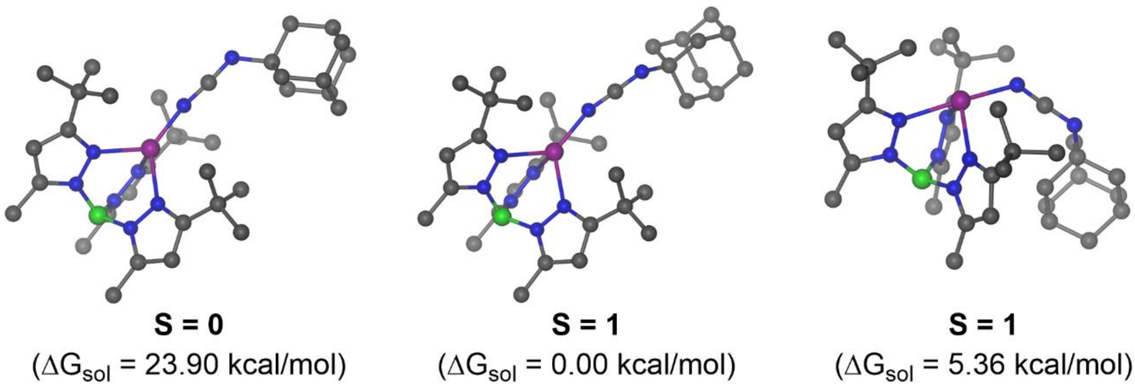

Figure S22. Calculated equilibrium structures (PBE0-D3/def2-SV(p)) of [(Tp^{tBu,Me})Ti(η^1-NCNAd)] (**I(η^1)**) considering different spin states (S is the spin multiplicity, M=2S+1). Relative solution state (toluene) Gibbs free energies of these species calculated at the TPSSh-D3/def2-TZVP level of theory are provided in parenthesis.

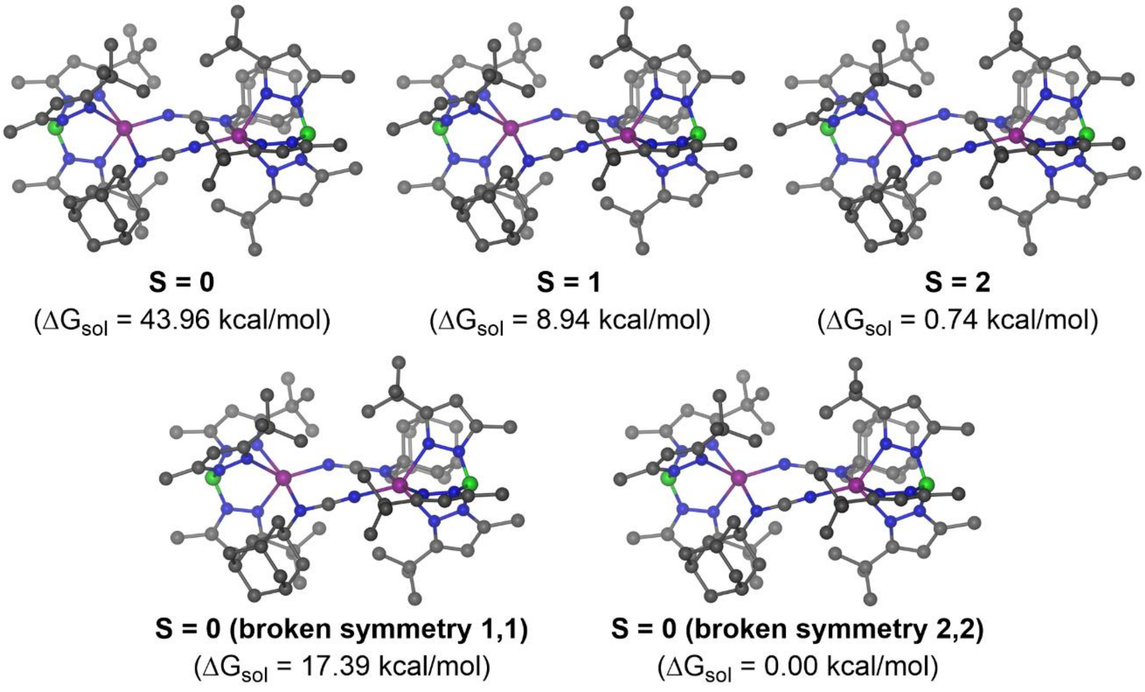

Figure S23. Calculated equilibrium structures (PBE0-D3/def2-SV(p)) of [(Tp^{tBu,Me})Ti(1,3-μ_2-NCNAd)$_2$Ti(Tp^{tBu,Me})] (**I(μ$_2$)**) considering different spin states (S is the spin multiplicity, M=2S+1). Relative solution state (toluene) Gibbs free energies of these species calculated at the TPSSh-D3/def2-TZVP level of theory are provided in parenthesis.

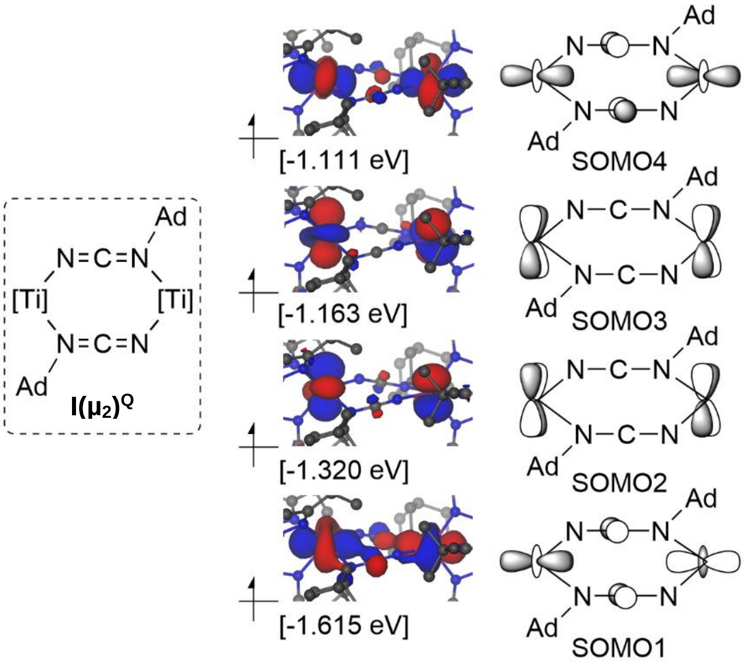

Figure S24. Most relevant quasi-restricted molecular orbitals (QROs) for **I(μ2)^Q^** plotted at an isovalue of ± 0.03 a.u.

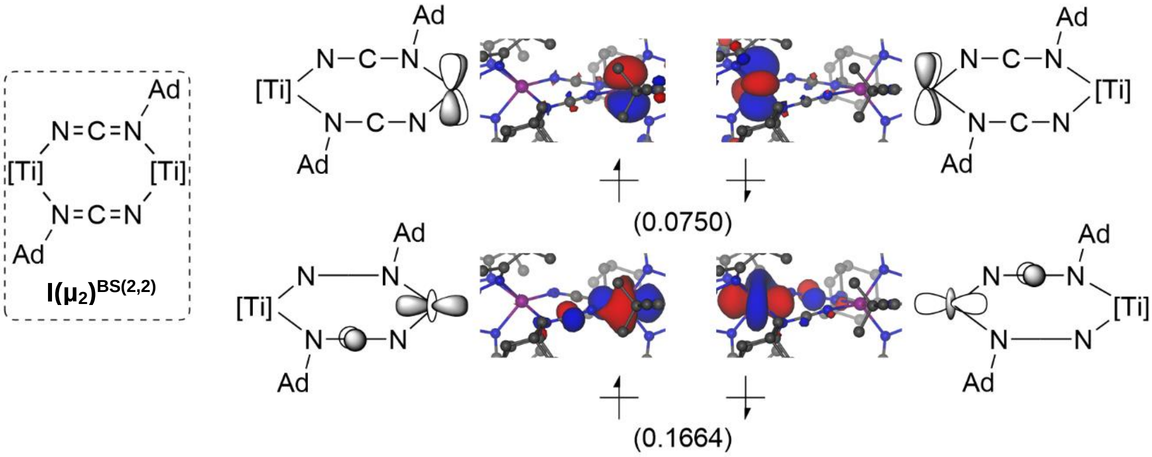

Figure S25. Most relevant unrestricted Hartree-Fock (UHF) corresponding orbitals for **I(μ2)^BS(2,2)^** plotted at an isovalue of ± 0.03 a.u. The overlap between the corresponding orbitals is provided in parenthesis.

Table S5. Calculated Mayer bond orders for [(Tp^{tBu,Me})Ti(1,3-μ$_2$-NCNAd)$_2$Ti(Tp^{tBu,Me})] (**I(μ$_2$)**) at the TPSSh-D3/def2-TZVP level of theory. Data is provided for the most stable quintet (S = 2) and broken symmetry singlet species (S = 0).

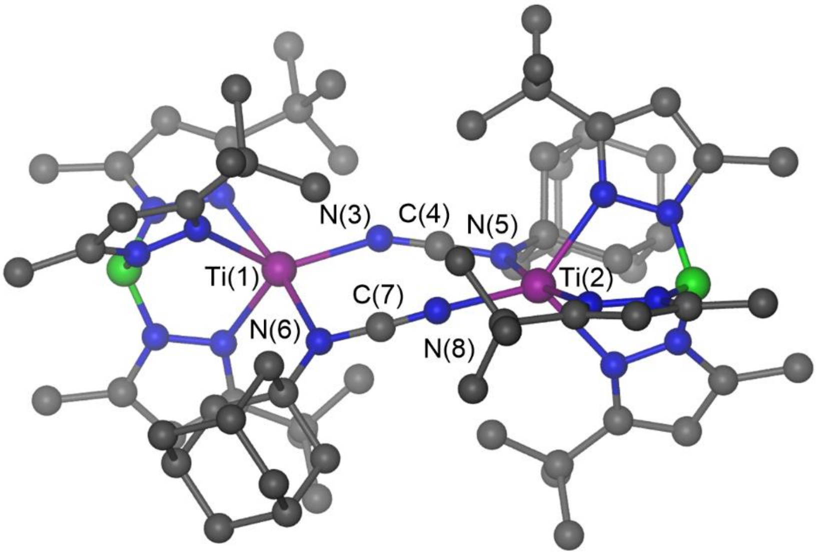

Bond	Quintet	Brok. Symm. Singlet (2,2)
Ti(1) – N (3)	0.4152	0.4219
Ti(1) – N (6)	0.3810	0.3913
Ti(2) – N (5)	0.3787	0.3902
Ti(2) – N (8)	0.4087	0.4166
N(3) – C(4)	1.4279	1.4258
N(6) – C (7)	2.2748	2.2693
N(5) – C(4)	2.2822	2.2763
N(8) – C(7)	1.4265	1.4272
C(4) – C(7)	< 0.1	< 0.1

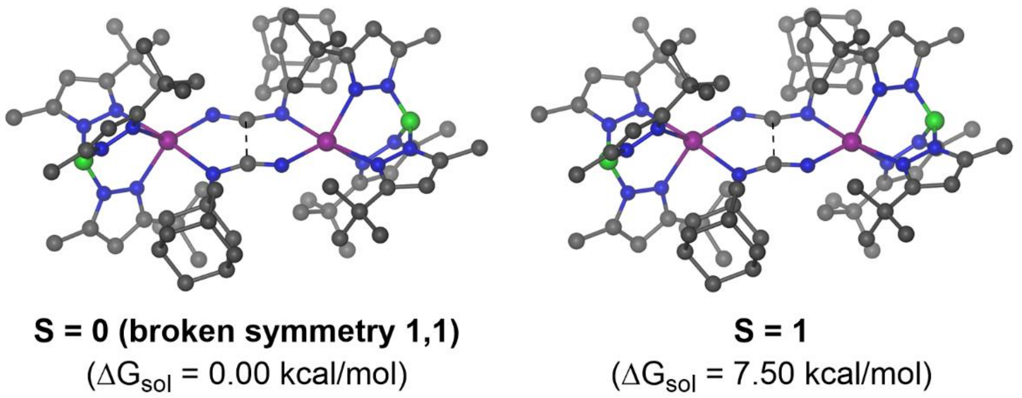

Figure S26. Calculated equilibrium structures (PBE0-D3/def2-SV(p)) of $\mathbf{TS^{I(\mu2)\rightarrow3}}$ considering different spin states (S is the spin multiplicity, M=2S+1). Relative solution state (toluene) Gibbs free energies of these species calculated at the TPSSh-D3/def2-TZVP level of theory are provided in parenthesis.

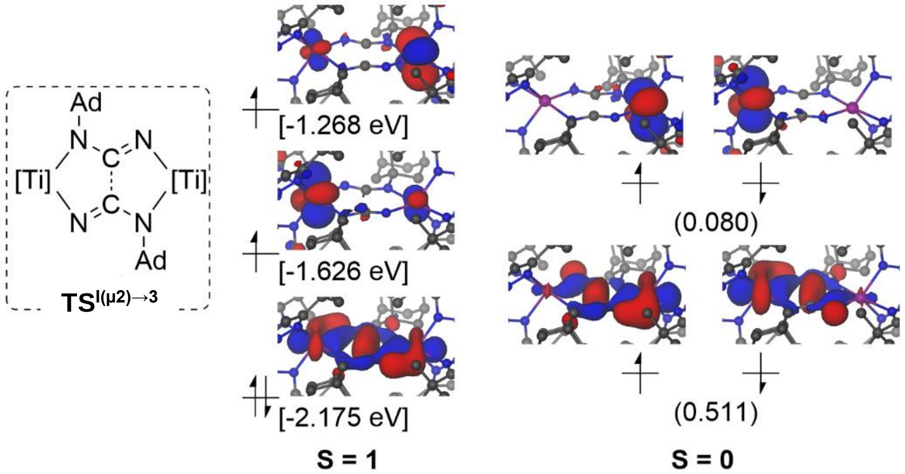

Figure S27. Most relevant Quasirestricted orbitals (QROs, left) and unrestricted Hartree-Fock corresponding orbitals (UHF, right) for $\mathbf{TS^{I(\mu2)\rightarrow3}}$ plotted at an isovalue of ± 0.03 a.u. The overlap between the corresponding UHF orbitals is provided in parenthesis.

Table S6. Calculated Mayer bond orders for $\mathbf{TS}^{\mathbf{I(\mu 2)\rightarrow 3}}$ at the TPSSh-D3/def2-TZVP level of theory. Data is provided for the triplet (S = 1) and broken symmetry singlet species (S = 0).

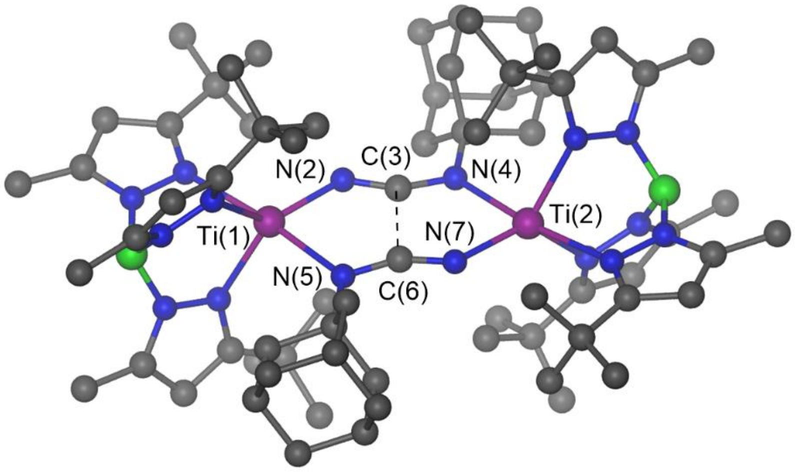

**Bond**	**Triplet**	**Brok. Symm. Singlet (1,1)**
Ti(1) – N (5)	0.4181	0.4482
Ti(1) – N (2)	0.6560	0.7695
Ti(2) – N (7)	0.9060	0.7942
Ti(2) – N (4)	0.4587	0.4483
N(5) – C(6)	1.4089	1.3638
N(2) – C (3)	2.0032	1.9192
N(7) – C(6)	1.8651	1.9284
N(4) – C(3)	1.4011	1.4110
C(3) – C(6)	0.2892	0.3213

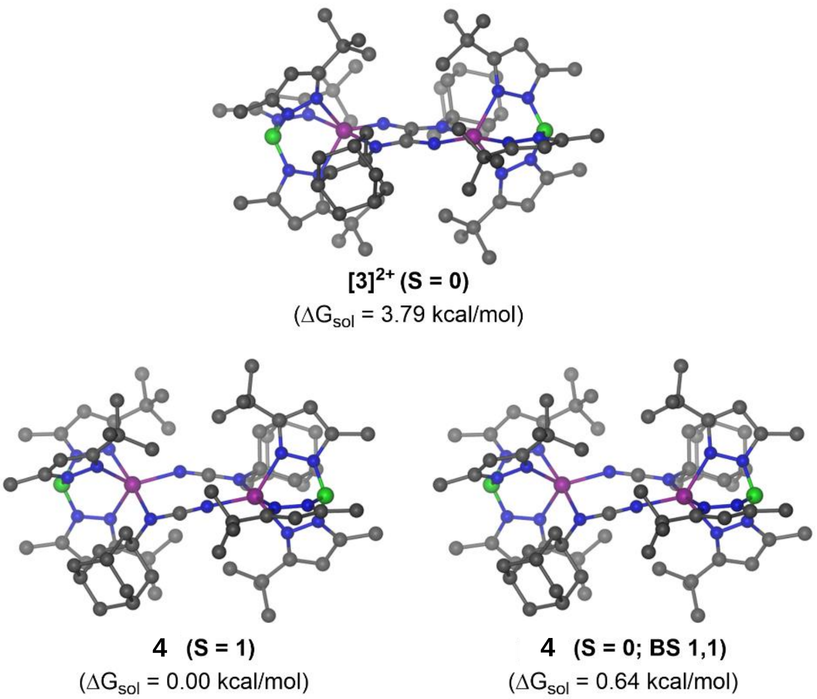

Figure S28. Calculated equilibrium structures (PBE0-D3/def2-SV(p)) of dications $[(Tp^{tBu,Me})Ti\{AdN(N)C–C(N)NAd\}Ti(Tp^{tBu,Me})]^{2+}$ (**[3]**$^{2+}$) and $[(Tp^{tBu,Me})Ti\{1,3\text{-}\mu_2\text{-}NCNAd\}_2Ti(Tp^{tBu,Me})]^{2+}$ (**4**) considering different spin states (S is the spin multiplicity, M=2S+1). Relative solution state (toluene) Gibbs free energies of these species calculated at the TPSSh-D3/def2-TZVP level of theory are provided in parenthesis.

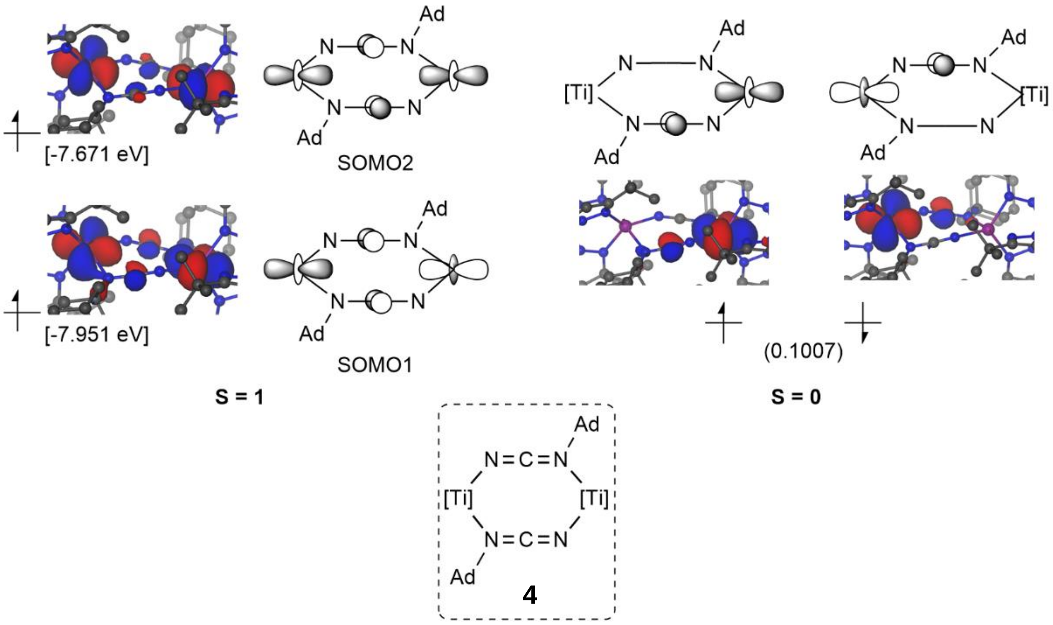

Figure S29. Most relevant Quasirestricted orbitals (QROs, left) and Unrestricted Hartree Fock (UHF) corresponding orbitals (right) for **4** plotted at an isovalue of ± 0.03 a.u. The overlap between the corresponding UHF orbitals is provided in parenthesis.

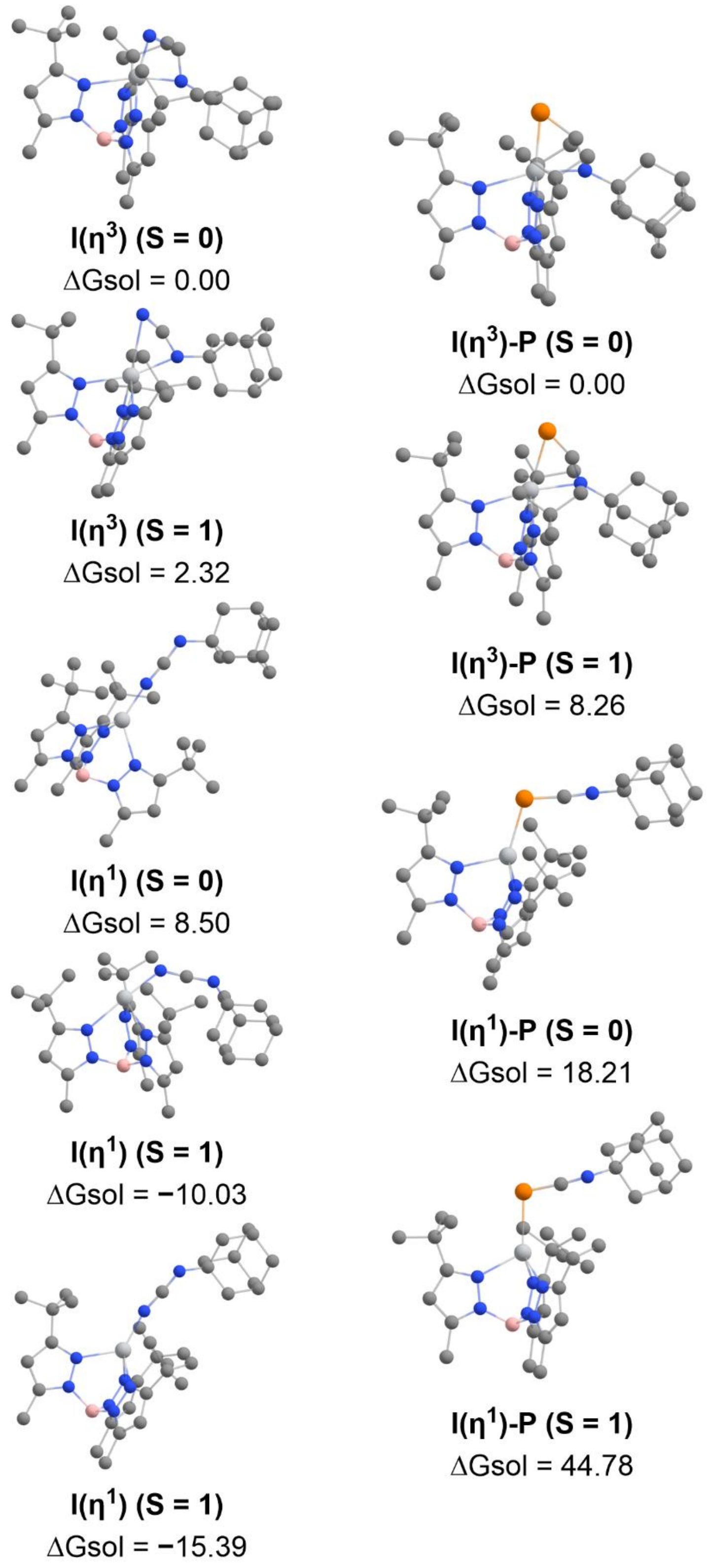

Figure S30. Solution state relative energies for [(Tp^{tBu,Me})Ti(η^3-NCNAd)] (**I(η^3)**) and [(Tp^{tBu,Me})Ti(η^1-NCNAd)] (**I(η^1)**) complexes and their (Tp^{tBu,Me})Ti(PCNAd)] analogs. Energies are calculated at the TPSSh-D3/def2-TZVP level of theory.

Cartesian coordinates of optimized structures (in Å) at PBE0-D3/Def2-SV(p)/Grid4 level of theory. S is the spin multiplicity (M=2S+1).

[(Tp^{tBu,Me})Ti{AdN(N)C–C(N)NAd}Ti(Tp^{tBu,Me})] **(3) (S = 0; broken symmetry 1,1)**

```
Eh = -5351.489954148951
Ti    4.23297640576319    5.81935660681442    5.04105426310264
N     5.21547390618339    4.27764361436699    3.75927460180287
N     4.38063620504134    3.94102660696295    2.74773547162826
N     2.14684421683738    5.87711016173188    4.20655197070494
N     2.08705818801936    5.10156591309446    3.09878841954656
N     4.51126765035873    6.96947552173766    3.08037392866498
N     3.98710058759036    6.30377601594116    2.02725003342748
N     4.95435696804012    6.72034935499713    6.45437375670433
N     5.47463358483923    6.68017375109630    8.81517852946360
C     4.75600169236433    2.76365129401052    2.20004148334212
C     5.88970918798551    2.33936042043039    2.87541365896228
H     6.46052008741092    1.43443046014573    2.67679682740509
C     6.15555239296226    3.32928523597916    3.83884061450122
C     4.04484762655599    2.10782500614809    1.06808387792079
H     2.97474216975752    1.95964686830445    1.28865599023831
H     4.49377163645823    1.12102672002068    0.87597494028392
H     4.11252019819831    2.70217766799761    0.14066457013937
C     7.36060661096781    3.38968132692759    4.74707286689155
C     7.48472129462003    2.07587309123892    5.52809183537415
H     6.59481612768981    1.90936204494011    6.15756450727210
H     8.37174218003729    2.10792216603750    6.18582448765146
H     7.59564399325651    1.20933414576483    4.85341191851444
C     7.25923588690671    4.55765421108515    5.71554100773907
H     7.18569965981312    5.51850938358710    5.18011143025913
H     8.15225871769804    4.60120024886462    6.36021332729260
H     6.37762375174759    4.45635388836083    6.36541786299295
C     8.60424539946886    3.57512271485273    3.86279545483793
H     8.72077041599945    2.74162984644668    3.14860916819372
H     9.51263878983689    3.62116756646801    4.49060416710290
H     8.53613642004161    4.51296578736967    3.28442372371107
C     0.80211098148311    4.79833553161521    2.80697186407340
C     0.00624961092850    5.42479619953585    3.75466965940445
H    -1.08095606526373    5.40606663366779    3.79979105375707
C     0.89226735271374    6.11344170074943    4.60351959081523
C     0.39156455232723    3.94418471263301    1.65844642804578
H     0.65403370379009    4.40214223474632    0.68927550977542
H    -0.69851736096658    3.79312948327911    1.68274857648492
H     0.87347656124864    2.95275584738498    1.69696698140397
C     0.53502310108316    7.12467442641434    5.66654492046623
C    -0.51361486798344    6.56294546589717    6.63230076810235
H    -1.41112710636693    6.20911685056987    6.09570568641772
H    -0.83338411950782    7.34979336879487    7.33814382855189
H    -0.11108872097138    5.72248416286823    7.22030064957259
```

C	-0.05644236317178	8.34813995319548	4.94509942188467
H	0.66988279016919	8.76952186511211	4.22877310416337
H	-0.31446609481682	9.13201581508275	5.68012649014189
H	-0.97137690772595	8.08247237309254	4.38775549212891
C	1.77942882061523	7.55215706387751	6.42960004883964
H	2.24784640334031	6.69415073434574	6.93537699669062
H	1.52588022824110	8.30238931798629	7.19523032215196
H	2.52450519115397	8.00628024411911	5.75622111908728
C	4.11121306884403	7.02393577481874	0.89164309572851
C	4.74955988303725	8.20672324991853	1.23007434275759
H	5.01089022692631	9.01555844354698	0.55308762495683
C	4.98608412188334	8.13169629624112	2.61576131871454
C	3.62441986707933	6.56591113688020	-0.43971633383978
H	4.10091944886051	5.61938728584347	-0.74800133213745
H	3.85727495512024	7.32860954401095	-1.19905676457977
H	2.53286602867673	6.40173939325997	-0.44270354033105
C	5.69277746764423	9.15205872465915	3.47836604936269
C	6.08348043955391	10.36294682401245	2.62786927665827
H	6.60773857229429	11.10400966204938	3.25576325214275
H	5.19562704927365	10.85566546996670	2.19422525745961
H	6.76080963820727	10.07959245104233	1.80366343141565
C	6.96493852395685	8.51229041793342	4.04865705570887
H	7.52954082100989	9.24055753461955	4.65682899549084
H	7.62068980735244	8.15827877720321	3.23372162575516
H	6.70913964148482	7.65665017107674	4.69267491139586
C	4.76622923126868	9.61723655798803	4.60911642764812
H	4.57960552517360	8.80007652045296	5.32435823286976
H	3.80172402025627	9.97122146228077	4.20392893339281
H	5.22943766405571	10.45191098924146	5.16441963474561
C	4.93007636527119	6.24882581822757	7.68426235770262
B	3.33078610561167	4.92911204669449	2.19611433977379
H	2.95836053023631	4.51608926757818	1.12168243387902
N	3.97810583041280	4.55609085420871	9.12504142209453
N	3.75287631339189	4.41798675294450	6.72377609420157
C	4.14114011565552	4.94689648817175	7.87702400331738
Ti	4.74994059724269	5.42792645236705	10.52951700050446
N	3.96209883783085	7.16439633345793	11.70097276112521
N	4.85149531675024	7.45443140833866	12.67999320167029
N	6.76861723803241	5.09349915559342	11.43273774309026
N	6.90091776906622	5.86588545097083	12.53691914306146
N	4.20888850461327	4.46196346539506	12.53154414883756
N	4.75243018114162	5.12861418692660	13.57354874464451
C	4.66248586523851	8.71727398929004	13.12568126363777
C	3.59087134585442	9.24566719186129	12.42164797075715
H	3.15929491203647	10.23671498428306	12.54745699068142
C	3.16309564094303	8.22499682176215	11.55142615928759
C	5.49395453122584	9.35081852909103	14.18550202142129
H	6.56734475673477	9.31003391736874	13.93561476650781

H	5.20970091280386	10.40877389375285	14.29484985705477
H	5.36284346014562	8.85578868664569	15.16302169770355
C	1.90060857157029	8.21246724974778	10.72131795003343
C	1.83465059074205	9.44096458095538	9.80716658188604
H	2.61498718329170	9.40539818134962	9.03068235199983
H	0.85265661368388	9.48336712713076	9.30313724789490
H	1.96059973956353	10.37795049094079	10.37744989679159
C	1.81186670669315	6.93441285115208	9.90044159402544
H	1.81954896996495	6.04497451725421	10.55126817993985
H	0.87871178130368	6.91403420437495	9.31303077043044
H	2.65417510719006	6.85210841438475	9.19750100515481
C	0.71911291418418	8.25115290881906	11.70696736870748
H	0.72856861649932	9.17235621361057	12.31500067603977
H	-0.23707532282573	8.21180984962212	11.15425529165593
H	0.75658058432830	7.38742574992608	12.39355305641005
C	8.20042395717528	5.94800618486336	12.90029404357062
C	8.92711868769539	5.18069661870553	12.00398001217254
H	10.00337933307962	5.01918421030053	12.01429854056120
C	7.98660254653626	4.65105880804596	11.10211362859522
C	8.69283816976926	6.72986685879081	14.06791801414039
H	8.31493298961497	6.32608720173805	15.02316289190628
H	9.79303598042189	6.69750344035823	14.09327390583584
H	8.38361862229203	7.78685726912146	14.01063517589484
C	8.27749085562708	3.68930339974923	9.97530454917701
C	9.30536680602028	4.32016934248928	9.02705359385688
H	10.24243246913313	4.57204726087366	9.55310712236250
H	9.55156313210803	3.61853590819797	8.21023007672204
H	8.90549908400280	5.24523209651527	8.57990310203170
C	8.86438226326675	2.40390897316945	10.57807973225128
H	8.14650315791644	1.93243574900903	11.27149248497676
H	9.09584743158957	1.68063013352607	9.77536449699103
H	9.79540594124902	2.60544488991712	11.13557572789392
C	7.00909632601074	3.34980487651440	9.20766414502468
H	6.57253340830977	4.25072858838557	8.75103756349112
H	7.22768143115550	2.63455879444372	8.39815364328427
H	6.25154216932372	2.89286801964590	9.86500387249386
C	4.37150991778175	4.58136377479495	14.74785857749873
C	3.54771155681907	3.50798213875732	14.44549318560983
H	3.06715230427691	2.84183532737372	15.15710399139365
C	3.47365359977897	3.46580941352382	13.04027389347767
C	4.80205235564412	5.09448241519131	16.07853030277162
H	4.48974424979505	6.14175425078465	16.23372572570106
H	4.34922289477902	4.48176854119832	16.87362139363129
H	5.89870778886417	5.05702136789926	16.19875248861524
C	2.72460758486379	2.46449176639307	12.19167422376122
C	2.01301775458736	1.45230146233682	13.09312727218643
H	1.47636974546412	0.71479218931682	12.47130606536259
H	2.72750818269666	0.90096918076934	13.72901394943722

H	1.27264034370567	1.94323550674981	13.74877300969683
C	1.67777627529939	3.18925294520713	11.33578931847803
H	1.04762303429850	2.45856905718469	10.79904926964599
H	1.01835309499676	3.80920412006929	11.96885502810093
H	2.16432164429906	3.83133268718937	10.58406292720124
C	3.73076234051326	1.72124515695267	11.30325854889093
H	4.21866749003022	2.41937844306850	10.60517065747210
H	4.50760788233274	1.23398655564585	11.91849658806115
H	3.22392379684779	0.94312872882325	10.70592163672997
B	5.67651709868388	6.33096172525259	13.35334912565029
H	6.07336843494474	6.74849570202681	14.41724480829196
C	6.29808697601608	7.89227259863323	8.81876489101494
C	5.42241207221952	9.14110020464227	8.61997322354213
C	7.39688417505680	7.87755023621271	7.74273288172050
C	7.00002312313579	8.01922067674560	10.17591427697846
H	4.66377629302362	9.15745082190105	9.42081246801426
C	6.26714085086108	10.41709778008995	8.68219247932117
H	4.89439536328983	9.06386719006155	7.65413747351395
C	8.24445398671737	9.15170600642824	7.81322802427448
H	6.93007864584426	7.77865472069553	6.75336499025732
H	8.03209211775283	6.98608347166790	7.89516782032884
C	7.83668321739768	9.29551103077035	10.26400467901627
H	6.24633936647526	8.02074651027811	10.97700398108036
H	7.64950115521677	7.14177450891171	10.32844604470185
H	5.61336280535839	11.29366617239082	8.52074603628312
C	7.34729834918687	10.37454196206663	7.59792201981134
C	6.93382707835362	10.51449567709618	10.05768769158155
H	9.01340535547832	9.11515052864350	7.01998463092769
C	8.91866537845439	9.25787884824263	9.18316772022573
H	8.30885259621798	9.34537638881895	11.26286871626354
H	7.95250256147250	11.30028104853232	7.62730859468484
H	6.87663746972259	10.32448743036303	6.60122337998598
H	7.52550206566822	11.44662718718307	10.13241964609437
H	6.16205422253210	10.54920504651001	10.84980403518619
H	9.54725434111694	10.16704894687880	9.23346792055081
H	9.58424746918290	8.39022925669645	9.34900104760888
C	3.01019506024558	3.15613913873244	6.71387254292278
C	3.72492517691444	2.03367865032648	7.48601919600515
C	2.85313309755380	2.67704321370638	5.26659653253695
C	1.60398749814511	3.35211967959054	7.30442510597353
H	3.86687474597112	2.34622791616729	8.52990907661864
C	2.92002814736750	0.73159079918180	7.41731807250105
H	4.72850670021817	1.88546047124990	7.04717975501304
C	2.04075364825060	1.38481465265190	5.18201731520567
H	3.85247930093511	2.51172311455961	4.83298258852161
H	2.35360379731726	3.45921381578505	4.67629849082578
C	0.79901711124365	2.05106919679732	7.23957894791292
H	1.69513499383934	3.70604001006863	8.34496233427788

```
H     1.09641455970270    4.13938462462277    6.72089078368248
H     3.45047460201194   -0.04874301381579    7.99300336968156
C     2.76821112711403    0.28689318542565    5.96057643876040
C     1.53003078729284    0.95578782555429    8.02114959931335
H     1.94691800230029    1.09034826403920    4.12016460424082
C     0.65018576686404    1.61607827540693    5.77860425968069
H    -0.19948567340770    2.21973983592685    7.68226933144747
H     2.20529203856465   -0.66394701570167    5.90476626313549
H     3.76277309213959    0.10615590726796    5.51211697789707
H     0.94799590417575    0.01548781982767    7.99193290742903
H     1.62288810592869    1.24873074186212    9.08070744487479
H     0.04534389071268    0.69195598589286    5.71323599322003
H     0.12040466808617    2.39819719560516    5.20288830957854
```

[(Tp^{tBu,Me})Ti{AdN(N)C–C(N)NAd}Ti(Tp^{tBu,Me})] **(3) (S = 2)**

```
Eh = -5351.423579949724
Ti    4.19548855799540    5.78845489749262    4.96054561484742
N     5.22485792593993    4.25280027623672    3.72718850584180
N     4.42478964733810    3.89684973081065    2.69454091841817
N     2.13735693180319    5.80207800117747    4.09511177407474
N     2.11132009476943    5.02858619894072    2.98310959733961
N     4.50104209431887    6.93092097175999    3.00249136309725
N     4.01387743996535    6.24899782170462    1.94202176410961
N     4.83646837965331    6.70823071924599    6.41590661607296
N     5.42480909518329    6.68018196711729    8.75416277281318
C     4.82767990826922    2.71659324323119    2.17371787581500
C     5.94413291488852    2.31156115354761    2.88917020240992
H     6.52892681354050    1.40961129200592    2.71958951590818
C     6.17048951837735    3.31445018537349    3.84903542227097
C     4.15440652662820    2.03931569971138    1.03139082153599
H     3.08189581784785    1.87570855850623    1.22886302605647
H     4.62330756301634    1.05830864882327    0.85874592981888
H     4.23388990132033    2.62645827013256    0.10040246848444
C     7.34240218466294    3.39448402355033    4.79878510395519
C     7.43361503972744    2.09864047814526    5.61374371991811
H     6.52420354395322    1.95370057568257    6.22052678853205
H     8.30095635952260    2.13950926473944    6.29669403233780
H     7.55759030127070    1.21573718949124    4.96300858581363
C     7.20819720699007    4.58324723222940    5.73802403589621
H     7.15118757748144    5.53217495862848    5.17905864554553
H     8.07924281553103    4.64364441319979    6.41117046313489
H     6.30607831848010    4.49054419232006    6.36009742710972
C     8.61844277411881    3.55846376696924    3.95785521733465
H     8.76174872449720    2.70837135250947    3.26861709053345
H     9.50203806524230    3.61925736080618    4.61887204530749
H     8.57357251445121    4.48252033106179    3.35558713463782
C     0.83722842365069    4.71314799060438    2.66098919385327
C     0.01339299911079    5.32739964934143    3.59231378355697
H    -1.07379225878819    5.29340793253589    3.61352700612181
C     0.87087956325604    6.02228885762169    4.46490880261573
C     0.46124074879545    3.85845701581368    1.50115910624194
H     0.73931049562463    4.32281930722493    0.53947254703114
```

H	-0.62740562235371	3.69575488379061	1.50157182985638
H	0.95305473724545	2.87226186821310	1.54664870527915
C	0.46818680307477	7.02908635660247	5.51595399057736
C	-0.73794546797380	6.52325243123608	6.31323607173401
H	-1.60303602773046	6.31675928142778	5.65952234197495
H	-1.04859706435396	7.28593053978500	7.04864831622190
H	-0.49439579409709	5.59971333419654	6.86434893878289
C	0.08067493728056	8.31693741073400	4.76719706055095
H	0.93462996223163	8.69419398797913	4.17795382885506
H	-0.21652801670017	9.10006055605369	5.48790565359293
H	-0.76187674434967	8.14176054817118	4.07614188160606
C	1.62046690962114	7.34019924451539	6.45942721497480
H	1.94141643007747	6.44858738201205	7.01985668664673
H	1.31797421785792	8.11212866383089	7.18667252352751
H	2.49232583275009	7.73589977296002	5.91350846702575
C	4.14277804780634	6.96835482510922	0.80644963574443
C	4.74688085502469	8.16698382612273	1.15261789595734
H	5.00289730067136	8.97965286407591	0.47815172718492
C	4.95768232156337	8.10212128184810	2.54246443203546
C	3.68972056463930	6.49617178209674	-0.53168947428741
H	4.18727710286597	5.55620546855169	-0.82612171030774
H	3.92470110856502	7.25916582446297	-1.29001308597800
H	2.60106840776482	6.31489455142830	-0.55463338559678
C	5.62776775219541	9.13602450813477	3.41679585252284
C	6.01337244015487	10.35393157750123	2.57430885048163
H	6.51057805437378	11.10475623618553	3.21247044132358
H	5.12555594252977	10.82983233524240	2.12229505028001
H	6.71278397068984	10.08305911422484	1.76452134348505
C	6.90037108192658	8.51584425813630	4.00695267235031
H	7.44275716385347	9.24896018971528	4.62876103958546
H	7.57542096699361	8.17652652002177	3.20169336529789
H	6.65010185849720	7.65075519854225	4.64038286248023
C	4.66993342010907	9.58454285465313	4.52765311783254
H	4.46049304428617	8.75998744393215	5.22821813220459
H	3.71750104970860	9.94393767432974	4.09920437943462
H	5.11566841555204	10.41135113576910	5.10809642391916
C	4.84915753813756	6.25817871200469	7.64471863715976
B	3.37603810030118	4.86565180778457	2.10778634869750
H	3.03298025953919	4.43819436445260	1.02964816985978
N	3.80037812845361	4.57122869836935	9.00630504540327
N	3.71660076968521	4.38881141185418	6.60979863685992
C	4.03121243857012	4.91734041483701	7.79007470412320
Ti	4.89441195937733	5.56277696803405	10.62359395013356
N	4.10197077583909	7.22318026767878	11.89951948377312
N	4.99766275823105	7.47207206670781	12.88169288305366
N	6.86986338712731	5.16125284016876	11.56277224959141
N	7.02666562907225	5.86970253328138	12.69943639270104
N	4.30557136679585	4.48464429174454	12.60194120467260
N	4.85044407193454	5.10708281582687	13.67229588439121
C	4.83154146614112	8.72486657495210	13.36454468463914
C	3.76231028581443	9.28938663631791	12.68281103753613
H	3.34370688836426	10.28106228255216	12.84329793697740
C	3.31780639573280	8.30007028252447	11.78453472439729
C	5.67441064695459	9.30886363330655	14.44421556112446
H	6.74879387820957	9.23105795022131	14.20770171931887

H	5.42879241181745	10.37468366300538	14.57137481088504
H	5.51344795159267	8.80069792364713	15.41088228564210
C	2.04584391231980	8.30386114427506	10.96937141920679
C	1.83309960095706	9.65505706949143	10.28035596042600
H	2.64496498663055	9.87809157617774	9.56875902848903
H	0.88235376405241	9.64378434780787	9.71797479954757
H	1.77836492447253	10.48165646002202	11.01025599604660
C	2.06247442400911	7.18546450431219	9.93674740117136
H	2.19681021915515	6.20396628455347	10.41977352140412
H	1.10562419041496	7.15150722697287	9.38941850183414
H	2.87210786672092	7.32455193092625	9.20288047349283
C	0.88882858090304	8.05420223736195	11.95369790193786
H	0.84370991963027	8.84259703636598	12.72497990185091
H	-0.07589254122355	8.04000721607954	11.41415780256276
H	1.01657996513532	7.08349642661274	12.46379955991731
C	8.32852470194216	5.88908617029912	13.06745662210201
C	9.03089048248742	5.14636607673361	12.12968237152569
H	10.10100139726827	4.94671665811243	12.13138043586847
C	8.07032346022561	4.69611529584483	11.20480653081493
C	8.83682575033056	6.58161302613331	14.28369746713424
H	8.43682551949182	6.13082142566807	15.20876736579637
H	9.93536539890977	6.51297354303597	14.31573832152335
H	8.56031894329421	7.64937866610822	14.29293523152169
C	8.29089097497029	3.78314171583769	10.02367933852306
C	9.33234512964236	4.40107919682767	9.08262191357589
H	10.29157183971569	4.57348269458136	9.60068114502046
H	9.52350767951037	3.72830033116980	8.22733597350741
H	8.97862445475687	5.36881436214006	8.68892103253492
C	8.81047854966704	2.43332897625418	10.54136209047672
H	8.07918791682164	1.96906134735829	11.22558589626915
H	8.98307624564367	1.74156864732541	9.69693198148135
H	9.76197346407456	2.55040655053243	11.08846282845755
C	6.98192403364902	3.56419248730402	9.27617772004212
H	6.59995571509461	4.51305237032547	8.86451018715881
H	7.12389209570836	2.87157385099523	8.43070426552639
H	6.21573260441726	3.12806386419418	9.93921865474281
C	4.43705612187255	4.53758075959072	14.82679259504811
C	3.59055957060986	3.49504520132360	14.48344090236431
H	3.08284026743984	2.82220668849252	15.16991728864160
C	3.53811360085977	3.49755029181431	13.07575469431622
C	4.85646738590025	5.00143238506460	16.17938386980125
H	4.56244833087824	6.04956772608173	16.36301811645692
H	4.37895604379542	4.37361101985812	16.94811108637470
H	5.95012348584648	4.93946126272579	16.31647764582049
C	2.76831182100849	2.54931686228187	12.18448116927831
C	2.05665455590634	1.49540473479815	13.03655594605134
H	1.51204259022252	0.79216837194353	12.38225723557624
H	2.77308662220526	0.91101793546708	13.63983483318679
H	1.32336351937182	1.95607213278455	13.72135528202040
C	1.71597199535955	3.32725705300648	11.38363007813275
H	1.08703056932643	2.63328243243764	10.79773576174175
H	1.05693053186028	3.89869658732227	12.06106238629848
H	2.19206260112091	4.02844806191291	10.67968748322776
C	3.75018355486500	1.84363013410075	11.24253528500295
H	4.26312403849660	2.57633836249014	10.60076743984834

H	4.51409906162685	1.29160644821638	11.81804883578265
H	3.22096088348318	1.12717864113633	10.58943365283367
B	5.79485175146038	6.30700622509069	13.51805433256662
H	6.18042827386099	6.67309556092068	14.60689665406407
C	6.23441752080340	7.90264014650241	8.74379509227084
C	5.34371318008484	9.13996912301239	8.54310461661180
C	7.32933747990803	7.89505516105219	7.66608304805806
C	6.93417024476936	8.03649407113871	10.10324672579891
H	4.58231279188124	9.14778640891318	9.34161309983093
C	6.17608889772834	10.42432556945330	8.60710332744434
H	4.81921788418069	9.05781590787534	7.57553699367482
C	8.16448474638200	9.17787948111480	7.73381243264847
H	6.85964189310735	7.79209596842865	6.67849561624664
H	7.97376999518434	7.00961175710486	7.81628349845603
C	7.76055787172031	9.31971374153119	10.18692143067884
H	6.17933310908243	8.03894388371703	10.90533208774061
H	7.58718092227281	7.16238140937237	10.26274808352247
H	5.51332798257409	11.29431471563172	8.44648328586039
C	7.25520008906212	10.39258159193898	7.52133533573941
C	6.84404812064993	10.52854860845447	9.98154622078732
H	8.93246662238815	9.14885619402161	6.93931350591099
C	8.83982522598831	9.28998849961902	9.10295026113559
H	8.23369253620938	9.37456144478655	11.18426785950133
H	7.85232853060589	11.32338967653479	7.55160595359419
H	6.78382069843149	10.34102115510114	6.52489676944532
H	7.42579532547898	11.46700070068107	10.05401926024069
H	6.07498579985855	10.55479620299802	10.77626972969449
H	9.46067110238702	10.20445703346336	9.15105846194098
H	9.51373180409082	8.42872955261260	9.26805259224538
C	2.94474184375111	3.14382202283829	6.61300356712333
C	3.59747806498925	2.04995405910903	7.47236333182167
C	2.84593648054997	2.59950064311161	5.18395452328209
C	1.51714516492989	3.39327066288330	7.12959917491867
H	3.69242210878373	2.40875783359828	8.51030805169264
C	2.77237262058616	0.76019428061985	7.43693208749418
H	4.62127848180859	1.86579325260336	7.09848640519454
C	2.01153188500698	1.31846776595414	5.13086648001921
H	3.86102831255045	2.39833075882561	4.80324018335888
H	2.38686587893016	3.35911960075497	4.53466824813607
C	0.68952571366198	2.10604078457701	7.09539213905876
H	1.56440902247913	3.79781344949730	8.15637205796509
H	1.05000954023825	4.15908817975470	6.48773065608403
H	3.26196766925761	0.00096773483829	8.07346398908384
C	2.67953781116872	0.24828245409983	5.99682113568075
C	1.36215229192048	1.04065583288896	7.96539165705513
H	1.95868690159575	0.97069194568606	4.08258815493066
C	0.60006131716601	1.60286050275941	5.65137289734564
H	-0.32457445200547	2.31475908098066	7.48194443347650
H	2.10109062554278	-0.69386818794958	5.96062455273895
H	3.69039058606949	0.02766563658664	5.60654830968666
H	0.76351278489868	0.11074070837872	7.95937774602262
H	1.41370796107256	1.38771930337362	9.01247469128717
H	-0.01848405837512	0.68704871224818	5.60216891532070
H	0.11142227193644	2.36511595115922	5.01584477972099

[(Tp^{tBu,Me})Ti{AdN(N)C–C(N)NAd}Ti(Tp^{tBu,Me})] **(3) (S = 0)**

```
Eh = -5351.435730124583
Ti    4.20436472433451    5.76722746669345    5.10502643107110
N     5.26037230223658    4.32342261779188    3.82181851030046
N     4.40333407868466    3.98554628414849    2.82064606715164
N     2.08693897041470    5.80694604066017    4.31801970648541
N     2.04301715624035    5.04925485684604    3.19708373177430
N     4.42837176584338    6.98196575286946    3.18374822796598
N     3.88525677214749    6.33183159610709    2.13146227672531
N     5.02434184591342    6.59145049744713    6.40809970636400
N     5.47545076348073    6.64078414709086    8.89777327560823
C     4.78942775745587    2.82395624831456    2.24997038389647
C     5.94768767601577    2.41305047216285    2.89063700376655
H     6.52691227559032    1.51974082075325    2.66599183723601
C     6.21840061239450    3.38953422523974    3.86487093424361
C     4.06396579212090    2.15853817117616    1.13279828604277
H     3.00255589419014    1.98960177143793    1.37907532136219
H     4.52719820954824    1.18083727833716    0.92825850358441
H     4.09870154493829    2.75567280144959    0.20551935665719
C     7.43732696933782    3.41949062350027    4.75749567199862
C     7.53730574039688    2.08890004326910    5.51549512583318
H     6.65185109110928    1.93699616644749    6.15435261018856
H     8.43285053732034    2.08926904114772    6.16212803512097
H     7.61736420782571    1.22991700199818    4.82688334595987
C     7.40288809800920    4.57373494121160    5.74802325216018
H     7.36579610058170    5.54437023349518    5.22954512831405
H     8.31529720801346    4.55309403277758    6.36632140334211
H     6.53072415671041    4.51873088980473    6.41690325038537
C     8.66970058618973    3.57768467679820    3.85096441752822
H     8.75095284092702    2.74989635920262    3.12524866811315
H     9.58933226441743    3.58960700780200    4.46351682371071
H     8.62000132111446    4.52447604629591    3.28534922724319
C     0.75841183109635    4.77458468164827    2.87115514413588
C    -0.04860011859330    5.40105667649232    3.80993298473165
H    -1.13692082030924    5.39934816165298    3.83130192058810
C     0.83149084039193    6.06161365029008    4.68822802941081
C     0.36110586038438    3.94945620110410    1.69699454789907
H     0.62677015269573    4.43573863407533    0.74241108561007
H    -0.72809509608524    3.79066368853381    1.70901587215849
H     0.85005561932348    2.96090900087297    1.71146407372478
C     0.48822961295886    7.05535132834067    5.77061754957270
C    -0.46704621688567    6.44185403561370    6.80072355102519
H    -1.36172874193697    6.01568465544656    6.31381177427050
H    -0.80763707486331    7.21739377341674    7.50928066330357
H     0.02260024658427    5.64265049172676    7.37819422577256
C    -0.21198155448606    8.24476198902982    5.09067005961303
H     0.43917252710516    8.69689254819139    4.32257731489190
H    -0.45318034034582    9.01905084247472    5.84139618044275
H    -1.15238982439585    7.93540628326965    4.60247817613802
C     1.76297408192700    7.55265478388984    6.43998144003510
H     2.32309896302699    6.72917051699431    6.91315203267542
H     1.52489358611126    8.29050460686162    7.22094906601184
H     2.42115189625419    8.04872314827265    5.70812441278590
C     3.94776692143223    7.08317260549474    1.01412330460257
C     4.57093696162669    8.27329953969982    1.36099109942154
```

H	4.78901621899277	9.10473701480717	0.69631368585731
C	4.86412925077390	8.16732325769404	2.73216740112192
C	3.42134432579684	6.64741844288545	-0.30950423504261
H	3.91232623391558	5.72439259810238	-0.66254050692094
H	3.60000372901901	7.43695947492372	-1.05586761666198
H	2.33589557085672	6.45044407522030	-0.27243761455911
C	5.60059708410459	9.17237563193679	3.58645987672495
C	5.95449165150823	10.40125666641369	2.74533617725933
H	6.50086568929689	11.13082428360637	3.36768203759098
H	5.04980619956896	10.90043818745578	2.35606286417270
H	6.60080367063974	10.13645293923144	1.89067574536312
C	6.89705521713112	8.52159823937811	4.08861989067468
H	7.49401836986258	9.24297850406323	4.67325514054964
H	7.51034341119156	8.17559575756238	3.23793935241686
H	6.67021946839500	7.65928828368678	4.73485621675355
C	4.71762586158883	9.61339990301264	4.76061949973306
H	4.55774336546768	8.77965983412001	5.46263314365423
H	3.73861153688100	9.97441086531428	4.39861813600284
H	5.19975433679899	10.43725836004450	5.31510801842927
C	4.95028310288756	6.17215851482951	7.70001638997530
B	3.29507497182767	4.92858909457036	2.29425910771839
H	2.93575853611090	4.50815667314100	1.21885514530859
N	4.11190438082377	4.57967362452908	9.14146474317277
N	3.78285882910840	4.45197154843063	6.63744776074026
C	4.21566334934537	4.98149898461886	7.84459408971027
Ti	4.78030453461029	5.49653239108214	10.46489719656144
N	3.96582275388700	7.24944891201033	11.60154888040713
N	4.84322306998577	7.51095752158159	12.60037097864709
N	6.74422202709272	5.04845541415086	11.36797148478700
N	6.86290881073701	5.84713023420521	12.46214536037022
N	4.15063892780866	4.53198527297375	12.44132319236514
N	4.68756936181528	5.19225097690413	13.49000588329876
C	4.63096612966157	8.75435251352127	13.09100808718535
C	3.55943608393060	9.29544956716268	12.39556922110461
H	3.11561175483371	10.27676619065886	12.55196178816019
C	3.15623716199527	8.30211379993209	11.48298930497863
C	5.43977648420114	9.35943399950347	14.18471633313062
H	6.51817892621278	9.32650130368266	13.95654906094985
H	5.15138442324054	10.41367079834204	14.31686838045774
H	5.28906619932173	8.83849961133158	15.14589698341132
C	1.92112288313651	8.29262174747299	10.61622494099655
C	1.89268217354077	9.51646866359360	9.69372369113025
H	2.69650736146079	9.47239833139432	8.94212947551503
H	0.92643243164432	9.56602389667248	9.16079550217076
H	2.00880833713441	10.45371927066247	10.26601147178317
C	1.85489891686705	7.00293787964755	9.80893880751048
H	1.82854501908028	6.12563144789674	10.47536045477426
H	0.94075350011934	6.98227083641394	9.19500602103098
H	2.71925765225143	6.89927278094005	9.13262894615724
C	0.70739070342443	8.34686100044294	11.56057817834881
H	0.69709443099354	9.27624351990012	12.15622526492532
H	-0.22877671567669	8.30251150253197	10.97490604408919
H	0.71740500766395	7.49228691027172	12.25956040677877
C	8.15667330554353	5.92508836664028	12.84242901832645
C	8.89118537393345	5.12875583463557	11.97788325355559

H	9.96589634292563	4.96063725523772	12.01072113437112
C	7.96432003672654	4.58808572077892	11.07014403605908
C	8.64422753500443	6.73701048803205	13.99143880465290
H	8.26125854826869	6.36060111924319	14.95562251945206
H	9.74422284287730	6.70334375089977	14.02246865414947
H	8.33692568692765	7.79228578282845	13.90236620065773
C	8.28254796947927	3.61060657030514	9.96438500178258
C	9.32641430898930	4.24306399807793	9.03442031504630
H	10.24786773678491	4.51202081787386	9.57942672644866
H	9.59932309918574	3.53526219591851	8.23186142534352
H	8.92511095190690	5.15717833331829	8.56736677631835
C	8.87619610631719	2.34585101641638	10.60456643788705
H	8.15018141104349	1.87484136958867	11.28986939691778
H	9.13469776087971	1.61297306235245	9.81920434501447
H	9.79225058955221	2.57004662878011	11.17807858644072
C	7.04376948808621	3.22836165957096	9.16732722420150
H	6.57757149381022	4.10222419301405	8.68697751570634
H	7.31876115558948	2.50924813073017	8.37857070242465
H	6.28552982842514	2.75481728603368	9.81056777698135
C	4.27046166496388	4.66634810691499	14.65886290243512
C	3.42666735059012	3.60946162621778	14.34818791897942
H	2.91585747960648	2.96145002629807	15.05531369114272
C	3.38112472634321	3.55524636914989	12.94373294323167
C	4.68677739182163	5.18130795469092	15.99308834233145
H	4.40132814626885	6.23874561909489	16.12956500824978
H	4.20031327323820	4.59079450399579	16.78500684049920
H	5.77917685475333	5.11387070492122	16.13572975546755
C	2.63607317689686	2.55342722888399	12.09404840112003
C	1.86727099257958	1.58164997081036	12.99365132108899
H	1.33273117399593	0.84450175732527	12.36966508634205
H	2.54376434338830	1.02556939413145	13.66581272223901
H	1.11804594325069	2.10683376997052	13.61171318864050
C	1.63668041290980	3.28297295386913	11.18715681187193
H	0.99911532636886	2.55698030900186	10.65369612071088
H	0.97842750405260	3.93977871419196	11.78342529810916
H	2.16386063514769	3.88977950056443	10.43386620002572
C	3.65994157102816	1.76483810850547	11.26570468790518
H	4.19286137277042	2.43367558458310	10.57228165268021
H	4.39945128911644	1.27892123850743	11.92641193210079
H	3.16142080249773	0.97975552424770	10.67117172789321
B	5.64571876231915	6.36396303499231	13.26610725355348
H	6.05419769071807	6.77181675948751	14.32860203506777
C	6.32904086886325	7.83141321109925	8.87234140329601
C	5.48243054377034	9.09921760004818	8.66335193465593
C	7.40641718900335	7.77215017628884	7.77451945916090
C	7.06950832950538	7.97383860805633	10.20682188960779
H	4.73694965779659	9.14801834474508	9.47478491561767
C	6.35576169800551	10.35720943300775	8.68308523839872
H	4.93482642820405	9.01559743766822	7.70895284360726
C	8.28391677488384	9.02828221898010	7.79805482858352
H	6.92094510375546	7.66224743110322	6.79500736828030
H	8.02336990545363	6.86920160711967	7.93101137956561
C	7.93499461107912	9.23280302087291	10.25067347663032
H	6.34149129753573	8.01101963408704	11.02882813083979
H	7.70694383591440	7.08816753838341	10.35678361841546

H	5.71893789344092	11.24565406437667	8.51770387383997
C	7.41269555006310	10.26890508801280	7.57933954289786
C	7.05343131876423	10.46648746010076	10.04180404164193
H	9.03234782097591	8.95684929276355	6.98771576670092
C	8.99233079183479	9.14880106135421	9.14889918757466
H	8.42939082297524	9.29074173110111	11.23840385046317
H	8.04117224352180	11.17964680786727	7.58012597937197
H	6.92067143066083	10.21433670483718	6.59422563460999
H	7.66502971353117	11.38762764692172	10.08660968136425
H	6.29954939826934	10.53108639404751	10.84921812106670
H	9.64121781871945	10.04491149492626	9.16532215596764
H	9.64216658183044	8.27011392588828	9.31932213022924
C	3.07579671477490	3.16952939096311	6.64891481151698
C	3.79839416922403	2.09387270110726	7.48044129126638
C	2.96290759188115	2.61935728892419	5.22269553469802
C	1.65178950009728	3.34696369546398	7.20294019154405
H	3.90944892831897	2.44702809908753	8.51489455201699
C	3.02793980568082	0.76894081953988	7.44912878126306
H	4.81675830073947	1.95669457356877	7.07419019706748
C	2.17910312966380	1.30763558775325	5.17513793422190
H	3.97593868398296	2.45085420236863	4.82537530100766
H	2.46408893307676	3.35810793765300	4.58118863732461
C	0.87930847947209	2.02533547821677	7.17753307035430
H	1.70949647508153	3.75020859691886	8.22836237691545
H	1.13703904618038	4.09429310989753	6.57670505591258
H	3.56644023571978	0.02756401978717	8.06739091928806
C	2.91529676682601	0.25930039447869	6.01077344439243
C	1.61977949762281	0.97924555584483	8.01432149039283
H	2.11427856672208	0.96939018682956	4.12408510289033
C	0.77111509859008	1.53079854856177	5.73236423002787
H	-0.13199392984427	2.18687478167292	7.59353739240908
H	2.37580397367530	-0.70633494357219	5.98454416578911
H	3.92242518843388	0.08431514381902	5.58873108573478
H	1.06239882320541	0.02361346736037	8.00874933503403
H	1.68051036732592	1.31411726504496	9.06286583660668
H	0.18736160048414	0.59186123460821	5.69043449216373
H	0.23681382843265	2.27840754967750	5.11614382461892

[(Tp^{tBu,Me})Ti{AdN(N)C–C(N)NAd}Ti(Tp^{tBu,Me})] **(3) (S = 1)**

Eh = -5351.490087578523

Ti	4.23505452669620	5.81895850008203	5.04113190494699
N	5.21851986151811	4.27700770127471	3.75975016452274
N	4.38160159041169	3.94030203772667	2.74992699946873
N	2.14739673920160	5.87521034239656	4.20884009850249
N	2.08749868650763	5.10132964041332	3.09982943146717
N	4.51131439650284	6.96949624498407	3.08139720140480
N	3.98805670811047	6.30298245666721	2.02832446334341
N	4.95779061453657	6.71838416009665	6.45379271061838
N	5.47487776974466	6.67897963882384	8.81584304738724
C	4.75468013093642	2.76176473944881	2.20305160232416
C	5.88920834209498	2.33709551185909	2.87685276731840
H	6.45864140158917	1.43126506093660	2.67837627908728
C	6.15762024512585	3.32778371357090	3.83879321126827
C	4.04078499631789	2.10496282106704	1.07340980723484

H	2.97071147768668	1.95922433970881	1.29567472827103
H	4.48764936322676	1.11695410885276	0.88273086111959
H	4.10827294317245	2.69726372807370	0.14465725079323
C	7.36361934258682	3.38799030349600	4.74578029709252
C	7.48625765975785	2.07535691990877	5.52904811852458
H	6.59654579911431	1.91163098881032	6.15950371182612
H	8.37388538714637	2.10696263497691	6.18597163533287
H	7.59503450802814	1.20751403348211	4.85568576656182
C	7.26491417321297	4.55792008312088	5.71228669945309
H	7.19160761891656	5.51779661989480	5.17505483251293
H	8.15901654540998	4.60153428865478	6.35547596835822
H	6.38406292782621	4.45907823748840	6.36360061173177
C	8.60666835505103	3.56990242642824	3.85999754718239
H	8.72115076721031	2.73502860438128	3.14707997665005
H	9.51580889476849	3.61537764408880	4.48675305055449
H	8.53959174648156	4.50689247035059	3.28012194886504
C	0.80222364154301	4.80173322517095	2.80558473304740
C	0.00642404593724	5.42888372685317	3.75284414831244
H	-1.08089856274850	5.41280836266550	3.79619059087370
C	0.89281040558212	6.11416012483034	4.60405667354295
C	0.39101375539106	3.95089987242813	1.65482845277475
H	0.65540271932949	4.41044960778702	0.68692303628696
H	-0.69943516096613	3.80224418078489	1.67770013205907
H	0.87061567391254	2.95830941753712	1.69154961271271
C	0.53606730832344	7.12517576390742	5.66748974324338
C	-0.51345847532348	6.56395212104281	6.63256718837251
H	-1.41050336513897	6.20991860048159	6.09531122085445
H	-0.83381267995849	7.35115206120005	7.33775654182377
H	-0.11157797280110	5.72376990132829	7.22137970532511
C	-0.05414343839370	8.34950297970930	4.94642596058256
H	0.67273981023817	8.77036302251042	4.23035520092241
H	-0.31140593907037	9.13341385033672	5.68168629022656
H	-0.96929856070908	8.08502150716984	4.38889305454859
C	1.78047815169108	7.55144697910569	6.43117851740879
H	2.24807493167530	6.69311838033069	6.93712539531010
H	1.52709026343640	8.30201356262251	7.19650363619495
H	2.52624344144091	8.00503554211968	5.75820717013446
C	4.11256713327578	7.02258129589867	0.89241814012157
C	4.75049187003009	8.20568748543609	1.23054479319902
H	5.01189251617765	9.01427142743374	0.55328565337045
C	4.98602162056224	8.13164356701796	2.61645513054071
C	3.62657746248629	6.56369846939787	-0.43893317762268
H	4.10375562915754	5.61731290301352	-0.74659898596729
H	3.85948337651127	7.32615097098357	-1.19851708150946
H	2.53510114833137	6.39907808720057	-0.44245357262133
C	5.69157744492377	9.15286278312174	3.47898031376966
C	6.08262575601884	10.36322300958421	2.62789888681135
H	6.60548831571661	11.10523437084484	3.25583216478521
H	5.19504136196026	10.85491210569600	2.19252838923660
H	6.76134091177982	10.07959852867419	1.80492996160441
C	6.96340822263325	8.51400089333579	4.05109188110181
H	7.52743161083083	9.24316902771155	4.65873646754515
H	7.61986193924943	8.15907925666200	3.23712575742677
H	6.70728233099677	7.65920301006222	4.69613302988323
C	4.76378037190474	9.61864616033557	4.60844614652496

H	4.57696983398152	8.80207302821993	5.32429620727005
H	3.79945258090240	9.97180899718100	4.20211877039264
H	5.22609422516981	10.45406007403930	5.16338527784894
C	4.93218926015518	6.24698247061312	7.68415078898304
B	3.33178004159997	4.92837459203221	2.19783421004562
H	2.95966383453452	4.51485420088518	1.12347367540054
N	3.97953429198334	4.55487824117998	9.12429598163938
N	3.75503428050828	4.41777341072144	6.72266364698244
C	4.14352540927851	4.94620941269354	7.87612990336389
Ti	4.74974334539633	5.42678463968715	10.52861521299318
N	3.96298084336893	7.16492569921663	11.70032728303584
N	4.85139612963928	7.45444104377302	12.68034964537071
N	6.76907066675417	5.09301510188366	11.43066644790786
N	6.90121058997036	5.86612674744006	12.53442314242699
N	4.20922581091703	4.46134809800990	12.53068846821741
N	4.75342718734433	5.12790816597083	13.57238972977158
C	4.66000613983077	8.71609836344388	13.12847624465259
C	3.58809693698068	9.24423372426242	12.42470010919861
H	3.15495085589898	10.23440195689238	12.55205350360805
C	3.16262751984090	8.22459243577755	11.55211995845601
C	5.48961730647857	9.34860121866553	14.19038569680331
H	6.56336914768416	9.30890736344652	13.94197087380997
H	5.20448358510538	10.40617502047872	14.30113684441180
H	5.35752130875283	8.85187454288650	15.16692700967530
C	1.90108871540518	8.21151451838657	10.72060625276607
C	1.83394343689694	9.44158616477452	9.80863456737793
H	2.61514496248012	9.40905566754118	9.03290644706613
H	0.85238883154278	9.48340277575890	9.30369857024692
H	1.95775803859650	10.37764513228177	10.38092778011372
C	1.81582125194998	6.93459344760264	9.89762567253964
H	1.82413197909070	6.04428706752696	10.54730024859783
H	0.88363696943594	6.91321032437595	9.30872563247103
H	2.65930383598685	6.85443549271105	9.19580195022625
C	0.71826190905844	8.24634438993170	11.70475257903067
H	0.72511370457016	9.16660028456979	12.31425878956114
H	-0.23708114547731	8.20605807936557	11.15065483310697
H	0.75635680944897	7.38165775251703	12.39009646921613
C	8.20093401826323	5.94998450721567	12.89668323131624
C	8.92787471119937	5.18316787257040	12.00011445013384
H	10.00431147798166	5.02280712217304	12.00979666701912
C	7.98726212102636	4.65184077910964	11.09935418584085
C	8.69352404611438	6.73301920564539	14.06343840969714
H	8.31695656565242	6.32940095457765	15.01927103499780
H	9.79377901092511	6.70188257477409	14.08781560366761
H	8.38312923412313	7.78964498212124	14.00583853284925
C	8.27819304410903	3.68876393208073	9.97364069833199
C	9.30484537871359	4.31904917460235	9.02369474622717
H	10.24180269724569	4.57319178839131	9.54886183041170
H	9.55169020007885	3.61615194708042	8.20815977649178
H	8.90359462457203	5.24271291934470	8.57488566382242
C	8.86669011660214	2.40492319330303	10.57822551520202
H	8.14962245922293	1.93387731946566	11.27277078372815
H	9.09845425078693	1.68057198964216	9.77656693829202
H	9.79777919344028	2.60827120700349	11.13494324657395
C	7.00967965934050	3.34649715075429	9.20733507594804

H	6.57164641307752	4.24590086651953	8.74917114525933
H	7.22866442034668	2.62964417635785	8.39934792746454
H	6.25289987730910	2.89001601512455	9.86591985684028
C	4.37279786449247	4.58081339241575	14.74688187310934
C	3.54837252712363	3.50777141397711	14.44491500983004
H	3.06778162239406	2.84189591235599	15.15675553572813
C	3.47369325169076	3.46559878052914	13.03974019543967
C	4.80388494648753	5.09380037307542	16.07742446396629
H	4.49167527019487	6.14107321575330	16.23285161128847
H	4.35126185805908	4.48104521459748	16.87260451545428
H	5.90056629599694	5.05627511832828	16.19730922154901
C	2.72356288970712	2.46494617334876	12.19129326176722
C	2.01105250138858	1.45355580728590	13.09291678256494
H	1.47342132049704	0.71661600336277	12.47126789405431
H	2.72503188751828	0.90140362138390	13.72866301276602
H	1.27139471061369	1.94535648880547	13.74872712900381
C	1.67740163019977	3.19068104801318	11.33537067524269
H	1.04592632358471	2.46054971227000	10.79941996688301
H	1.01909123601701	3.81204747140473	11.96820634058595
H	2.16457409516007	3.83145697467978	10.58291379431690
C	3.72885657374325	1.72073315835900	11.30267633595799
H	4.21768616431323	2.41844016486252	10.60479119372011
H	4.50514587339934	1.23235613795853	11.91773172553782
H	3.22106040776138	0.94346028980695	10.70505107954451
B	5.67721377061386	6.33050036561608	13.35201740048120
H	6.07485529398384	6.74737061229166	14.41590196385007
C	6.29776515850273	7.89162968292450	8.81947508121305
C	5.42073711090247	9.13948976698883	8.62103598717827
C	7.39639082122146	7.87792013587100	7.74323001607767
C	7.00014272744113	8.01924128221888	10.17632504751388
H	4.66251534195146	9.15518235653269	9.42225380484869
C	6.26414791769980	10.41637674254999	8.68294809080096
H	4.89245691727672	9.06176570199071	7.65541892791404
C	8.24254584364000	9.15306696258402	7.81340910825585
H	6.92978495301655	7.77816158632834	6.75386586043819
H	8.03267438715802	6.98729361944079	7.89604269671581
C	7.83537317634724	9.29649862832030	10.26421051297274
H	6.24694155819076	8.01981142395151	10.97778574411110
H	7.65092604008897	7.14268636944160	10.32827504430935
H	5.60934244244053	11.29220201633146	8.52163223205361
C	7.34399781868659	10.37492421536192	7.59832442769049
C	6.93113150150644	10.51451315335006	10.05822327712359
H	9.01122522399523	9.11727810824933	7.01987392214256
C	8.91709998910946	9.26010620149595	9.18309469782781
H	8.30773516882326	9.34680707540334	11.26295779416930
H	7.94818536363325	11.30133412889853	7.62746138611674
H	6.87306629846132	10.32430397345574	6.60179839995284
H	7.52181167525289	11.44729605510870	10.13272195450398
H	6.15959995928884	10.54842753333725	10.85060364766003
H	9.54464867320912	10.17000446890976	9.23314872516380
H	9.58369153885683	8.39319994745735	9.34875493358713
C	3.01142360160918	3.15629067758150	6.71342875850978
C	3.72474553167809	2.03409280320239	7.48730042636071
C	2.85454331721732	2.67556938835625	5.26668776554756
C	1.60521904663981	3.35428797027259	7.30330443069888

H	3.86630187139682	2.34776763651728	8.53091077927339
C	2.91872727745921	0.73264696200582	7.41964494631282
H	4.72849822136291	1.88455032807917	7.04929924977141
C	2.04093679685451	1.38405331722534	5.18331396407426
H	3.85394255836695	2.50874123188269	4.83389442068890
H	2.35603459322353	3.45740341016238	4.67517263743836
C	0.79907694104874	2.05389678526634	7.23965041554078
H	1.69636729349399	3.70937770357445	8.34345533565540
H	1.09845855111779	4.14125330107572	6.71873430739769
H	3.44828949710694	-0.04744938260874	7.99646188759948
C	2.76706062328371	0.28635195848645	5.96339953531825
C	1.52870138451119	0.95876183371502	8.02269023109432
H	1.94716164619335	1.08840938078459	4.12179070987124
C	0.65037872603545	1.61738437849736	5.77911611468793
H	-0.19943798685314	2.22405581680807	7.68174380026616
H	2.20330891475766	-0.66404952792436	5.90851597963309
H	3.76161630478687	0.10419760632295	5.51551353710554
H	0.94575456512960	0.01900399602696	7.99414857126691
H	1.62140913194956	1.25258914305761	9.08201241620020
H	0.04466951741355	0.69377235554508	5.71459840431004
H	0.12157781655237	2.39935884110236	5.20230175120653

[(Tp^{tBu,Me})Ti(η^3-NCNAd)] **(I(η^3)) (S = 0)**

Eh = -2675.646603996399

Ti	5.12776666250205	11.94768139083202	9.70423826273360
N	6.91902880055899	12.08812778238875	9.92486741168320
N	3.97662758889252	10.12036153756144	9.49270179445243
N	2.70105846747767	10.52789728083582	9.29469095465445
N	3.99451783545538	11.90360170645132	11.64033619195996
N	2.66964072342266	11.89865636317329	11.37598243774851
N	5.65071384700326	11.97012113571842	7.92717448054152
C	1.97620348051705	9.52291826706361	8.75473221137861
C	2.82229999719283	8.43199477395173	8.61350514098149
H	2.55174181890657	7.45445150426621	8.22195887747658
C	4.07577951532058	8.84835185029445	9.10025410642465
C	0.52935570405930	9.63381505823884	8.42116868404250
H	-0.09191512360576	9.76508581028471	9.32382486064608
H	0.19845072963819	8.71744356233326	7.90850573468235
H	0.32891917839330	10.48949820576759	7.75493140270241
C	5.35352863626269	8.04231038504467	9.18677984863458
C	5.00098222310906	6.55899798994388	9.34003053587018
H	4.37017013324811	6.38591128443252	10.22935037314771
H	5.92490297042037	5.96540485404555	9.45120244895745
H	4.46451769218206	6.17314622834454	8.45613281831101
C	6.18619368480734	8.48796396618570	10.38909540751505
H	6.52711084628959	9.53203582451873	10.28438013674264
H	7.09046820773461	7.86069662318045	10.47429269485057
H	5.61239634624642	8.40210437884386	11.32670434590247
C	6.17601622702542	8.23698991905245	7.90749851833426
H	5.60079560744562	7.94672668944316	7.01065516726844
H	7.09199603565439	7.62067557155556	7.94575842681187
H	6.47853995566913	9.29033492370486	7.79952251832250
C	1.95728055730322	11.91483612004489	12.52088591066257
C	2.87034394292442	11.93081999925979	13.56665387094579

H	2.63313212543299	11.94543766362608	14.62715780775593
C	4.14573458489666	11.92164381833911	12.96964123730350
C	5.49128410698807	11.90970966164288	13.66449091727872
C	5.27415051777948	11.94127127249231	15.17946961982352
H	6.24943078442157	11.93067196778504	15.69565254548319
H	4.73672784225401	12.85357565367727	15.49230658409534
C	6.32498030202818	13.12779992854152	13.24964179572242
H	6.60188001776041	13.07337359916442	12.18475234200713
H	7.26138489493743	13.15522272198573	13.83489627398356
H	5.77626778649142	14.06775818003046	13.43211129368332
C	6.85330082718025	12.08999865660638	8.58026407623593
C	5.45000139637876	11.96114704822557	6.49274794024701
C	6.63177700398843	11.35659948584456	5.72562676915228
H	6.82451040733238	10.33228371092451	6.08490469877670
C	6.32587517866355	11.33749952235632	4.22420574307821
H	7.18152783272072	10.89068625252971	3.68744998213729
C	6.09587560806898	12.76661667258393	3.72384238842955
H	5.89403873774778	12.76173696254515	2.63638539184348
H	7.00554101083013	13.37446537820498	3.88174379651244
C	4.91560711322341	13.38395372379390	4.47931205431286
H	4.75608774338004	14.42227985510472	4.13727481410714
C	3.65321393086859	12.55627886769451	4.22222835424076
H	3.40886947602338	12.55889328803581	3.14358484134298
H	2.79226136209658	13.00543232868418	4.75250136024454
C	5.22823319908820	13.39204258868660	5.97768180559955
H	6.14029021863176	13.98033127767201	6.16994289758929
H	4.40539956080890	13.85915885570902	6.54755814444835
B	2.17538030774239	11.84700337988564	9.92161106351289
H	0.96594278155965	11.82696871021471	9.88579855793232
H	7.53917090964495	11.94626190559865	5.94136890404828
C	5.06640480015102	10.50239527253158	3.96856297994145
H	4.85574041805939	10.45449191883471	2.88395239924838
H	5.22472258077289	9.46388951109677	4.31447794989300
C	3.87908997443726	11.12291218325536	4.71289166387194
H	2.97146075002127	10.51893042736466	4.53376564137134
C	4.18121463351534	11.14320051538269	6.21168060993774
H	4.31293776341399	10.11676058849941	6.59445033834601
H	3.34165990237132	11.59039324453683	6.77357865692186
N	3.86401209770645	13.68854234743271	9.51997431853768
N	2.66007261213424	13.14920802518691	9.21245070557763
C	1.90724686804841	14.05959915970329	8.55519868950074
C	2.65601104669561	15.22597829471522	8.45954646266096
H	2.33475674392367	16.16072353618387	8.00653218742621
C	3.88158072003966	14.95505138016921	9.09739881339552
C	0.52330935835723	13.79012967863712	8.07675123360545
H	-0.17815443914996	13.63544871653831	8.91463691183192
H	0.16435242264716	14.64395446224450	7.48179680080542
H	0.48323170664779	12.88941023066981	7.44090947957470
C	5.03161085827528	15.90325718179698	9.36952317495886
C	4.49308377254286	17.33817063643969	9.39600243197555
H	3.69356336009045	17.45516841643178	10.14832336305645
H	5.30886447440666	18.03729531763159	9.64916973026270
H	4.09074459603831	17.64142263293987	8.41384924649635
C	5.65523396516282	15.57209126115207	10.72701580848651
H	6.11904959189040	14.57348741486387	10.71662451499944

H	6.44829816555646	16.30214937387519	10.96465113019594
H	4.90137037041232	15.59618749872853	11.53247233602794
C	6.11175814599530	15.79636199229646	8.28667439175291
H	5.70299453992217	16.02763279347790	7.28871861853829
H	6.92408518549172	16.51317879252348	8.50171362509010
H	6.55189296832324	14.78623438514394	8.25660264988669
H	4.69941052307844	11.06467765211406	15.52609042520467
C	6.25614536952791	10.63364254430156	13.29321393494076
H	6.51024111153051	10.62755782162624	12.22213418837016
H	7.20212618643904	10.58252568553757	13.86100435739145
H	5.66310667760549	9.73139646037832	13.52380051316328
C	0.46864241132440	11.90925227590456	12.56390404477322
H	0.04185070691258	12.78123905143273	12.03939201545406
H	0.05049455814403	11.00305049554602	12.09239562386190
H	0.12819288050975	11.93795288792386	13.61025978734875

[(Tp^{tBu,Me})Ti(η^3-NCNAd)] **(I($\boldsymbol{\eta^3}$)) (S = 1)**

Eh = -2675.683750143426

Ti	2.72303853409005	12.56680604387813	5.27078718167296
N	0.60224109554885	13.00201771661006	5.83164254245632
N	0.15352265491487	12.26120565108739	6.87264662820089
N	3.02731843312754	12.79623987311594	7.45362340035769
N	2.25781990626915	11.98113872920598	8.20972884417829
N	1.90593408312270	10.47792912936501	5.39205106191302
N	1.45440441041635	10.13804399456527	6.61796720317705
N	4.67686453810046	12.44612339082404	4.31526137100043
C	-1.13453623274056	12.55280621921155	7.14060416368295
C	-1.53422914071121	13.52565014171996	6.23356209648990
H	-2.51787106467580	13.98315232288586	6.17294244183334
C	-0.41150214348798	13.78355324667775	5.42954983593318
C	-1.92792160750979	11.90689060592909	8.22415224424473
H	-1.99679113183232	10.81437389237212	8.08598891970246
H	-2.94990299448879	12.31647902600223	8.22596475773482
H	-1.48621226485485	12.08539865084326	9.21944871746291
C	-0.30487152853845	14.78637428335924	4.30040756733852
C	0.66212184545650	15.91046765806107	4.69766665164980
H	0.34061748952363	16.38711808458762	5.64053483269247
H	0.69083074732810	16.68203393373456	3.90788740793406
H	1.68995815218405	15.53402209206204	4.81762696299707
C	-1.68471594236837	15.39543725815617	4.03267601101693
H	-2.42167510695192	14.62251107496950	3.75217197766140
H	-1.61721947241216	16.11907922401308	3.20208618123055
H	-2.06779200053323	15.93544779456265	4.91590233558977
C	0.18539105524728	14.10226657381676	3.01930365954612
H	1.22845091572244	13.76008253482038	3.11589073362786
H	0.17331869553648	14.81840093794417	2.17869007864222
H	-0.45783031384908	13.24428473586967	2.75626357987826
C	2.51229369520042	12.16220611228435	9.52035820816442
C	3.48547037570425	13.14732431356897	9.61614512633969
H	3.91712786423275	13.54044518703752	10.53271086948082
C	3.77094369495530	13.53338702550929	8.29715877321560
C	1.82082811602003	11.42295157492259	10.61378285352988
H	0.75479506495167	11.70001914585822	10.68513720881413
H	2.29912664620173	11.65882386729430	11.57727832326560

H	1.86694154959703	10.33150005379082	10.46396065318577
C	4.73245244810355	14.61584580179613	7.86140380712151
C	5.17340897302944	15.43540477231272	9.07784625003059
H	4.31138478829822	15.90205846683264	9.58566952710687
H	5.85463313053836	16.24089321665925	8.75361860376274
H	5.71581891420371	14.81593996072310	9.81257156748498
C	4.04810682321475	15.55727332489529	6.86439519765490
H	3.78948703580011	15.05589997458437	5.91746099954689
H	4.72294173317523	16.39084389716078	6.60143524473981
H	3.12252323468737	15.98107169932766	7.29082057949192
C	5.96295765718036	13.96675951009462	7.22424362010760
H	6.48039551395713	13.31068392323339	7.94651410256325
H	6.67546743612535	14.73831934394426	6.88145458022530
H	5.66326302429251	13.35835442224124	6.35806684127975
C	1.05035963671628	8.84729732962899	6.63869548419604
C	1.24300439479506	8.33951958038036	5.36492363600149
H	1.02055064089554	7.32830589480667	5.03055966600602
C	1.75661096063830	9.40717954127221	4.60317833861100
C	0.48150269187774	8.17515472589840	7.84040374043828
H	1.12600225315310	8.30421087772812	8.72602901431483
H	0.37636200608692	7.09657990905306	7.64535361233723
H	-0.51579036509093	8.57294119000685	8.09803853573477
C	2.03209543006243	9.38724243520838	3.11815289994570
C	0.82111519475079	8.77164829228336	2.40005414687304
H	0.63717211949184	7.73301979875362	2.72326466124663
H	0.99618160828806	8.75834140630101	1.30952597220040
H	-0.09350941330224	9.35777187246922	2.59539487990769
C	3.26539107367419	8.51329736726141	2.84949481161322
H	3.52590289717964	8.51976884286896	1.77590502668526
H	3.07475835465444	7.46799837639264	3.14903466436493
H	4.13754858559420	8.86833523383648	3.42086179559330
C	2.22810437916402	10.80499594977872	2.58646815477893
H	1.33142020808687	11.41925336109813	2.77746033471271
H	2.39863652137227	10.79217334919416	1.49612455989340
H	3.10747204337689	11.28917655501228	3.04183364924728
C	4.23382445066491	13.54589086896005	3.78683445505761
C	5.94481521776742	11.83339643432820	3.93149244322154
C	5.86611891393680	11.24234044209906	2.51179764753153
H	5.03541222989953	10.52312716335724	2.45747014257195
H	5.63882916229984	12.05565925704135	1.79721199138080
C	7.17858872365161	10.54823898525351	2.13770088835630
H	7.08668316447004	10.11993600826211	1.12365626585186
C	8.31952491364157	11.56658176724786	2.16959988066163
H	8.13397485826571	12.36687990084401	1.42970952549419
H	9.27359628835383	11.08149669455596	1.89101637369907
C	8.42090418270353	12.16418865799111	3.57522004416429
H	9.23500003606541	12.91018557882548	3.60481871143262
C	8.70732308430495	11.04926514502969	4.58681012367151
H	9.66912461600946	10.55879676958086	4.34794478298227
H	8.80415634710473	11.47595076363198	5.60216569084867
C	7.57005084030618	10.02248106629994	4.55493887013192
H	7.77687068590275	9.21707724150489	5.28151706638165
C	6.25542116668206	10.71300804689520	4.92768403932730
H	5.41680894114820	9.99305606617719	4.93876240721993
H	6.31618561361984	11.14086517044823	5.94406698135748

C	7.46080416100927	9.42919023732612	3.14605267271689
H	8.39840696606173	8.90656873633155	2.88027391954408
H	6.65198603206219	8.67579653483651	3.11276572420628
C	7.10040207907275	12.84857745193332	3.94144110731210
H	7.18215154545519	13.31238990512731	4.93697638657212
H	6.87375329059001	13.65933194260963	3.22371080543102
B	1.05716066285091	11.24257175779103	7.60461375324699
H	0.42782457046175	10.74079665579903	8.50708997381488
N	3.55322370306781	14.48168544635086	3.52009441414865

[($Tp^{tBu,Me}$)Ti(η^1-NCNAd)] **(I(η^1)) (S = 0)**

Eh = -2675.644881085509

N	1.28648007243336	13.74125539412441	5.28645222109193
N	0.70174443302315	12.90432865523234	6.18077326389914
N	3.68850644463012	12.72534023086377	6.73073388778172
N	2.73031903319571	12.05843116430484	7.40978642222320
N	2.51189978562380	11.18694348429052	4.47324019477618
N	1.75752293907031	10.72873249392919	5.49818813042363
N	5.22936368311296	15.63193424491359	1.67212266130632
C	-0.55319080023257	13.33541362449825	6.48289001859314
C	-0.77260993285125	14.49082299267918	5.75501669765843
H	-1.67703460773792	15.09430345264182	5.76165727767725
C	0.40406833564632	14.71732832087140	5.01300023299190
C	-1.45748026370702	12.62856132045078	7.43029750551618
H	-1.68009473683787	11.59784437049270	7.09985751723803
H	-2.41054850192968	13.17516674173679	7.50463648558410
H	-1.02510761004953	12.55854929162554	8.44462989911090
C	0.70648760337836	15.83328283907662	4.03993572222433
C	-0.49306801562699	16.77831756396975	3.94726939185497
H	-1.39427560357974	16.25020636886544	3.58979099380237
H	-0.27471356582937	17.59572389998881	3.23810269127738
H	-0.72456469329416	17.23169806375117	4.92685511206543
C	0.97686827325373	15.24310336554429	2.64948446342726
H	1.83989163901652	14.55682517962471	2.66677088574637
H	1.21060920009655	16.04290093109139	1.92431449144308
H	0.09937705796586	14.68134400993407	2.28438189191470
C	1.92582144461987	16.62946893505489	4.52396284181377
H	1.72104310837224	17.09535896737393	5.50348756682563
H	2.18358215915543	17.42533005765516	3.80274565764809
H	2.81477967492100	15.98457585824098	4.62999471478278
C	3.11765375071529	11.86112430403756	8.68512890900025
C	4.38008006561097	12.43349031534822	8.82892901210025
H	4.98185342415170	12.45099230221311	9.73442338640482
C	4.70872941551038	12.95912143302261	7.57081058341712
C	2.28796731460428	11.14556921204469	9.69356079451957
H	1.31857526929790	11.64897155459114	9.85332567789578
H	2.82078626838277	11.11247435185821	10.65628006601748
H	2.07309737868857	10.10850277101225	9.38365640318179
C	5.95531844529010	13.68016397730723	7.11811148912965
C	6.95852830908245	13.74918019410840	8.27047533595085
H	6.54578848712304	14.30459121267195	9.13048117663579
H	7.87543128977030	14.26796087174852	7.94187482536128
H	7.24428453799400	12.74000569414961	8.61515148555992
C	5.58887996170842	15.10500062360837	6.67817965386297

H	4.91275898571597	15.09649790573468	5.80475383871755
H	6.49465973669106	15.66157627437961	6.37923300289527
H	5.09342139043497	15.65645889638070	7.49602848164115
C	6.58448727928759	12.91579694110859	5.94430299740390
H	6.81643227777340	11.87522599137390	6.22960093780752
H	7.51785136214608	13.40921295431282	5.62036004818576
H	5.90802328043979	12.90199709917935	5.07073499980334
C	1.33505302633697	9.47368801001630	5.23915428943843
C	1.83314862471301	9.11746560818112	3.98938447682148
H	1.68037900006103	8.16899989100377	3.48033383032036
C	2.57425093337600	10.22168388569097	3.54158369324500
C	0.48901747517328	8.68197814458865	6.17358925391897
H	0.96452856110640	8.57206969784925	7.16300550331454
H	0.32089530907150	7.67598956436928	5.75920882505458
H	-0.49526673034826	9.15647746536636	6.33295391302619
C	3.32317917016955	10.41287717523291	2.24399823617837
C	3.22555938279054	9.14220416968636	1.39840925537534
H	3.66376197115638	8.27578321607924	1.92356817497873
H	3.77522429922353	9.27751936685452	0.45095837556971
H	2.17685240778280	8.90265019374193	1.15090880211545
C	4.79971185374457	10.71529428172992	2.53330346436344
H	5.35836042897643	10.84276820129907	1.58927758166772
H	5.26662205178566	9.89727568776400	3.10820557507199
H	4.90595709399671	11.65384545116632	3.10695452292879
C	2.69425966639894	11.58048068766736	1.47074703609669
H	1.61892434485328	11.40660334812456	1.29508971379879
H	3.19260757260559	11.71227769884680	0.49339528832166
H	2.80146847676634	12.53125687160970	2.02108902319151
C	4.74290329023625	14.90762402407439	2.53696970757334
C	5.14984002173057	15.43833110358608	0.24003203185617
C	5.86021491818558	14.13727211122321	-0.16999167125883
H	6.90867542240046	14.17807498894255	0.17686085091854
H	5.37956995263035	13.28600752332095	0.34882616129112
C	5.79625756598965	13.94875680130984	-1.68813652077717
H	6.31126514983081	13.01064197414592	-1.96276630337804
C	4.33041101210745	13.88182948512710	-2.13015504612612
H	3.82862981355595	13.02098714871795	-1.64910635115775
H	4.26988554530802	13.72326604045195	-3.22286244708318
C	3.61841773191921	15.18346190052807	-1.74600295042761
H	2.56089320762769	15.13608429733265	-2.06136637536763
C	4.30944630479110	16.36614294308686	-2.43212370946774
H	4.25295587234512	16.25483475935647	-3.53079023627878
H	3.79037612879572	17.30799209390892	-2.17516384931823
C	5.77296493768108	16.43420042445288	-1.98436761781679
H	6.27090997896840	17.28956409195821	-2.47417150711092
C	5.83383372937877	16.61438316410810	-0.46492894385209
H	6.88033837275030	16.67804285787937	-0.11695689421605
H	5.33340289975879	17.55108384561537	-0.15992547685082
C	6.48385953438474	15.13401283218277	-2.37451906775284
H	6.46138563107979	15.00312055960942	-3.47241870439627
H	7.54754721473233	15.17923512294218	-2.07661589106185
C	3.68669111251549	15.36806781224516	-0.22786721731424
H	3.16868306984777	16.29481185347734	0.07903935737587
H	3.18162878497274	14.52931426798214	0.28590362904564
B	1.42011143355814	11.65164444777901	6.69471270722554

```
H     0.71102843715599   11.05349421402279    7.47264753130598
N     4.29960924428509   14.24762110584767    3.45058282605952
Ti    3.24912373947736   13.16291347389502    4.70972157337240
```

[(Tp^{tBu,Me})Ti(η^1-NCNAd)] **(I(η^1)) (S = 0, Isomer 1)**

```
Eh = -2675.689116691016
N     1.25092693946089   13.65647601739610    5.08236275036700
N     0.63200808243580   12.93958460577417    6.04718540323945
N     3.61717732862857   12.81468465323971    6.75804718078336
N     2.61509368871294   12.22619082827109    7.44706446716552
N     2.52808563162599   10.95313758712865    4.65864605297034
N     1.69154824701687   10.67069565348513    5.68079945728786
N     5.64274510114468   14.36891999203285    1.20052137196223
C    -0.60973385092045   13.42754873293318    6.26506092885368
C    -0.78938937244218   14.50044053724353    5.40076458206522
H    -1.67964286000854   15.11858171775371    5.31661636148726
C     0.40773949796235   14.60961539366330    4.67120678127341
C    -1.55532198698250   12.86175407224919    7.26743613269944
H    -1.80388301537458   11.80897109711200    7.04763417398433
H    -2.49050498061782   13.44255925552089    7.26096249551331
H    -1.13880305315419   12.89412919797239    8.28893731052812
C     0.77852102396088   15.58505534895356    3.57740298675654
C    -0.37705287434202   16.55968863980864    3.34534867446105
H    -1.29530578469480   16.03064178144128    3.03651979087280
H    -0.11309804250291   17.27394632341673    2.54633167989783
H    -0.60256754000664   17.13970281583081    4.25705651579693
C     1.05134528223358   14.81007651632080    2.28088543095741
H     1.92216071223983   14.14119323064314    2.39134384468332
H     1.27851571229575   15.50733670442749    1.45454905751501
H     0.17809053271964   14.20016147240439    1.99140030763976
C     2.03002289500426   16.37486852898534    3.98431156630206
H     1.86331996951848   16.91879964213204    4.93014514340989
H     2.29062738832746   17.10933189486094    3.20135412063520
H     2.90481117742214   15.71236268545232    4.10700897862878
C     2.94394756580697   12.13092228249374    8.75502969667652
C     4.21055587075399   12.68348616319585    8.90367403222487
H     4.77375634122914   12.76696003507773    9.82997647258633
C     4.59885263696253   13.10217724985789    7.61856369891092
C     2.05320543191083   11.52872608570514    9.78610251930876
H     1.09263894792381   12.06695847013686    9.86206743324999
H     2.54670989105808   11.56944014718016   10.76937247624029
H     1.82194038604065   10.47284965525934    9.56275269417845
C     5.88388817210089   13.75406295882512    7.16202071360573
C     6.81349293639128   13.95054711951844    8.36058580756265
H     6.35337486125212   14.60235945983931    9.12336541016914
H     7.75579641423377   14.42362645622529    8.03472324646091
H     7.06515717034055   12.98660178803554    8.83616380523519
C     5.57974075904148   15.11844174695304    6.52699030991923
H     4.95483575036758   15.01343228681074    5.62252638818623
H     6.51703730216309   15.61259105449673    6.21592422382814
H     5.05645861843786   15.77908786725128    7.23979149150129
C     6.57135598321157   12.84761435925605    6.13143594122172
H     6.74789956000495   11.84001478121581    6.54566636599085
H     7.54197362677183   13.27794889064494    5.82738409785392
```

H	5.96316255546661	12.74695143653911	5.21488387875059
C	1.29523476641910	9.38019175552560	5.61248025358352
C	1.90739164831899	8.81787118268689	4.49822840072862
H	1.80359056614872	7.79193704348724	4.15379723337038
C	2.67724050186010	9.84542214726526	3.92477862542694
C	0.36694586177439	8.75071520148023	6.59245564067916
H	0.76892655215727	8.78655733516516	7.61958764998257
H	0.20692582504744	7.69482705019399	6.32515760808830
H	-0.61547171161160	9.25361733079321	6.60660307602681
C	3.55286082935906	9.82436121653278	2.69177149504626
C	3.58301860067114	8.41185724187565	2.10533912418168
H	3.98964220328781	7.68534790743450	2.83038845830237
H	4.22356218884417	8.38954473218051	1.20678026222412
H	2.57375060464779	8.07646881336908	1.80942104620862
C	4.98169336006845	10.24534790544550	3.06175569199660
H	5.63702743214554	10.19551900434983	2.17451666577227
H	5.39965908976029	9.58975531182673	3.84490994405741
H	5.01573060835876	11.28872696085303	3.42166743356066
C	2.98364899611490	10.79574286164392	1.64722040050907
H	1.94741943700388	10.52642754907211	1.37872019183680
H	3.59964820472882	10.78112933936899	0.73072121163739
H	2.98701311133390	11.83385591033004	2.02258713558284
C	4.99490915780646	14.10381329144778	2.21662272853554
C	5.11505570003610	14.91976792643113	-0.02626953313057
C	4.00582660018340	14.02536999956701	-0.60796699698424
H	4.41119790144239	13.00852333970823	-0.76062933137383
H	3.18647850226971	13.94026098113397	0.13016552783446
C	3.48136925183466	14.60500859016612	-1.92428385163547
H	2.68791879986291	13.94866934728161	-2.32478664822009
C	2.91200939821675	16.00527932283903	-1.67076140624661
H	2.07008390663093	15.94754001640903	-0.95520923855282
H	2.50962370303202	16.42828581730246	-2.61015178489726
C	4.01284313177877	16.91334723265291	-1.11187604459017
H	3.60296367520129	17.92248078217816	-0.92667345715609
C	5.16283529570417	17.00201551232004	-2.12052434352811
H	4.80278849388147	17.44122950454526	-3.06955068112437
H	5.95421831206688	17.67028541676218	-1.73396651594722
C	5.73391809629325	15.60197726122038	-2.37047538804741
H	6.56637083213665	15.66458702959038	-3.09409394514553
C	6.24963718032969	15.01957250363683	-1.05226686882992
H	6.67640699850398	14.01216788034821	-1.20658577287149
H	7.05375955410708	15.64862180186624	-0.63015605852662
C	4.63226406430607	14.69637814712165	-2.93198311780820
H	4.26400460944506	15.09711273715688	-3.89490029967373
H	5.03812174073187	13.68804378551948	-3.13527010431916
C	4.53536587408270	16.32678475227986	0.20272075530511
H	5.32379229211491	16.96981654027267	0.63399775340970
H	3.72162934063160	16.26279513496658	0.95018679834158
B	1.33175698573572	11.74483158558898	6.73377636942566
H	0.58175531615929	11.26145838356565	7.55088921074077
N	4.43770058110104	13.81461033832453	3.24769178196410
Ti	3.25076101679990	12.97814882594027	4.63977662284957

[(Tp^{tBu,Me})Ti(η^1-NCNAd)] **(I(η^1)) (S = 0, Isomer 2)**

```
Eh = -2675.683750143426
Ti    5.39531884390906   11.96258474753965   10.49039743925448
N     6.84742699848034   12.11618841145723    9.02633789024003
N     4.23497459036667   10.29047667327388    9.71750698615614
N     2.96785172845749   10.58098941433255    9.35627425457301
N     3.69971912682664   11.95380417945401   11.92908796464453
N     2.46713501793644   11.93887778905188   11.39432122396009
N     6.84049482549165   11.37629549123537    6.69526088881108
C     2.41292371502792    9.53169046865348    8.71186639241930
C     3.36652711285096    8.52258932056555    8.66957243539363
H     3.24579964567888    7.53815555350832    8.22193828829781
C     4.49849934511781    9.04098737133113    9.32492508955780
C     1.02329655686191    9.53808825491018    8.17590331840668
H     0.27267670353930    9.62623893632863    8.98059230857496
H     0.83261956664958    8.60133906834770    7.62976077487591
H     0.86116447597978   10.37801087605012    7.47960568912696
C     5.80729671440173    8.34686463421049    9.61963000741464
C     5.50290509931882    6.97001007514980   10.22838574241946
H     4.91307700020327    7.06562519133272   11.15719604463475
H     6.44361748172131    6.44224736042319   10.46618870773768
H     4.93159778480569    6.33745546565878    9.52767857295211
C     6.62245657764538    9.15618504334132   10.63062570327965
H     6.94935447586448   10.12252045081081   10.19601851033163
H     7.54330686175030    8.61384606947990   10.90609650924775
H     6.03918089816552    9.31895000738671   11.55579084268300
C     6.60419072868473    8.16688647418880    8.31932130451155
H     6.04267967752465    7.53810392301698    7.60572828273169
H     7.56671132123731    7.66536503309950    8.52758485603558
H     6.80959081623980    9.13216536873710    7.82529595879429
C     1.53193976730415   11.96045979280386   12.37251933320298
C     2.20659778020105   11.99315325509782   13.58733786428995
H     1.75565264197738   12.01731071888300   14.57785325785631
C     3.57510257995513   11.98805429094346   13.25349682847578
C     4.78964432892978   11.99792637105609   14.14902071045687
C     4.83481393996132   10.69192972185193   14.95448949720583
H     5.71848089825389   10.67047972004668   15.61789788834237
H     3.93303074284255   10.58433237520672   15.58128670695812
C     4.71610926978050   13.19635178096348   15.10283905468655
H     4.68640568872279   14.14662109650682   14.54155113615753
H     5.59420627931773   13.21523666800170   15.77331578875203
H     3.80995119365708   13.14701047683481   15.73055702684304
C     6.80734418478748   11.78560830267209    7.86557495109295
C     5.82476485259017   11.65261841810383    5.70116709518180
C     6.14762843810009   10.85005184290658    4.43590415130051
H     6.18613695442197    9.77718353411261    4.69748845793779
C     5.09974308038222   11.10773847818915    3.34989958346985
H     5.35143305651111   10.51880705753893    2.44989877906699
C     5.08592351754603   12.59946260319656    3.00029403449513
H     4.34769534586135   12.79774582205512    2.20065153012567
H     6.07479742888234   12.90517952785584    2.61133622122376
C     4.74078733883262   13.41311630618602    4.25151074596609
H     4.73471398759246   14.49044657750087    4.00540211308758
C     3.35947632966086   12.99754934487712    4.76848871630064
H     2.58736807178162   13.20365107010311    4.00318105290554
```

H	3.10336081457960	13.59215019804086	5.66439423236379
C	5.78471510199074	13.14675073692922	5.33988550288872
H	6.79076041858577	13.46153351968301	5.00824250587167
H	5.54120979731691	13.72648980251670	6.24856923881667
B	2.30074803602416	11.87818500844456	9.85950069398508
H	1.12335565449075	11.85568919564751	9.57301953514711
H	7.15661624301470	11.13317257800299	4.08533158037628
C	3.71658735695937	10.69266563962917	3.86491004491251
H	2.95357738341426	10.85903454119513	3.08122041082504
H	3.70999252787533	9.61235861608063	4.10080036848941
C	3.37362025091319	11.50573902969995	5.11760976823035
H	2.37907400272959	11.20289830371837	5.49596938103922
C	4.42702707355311	11.24428654991132	6.19731943130218
H	4.43924645149626	10.17601075481968	6.47911157685199
H	4.18566132091976	11.81658095270050	7.11168177529333
N	4.16370241316646	13.55203503638241	9.65735687097980
N	2.94738177166024	13.13777523153475	9.24218366707800
C	2.40382203526631	14.04966959772793	8.40559993287436
C	3.30905493631955	15.09730650817631	8.29473686450775
H	3.17769093209780	16.00242646988668	7.70537387984430
C	4.39952002134036	14.74948365583621	9.11466906824765
C	1.06938476673198	13.88662330221649	7.76497187283447
H	0.25582930469052	13.86671141409801	8.51103110310547
H	0.88447418124920	14.72601245149122	7.07723318135543
H	1.00966466569259	12.95080238344637	7.18358440928307
C	5.62527549017948	15.57613804495027	9.43585516113994
C	5.17503923617022	17.01563462837554	9.72490829383688
H	4.45974218763930	17.04910766747384	10.56566116608026
H	6.04737143487216	17.64119816799830	9.98506972443756
H	4.68792335653190	17.47193237702955	8.84618149994493
C	6.33484108617357	15.02792463559925	10.67682734639577
H	6.78906247899091	14.04158293426297	10.46301375480531
H	7.16713644204327	15.69405652499000	10.96354475081178
H	5.64061704305165	14.95911317469339	11.53306118670306
C	6.59843402655262	15.57120399176518	8.24941775719199
H	6.10971031032453	15.94996458523765	7.33502811516827
H	7.46746628480077	16.21850456843455	8.46654081201758
H	6.96365866072231	14.55006259796339	8.05503139364911
H	4.88303429862773	9.81763590826857	14.28155418917587
C	6.05570367116377	12.10907171244399	13.29483158348513
H	6.04410350176532	13.03520670683019	12.68869729513973
H	6.96307706347062	12.14639113566075	13.92008756456075
H	6.16800139576699	11.21714819108023	12.64452027602363
C	0.06783674385176	11.94803618413042	12.10081693975106
H	-0.24038026572266	12.81412139653038	11.49016237078617
H	-0.23757215196660	11.03822432968822	11.55535059169337
H	-0.48864814567959	11.98127025240646	13.05012381824970

[(Tp^{tBu,Me})Ti(1,3-μ$_2$-NCNAd)$_2$Ti(Tp^{tBu,Me})] **(I(μ$_2$)) (S = 0, broken symmetry 1,1)**

Eh = -5351.442409506358

Ti	3.89460933465872	5.50648009932321	5.20345455420318
N	5.09026448743062	4.43151905548854	3.68215365510668
N	4.32094752147621	4.12744154114058	2.61363304651473
N	1.83713013378764	5.32093334869510	4.43275316507747

N	1.87265994138250	4.72047417675089	3.22227663034448
N	3.86616976712827	7.04072788148098	3.45917018039375
N	3.43217755074775	6.45115847347478	2.32111871817720
N	4.74270461319372	6.67237776440466	6.69289571043755
N	5.90018122019234	7.05517117962877	8.83344132449945
C	4.91616062663014	3.18033850089841	1.85427703046206
C	6.12780702343004	2.87566844372841	2.45671184031357
H	6.86712264303928	2.16158775584679	2.09856433607373
C	6.20482940343854	3.69394505058850	3.59870457406516
C	4.32939023130738	2.63068755902145	0.59992397395513
H	3.33039735468314	2.19488008321874	0.76988460147575
H	4.98364431221354	1.83847374475250	0.20367782147033
H	4.21985268752918	3.40726585150146	-0.17673919439980
C	7.36919166559020	3.79162360750981	4.55672786721204
C	7.67479593869975	2.40441787333271	5.13620154177046
H	6.80556673908607	2.01176500013683	5.69077505296337
H	8.53035231470268	2.46214772731882	5.83338046056184
H	7.92862824558783	1.67901681375307	4.34385300972515
C	7.06296514459332	4.76109734448869	5.68825703188085
H	6.85390424885363	5.77160333389185	5.30184560600705
H	7.92032352378742	4.83362540162041	6.37935066954969
H	6.19369300521941	4.42572175384331	6.27722588105016
C	8.59292688168921	4.30235543462479	3.78071292719229
H	8.85996270873259	3.62533942553705	2.95093993238871
H	9.46517065286807	4.38100096601882	4.45495731514242
H	8.39434133663521	5.30136871240033	3.35531545157294
C	0.66578194884261	4.18810701845312	2.92611305222630
C	-0.18504744609847	4.48753856278495	3.98257078889983
H	-1.23617148774938	4.22017858728024	4.05951306089798
C	0.58730478604815	5.22202535677785	4.90115061722380
C	0.37815031170886	3.44139381358945	1.67064362126316
H	0.46113574833585	4.08855360209825	0.78025165639653
H	-0.64527890830207	3.03692346737298	1.70670561008889
H	1.07478181069478	2.59766690063429	1.53034915466174
C	0.11039570165062	5.97255891776177	6.12355797387072
C	-1.22600594751060	5.40052073516663	6.60359433006383
H	-2.01462066044661	5.50459874930608	5.83860929598979
H	-1.56291500823455	5.94031611241513	7.50565927175884
H	-1.13218323317421	4.33091641177996	6.86226734900552
C	-0.07790132531098	7.44216371581371	5.70689668032036
H	0.87786104580471	7.87256473433040	5.36234629726784
H	-0.44241437968914	8.04056999923995	6.56219600533618
H	-0.80873187170123	7.53073942297139	4.88419310241077
C	1.11467488050823	5.90575858360650	7.26834675195412
H	1.28510494129795	4.87385635540597	7.60277020249439
H	0.73897589833498	6.48248610417752	8.13023763078511
H	2.08892935207496	6.34695081612338	6.99259307799780
C	3.35615533835229	7.34276294999282	1.30835095719756
C	3.76079552644982	8.56609874935302	1.81676250779855
H	3.82162069531379	9.50386063319104	1.27061206677428
C	4.07244863414634	8.33254617643603	3.16969102173194
C	2.91268545913795	7.00018377759861	-0.07195853698942
H	3.55367517294068	6.22684908593889	-0.52979405497746
H	2.95607542344975	7.89940775747290	-0.70625280494968
H	1.87639540712098	6.62011817095418	-0.08909785377251

C	4.58931167092501	9.34646058822777	4.16523929172352
C	4.69000085849463	10.72454184200392	3.50450219434493
H	5.06801993785789	11.45903133549580	4.23736942781135
H	3.70641079631847	11.07820577756199	3.14925538784292
H	5.38564672471378	10.71487317565100	2.64743542860417
C	5.99304798008372	8.92509086394294	4.61630372473622
H	6.39384452048736	9.64326910479747	5.35384494383337
H	6.68171475739055	8.89681020456203	3.75338959128543
H	5.97316655099664	7.92798389025110	5.08040520709804
C	3.62709153127576	9.44911769500722	5.35559923244898
H	3.51465407997227	8.47942356012422	5.86225738005590
H	2.63259061175691	9.79065753436336	5.01790199428376
H	4.00705964230817	10.17652141931630	6.09624646719383
C	5.32712876137646	6.89162892636896	7.70230591987564
B	3.07079051116944	4.96362809128648	2.27146525523500
H	2.72004611599142	4.66201111133222	1.15227418264500
N	3.91552843959515	4.74648648431777	8.92806936818720
N	3.64699907235030	3.83359211420839	6.65524349385515
C	3.77296151929734	4.26611796066656	7.85237391985148
Ti	4.85360758853411	5.86708167340787	10.39931237860089
N	4.28444421876723	7.65591661728414	11.56754963394823
N	5.08468089512127	7.74215684258474	12.65353383515247
N	6.65073118777731	5.12403480107835	11.46944779366384
N	6.80875826507433	5.80899997894678	12.62386553327826
N	3.86271841384727	4.96243706253584	12.28561672663912
N	4.45117886996343	5.44731813789261	13.40379063563592
C	5.09723548807833	9.00750776159281	13.12879970799060
C	4.24321027706721	9.75509710125108	12.32800928353039
H	4.01145196398869	10.81118806535990	12.44401983074152
C	3.73509891972409	8.85995980502343	11.36847563880303
C	5.90288921675266	9.44329264454272	14.30263668920388
H	6.97503392756654	9.22288134961775	14.16497166439452
H	5.79479132289483	10.53015427846508	14.44185057546187
H	5.58127125079326	8.94229990521769	15.23206290936499
C	2.60990461152553	9.09410879166734	10.38575376134157
C	2.81915581318744	8.33868219304284	9.07892819869334
H	2.90405191182535	7.24846224187617	9.23722104373219
H	1.95347350550867	8.49459839196986	8.41358975036094
H	3.71623564921272	8.68145407378604	8.54599942699260
C	1.31668424101830	8.58810754909586	11.04831675147378
H	1.12222340184204	9.11612983112977	11.99792768133038
H	0.45372919090816	8.75012926720560	10.37668906585420
H	1.39240437079112	7.50892363474147	11.26586259755157
C	2.47842129584642	10.58745199460894	10.07584974774296
H	3.40972590721900	10.98804712390276	9.63841329350950
H	1.66454699603594	10.74949658867053	9.34780936380860
H	2.23944946660258	11.17508766591334	10.97889805809524
C	8.07098702789432	5.67241613248011	13.08832976540105
C	8.74545014144721	4.84601398476823	12.20262665762548
H	9.77965616084279	4.51667772580628	12.28394850656722
C	7.81315832500619	4.51411451197841	11.20289830525092
C	8.57324893338454	6.31063500170742	14.33633452981948
H	8.05980278178332	5.92158095564274	15.23266658935434
H	9.65061342380874	6.10903909167457	14.44342997008827
H	8.42916827404507	7.40384489571047	14.32405613083846

C	8.04269074392847	3.56900879425431	10.04762125190267
C	9.29130204922941	4.00195126658124	9.26873630488113
H	10.19180360419692	3.99614968792844	9.90708668773767
H	9.47012197171431	3.31361057734394	8.42313580090908
H	9.16716173051105	5.02048500809177	8.86291784373246
C	8.26323913679283	2.15902402178325	10.61840859046384
H	7.37829379048853	1.82668292547490	11.18835234575415
H	8.43746557519794	1.43854328271944	9.79859270877506
H	9.13511939662179	2.12876196767996	11.29479144200837
C	6.84200962830410	3.54696741147868	9.11451814100523
H	6.64436780010272	4.54736728960115	8.69701357384474
H	7.02420438475539	2.86321557321780	8.26805070281551
H	5.93512906839975	3.20416761840761	9.63816541817999
C	3.83916596967325	4.98452698357491	14.51640768515753
C	2.80664070438463	4.16206754360519	14.09592442511712
H	2.11235289396173	3.62159443765405	14.73404407681579
C	2.85428613573041	4.17690389359106	12.68827046314058
C	4.25538862378067	5.33740857934685	15.90291504860804
H	4.17546494318736	6.42168759875429	16.09444425374137
H	3.60947080795354	4.81418960949611	16.62538986517419
H	5.30067639497532	5.04551529592052	16.10461217401107
C	1.94527246192062	3.41717805827785	11.74551884994669
C	0.87572877442190	2.66666892414403	12.54441266999014
H	0.21196530194493	2.11966645064476	11.85186782016425
H	1.32361694370613	1.92977045428032	13.23357607752279
H	0.25072369913592	3.35889246663983	13.13522532467742
C	1.23863580276750	4.38892813430155	10.79147728957309
H	0.58939155406898	3.83364144749518	10.08954987795083
H	0.60618681520036	5.09416288952368	11.35959279344088
H	1.96317704059931	4.96351769681804	10.19584993303630
C	2.77978536496041	2.38900241606533	10.97115931504336
H	3.56865146742543	2.88555328371058	10.38624702640897
H	3.25471197687470	1.67606077517884	11.66820932810909
H	2.13936959836854	1.81661327425865	10.27575798057813
B	5.60472883008924	6.45305006067115	13.33942791119632
H	5.95761059636819	6.74936582907705	14.45959509589535
C	6.94545396097488	8.09249347203479	8.93976950344103
C	6.34569061269765	9.49778477588445	8.77880439618230
C	8.02435181163523	7.89260391335575	7.86555262778020
C	7.60946080961969	8.00531372463527	10.31389284983304
H	5.57512132629148	9.64641253444232	9.55474791254979
C	7.43439789915946	10.56684440746152	8.90801030860078
H	5.84847800326322	9.57149244852853	7.79379988015539
C	9.11877342953234	8.95554987935077	7.99750019659963
H	7.56050822404671	7.94179110248176	6.86442304388527
H	8.45273430434256	6.88012056038442	7.97450343483235
C	8.69730354517328	9.07119517319178	10.45761034207776
H	6.84478646950203	8.14257949792594	11.09464612097453
H	8.04642826542534	7.00180602204188	10.44785232491493
H	6.97928297765131	11.56591765447787	8.78340070538538
C	8.49992420748913	10.34717616945587	7.82966136738295
C	8.07697200898189	10.46170737613723	10.29448910972271
H	9.88138157812513	8.79079937219096	7.21560367728369
C	9.76554250361127	8.85090542359131	9.38243827882226
H	9.15474037884020	8.98073112974710	11.45944399036528

```
H     9.28429142892765   11.12309539201420    7.90524390624394
H     8.04792196124966   10.44138301697013    6.82527267458099
H     8.85019564496639   11.24314881348533   10.41682254194822
H     7.31325616642580   10.62802620579845   11.07712715235009
H    10.57268782018552    9.60012271954285    9.48361925133634
H    10.22846356289411    7.85483019532179    9.51153668451369
C     3.19682506836866    2.43901689809325    6.47070348150895
C     4.08524192210252    1.47271284193946    7.26755784188442
C     3.28171267408781    2.06664113630749    4.99026471071109
C     1.74136155501068    2.26131303348737    6.93135103062081
H     4.04329990732623    1.73186142569746    8.34075575219920
C     3.63538777015974    0.02489473828914    7.05574394244627
H     5.13305166000244    1.60449420591322    6.94360217930583
C     2.82298804606707    0.62302274553463    4.77004566405268
H     4.32004617657446    2.18954924490305    4.63933458629656
H     2.64935668815445    2.75449705652643    4.40510456828818
C     1.28411966491426    0.81379523357700    6.72730618447377
H     1.65799331851786    2.54335248619239    7.99738254904656
H     1.09783058190990    2.94635327802528    6.35239295099793
H     4.28997961400213   -0.64835993484354    7.63730515921057
C     3.72670561471679   -0.32239328424618    5.56678259369724
C     2.18651232744446   -0.12913416007662    7.52886190568988
H     2.89585547222903    0.38723839107810    3.69306128306431
C     1.37300738703557    0.46804272415694    5.23764137373465
H     0.23951642196619    0.71143310517718    7.07234215872958
H     3.42420814387160   -1.37322314469679    5.40153919338236
H     4.77188419039354   -0.22697047891305    5.21848456285947
H     1.85347347147089   -1.17582156210856    7.40025591363641
H     2.11490423945452    0.10250758876171    8.60733292250801
H     1.02321730216468   -0.56671342971226    5.06305225349898
H     0.71510490894029    1.14036482877730    4.65586386741310
```

[(Tp^{tBu,Me})Ti(1,3-μ$_2$-NCNAd)$_2$Ti(Tp^{tBu,Me})] **(I(μ$_2$)) (S = 0, broken symmetry 2,2)**

```
Eh = -5351.471918634552
Ti    3.88369532277352    5.49855820111507    5.18792824879986
N     5.10599020781413    4.43291614995550    3.66572532326567
N     4.35172764528661    4.12934225254007    2.58675472621346
N     1.83639593800100    5.27355316035536    4.38439463666136
N     1.89061368587560    4.69165722687281    3.16613903794168
N     3.84339824463145    7.04429973944693    3.44210843995847
N     3.43977221501196    6.44917978827255    2.29592962469388
N     4.73334979613012    6.69312140740008    6.68538084695126
N     5.91444081680459    7.03987850346323    8.81631198088854
C     4.96134179546707    3.18848426487220    1.83070443113656
C     6.16582509863085    2.88596136254672    2.44801989972500
H     6.91293713061901    2.17660058887541    2.09668773516326
C     6.22376081314390    3.70005149221371    3.59440429787995
C     4.39439041178750    2.64222215410702    0.56570953741862
H     3.39636424425615    2.19841863689208    0.71996628261855
H     5.05917289609002    1.85677868714599    0.17351651297363
H     4.28966537987397    3.42247550739701   -0.20788631650139
C     7.37409216546089    3.79756123684444    4.56953737161931
C     7.66449445236972    2.41249726850011    5.16208336242084
H     6.78685551496153    2.02785594858254    5.70888524877694
```

H	8.51197412741019	2.46981049550401	5.86918746430897
H	7.92359149773368	1.68068948187296	4.37730653846772
C	7.05511346983543	4.77566140608020	5.69016348903857
H	6.85157313143031	5.78368244454282	5.29413902811411
H	7.90374070131835	4.85159606598154	6.39150356549724
H	6.17877934609428	4.44432338673560	6.27076161909611
C	8.61217431501414	4.29760016996209	3.80966366002286
H	8.88968744908924	3.61390857369178	2.98889890941192
H	9.47391006330314	4.37717479306629	4.49715530067059
H	8.42488726191329	5.29449265924028	3.37431531415586
C	0.69127630468945	4.15451782157428	2.84835249934989
C	-0.17285731449530	4.42820973729504	3.90086860805954
H	-1.22251209710655	4.15075312036199	3.96214928709166
C	0.58456323513937	5.15389186737239	4.83925722852632
C	0.42209086102615	3.42810881512334	1.57681387602105
H	0.50759347922696	4.09240818641496	0.69936528023470
H	-0.59805753911708	3.01426809643854	1.59562470235148
H	1.12736524752616	2.59326610367682	1.42738750946975
C	0.08764650590797	5.88330973555172	6.06617591562333
C	-1.18984511010869	5.22235123049859	6.59107277971998
H	-2.00339300965187	5.25300768572441	5.84599300675069
H	-1.54371131389361	5.75099487318901	7.49334647473767
H	-1.00919794559507	4.16745778797069	6.86330755344290
C	-0.21620347996546	7.32920004080189	5.63459338185543
H	0.69761018669448	7.81785100855733	5.25482854916385
H	-0.59520571858395	7.91547100793157	6.49185968992999
H	-0.97518157375676	7.35347437081520	4.83327364345300
C	1.12333846103582	5.91019798782677	7.18433478520773
H	1.37016732498095	4.90149615940193	7.54006339274151
H	0.72889203982947	6.48084384177074	8.04123842138729
H	2.05943134758339	6.41153919042245	6.87732449383059
C	3.35480563634496	7.34451058728291	1.28649717834903
C	3.72096976333581	8.57496943044658	1.80628300210948
H	3.76505394824958	9.51687083890414	1.26566729551294
C	4.01987279157963	8.34225192373753	3.16245919046905
C	2.93845612196336	6.99915307253871	-0.10160623645801
H	3.60255371711732	6.24234479656848	-0.55402141711384
H	2.97135451267915	7.90242597125944	-0.73075915092387
H	1.91097341247511	6.59688314191903	-0.13569598598166
C	4.49540572853682	9.36418661686717	4.17125855667791
C	4.55314657024296	10.75122711601724	3.52421740141790
H	4.89988102552973	11.49131240330705	4.26685001240681
H	3.56044634846924	11.07358841426858	3.16456143855417
H	5.25514991065865	10.77429884819237	2.67261035634149
C	5.91001354507590	8.98705180317105	4.62809003065015
H	6.27643608513685	9.70411368176350	5.38458018411762
H	6.60664922343703	9.00420532128912	3.77130745181873
H	5.92578211525953	7.98006972245407	5.07050246822517
C	3.52196490321353	9.42451702840239	5.35550754011357
H	3.43682825926322	8.44760817379976	5.85339791205922
H	2.51945474398307	9.73779705466611	5.01396176766765
H	3.87394136502366	10.15569120268698	6.10637803456478
C	5.32840049786662	6.89289787812103	7.69091834954349
B	3.09867218651028	4.95614253061375	2.23330101253262
H	2.76597723416362	4.65856478907898	1.10779965652817

N	3.90225341075084	4.72968254424704	8.94087309645218
N	3.66198304476742	3.81919808832069	6.66674990897171
C	3.77640608021203	4.25163839922389	7.86365013196430
Ti	4.86030171454269	5.87041568340352	10.41612043871757
N	4.32634953789909	7.66897434640626	11.60931193369783
N	5.13632655462418	7.73091775434952	12.68962210025607
N	6.66306693559610	5.10919948292145	11.48522502678351
N	6.82255940588339	5.76815501927558	12.65391751604795
N	3.85535326199832	4.97239715703225	12.30608704067739
N	4.45193058161595	5.44407967247396	13.42546301839571
C	5.18634660328114	8.99439001094320	13.16795546367270
C	4.34858856637764	9.76728172168247	12.37453049974278
H	4.14645312293003	10.82926017794836	12.49482180865319
C	3.81130208482604	8.88794242707663	11.41571783062514
C	6.01314805535394	9.40532857667342	14.33613967520414
H	7.07881681299883	9.16292873860114	14.18518479402261
H	5.93007837831227	10.49331071722719	14.48338959705313
H	5.69089934413294	8.90505306900814	15.26565137498894
C	2.68540770239623	9.15640558403522	10.44324507456934
C	2.77221729749054	8.26859680801521	9.20742518949640
H	2.75735692522361	7.19411683159477	9.46773441647054
H	1.90010975769975	8.44737630633935	8.55651193834284
H	3.67537956309048	8.47584793622717	8.61746552743302
C	1.37165952629643	8.85103235621971	11.18445215181166
H	1.26976666473178	9.47787844609324	12.08713395048461
H	0.50624329837060	9.04645980635867	10.52492419957709
H	1.34190382505585	7.79263916329741	11.49663800671351
C	2.69385139286000	10.62340371918160	10.00363309970631
H	3.63760728736693	10.88467681774748	9.49453648020211
H	1.86561910603002	10.80851942488786	9.29743480409807
H	2.56125314360285	11.30821628111824	10.85890573700967
C	8.07862111135830	5.60170699435149	13.12614354934137
C	8.74817328828993	4.78607942195515	12.22728696241357
H	9.77682975156451	4.43984021564254	12.30892866176174
C	7.81827887265604	4.48917969933625	11.21396906007037
C	8.57845158089039	6.19865113258627	14.39563510849330
H	8.04937681230052	5.79406708428292	15.27585330915330
H	9.65107857674703	5.97571063434687	14.50798709955255
H	8.45298793961474	7.29401119100304	14.41262738575931
C	8.04555204238791	3.56791595993079	10.03891138433502
C	9.27851489717037	4.03189735710645	9.25242952725628
H	10.18531117210120	4.03312503425174	9.88188767333144
H	9.45979177688844	3.35743576095433	8.39617229699775
H	9.13334119889499	5.05309752086725	8.86076757982079
C	8.29280730832696	2.15092427552547	10.58054441719096
H	7.41986226951359	1.79534405786099	11.15496956922699
H	8.46679340342415	1.44849806291760	9.74519063109674
H	9.17341281818748	2.11958616235617	11.24544573134832
C	6.83342914248137	3.54590877373179	9.12065272311769
H	6.62292306158055	4.54921031030179	8.71653766068207
H	7.01096636496423	2.87372075808930	8.26405026642915
H	5.93604323619395	3.18947730040599	9.65155396588627
C	3.82048191129254	5.00189176721111	14.53620842282641
C	2.76760253723808	4.20776406076487	14.11255357343412
H	2.05514197832157	3.68887256572015	14.74854549894613

C	2.82301265212880	4.21754780490994	12.70485316400109
C	4.23666543850652	5.34763108223099	15.92461860297987
H	4.18251026368300	6.43399515594952	16.11333059910364
H	3.57283937599921	4.84251573848560	16.64374076204830
H	5.27276590817105	5.02994452006933	16.13435156759548
C	1.89622795956127	3.48273737848523	11.75895569995393
C	0.80257222411300	2.76528459993995	12.55545252364343
H	0.12681027161800	2.23458499637126	11.86191148167541
H	1.22731491987228	2.01884269242211	13.24895142381798
H	0.19476191984351	3.47676066453613	13.14130987839239
C	1.22000587466564	4.47190470145002	10.80027233483836
H	0.55093303463714	3.93374259669028	10.10339935403444
H	0.61375599954989	5.20269256514282	11.36497815845473
H	1.96060218558792	5.01693681371795	10.19692655680311
C	2.70299256581639	2.42840719630789	10.98984780432512
H	3.51278300632920	2.89834155616461	10.41189623013248
H	3.14830325230709	1.69946416002590	11.68984894994982
H	2.05015617795551	1.87812524819443	10.28793199588613
B	5.62707888100272	6.42626005839387	13.36939901021251
H	5.98056617235726	6.70795583334387	14.49288859063997
C	6.95174690646287	8.08251211971428	8.92656689994763
C	6.32969721032518	9.48276445112259	8.81591862222597
C	8.01013705328602	7.92326989977777	7.82587764249489
C	7.64455494233476	7.96968919958120	10.28518576902109
H	5.57875291545393	9.59636220988999	9.61584684826453
C	7.40587935496760	10.56359561894898	8.94774994699604
H	5.80446200346473	9.57502316978678	7.84684692045801
C	9.09184459976292	8.99858114183080	7.96141234521789
H	7.52455245092891	7.99047953692973	6.83617041054386
H	8.45468816516515	6.91462674314214	7.90043797226917
C	8.72139119794818	9.04665587747811	10.43214803312035
H	6.89471520303417	8.07950585677535	11.08519041749416
H	8.09554504357342	6.96848458608222	10.38763787232852
H	6.93493039524991	11.55911932854377	8.85825789761726
C	8.45010893292205	10.38493636827130	7.84138559826503
C	8.07991847850285	10.43248927432095	10.31698882554439
H	9.83982498750846	8.86445234645455	7.15976236652037
C	9.76928389247032	8.86879503344342	9.32945956768016
H	9.20120312350122	8.93761723692874	11.42145695777850
H	9.22526812066777	11.16985043788081	7.91887014071071
H	7.97467703768927	10.49680139618824	6.84962347300100
H	8.84600956159667	11.22045727941748	10.44204957900131
H	7.33136379019967	10.56945167938672	11.11967960657383
H	10.56731585436235	9.62737550342514	9.43283801723426
H	10.24957509214862	7.87701448375241	9.42342695203575
C	3.20904247700551	2.42750842432182	6.47769643510903
C	4.07362895936331	1.45808507836909	7.29660974950546
C	3.32242520598553	2.05163366201106	4.99952161088787
C	1.74214174179346	2.25990852959413	6.90584440103497
H	4.00964838859404	1.72082567183303	8.36801082716718
C	3.61909410850061	0.01273294095529	7.07911876204202
H	5.12921362955165	1.58229788514063	6.99599813189427
C	2.86072991292814	0.60976221715032	4.77376738613809
H	4.36784878045000	2.17068437854331	4.66852128241160
H	2.70478599761583	2.74111271978952	4.39992476387715

C	1.28086274763373	0.81459714687164	6.69705744559524
H	1.63428552058100	2.54809919957526	7.96826938682375
H	1.11695918994551	2.94494565621645	6.30754724904074
H	4.25668702314313	-0.66304102405702	7.67640408673050
C	3.74054088566600	-0.33892153854045	5.59318026715316
C	2.15963739826206	-0.13023004459226	7.52200220424387
H	2.95553320441989	0.37014523129766	3.69939602206196
C	1.40019765313326	0.46403585902708	5.21059902227392
H	0.22816708127493	0.71995740738874	7.01902357194058
H	3.43421228132981	-1.38803132252656	5.42403084527921
H	4.79385958360528	-0.25213402774638	5.26789568411886
H	1.82270296961573	-1.17542627373483	7.39168662800687
H	2.06684957631705	0.10770990464681	8.59764714884597
H	1.04880607196681	-0.56941230211605	5.03150320294686
H	0.75840949976352	1.13809123909091	4.61309837820034

[(Tp^{tBu,Me})Ti(1,3-μ$_2$-NCNAd)$_2$Ti(Tp^{tBu,Me})] **(I(μ$_2$)) (S = 2)**

Eh = -5351.471139419604

Ti	3.88324912408272	5.49893272552009	5.17184506396514
N	5.10240693012561	4.43402592924356	3.64937356704381
N	4.34978577777005	4.13341034492914	2.56870556504967
N	1.83505262152085	5.27239150930367	4.37362324805063
N	1.88955424538558	4.69046828780514	3.15525954778927
N	3.84048254002583	7.04760232309251	3.43084862482137
N	3.43397946329527	6.45332410565187	2.28504129170788
N	4.72574047247413	6.69718791373042	6.67582483928050
N	5.90927282019175	7.02504038366884	8.80754795422303
C	4.96161072826215	3.19629641220186	1.80973617253023
C	6.16577716074293	2.89302612019792	2.42745724756621
H	6.91420499000002	2.18587136330485	2.07449047471401
C	6.22118893879827	3.70284699110011	3.57704977492509
C	4.39660318401111	2.65449560311381	0.54197184088953
H	3.39963380731697	2.20746073785087	0.69382202989160
H	5.06349772129704	1.87259557486703	0.14630182561263
H	4.29012901607491	3.43812984046987	-0.22795449693752
C	7.36926387637539	3.79857478072697	4.55503732758090
C	7.66448222046658	2.41109044945867	5.13947172295376
H	6.78846739283678	2.02028217560353	5.68461630732515
H	8.51189803954448	2.46747025426544	5.84673419426104
H	7.92598104908106	1.68450072831442	4.35068065946529
C	7.04378742627308	4.76783600260487	5.68165915522438
H	6.83703253285078	5.77761252740305	5.29168717342081
H	7.89076260825083	4.84337365167330	6.38508399294718
H	6.16769736592951	4.42887830571448	6.25859983579245
C	8.60664067450007	4.30901654924340	3.80083145373693
H	8.88801764568249	3.63264083426205	2.97529680961982
H	9.46712462783459	4.38675600967267	4.49009143227606
H	8.41613115886932	5.30845110714728	3.37275991912144
C	0.69173660358688	4.14874796693171	2.83959004089412
C	-0.17179747087770	4.41946498974182	3.89345527312160
H	-1.22041216607599	4.13840062589038	3.95612966407302
C	0.58439349274720	5.14823580271091	4.83048984684411
C	0.42310549941225	3.42137795923658	1.56846985070983
H	0.50406023678497	4.08628181463257	0.69103962486373

H	-0.59519738296670	3.00310118775684	1.58919370321414
H	1.13170240650563	2.58965948823440	1.41732494345049
C	0.08551795016304	5.87849636028278	6.05632010086716
C	-1.18795295730311	5.21262286117200	6.58465505603927
H	-2.00258803699828	5.23684507162073	5.84053422486145
H	-1.54345870472930	5.74190686919536	7.48590471859480
H	-1.00119969743880	4.15951082170703	6.85961306109108
C	-0.22574124105907	7.32141581806828	5.61982125073057
H	0.68580937651293	7.81269064346393	5.23792603831003
H	-0.60676094268495	7.90893988813563	6.47533013218334
H	-0.98521125053708	7.33953440779323	4.81882063682692
C	1.12165276833266	5.91550630693574	7.17402258913886
H	1.37194242018016	4.90984126524851	7.53610216423567
H	0.72461994796934	6.48980682299576	8.02733062142446
H	2.05600461728089	6.41824089317324	6.86434907935897
C	3.34489191730654	7.35006807381578	1.27707952533777
C	3.71066554631529	8.58034470713217	1.79751225245182
H	3.75167504551473	9.52305885294781	1.25805142562039
C	4.01350652732209	8.34625601444976	3.15257767172784
C	2.92519510901976	7.00623195430665	-0.11039639739510
H	3.58980784874157	6.25190269353111	-0.56620947003356
H	2.95410363320414	7.91075648598994	-0.73794568650739
H	1.89861481453212	6.60150373400240	-0.14230161124252
C	4.48843500066961	9.36778190071663	4.16212359376417
C	4.54536414239977	10.75530487142363	3.51608817010885
H	4.89256617457178	11.49490101675942	4.25899374019438
H	3.55224963834412	11.07775057351878	3.15765832772546
H	5.24651613094323	10.77919522811881	2.66379879729721
C	5.90326830038441	8.99120426131572	4.61876342236092
H	6.26872529858191	9.70702418809117	5.37689618390833
H	6.60015479290658	9.01080204451304	3.76223200873342
H	5.92015972247777	7.98338221445456	5.05915216518608
C	3.51472244081337	9.42659996142603	5.34631918186008
H	3.43062168884571	8.44941561833910	5.84385091129459
H	2.51189629364219	9.73881640409773	5.00465586403616
H	3.86559704058603	10.15799959972705	6.09751661581728
C	5.32011928757843	6.88935516800676	7.68251638253389
B	3.09507376656604	4.95960889302476	2.22006624082328
H	2.76015800903239	4.66425580327471	1.09457919258548
N	3.89598296447452	4.73353347654429	8.95165664939615
N	3.66874686596706	3.83074339043907	6.67400824748767
C	3.77655537711682	4.25967382139930	7.87255553421033
Ti	4.86274417618623	5.86880283648614	10.43123523349189
N	4.33155129073360	7.67131324619836	11.61743337302899
N	5.14128572710597	7.73331297693328	12.69801638359653
N	6.66215995480267	5.10755691657760	11.50250388425516
N	6.82093215198167	5.76507525734586	12.67187050561940
N	3.85241108011573	4.97524576206842	12.31834739031286
N	4.44701492595328	5.44957907819172	13.43777106380322
C	5.19485475731105	8.99777674167095	13.17331165665472
C	4.35949686586442	9.77123833296487	12.37784239664190
H	4.16044672707058	10.83407162173263	12.49572698743653
C	3.81944891664108	8.89108292095121	11.42118822610754
C	6.02206446419478	9.40897992247805	14.34111441418457
H	7.08702926025046	9.16246594534808	14.19184399039206

H	5.94256289500495	10.49761566322289	14.48549443487692
H	5.69715837937310	8.91220064694827	15.27159247414678
C	2.69224949649473	9.16057014390955	10.45032172471036
C	2.76988279769602	8.26837361472288	9.21684265762000
H	2.75302496630575	7.19473652261492	9.48001568969616
H	1.89492169920134	8.44859776346161	8.57015257826834
H	3.67037639535109	8.47035010472727	8.62090610051547
C	1.37971606724035	8.86264999682370	11.19681198660205
H	1.28311937125759	9.49172777128930	12.09854013440897
H	0.51295213690949	9.06013999118385	10.53967101385786
H	1.34652822973646	7.80509195629813	11.51154462167228
C	2.70500299392992	10.62615730593395	10.00608217588236
H	3.64841227309469	10.88234023397680	9.49377878986843
H	1.87574942548580	10.81192103795718	9.30126208075034
H	2.57688754028191	11.31420169555478	10.85943074081839
C	8.07558823792814	5.59492796828738	13.14662662369551
C	8.74493360673140	4.77855903207924	12.24820660814585
H	9.77257427613905	4.42973004091640	12.33161902830416
C	7.81645062549558	4.48530129833877	11.23242162479261
C	8.57385908298011	6.18913983014985	14.41802882754637
H	8.04018624444605	5.78632697545839	15.29626931197892
H	9.64516622063369	5.96164223187535	14.53377267683154
H	8.45292759105764	7.28503022870212	14.43530844009761
C	8.04390344918176	3.56693614002624	10.05502346507412
C	9.28107846344143	4.02866685230252	9.27377799576177
H	10.18560770970808	4.02673822874978	9.90648306828131
H	9.46370451895912	3.35483829845982	8.41728813313378
H	9.14010525336124	5.05064510198109	8.88261671450074
C	8.28392684586454	2.14726932100399	10.59284014136119
H	7.40777415060399	1.79327985226659	11.16336578238748
H	8.45792213353297	1.44674643710837	9.75589246424745
H	9.16222833430338	2.11045165797730	11.26051338803692
C	6.83488311537621	3.55284299597999	9.13223532021913
H	6.62986000079997	4.55808886923504	8.72968480053453
H	7.01264329544587	2.88192380186981	8.27457068954740
H	5.93415815250350	3.19835922351961	9.65892131856394
C	3.81316399271228	5.00999895556058	14.54827106061405
C	2.76103773954420	4.21493122697939	14.12436328980715
H	2.04723385771479	3.69766567083369	14.76019243174834
C	2.81947813643672	4.22115536203379	12.71677371075356
C	4.22657101487380	5.35852525570388	15.93682338252836
H	4.17387112035509	6.44544468197996	16.12267157470989
H	3.56001026079857	4.85641443845047	16.65552838114955
H	5.26154687182242	5.03947285112418	16.15007289442667
C	1.89521033115657	3.48410286421238	11.77006315811503
C	0.80299646531982	2.76354778550082	12.56572743578363
H	0.12953156467523	2.23051969690477	11.87173104154726
H	1.22949605186177	2.01874696245873	13.25992671255113
H	0.19235101453492	3.47328847314312	13.15073106301699
C	1.21697061856381	4.47213314625705	10.81146082803909
H	0.54894774911532	3.93289977100704	10.11436896888374
H	0.60940510911834	5.20159313286856	11.37647879479843
H	1.95639815248411	5.01897979246368	10.20830554607591
C	2.70494292893326	2.43203081260770	11.00093796586915
H	3.51457295828284	2.90373812924922	10.42422200785451

H	3.15076183714025	1.70331211922290	11.70085444310522
H	2.05396105812494	1.88117233923019	10.29773419924766
B	5.62544761994445	6.42824463873788	13.38249280466127
H	5.97653386211353	6.71152908286615	14.50639426935398
C	6.94860313715959	8.06629981340649	8.91918766784515
C	6.32889949666610	9.46775571671847	8.80929810360638
C	8.00754345205086	7.90662510202837	7.81896106298588
C	7.64045954699109	7.95212455227900	10.27824118177573
H	5.57765130153025	9.58199071326185	9.60881601974445
C	7.40635451198884	10.54708592304515	8.94250610781857
H	5.80436639136519	9.56168406760317	7.84001018590856
C	9.09061463484738	8.98046492696606	7.95600867548056
H	7.52266165203559	7.97574009370372	6.82901080148647
H	8.45081925593255	6.89733901594404	7.89250062643430
C	8.71876577705895	9.02747866187566	10.42650088407696
H	6.89053384417225	8.06323047504794	11.07814176190366
H	8.09010618036409	6.95048121506165	10.38131089932992
H	6.93648239374404	11.54314673102950	8.85335152648561
C	8.45092589578529	10.36781935286994	7.83659282372934
C	8.07934037794138	10.41427233346346	10.31204902870265
H	9.83887244067812	8.84584945861208	7.15470505002851
C	9.76706155959755	8.84887030995377	9.32434790107283
H	9.19764504705335	8.91672860515058	11.41603848611711
H	9.22719808087872	11.15151938335078	7.91512447965335
H	7.97621531189801	10.48111188824529	6.84465587198585
H	8.84643966362444	11.20108336753879	10.43811407302011
H	7.33045734895576	10.55162772822396	11.11436987997071
H	10.56602759685263	9.60631855280459	9.42871719597138
H	10.24605827025475	7.85643776676089	9.41796670427289
C	3.21927715359294	2.43751678537084	6.48425090221591
C	4.08510980394785	1.46969727098286	7.30383282377336
C	3.33479868711905	2.06214692589298	5.00603435938627
C	1.75223904063658	2.26615472647226	6.91058608496501
H	4.01900414098731	1.73173891689569	8.37530346040795
C	3.63429893168376	0.02334626956346	7.08533412077923
H	5.14080632472117	1.59637353555970	7.00470759114316
C	2.87697016999724	0.61922113547278	4.77934720989227
H	4.38003477359025	2.18402409421341	4.67574054196111
H	2.71582430289084	2.75009165766962	4.40589099682485
C	1.29467502816456	0.81976295144571	6.70097460927720
H	1.64216263157870	2.55404141195835	7.97288692287564
H	1.12605696592488	2.94960091437919	6.31157449477673
H	4.27293634309630	-0.65102376109873	7.68309133309579
C	3.75824388543441	-0.32751524708663	5.59943343271841
C	2.17472694597600	-0.12317352324339	7.52668371552983
H	2.97360337934041	0.38033088245646	3.70500593880759
C	1.41635810043099	0.46981381914169	5.21457977363772
H	0.24185459539154	0.72265948898718	7.02179195677721
H	3.45466809158596	-1.37730277814367	5.42958195605004
H	4.81170820712655	-0.23816462036843	5.27530094452179
H	1.84036364272976	-1.16912842038104	7.39586149607507
H	2.08028855056053	0.11440649363467	8.60226831512767
H	1.06772414212599	-0.56447129662667	5.03493470081058
H	0.77360239570998	1.14242667021361	4.61651910729259

[(Tp^{tBu,Me})Ti(1,3-μ$_2$-NCNAd)$_2$Ti(Tp^{tBu,Me})] **(I(μ$_2$)) (S = 0)**

```
Eh = -5351.38701795785
Ti    3.87870995288299    5.49345999779070    5.25548006080387
N     5.09438972783843    4.41838388319143    3.78068266379676
N     4.32428355894611    4.13422692599812    2.70587998470810
N     1.83379416241236    5.38856176206261    4.55300365029191
N     1.88529388652506    4.74979284639693    3.35943895277701
N     3.89254695031766    7.04489087973824    3.54519964508355
N     3.43488002865722    6.45876008342313    2.41437728175452
N     4.68262769507465    6.62744355071767    6.71679207462140
N     5.86054421951985    7.06356470359526    8.85151511213416
C     4.94115847875994    3.23767120624467    1.90867147639577
C     6.16746893391062    2.94470630673807    2.49062055113103
H     6.92441959138998    2.26759341693849    2.09898595303243
C     6.23147530697926    3.71996277765766    3.66076578880836
C     4.36435002728492    2.72056853461529    0.63596887007052
H     3.37108910746611    2.26764508796568    0.79183401969535
H     5.03005310282066    1.95051745740733    0.21582537609273
H     4.24580616917943    3.52059307167077   -0.11481469757736
C     7.39900600847442    3.80847976174576    4.61453659945248
C     7.68110203411504    2.41947549341099    5.20215141736870
H     6.80641506333417    2.04663483024459    5.76084036109546
H     8.53964438753741    2.46611361996536    5.89628613890980
H     7.91883125584630    1.68577794262668    4.41230594929498
C     7.11299325942021    4.79560010254653    5.73568407763463
H     6.92747447458139    5.80674800021553    5.33925836434057
H     7.97164513081081    4.85135671627817    6.42600323127742
H     6.23349929691523    4.48828861840380    6.32332541502091
C     8.62894571624885    4.28977680317969    3.83002198129559
H     8.88898831592801    3.59649877470392    3.01162179115290
H     9.50065447181227    4.36614359558626    4.50491010109544
H     8.44546463333036    5.28524287997630    3.38984765230364
C     0.69685995423612    4.15795435639556    3.09353364607220
C    -0.16491242546328    4.48206191982672    4.13331810965650
H    -1.20701802803395    4.18526010828900    4.22315828461624
C     0.58181220238244    5.27606060047667    5.02105140496902
C     0.44202728203024    3.33122031807370    1.88265327250598
H     0.49851783019753    3.92704540890641    0.95508700987583
H    -0.56289632182379    2.88527739897639    1.94387141085568
H     1.17497354243388    2.51068119684956    1.79244378928378
C     0.10058990611540    6.02750859075936    6.23886673129118
C    -1.26663755827674    5.49641834265995    6.67517822864064
H    -2.03005597493918    5.63947882839305    5.89099320774885
H    -1.60765048893056    6.03397205446625    7.57699856913398
H    -1.21597499017777    4.42058153266203    6.91952420330645
C    -0.02429193837434    7.51190986269974    5.85498536749388
H     0.95306538193918    7.91771829396364    5.54278710128505
H    -0.38838032428527    8.10222397530504    6.71613133798846
H    -0.73142255325188    7.64707083093719    5.01796617823674
C     1.07972275041917    5.88545631889910    7.39936714585821
H     1.20305162180162    4.83615250823313    7.69860285944994
H     0.71453830854416    6.44759178223658    8.27537992217337
H     2.07713481982392    6.28775911514798    7.15301111125259
C     3.35660484510590    7.34392144357224    1.39919956083907
C     3.77510223868002    8.56741059779724    1.89912024178268
```

H	3.83704802092592	9.50238858752430	1.34833300229872
C	4.10392709130447	8.33581516895945	3.24712534838964
C	2.89804331354085	6.99831248958190	0.02460903115504
H	3.52731437999317	6.21537912619874	-0.43292253916385
H	2.94515458209977	7.89284394391755	-0.61603354647771
H	1.85801113808740	6.62861952952394	0.01962131989657
C	4.65017353657513	9.34794017532297	4.22628525098601
C	4.79617873012337	10.71105336551339	3.54390670178716
H	5.20245435252645	11.44283522136745	4.26419025355807
H	3.82443683321576	11.09436584749624	3.18651004654269
H	5.48783970672898	10.66357652043895	2.68488379031184
C	6.03551288737591	8.88208970633832	4.68690973150775
H	6.46807060002478	9.60545723648100	5.39989834988425
H	6.72059134432667	8.79630190010951	3.82474995415455
H	5.96512938125492	7.90145125010633	5.18029459640138
C	3.69143255085630	9.49854888223005	5.41402011707442
H	3.56814670263379	8.54394081468246	5.94563081612558
H	2.70100429713355	9.84539779942535	5.06944349787908
H	4.08318146349341	10.24033261128261	6.13380997317142
C	5.27839057901989	6.89133275200534	7.71527956302308
B	3.06534328061171	4.97629211448017	2.38948105632560
H	2.69603528873868	4.66297259702404	1.27836889590117
N	3.99681703284939	4.78448181178017	8.88610303867602
N	3.71118838614538	3.82603085285956	6.61659032365958
C	3.82892002594338	4.27852794132439	7.81881267735492
Ti	4.87115236790820	5.85455698573361	10.35008300422667
N	4.24810894639788	7.64967891529956	11.46850633771851
N	5.03837127768199	7.73039273707117	12.56373551667447
N	6.64525385503470	5.10375665208849	11.37515325807466
N	6.78865564985571	5.81151928063657	12.52079312519153
N	3.84647867725213	4.94547823155928	12.18924672600590
N	4.43599944186042	5.42592633518501	13.30946622311382
C	5.03140039045364	8.98735302463969	13.05588793455982
C	4.17207021147204	9.73583494024514	12.26096040458201
H	3.92445834128019	10.78635364293004	12.39194527605792
C	3.68118291809298	8.84999607361337	11.28657287395958
C	5.82143893265970	9.42121068079161	14.24079646440740
H	6.89725989114158	9.21711445593248	14.10836446894419
H	5.69649211027139	10.50493230874865	14.39014425785500
H	5.50007251293023	8.90580783500169	15.16205638921244
C	2.55964260173096	9.09420923644754	10.30355401184009
C	2.80745500340838	8.39905420957593	8.97176272853286
H	2.94002919096467	7.31031022379123	9.09222128245936
H	1.94203568357740	8.54524872092681	8.30377399350158
H	3.69682367060237	8.79699191498513	8.46501928320575
C	1.27492045363418	8.52886308351347	10.93418138842708
H	1.06070103539734	9.01254985108256	11.90309934713824
H	0.41345259134732	8.69874852947862	10.26271225517986
H	1.37397236219905	7.44377472611197	11.10800273062724
C	2.39065203584056	10.59515186525936	10.05491869734422
H	3.31726353506296	11.03984954343798	9.65138887504487
H	1.58543979311855	10.76353507527598	9.31883979045744
H	2.11817028730625	11.13783672579254	10.97646620794545
C	8.05625416284600	5.71392258966481	12.98035487512806
C	8.74347511896018	4.87538029981821	12.11554563413508

H	9.78302703629670	4.56555390551230	12.20518579582631
C	7.81882122767629	4.50226344277892	11.12566912147849
C	8.55668904010846	6.40081285566916	14.20237342544485
H	8.04642514108957	6.04740113470740	15.11479830499991
H	9.63516057684729	6.20835895005360	14.31499744004030
H	8.40889642916718	7.49238681640618	14.14447162235698
C	8.06567936787436	3.53399260127053	9.99476373837751
C	9.30554388983358	3.97281813817697	9.20515747816760
H	10.20602280073175	3.99675111776255	9.84309030201550
H	9.49621438625383	3.26977407883383	8.37457586909390
H	9.16333510735906	4.98018868749487	8.77840160662205
C	8.31562720716570	2.14313305442531	10.59940149046730
H	7.43923249815013	1.80738601344437	11.18035673658542
H	8.50115136479476	1.40666877014823	9.79646384909620
H	9.19040083832122	2.14638380449548	11.27268357988066
C	6.86422208951574	3.46547592930551	9.06612336241662
H	6.63124898780350	4.45488551957182	8.64216828995995
H	7.06648318221234	2.78091897570768	8.22558107549740
H	5.96954684730704	3.09821127888293	9.59417890629902
C	3.83571669440169	4.95299165216537	14.42092419526342
C	2.80849233660794	4.12113682872837	14.00081310749176
H	2.12277387806011	3.57097826025529	14.63994840445856
C	2.84810562016627	4.14545314198436	12.59457023777549
C	4.25375205837694	5.30262041314881	15.80774949016597
H	4.16392482469370	6.38518829274963	16.00370366092535
H	3.61621510534594	4.76980952859117	16.53066769373996
H	5.30272022247442	5.02008821067306	16.00256346495473
C	1.94012669271759	3.38130963435331	11.65631502560127
C	0.86712637697738	2.63866844847526	12.45743597708968
H	0.20010403272629	2.09453179161338	11.76588468241141
H	1.31027790827931	1.89983976560203	13.14768698315225
H	0.24628885458369	3.33574626660652	13.04702975230041
C	1.24540533348696	4.34765243812100	10.69046913529883
H	0.58700369619089	3.79112527717733	9.99876604057242
H	0.62389755964024	5.07142714066367	11.24735139720033
H	1.98506152606863	4.89980949128104	10.09146714344284
C	2.77593815613183	2.34992553495058	10.88887322734580
H	3.53913910977386	2.85160359199173	10.27439808738765
H	3.28030464166403	1.66103550996426	11.58951350503624
H	2.12978530630655	1.75083345681691	10.22280249060263
B	5.57734689095141	6.44149876323202	13.23457985776943
H	5.93280611983201	6.74216318827499	14.35383647344892
C	6.91579780453693	8.09709414073248	8.93931280009249
C	6.33628636459638	9.50406102988204	8.73227159745575
C	8.00463500562981	7.85514532170678	7.88469110448133
C	7.56866541337881	8.04112631832995	10.31965362947309
H	5.55868005649308	9.68651787802053	9.49420474002941
C	7.43638643152597	10.56373302602507	8.84345592384278
H	5.85167412945057	9.55352242855910	7.74008600138164
C	9.11085947780782	8.90822589282623	7.99620755866781
H	7.55236863105679	7.87666997052541	6.87818879968463
H	8.41985699138828	6.84188723970350	8.03082050122812
C	8.66721686525807	9.09787620640438	10.44633646698521
H	6.80141706137860	8.20514621818192	11.09123265172497
H	7.99490093194323	7.03728674326644	10.48051499147910

H	6.99544337501951	11.56472423990341	8.68632367147544
C	8.51176030748709	10.30243026336677	7.78427786472196
C	8.06353555106020	10.48988062547741	10.23906666488159
H	9.87881197634370	8.71266238061368	7.22657795361791
C	9.74389757941090	8.83587545670886	9.38934637134623
H	9.11357250683784	9.03066939806882	11.45534627333467
H	9.30507536828212	11.07035838567776	7.84907678714300
H	8.07335576335480	10.37627227411247	6.77251633897082
H	8.84323285402446	11.26687337289652	10.34896654781288
H	7.29330390888314	10.68484796691389	11.00879156454180
H	10.55885302791521	9.57848284725754	9.47726047488008
H	10.19348969626498	7.83841005746604	9.55115421088896
C	3.21669674125970	2.44014455680499	6.45031054023326
C	4.08907246826831	1.45935936293816	7.24695914168144
C	3.27915844922279	2.04764499623112	4.97447015145668
C	1.76445142951455	2.30040809480712	6.93018105505386
H	4.06371306417826	1.73098083654167	8.31703223572121
C	3.60320218665022	0.01992951041599	7.05413186906005
H	5.13662167804441	1.56225028951326	6.91214290325638
C	2.78053567568477	0.61485530408927	4.77238897813625
H	4.31775093948972	2.13657380167732	4.61403057929667
H	2.66294719435750	2.74643103163723	4.38707293517943
C	1.26885012908212	0.86284911228692	6.74395415685360
H	1.70626369401515	2.59281233337092	7.99453768452917
H	1.12823590207072	2.99546028880109	6.35578160359972
H	4.24788005882035	-0.66319109855470	7.63541997173763
C	3.66950182977833	-0.34493874702507	5.56819919382901
C	2.15587392372965	-0.09781969782160	7.54155996506224
H	2.83481589448331	0.36638058248016	3.69689271097445
C	1.33221787489937	0.50214215936569	5.25645585113768
H	0.22585778091829	0.78940992831025	7.10135884090723
H	3.33971687228615	-1.38977498298706	5.41718876385546
H	4.71272345542648	-0.27809868252164	5.20749488123570
H	1.79726637271852	-1.13688567742295	7.41933957543185
H	2.09930505329093	0.13813745558081	8.61957684606905
H	0.95297705801836	-0.52428203955992	5.09435573686975
H	0.68630364620565	1.18721393916956	4.67599216047361

[(Tp^{tBu,Me})Ti(1,3-μ_2-NCNAd)$_2$Ti(Tp^{tBu,Me})] **(I(μ_2)) (S = 1)**

Eh = -5351.456767551002

Ti	3.89861253163481	5.50881671962351	5.18783317438859
N	5.08364993637017	4.42264137042168	3.65606084653963
N	4.31743015586768	4.12550015237873	2.58462730192508
N	1.83519339000339	5.32499868646453	4.41326501733141
N	1.87264037225970	4.72019988765180	3.20518845816747
N	3.87484138912807	7.04402387974635	3.43119686592071
N	3.43094371970330	6.45243130762193	2.29809419431084
N	4.74022139593711	6.69900408259967	6.69600613979236
N	5.90246096578782	7.04380777217495	8.83850113807338
C	4.91702435579219	3.18644490237326	1.81827991524587
C	6.12699137470870	2.87799969287525	2.42235374319056
H	6.86842924249538	2.16773392307075	2.06100516050819
C	6.19849019221951	3.68746521996775	3.57146699311765
C	4.33526490899922	2.64857081558877	0.55641383805019

H	3.33641917417538	2.20940070084068	0.71840381637793
H	4.99215798373303	1.86165233020646	0.15403887447714
H	4.22700733753825	3.43306016120555	-0.21246896884686
C	7.35626011531143	3.78349426524222	4.53758205338706
C	7.67014568080109	2.39314602942492	5.10472966980659
H	6.80213412696506	1.98834229263637	5.65259162155749
H	8.52277964650730	2.45047910277225	5.80555447653439
H	7.93237781443659	1.67738951530509	4.30636438827706
C	7.03230050488295	4.73844072577461	5.67708036226710
H	6.81403784077688	5.75016852797194	5.29859486854527
H	7.88458228165129	4.81618942808112	6.37388855231869
H	6.16395116869243	4.38596531821987	6.25806706361425
C	8.58040116609646	4.31370714441381	3.77539611149022
H	8.85782427822552	3.64931711153031	2.93879169340817
H	9.44816852903516	4.39121809170479	4.45551863703680
H	8.37601315211722	5.31629976160615	3.36124271547809
C	0.66936945600131	4.17713629041309	2.91399029189559
C	-0.18122571406896	4.47308942561745	3.97174805626117
H	-1.22993307877186	4.19737946197621	4.05229711907014
C	0.58815772720202	5.21680721159820	4.88559081231626
C	0.38461427920034	3.42353951583146	1.66189789880077
H	0.45930092630395	4.06839735035687	0.76911330558309
H	-0.63507291764902	3.01012534925211	1.70239568407257
H	1.08823081490988	2.58549364374572	1.52232751426278
C	0.10999333515182	5.96966610955566	6.10636425856310
C	-1.22525625775325	5.39641164984362	6.58798189330958
H	-2.01371848737589	5.49591670209491	5.82221952257339
H	-1.56385312923023	5.93854009225218	7.48801579413658
H	-1.12922316278764	4.32791664929447	6.85045589681724
C	-0.08086285344547	7.43772285362419	5.68535826713345
H	0.87413847125580	7.86850585643275	5.33900508736585
H	-0.44596386485314	8.03820368399670	6.53893440934070
H	-0.81213966472118	7.52262563220914	4.86265585113768
C	1.11419662198390	5.90911319086084	7.25182408270082
H	1.28792415030869	4.87888355340252	7.58994602597596
H	0.73580297984710	6.48713346864217	8.11162450928838
H	2.08687833672262	6.35304620386481	6.97530011875922
C	3.34870088743463	7.34292063058789	1.28416645051361
C	3.75891981452557	8.56644524727538	1.78709596052629
H	3.81696129828224	9.50318310966735	1.23885702157958
C	4.08036655554358	8.33433057757346	3.13823259542579
C	2.89461039426002	6.99932453440646	-0.09251059887663
H	3.53187178963599	6.22561104912143	-0.55490864792323
H	2.93350951539012	7.89821961689532	-0.72757592149563
H	1.85810846413637	6.61962402060295	-0.10176762958879
C	4.60240944121524	9.34844080424666	4.13094883716531
C	4.71166466295742	10.72348986574873	3.46533816721357
H	5.09493031089884	11.45836526122009	4.19509427548458
H	3.73010297098833	11.08218213658765	3.10954589609509
H	5.40633758055994	10.70588057609748	2.60760796006206
C	6.00288776088164	8.92118200521816	4.58648934019588
H	6.40606621918006	9.64031738963812	5.32197855806845
H	6.69326918371920	8.88505540631623	3.72525523090499
H	5.97772249688179	7.92667531147343	5.05586332786956
C	3.63858577394094	9.46041676510944	5.31943150077741

H	3.52279700364214	8.49443888823049	5.83234416144044
H	2.64547024624121	9.80260440130821	4.97827802827117
H	4.01969697690447	10.19109970503454	6.05637299833631
C	5.32323235965868	6.89753023161899	7.70879922814825
B	3.06737583122517	4.96449710344024	2.24915627137339
H	2.71110496939437	4.66531312492118	1.13112553466222
N	3.90210826597345	4.75400080574631	8.94756388746898
N	3.66381280628635	3.83397237613146	6.67660234326754
C	3.77317257810594	4.26762868477376	7.87323800487825
Ti	4.84444198169660	5.86901052609540	10.42302355561396
N	4.27913256034066	7.65716214780608	11.58775728014124
N	5.08625301018663	7.74277287968118	12.66879962199772
N	6.64966238181324	5.13492363057095	11.48251054162221
N	6.80727303734520	5.80838241935575	12.64356855495385
N	3.86182218857226	4.96092444040571	12.30524445135794
N	4.44859317726365	5.44930025682583	13.42295740432047
C	5.11131979851444	9.01045058617288	13.13716262537732
C	4.25670585369649	9.76008919015321	12.33856056553115
H	4.03318715498190	10.81839231474831	12.45080464490652
C	3.73706306008602	8.86423496347943	11.38616606272640
C	5.93037386892512	9.44631414230821	14.30167678704123
H	6.99975010077368	9.21820353045799	14.15472701069332
H	5.83073115496329	10.53443820042962	14.43718840129036
H	5.61436310393134	8.95133207900049	15.23622421377677
C	2.60945332008973	9.10001952719737	10.40686611538785
C	2.80688673804392	8.33251996133651	9.10504484642536
H	2.88405821139510	7.24279186670199	9.27070974346193
H	1.93884938640268	8.48956736429150	8.44311274835219
H	3.70334629334116	8.66446537339158	8.56408418490644
C	1.31518548310812	8.60966457024688	11.07911539033747
H	1.12879746653015	9.14734143567856	12.02489692234085
H	0.45070872653824	8.77243086167826	10.40964378092820
H	1.38366838975283	7.53183386539075	11.30590260975780
C	2.48889901359431	10.59187407251630	10.08573988425694
H	3.42096046758990	10.98078170635275	9.63949022030830
H	1.67200141948300	10.75531365821519	9.36142139667267
H	2.26063374314951	11.18857562756491	10.98560649292616
C	8.06546371569078	5.65601272599972	13.11422156421398
C	8.73765794450633	4.83226117852837	12.22446242199379
H	9.76847575942189	4.49308950348498	12.30832142989958
C	7.80788853979066	4.51696625361349	11.21679263390014
C	8.56487632107332	6.27530651788509	14.37296666603916
H	8.04420377564534	5.87757666874939	15.26131621730177
H	9.64036351490662	6.06556025447330	14.48331370769552
H	8.42742773725164	7.36941230589671	14.37467700401932
C	8.03705695349699	3.57986839950338	10.05476249379876
C	9.28265281972300	4.02118565017633	9.27553926499843
H	10.18394203458065	4.01724294585563	9.91280742121668
H	9.46339776381482	3.33657852922626	8.42732130323491
H	9.15343755924739	5.04043044349461	8.87326941190208
C	8.26349737098668	2.16710841267019	10.61642459816557
H	7.38104246325998	1.82835773230707	11.18647476284185
H	8.43795392669196	1.45235882385746	9.79168699795915
H	9.13714767935199	2.13506866009121	11.29041575402489
C	6.83414476274899	3.55905136855250	9.12434085512059

H	6.63299171216054	4.56062630952931	8.71117870976200
H	7.01566223796854	2.87852896551500	8.27505065221534
H	5.92964003508932	3.21182196262423	9.64936700469384
C	3.83559261184763	4.98850956272310	14.53589419297418
C	2.80411501177267	4.16433260227512	14.11611531814639
H	2.10960568482166	3.62483257998673	14.75481334810098
C	2.85355395313537	4.17534977068194	12.70848466800471
C	4.25006989288737	5.34451930676756	15.92211970404352
H	4.16984730368289	6.42920236717709	16.11111337429001
H	3.60329915709666	4.82286659715300	16.64496390933195
H	5.29512561714438	5.05313362284873	16.12581093119168
C	1.94622080330021	3.41233238933739	11.76652192127376
C	0.88109588010787	2.65709243202261	12.56699190636998
H	0.21948630121167	2.10621854634677	11.87546248421232
H	1.33325331241533	1.92302501646865	13.25638264914048
H	0.25307187872571	3.34661070614901	13.15776002238735
C	1.23321214701194	4.38120439102221	10.81397881942181
H	0.58057502166816	3.82321635042696	10.11729635738895
H	0.60319627865110	5.08727403440584	11.38381435266260
H	1.95332270677829	4.95468752041923	10.21195521653595
C	2.78342268006411	2.38773976094670	10.99024422943932
H	3.57092829537478	2.88703331248594	10.40584613680532
H	3.26051302063010	1.67511951881186	11.68614447278442
H	2.14444282567858	1.81453682849220	10.29416078902816
B	5.60353341468054	6.45382119945800	13.35760142865648
H	5.95566238451408	6.75119275086936	14.47771733400025
C	6.94523923235647	8.08383723555778	8.94527502851321
C	6.33876402457802	9.48753226377875	8.79256069786858
C	8.02028818543704	7.89346311558180	7.86525180581684
C	7.61513363942801	7.99485481447639	10.31646255979464
H	5.57084197590630	9.62920451417633	9.57240018226846
C	7.42279819865799	10.56122456334277	8.92121732001568
H	5.83693038627008	9.56341771418648	7.80994964816785
C	9.11030376266527	8.96105928352665	7.99700403805044
H	7.55178152715582	7.94534542012674	6.86635153607055
H	8.45396532902077	6.88253773149015	7.96734445377865
C	8.69865192473225	9.06536661831905	10.45943942183752
H	6.85274127183453	8.12587968059943	11.10076613708542
H	8.05742534587197	6.99323129768139	10.44564692221489
H	6.96224233713230	11.55850274504874	8.80233165941597
C	8.48442548396348	10.35050189889223	7.83728715655518
C	8.07176244578885	10.45378736471370	10.30444448307362
H	9.87039482161656	8.80294955829858	7.21130333876504
C	9.76328130798481	8.85408919295679	9.37885956166490
H	9.16082733074484	8.97297529402846	11.45886821255252
H	9.26559123481184	11.12969276022589	7.91223829425330
H	8.02736390132848	10.44638991554042	6.83530539900709
H	8.84219389675470	11.23803162905490	10.42639546743457
H	7.31073922631128	10.61389430244654	11.09100011723397
H	10.56726109082456	9.60674952920551	9.47955023628233
H	10.23152949080359	7.85978904343135	9.50222877376913
C	3.20939924985654	2.44193723882807	6.48729999352024
C	4.09340703478401	1.47053930197798	7.28302206806179
C	3.29586083465263	2.07394993231607	5.00564892075742
C	1.75206856407329	2.26665142439295	6.94310742486263

```
H     4.04960643609458    1.72668863256541    8.35698012333991
C     3.64003022661008    0.02468023536512    7.06570075599548
H     5.14230714740309    1.60056831807080    6.96183277733896
C     2.83409490137707    0.63215378676789    4.77988844565275
H     4.33476006477320    2.19589730345653    4.65640832046539
H     2.66581819961080    2.76507199314555    4.42136640586563
C     1.29161965654191    0.82085305700909    6.73430234065228
H     1.66557970261054    2.54694877444852    8.00937886948407
H     1.11246863160087    2.95443347289478    6.36300608639077
H     4.29148197586881   -0.65230304978947    7.64645545759283
C     3.73366862621524   -0.31798259503344    5.57576797447138
C     2.18975692110917   -0.12655077796045    7.53538584858273
H     2.90893505215045    0.39979417289581    3.70235681507161
C     1.38281436240277    0.47902410037130    5.24389335821925
H     0.24600425895472    0.72037745907442    7.07678762837216
H     3.42873746365634   -1.36745794328114    5.40649458831006
H     4.77985720037879   -0.22439497685118    5.23005708194545
H     1.85406432545774   -1.17197525097325    7.40352506935789
H     2.11661309966080    0.10270462909590    8.61429294344259
H     1.03126524286871   -0.55452363411902    5.06572090433620
H     0.72760368864155    1.15433448792814    4.66259394431760
```

TS$^{I(\mu2)\rightarrow3}$ (S = 0, broken symmetry 1,1)

```
Eh = -5351.428325909633
Ti    4.11830637471581    5.67705200422314    4.98597703174233
N     5.14717350512840    4.21520463906651    3.67075633171186
N     4.34600182343706    3.89376080071147    2.63036389639354
N     2.07475548191914    5.75388780628156    4.10678340296560
N     2.03418836303560    5.01232352672612    2.97710557262152
N     4.44601539332410    6.91772019576586    3.05767703310986
N     3.93600707876040    6.27933013599306    1.97935524054658
N     4.88798346887450    6.71345329182205    6.46911449049223
N     5.53299107903118    6.79833418226496    8.84305606205437
C     4.75171582246062    2.73459391506306    2.06442148797701
C     5.87253581262407    2.30810598020910    2.76126925372627
H     6.46125754435616    1.41598203850816    2.55607737387015
C     6.09726103573966    3.27770182646769    3.75637204309896
C     4.08024025280242    2.10212221299806    0.89536336708510
H     3.00776843073807    1.92789799496327    1.08446251431767
H     4.55184019621069    1.13032526383086    0.68196154735570
H     4.15812598048594    2.72803780979261   -0.01031869274181
C     7.26628664589340    3.33602626156410    4.71229411546979
C     7.36630753639330    2.01950907579876    5.49210711277540
H     6.45950265185373    1.85278760761916    6.09739659157707
H     8.23471971399725    2.04711420923120    6.17465752948025
H     7.49330846759711    1.15483045931854    4.81777483492719
C     7.11905908783636    4.49749366711529    5.68504577060057
H     7.04577260699189    5.45923037073687    5.15051707267488
H     7.99286869574961    4.55301478062823    6.35627040819221
H     6.22226157348644    4.37775288098309    6.31225749898643
C     8.54417094338742    3.53388358193182    3.88177023977304
H     8.69489906546753    2.70596099443752    3.16750753437311
H     9.42584821248308    3.58041872052407    4.54673708920515
H     8.49447155685490    4.47504846767730    3.30702788855969
```

C	0.75569283091413	4.70263217886363	2.66478048967369
C	-0.05626417043935	5.29056166486408	3.62491054408832
H	-1.14334280005834	5.25606750273167	3.65951667241486
C	0.81488599864054	5.96224817507890	4.50300326412756
C	0.36813842672200	3.88339902496359	1.48345374951132
H	0.62967683263394	4.38211506037819	0.53420869576419
H	-0.71923623896378	3.71206405667952	1.49302429649453
H	0.86864518224172	2.90046805483128	1.48829158454823
C	0.44934447610620	6.93989941618948	5.59459069096337
C	-0.71341121789469	6.40921134548485	6.43833673448592
H	-1.59998502472211	6.19177282331228	5.81751801853949
H	-1.00737787148317	7.16256060200891	7.19038195183571
H	-0.43503860618356	5.48676863381805	6.97428446339068
C	0.01678748109707	8.24386419005449	4.90088135788669
H	0.83751199848170	8.64254862508613	4.27954204929974
H	-0.24935966107639	9.00596419008634	5.65589311459038
H	-0.85853416876999	8.08029038836763	4.24864001830922
C	1.65169380125016	7.23470017363402	6.48122624706697
H	2.01675215490108	6.32916422038495	6.99115657412659
H	1.38931366088069	7.97918951112347	7.25076275467062
H	2.48274195754226	7.65898167011850	5.89314721005625
C	4.06177293118875	7.03406725114656	0.86534938307851
C	4.68846194098795	8.21066660538726	1.24275717110708
H	4.94951312410562	9.04011841357164	0.59087911723384
C	4.91560599883202	8.09495350603513	2.62782228122056
C	3.58736285896943	6.61522709720906	-0.48344409879565
H	4.07444930533782	5.68422110004577	-0.82130023694176
H	3.81705477374586	7.40530789098954	-1.21541991044488
H	2.49737529880254	6.44106020823283	-0.49931500896674
C	5.61935018340658	9.09328337272799	3.52012123287284
C	6.04518133783428	10.31177935461018	2.69744744386666
H	6.56655112234155	11.03606758799945	3.34727032194529
H	5.17406112012977	10.82404463405638	2.25296573752958
H	6.73518422779479	10.03054029393310	1.88312653097838
C	6.87195675409669	8.43027834307628	4.10503584792564
H	7.43107790251765	9.14415820515899	4.73589909550465
H	7.54059331089009	8.08230432950253	3.29801209037095
H	6.59341721923120	7.56495011932741	4.72599056736496
C	4.68209870655491	9.56059252109601	4.63989622520333
H	4.40664673189419	8.72287236581214	5.29903092658678
H	3.76086817781773	10.00188885496845	4.21982915739665
H	5.17988566367934	10.32858065306762	5.25905377647071
C	5.05610352953730	6.51661793987890	7.66666628877375
B	3.29659124327725	4.89011275432777	2.09053782712988
H	2.94804305616985	4.50720964631207	0.99607832558826
N	3.98797120436928	4.59338980108589	9.12504404232477
N	3.68712467563843	4.30498764800903	6.70170691863346
C	3.98637105982863	4.69862514294776	7.90346390276177
Ti	4.84882889918130	5.57274964753776	10.59001262656107
N	4.04260553476893	7.25250737484294	11.79410050322878
N	4.92886880299190	7.52868733702510	12.77710258714458
N	6.82843858956914	5.18365266778150	11.52150430521320
N	6.96065931346067	5.91875543548950	12.64821282404058
N	4.25564940988156	4.52867769518991	12.55843955885680
N	4.77905029316542	5.18103710427802	13.62196681990239

C	4.76463830346015	8.79969155480227	13.21102833556754
C	3.70964019462344	9.34612002884979	12.49322076540302
H	3.29736958915971	10.34669956990789	12.60983486500629
C	3.26689105216345	8.32759206484166	11.62687455162948
C	5.60197827079262	9.41879868203139	14.27472333224407
H	6.67589120883847	9.35818650720112	14.03015262778372
H	5.33697828699744	10.48174789752042	14.38429646187713
H	5.45785744830676	8.92451969301432	15.25088249833172
C	2.00868409554219	8.31492215255244	10.78768757420728
C	1.95677803251070	9.51278461894478	9.83276855800099
H	2.71979787046303	9.43014477081730	9.04277860776291
H	0.96733196335357	9.56133346276803	9.34409054600533
H	2.11609633192379	10.46624525261131	10.36647471319848
C	1.90656659251074	7.01457296933470	10.00153659379212
H	1.93704706540702	6.14244076516527	10.67478014174122
H	0.95768376247548	6.97341044995106	9.44007864157143
H	2.72728077553774	6.91998684519404	9.27294313036531
C	0.82440738463718	8.39673937944540	11.76744231875522
H	0.84519268393130	9.33415330841363	12.34991720511368
H	-0.13110752424009	8.35555358189962	11.21372463960596
H	0.84828764352930	7.55249395041329	12.47839653857177
C	8.25811990134994	5.97131085332450	13.02600965597886
C	8.98296444558288	5.22354008393574	12.11053163379721
H	10.05674067969050	5.04589375549964	12.12595730343920
C	8.04263742224751	4.73613954883635	11.18393127327532
C	8.74620113879340	6.70397132067789	14.22690157225665
H	8.34143345990634	6.27700726409805	15.16092307676889
H	9.84488309159738	6.64703871250233	14.27126113859113
H	8.46004399789020	7.76886393581067	14.20051060320535
C	8.31674443773982	3.81026023931984	10.02224690024765
C	9.34898487248172	4.45239881202218	9.08633555382212
H	10.29155350790351	4.67945559230415	9.61396954958977
H	9.58220693515342	3.76848672251434	8.25062741861615
H	8.95977872931106	5.39300067733565	8.66244539094438
C	8.88300123402293	2.49423952595203	10.57681543159652
H	8.15971775201814	2.01297272360643	11.25778533031932
H	9.09777258585547	1.79432218202380	9.74909813826274
H	9.81994234620288	2.66007093604992	11.13625330123417
C	7.03818624419488	3.52092296423003	9.24900834630224
H	6.61941501690086	4.44416462777311	8.81807983855578
H	7.23753603801054	2.82623637392093	8.41608219112989
H	6.27499830172785	3.05923834905099	9.89669402563922
C	4.37149084665128	4.62046854850968	14.78213954827664
C	3.55186167321039	3.55363902124970	14.44945919916455
H	3.05538288906700	2.88064432609039	15.14362178580890
C	3.50833513214769	3.52930892968165	13.04166537576430
C	4.77458122050622	5.11334005822501	16.12920380239460
H	4.46702366277894	6.16096858330579	16.29146769502653
H	4.29848600380902	4.49378940796737	16.90532849320133
H	5.86780666827147	5.06581027127158	16.27524015834075
C	2.77427352007125	2.53399410878478	12.17009583779356
C	2.07096547947572	1.49648199361547	13.04955474345301
H	1.54845036845931	0.76206036185566	12.41197728624833
H	2.78936244972360	0.94497995574556	13.68074620806556
H	1.31974993466591	1.96550372427146	13.70881642534754

C	1.71844795294115	3.25345887123897	11.32149195414717
H	1.12576625066257	2.52014943211445	10.74561529914955
H	1.02579647218805	3.82626019913667	11.96334536570619
H	2.19251862770678	3.94276637904073	10.60533834218014
C	3.78705342005329	1.81476911946040	11.27068981447227
H	4.28271489106577	2.53133880244644	10.59777471444938
H	4.55922160584011	1.31057451967830	11.87818717850779
H	3.28240099221632	1.05311388421672	10.64951250895919
B	5.72200690415524	6.37804982758362	13.44861390783863
H	6.09999745593458	6.77064706388078	14.53011377048736
C	6.38509472371990	8.01523411178464	8.89246252763104
C	5.52456074555146	9.27624687046998	8.73522792068156
C	7.46430694680203	8.01121094574460	7.80210345626764
C	7.09617878149454	8.08543782907094	10.24536057542808
H	4.77061978118724	9.28278957547658	9.54020065896312
C	6.38627475337519	10.53931685715907	8.82050654919054
H	4.98748444520827	9.23594506089986	7.77042416630936
C	8.33216731683081	9.27021165935374	7.89200632876853
H	6.98870492522041	7.95075081621362	6.81185447943654
H	8.08799195417285	7.10672290282690	7.91888266772010
C	7.95446730053426	9.34744479439137	10.35384210598163
H	6.34933057487387	8.08050992590883	11.05380348585268
H	7.73060059122595	7.19300452497699	10.36969495578519
H	5.74231821769152	11.42832342303176	8.69322624534212
C	7.45044325454963	10.51110060323841	7.72006606549990
C	7.06941059822652	10.58589861568422	10.19099133177769
H	9.09160036765908	9.24183100341069	7.08989173962472
C	9.02192953253929	9.32398079750761	9.25795137556101
H	8.44013192803736	9.36239706269155	11.34688215605210
H	8.06953959196842	11.42659057671232	7.76512048545748
H	6.96631166975727	10.49590686363505	6.72690434182755
H	7.67704121351683	11.50561756575812	10.28569044964111
H	6.30614268205815	10.61062634739877	10.99145660430412
H	9.66547639458297	10.22096871056653	9.32773314836587
H	9.67572543511458	8.44174515084111	9.38951547924538
C	2.92546551032958	3.03227231585687	6.64475084806995
C	3.60444983406431	1.91518824846039	7.44903225418969
C	2.82492790874423	2.56291020331369	5.19204895234895
C	1.50348192083575	3.24107937289823	7.18446935313940
H	3.70530620505255	2.22434744753005	8.50073566697902
C	2.79819165735163	0.61578751025434	7.35721374644443
H	4.62522502561555	1.76256325326892	7.05404532407249
C	2.00936971388590	1.27237403921056	5.08558315973646
H	3.83850345336549	2.39623166178834	4.79201894798840
H	2.34678280861402	3.34713708843984	4.58536906544261
C	0.69321435713645	1.94486119562823	7.09717626838097
H	1.55553255124686	3.59693047602542	8.22911380780490
H	1.01872770072612	4.02914509822707	6.58353014133641
H	3.30444525767981	-0.16542562323639	7.95244738593280
C	2.70130490141835	0.17309325224466	5.89472462777539
C	1.38894535122935	0.84957345371110	7.91050610380966
H	1.95320546203891	0.97469052679525	4.02249250185802
C	0.59870742524806	1.50982142012203	5.63123457260647
H	-0.32006769435083	2.12138434117808	7.50105805741694
H	2.13668114424516	-0.77486662553866	5.81739859110670

H	3.71203376171380	-0.01289823057023	5.48674503927952
H	0.80325019191649	-0.08740756561950	7.86516249599921
H	1.44527931792536	1.14610497741370	8.97327085725607
H	-0.00635534766023	0.58801732570789	5.54344113935603
H	0.09353651816537	2.29363303332052	5.03637574962724

TS$^{I(\mu 2)\rightarrow 3}$ (S = 1)

Eh = -5351.416761030096

Ti	4.09648189501316	5.66003260991421	4.99981114237916
N	5.14468344236942	4.22420347298660	3.66168535257411
N	4.37137776945151	3.92237477790158	2.59522793478436
N	2.05176488971522	5.70536398220876	4.11406782385541
N	2.03050605919313	4.95958805665018	2.98700480946973
N	4.40317688975921	6.93515179619953	3.08460305321612
N	3.88203386016563	6.31117892492849	2.00297740742531
N	4.87256903766419	6.74475325728515	6.50108781669470
N	5.56053571884429	6.80638956962708	8.86946606537416
C	4.82657452685554	2.80586497276559	1.98232870355591
C	5.94907123407924	2.38517575906663	2.67925423576334
H	6.57125567004844	1.52340926332420	2.44463442535990
C	6.12253043223708	3.31404240757916	3.72249335001650
C	4.20261163461142	2.21081526936632	0.76756932169874
H	3.13307583300962	1.98927710080260	0.92049686653879
H	4.71420259069561	1.26879356367966	0.51569189280850
H	4.27748077486708	2.88528361822677	-0.10297578242516
C	7.25700175200875	3.34306000579509	4.72009985026170
C	7.27557490736514	2.03014843149574	5.51371053470270
H	6.34344772257371	1.90842956543430	6.09107357125747
H	8.12253221532174	2.02469182287323	6.22345218380868
H	7.38451845512950	1.15575256357677	4.84868870486863
C	7.11328736774314	4.51824153215513	5.67719699313523
H	7.07175044856318	5.47551267614850	5.13124578159071
H	7.97252938432589	4.56173085028800	6.36798242072520
H	6.20169261379627	4.42258946597478	6.28733686415965
C	8.57432985960996	3.48553206507562	3.94238360180477
H	8.72602023442239	2.64560954363366	3.24257284282013
H	9.42839853657188	3.50631900796730	4.64373287580131
H	8.58415989400550	4.42204782477133	3.35809979492994
C	0.75943639093750	4.62321809689654	2.67041158409029
C	-0.06857511028771	5.20085223898342	3.62311175862434
H	-1.15479858927408	5.14334300594483	3.65478159627376
C	0.78645858346691	5.89339133561178	4.50159098703327
C	0.39732865630299	3.78883265412732	1.49176166202254
H	0.63168647746187	4.29775858262491	0.54069755519968
H	-0.68205158208272	3.57289211285919	1.50820490613459
H	0.93877948239497	2.82773365601935	1.49348360288577
C	0.40412994178701	6.86874500620286	5.58907733647571
C	-0.77377760104463	6.33810787681865	6.41143300268896
H	-1.65142880438345	6.12903424417747	5.77521970102915
H	-1.07648678838380	7.08802796508988	7.16348058657658
H	-0.50825931254249	5.41076005507108	6.94540853864590
C	-0.01374293399903	8.17546990824523	4.89154726322550
H	0.81840793390846	8.57329131103844	4.28506859952952
H	-0.29104796278340	8.93666001669840	5.64355341404873

H	-0.87810755544377	8.01481042192835	4.22412747565642
C	1.59301894162626	7.15875018434876	6.49608306145547
H	1.94951830281629	6.25162063451708	7.00979712527064
H	1.31879233966374	7.90147707041154	7.26295157968167
H	2.43402904470277	7.58500627478437	5.92342745241141
C	3.95964496889744	7.09691697987675	0.90580238880326
C	4.56834749298568	8.27833814136282	1.29680420948812
H	4.79372371444547	9.12911876495386	0.65919720493223
C	4.83533394226219	8.13286068904145	2.67214376706298
C	3.45925551663150	6.70194227590738	-0.44091314801688
H	3.96213192490539	5.79513379145341	-0.81930094600238
H	3.64534588972816	7.51860436543533	-1.15599324006068
H	2.37456421841552	6.49689353130133	-0.43143926881626
C	5.54595039832076	9.12335392362056	3.56846452762446
C	5.96325328428101	10.34990175312013	2.75314283619842
H	6.49526092620456	11.06606153091181	3.40344158795401
H	5.08748434344284	10.86933973472686	2.32650914143113
H	6.64080406428555	10.07547425324059	1.92612613459458
C	6.80495454351180	8.45633849326765	4.13471358367685
H	7.37200794316541	9.16702496568072	4.76210265768728
H	7.46364784679914	8.11254650316636	3.31780451248456
H	6.53286384047598	7.58797423170574	4.75433457960456
C	4.62002129111942	9.58136779018207	4.70185755843218
H	4.34929910836641	8.73830972166872	5.35626177596316
H	3.69582709151763	10.02815431527159	4.29397923396431
H	5.12485374792114	10.34361789950339	5.32293060077745
C	5.07797795929852	6.56302487451463	7.68608195946913
B	3.29013173925680	4.89890636602344	2.08859235299243
H	2.94383857000500	4.53148671931713	0.98800498743301
N	4.02855848540021	4.59284333164451	9.15989973115548
N	3.70131351483713	4.27230805204273	6.74067533447777
C	4.00577052422478	4.67440278761553	7.93039094118339
Ti	4.85316966139309	5.55749886515652	10.59679672471634
N	4.06313109961514	7.25823983463988	11.75791652749255
N	4.94681691538952	7.54376951835949	12.74120408171174
N	6.83479882722847	5.18420954686313	11.50794986152941
N	6.96896433225642	5.92138807004717	12.63416432190464
N	4.26401551230934	4.54573305456614	12.56237298823205
N	4.78544589837079	5.21045903125171	13.61951178694131
C	4.78094387612322	8.81890461972137	13.16115590815565
C	3.72745760618682	9.35742514345732	12.43514696916612
H	3.31527642911387	10.35936911676349	12.53926225184183
C	3.28627088906437	8.33077630061961	11.57830184659238
C	5.61738152862085	9.44918818816206	14.21883608112843
H	6.69145127267007	9.38285165728029	13.97644764611803
H	5.35467697028710	10.51398309458630	14.31463029346845
H	5.47032942648036	8.96748271387333	15.20081090159883
C	2.03198059595128	8.31297086102125	10.73369196077505
C	2.02107694768298	9.46223826429794	9.71938978213961
H	2.78085643767171	9.31197799510761	8.93667357494084
H	1.03377582267582	9.52050617277190	9.22770514817563
H	2.21440126494801	10.43490586959119	10.20509042355857
C	1.89567110196277	6.97807933871628	10.01295944978507
H	1.91308513579527	6.13815055177383	10.72675101726633
H	0.94204287905903	6.93124268682093	9.46034489198619

H	2.70696212670810	6.83174181138757	9.28270426281264
C	0.84581817689617	8.47691562638416	11.70060650879444
H	0.88615420895045	9.44271906319497	12.23340358503606
H	-0.10703428443964	8.43101875733960	11.14297007300656
H	0.84366052572383	7.67093146527024	12.45504514248868
C	8.26559688578643	5.96476619993971	13.01561044076860
C	8.98695184782181	5.20748785428444	12.10564377886013
H	10.05918468600419	5.02147999206962	12.12483112722947
C	8.04691357069205	4.72518669999531	11.17634321761972
C	8.75633958777242	6.70128815431595	14.21299421246348
H	8.34503474784953	6.28496474905490	15.14892433459465
H	9.85429193302969	6.63463640080154	14.26100549006230
H	8.48012339734961	7.76859424961058	14.17748832684576
C	8.32306022768482	3.79562112690803	10.01806765336686
C	9.36711024860301	4.43038657670450	9.08999475203968
H	10.30772431769729	4.64976803652297	9.62430558684211
H	9.60067737972121	3.74535826213582	8.25534603342813
H	8.98807382400358	5.37415062817350	8.66404413666967
C	8.87601420344461	2.47675559084495	10.57934844140140
H	8.14373376018306	2.00112465937690	11.25469797042948
H	9.09270102662067	1.77439729726696	9.75426341639405
H	9.80943203595945	2.63674810992232	11.14630614625022
C	7.05015276532238	3.51337955486608	9.23382320939909
H	6.63689713589296	4.43914846853083	8.80335115552568
H	7.25435630191700	2.82055931149699	8.40075169547307
H	6.27999619884414	3.05086231157658	9.87268364727144
C	4.37430165375030	4.66428115841264	14.78483549258945
C	3.55526697822788	3.59374259528600	14.46254759713907
H	3.05645045402713	2.92969712389953	15.16351232216779
C	3.51501414949686	3.55125729569724	13.05539892946060
C	4.77455390065380	5.17336148747727	16.12653904223135
H	4.47047723519785	6.22420460976333	16.27367797784562
H	4.29353842519482	4.56601259603542	16.90918741189012
H	5.86712635504740	5.12337526139862	16.27666650540483
C	2.78766161076957	2.54041614911417	12.19638288789735
C	2.06443927820278	1.52881811821952	13.09013179554728
H	1.54569181500441	0.78341210935556	12.46241457552895
H	2.76914272280704	0.98667678596311	13.74438702958031
H	1.30726883362450	2.01880891675020	13.72693932360836
C	1.74954826157150	3.24321207422909	11.31332056852535
H	1.16087317487961	2.49638786026057	10.75138457022261
H	1.05110047959496	3.83776311620501	11.92858174068277
H	2.23787661080072	3.90806367629527	10.58354759034307
C	3.81061095567568	1.79324921543434	11.33132923557951
H	4.33088458752857	2.49168944441850	10.65758988456361
H	4.56246614693930	1.28937589622518	11.96407327023300
H	3.30865278540004	1.02788978941683	10.71229308451335
B	5.73490205188654	6.39955079877462	13.42981263731531
H	6.11528112051174	6.80530027608522	14.50511128295665
C	6.43034401168183	8.01437362606146	8.92120700366714
C	5.58424708308346	9.28627832601586	8.77206022192106
C	7.50203050961971	8.00010577670732	7.82271037114985
C	7.15425696905859	8.07352640670526	10.26690013968726
H	4.83507693723868	9.30152912680495	9.58131930590394
C	6.46072779587506	10.53910048953084	8.85250836531000

H	5.04109186511044	9.25335134113617	7.81040894358725
C	8.38438503986516	9.24947060401890	7.90703481208351
H	7.01979886085002	7.94558001290658	6.83532394200689
H	8.11678023635539	7.08895748519727	7.93454421861950
C	8.02667359798109	9.32639086650989	10.37112345449740
H	6.41583095857674	8.07324326596955	11.08206694582673
H	7.78165605926857	7.17528652571233	10.38246371717705
H	5.82590724777229	11.43559475729910	8.73176490284806
C	7.51520051809972	10.50022272794893	7.74333986772277
C	7.15458993783906	10.57511591049135	10.21782911492739
H	9.13695220928161	9.21365851624209	7.09884990420710
C	9.08555463162985	9.29403157505200	9.26737543772114
H	8.51991354901720	9.33361086031951	11.36062627629444
H	8.14477948856105	11.40877638567542	7.78345162038125
H	7.02215796853021	10.49084088752481	6.75444957395491
H	7.77309221453418	11.48775044762307	10.31061286625462
H	6.39718904656582	10.60629959828211	11.02371367854215
H	9.73874979725964	10.18428277566693	9.33376034406072
H	9.73121964776717	8.40484175591698	9.39237850558405
C	2.92690809939365	3.00798990212101	6.66421058739648
C	3.50620368651488	1.90787610546023	7.56274895499481
C	2.93863905456020	2.49437070909459	5.22268589417809
C	1.47150869501312	3.26672915420811	7.07663324321060
H	3.51675538739706	2.25182869583607	8.60822906412192
C	2.67935651803238	0.62349572303884	7.44120677999194
H	4.55631614226261	1.72008670218491	7.27268417465368
C	2.10315665096621	1.21974167922487	5.08866840211671
H	3.97842445896230	2.29889410459255	4.91094728070729
H	2.53224576528007	3.26888437198689	4.55392386268406
C	0.63810253451893	1.98832875060178	6.95966589925458
H	1.44873841096013	3.65489741075988	8.11150566228523
H	1.05967376412214	4.04642046256356	6.41497548464183
H	3.11362666720366	-0.14876314327644	8.10159855975862
C	2.69556412915856	0.13404943210235	5.99061322154066
C	1.23427438024683	0.90595150362846	7.86409858284795
H	2.12903169554422	0.88599165724730	4.03542742740109
C	0.65816560682265	1.50904302012848	5.50446716212466
H	-0.40142215041087	2.20156500451284	7.26813608684260
H	2.11559305100763	-0.80304309573153	5.89654772288559
H	3.73217729677547	-0.08921841221046	5.67733294212268
H	0.63184961539328	-0.01917413467034	7.79953171000197
H	1.20832189517995	1.23819844383160	8.91764095161647
H	0.03826583559091	0.59984334704892	5.39282379944523
H	0.22648737882442	2.28601254959410	4.84610523669931

3(κ^2) (S = 0, broken symmetry 1,1)

Eh = -5351.413352743703

Ti	2.11238158208795	10.21543652157054	8.39032998537642
N	0.30574469847708	9.94921565793119	8.53889544722569
N	3.81658039089453	8.86709560821101	8.76188733852130
N	4.93921554492405	9.61560599406542	8.81362023669510
N	3.17551565770083	10.31294278821307	6.42517005951333
N	4.46475919186803	10.67823544722063	6.60428096559480
N	1.46424757045000	10.15077163873008	10.45554620177251

C	5.91966702619884	8.94116584128264	9.45310272584772
C	5.40420079437066	7.69921207292976	9.80104119066447
H	5.93639011424417	6.90238186236445	10.31615769219080
C	4.07571389927782	7.69128018879114	9.33758776257623
C	7.27697571852029	9.49860165119349	9.70592048960070
H	7.80905206009748	9.73590902879441	8.76915913021455
H	7.87801180561651	8.76615669101070	10.26667113678533
H	7.22626751682970	10.42508354767087	10.30353210825779
C	3.06130715529469	6.57519615235721	9.42682379830988
C	3.77405336829731	5.23748008663929	9.19268860607843
H	4.27384785133909	5.21697856479438	8.20847506836766
H	3.04177761807580	4.41121770570671	9.22573142498534
H	4.53472763000859	5.03955602790664	9.96747588877411
C	1.97873133641602	6.75903444661520	8.36435017299311
H	1.37844691092523	7.67137864950543	8.53881957748773
H	1.27384668925729	5.90988440348494	8.38686295597422
H	2.41869455734495	6.81647456950240	7.35514486665038
C	2.42317697044631	6.57626506852572	10.82101961171334
H	3.19146470706952	6.51916815504844	11.61210343737990
H	1.74718340524578	5.70966440787140	10.93523743213889
H	1.84019292573956	7.49851815287546	10.97212451225404
C	5.11845812192358	10.70221820809281	5.42209027035636
C	4.20384262541870	10.34377979028463	4.44269955011216
H	4.40042272841743	10.26214633682515	3.37679869906447
C	2.99105056139224	10.10492190636984	5.11715571441485
C	1.65945860726271	9.71283279892171	4.51330363620676
C	1.86732995907427	9.29045366495554	3.05547303250560
H	0.90639704384144	8.96692917930212	2.61962688955721
H	2.24771487983999	10.12442068110454	2.44062627373999
C	0.71054359069052	10.91869588843071	4.54628543808832
H	0.44009559527603	11.18278049932458	5.58005390406758
H	-0.22269421018495	10.68935461426717	4.00296945600378
H	1.17682755911036	11.80206062319406	4.07649882062319
C	0.27344759801534	10.18038281966523	9.86937104853449
C	2.00672807186898	10.56697616326746	11.74172904375182
C	1.65985293008784	12.02526142369898	12.08140047659070
H	0.56588797223295	12.12027343290229	12.17394025090864
C	2.34742223526357	12.46439053476107	13.37651616616399
H	2.06250168047389	13.50941800285241	13.59788620081556
C	3.86605391381020	12.37006692638856	13.20790354886780
H	4.37774266667726	12.71175240314658	14.12756848999589
H	4.19447642209745	13.03172646150604	12.38418364627356
C	4.24427920569070	10.91955326960429	12.89682853326719
H	5.33782543956019	10.84121278928160	12.75013876799282
C	3.81000372971869	10.01388262167221	14.05116400015314
H	4.31652593571107	10.31319447880284	14.98836609558016
H	4.10448551686872	8.96864064171123	13.84107506282227
C	3.53840892066048	10.47595078833058	11.61541144180941
H	3.86552863742803	11.11173517231620	10.77720507704736
H	3.81604253795253	9.43566938907638	11.37211291697714
B	5.03953212548356	10.93204686100406	8.01093346446298
H	6.20788136206211	11.23559378182338	7.92859546820694
H	1.99829584107731	12.66268931454051	11.24733231253758
C	1.90045084762144	11.55545068778785	14.52406341261148
H	2.36818326381788	11.88011951235363	15.47345685180209

H	0.80670231989956	11.62732288240333	14.65194099152431
C	2.29065131151643	10.10850308095774	14.20973148699970
H	1.96277911980290	9.44994342318583	15.03470389260976
C	1.59833742366105	9.66125395700983	12.91801877144179
H	0.50534039961818	9.70880793506367	13.02137321338480
H	1.87678450922757	8.61935262470539	12.68816989851120
N	2.93807178020322	12.25956268610885	8.48090629087603
N	4.27632674967306	12.12608895960066	8.64957204873565
C	4.81158299357328	13.29892392660093	9.06163932088948
C	3.77920137880011	14.22196375978333	9.12786848926088
H	3.87016248104096	15.26979447081734	9.40243188662082
C	2.61667688756457	13.53533317522726	8.72626054137990
C	6.25930978640312	13.49070020205621	9.35002654968871
H	6.87734194730400	13.40228007111048	8.43991995367146
H	6.42012647687288	14.49261462169160	9.77738854201693
H	6.62962093175180	12.74671341596851	10.07509039047491
C	1.25101431628823	14.12672924398482	8.43005387694059
C	1.38163232174399	15.64783613181766	8.29849014307720
H	2.11952896058354	15.92749059490263	7.52681614644934
H	0.40623247015122	16.07941709137294	8.01398583447432
H	1.68370252193486	16.11287049597868	9.25284775934379
C	0.75862121775228	13.54773196422888	7.10121554949050
H	0.62673843637207	12.45880686537984	7.18473422218105
H	-0.21939161990102	13.98128087181295	6.82914118623925
H	1.47566163797238	13.74506425406030	6.28605901589504
C	0.22307551607331	13.82827482314283	9.52319504598827
H	0.53437772048835	14.24566176575279	10.49468065607041
H	-0.74723933691267	14.27859916078972	9.25189997432132
H	0.06287247247216	12.74761831823287	9.64896863128608
H	2.57911890014738	8.45008836093818	2.97630464456767
C	1.04076260504155	8.54303727492673	5.28477571246756
H	0.81626121736229	8.81010828851927	6.33162862438155
H	0.08756239088605	8.24230298963058	4.81554952598173
H	1.71600746623422	7.67016346025833	5.28172347669990
C	6.56136080886551	11.04480917054357	5.28281160175787
H	6.78135150846303	12.05582252202012	5.66680269201367
H	7.20469070038524	10.33606305275797	5.83252940640512
H	6.84726714342016	11.01285064031484	4.22005775622721
Ti	-2.93783286311143	10.41616761136937	12.00659007859039
N	-1.11032192134076	10.50007116764826	11.90508512869522
N	-3.83114253839163	8.44417267109296	11.65050560844186
N	-5.15134796060751	8.68158599709123	11.44710499862770
N	-4.02949097573925	10.11140349099217	13.92436505503927
N	-5.31392583988299	9.75568564437502	13.70790771289050
N	-2.24397284096933	10.67261206584485	9.97647871831777
C	-5.70697049510958	7.64778648740986	10.77101121345028
C	-4.71201043444710	6.70588871094876	10.56758710908914
H	-4.82937873113115	5.74573653520446	10.07110304831286
C	-3.54675562316728	7.23465742870540	11.15989775339486
C	-7.13942953774623	7.60157659650592	10.36958880771279
H	-7.81180102341398	7.52659234055349	11.24185338533447
H	-7.31428024330507	6.72615418768903	9.72527182381360
H	-7.42809747782514	8.50364209993407	9.80377933509319
C	-2.21069338686454	6.53805393277768	11.32432837770041
C	-2.47849484762569	5.04192836619150	11.54604384499443

H	-3.14247446479688	4.87684380983954	12.41244052414304
H	-1.52495767568169	4.51880200629513	11.73641058582938
H	-2.94286278025512	4.57192541769734	10.66201306748819
C	-1.46362998889434	7.08689762514690	12.54090779245487
H	-1.18537190031991	8.14412174589425	12.40045973603746
H	-0.53068321393313	6.51695615837971	12.69582037927247
H	-2.07798010412322	7.00600518878914	13.45411495765072
C	-1.33842188298225	6.69736052056619	10.07427935651329
H	-1.86069592095613	6.34530606789379	9.16817024524919
H	-0.41596086874794	6.10480553349913	10.19191204997680
H	-1.04762531808191	7.74554455644861	9.90800318340773
C	-5.93338266809177	9.48627274669696	14.87777980507006
C	-5.00002073171198	9.67689889341222	15.88757964355463
H	-5.16886674157113	9.55149602382792	16.95426047294726
C	-3.81319658306706	10.07437194461835	15.24226019846589
C	-2.47765188648909	10.42356568828141	15.86004940930998
C	-2.62592470858982	10.51409259585453	17.38055974870982
H	-1.65811040627853	10.78578694568426	17.83618693422638
H	-3.36716872132913	11.27942160074884	17.66964467366486
C	-1.99465366748483	11.77451546501418	15.31942405725906
H	-1.72340449965436	11.70181862143104	14.25269406231488
H	-1.08984952048555	12.09994091181755	15.86122117918357
H	-2.76921876218225	12.55272766182081	15.43568199523488
C	-1.06688588413936	10.47089854374271	10.55998461345425
C	-2.79785040144157	10.55252367445564	8.63890917328058
C	-2.18181856556446	11.53350519817687	7.63045060382265
H	-1.11144797088092	11.30354906082587	7.54304483931288
C	-2.87164331903208	11.41192308350209	6.26840373674599
H	-2.39182314161418	12.11232321935381	5.56009490668179
C	-4.35879034472724	11.74791254649033	6.40385904776190
H	-4.85958712866835	11.68028080746743	5.41976692559933
H	-4.48353079920068	12.78741195119100	6.76124209582628
C	-5.00214016550450	10.77397788732962	7.39509972736286
H	-6.07022884141877	11.02734296567005	7.52712525264655
C	-4.86717397618926	9.34139309147779	6.87265846210042
H	-5.38414597583974	9.23629340053491	5.90004567844921
H	-5.34615272445295	8.63521242712546	7.57734133120397
C	-4.29712903300789	10.88716154062065	8.74620967010600
H	-4.41484553977612	11.90752480554601	9.14767514243893
H	-4.76433072155675	10.19650762127818	9.46839757771639
B	-5.90206428447529	9.75534940032299	12.28462166146341
H	-7.07521177704815	9.45793069129716	12.32003512452384
H	-2.28061548643286	12.56370559637017	8.02010370564531
C	-2.72441176847390	9.97858172503013	5.74752069699633
H	-3.18713757780174	9.88931749124512	4.74611472869932
H	-1.65559608510295	9.72795124439717	5.64264265887169
C	-3.38181916369163	9.00468229897499	6.72771441780466
H	-3.26918871391076	7.97034091768847	6.35389603833003
C	-2.68663071309201	9.12190714624937	8.08540180192477
H	-1.61919483513133	8.86791529779131	7.99277841642024
H	-3.15171600916838	8.43054632435847	8.80818173255436
N	-4.61000455364110	11.85230223806856	11.78879651960205
N	-5.78112895936104	11.18646700851229	11.71548818661828
C	-6.76071648834270	12.01214566550648	11.28491886070461
C	-6.18864791995667	13.26303161753098	11.09213248246116

H	-6.69987338867878	14.16306950441302	10.75558189736048
C	-4.83041455607989	13.11748692822604	11.42929723136458
C	-8.17349217645400	11.58418835776493	11.08493005170783
H	-8.64844981310842	11.27863164653970	12.03297808173361
H	-8.75582710269835	12.41816250622784	10.66353184153054
H	-8.24628326623114	10.73054967714021	10.39006639432586
C	-3.75715022831344	14.17865950663453	11.45762522215841
C	-4.14622816625829	15.23272606677299	12.50445028315879
H	-4.22878423668338	14.77688813792121	13.50667329307717
H	-3.37898236498625	16.02680552986406	12.54775741133911
H	-5.11494852819537	15.70435374581782	12.26360314810710
C	-2.41817890753100	13.55647232991240	11.83831018015176
H	-2.11240202319991	12.80148891943762	11.09470731871749
H	-1.62671614731155	14.32329534624252	11.88296758015415
H	-2.47261081440584	13.08603009666330	12.83425184692686
C	-3.64942935880394	14.84244893334736	10.07854269207298
H	-4.60791568391170	15.29783844769979	9.77407891511455
H	-2.88493687085596	15.63954016600024	10.09814511566243
H	-3.36046595942790	14.10713987873163	9.30864793736992
H	-2.93939213227533	9.54855803403055	17.81361008005921
C	-1.44667715704546	9.33940299548507	15.51820649700528
H	-1.28529336752333	9.27406740508281	14.43032894739251
H	-0.47397741463393	9.57244861926180	15.98708892793907
H	-1.77805548143701	8.35021174965078	15.87917061954529
C	-7.36263100427553	9.07939325048640	14.97836119829517
H	-8.03763175277587	9.85632911834318	14.57945316892758
H	-7.56362241954505	8.14988263865722	14.41825132010263
H	-7.62531534631821	8.90824934347516	16.03379979889433

3(κ^2) (S = 2)

Eh = -5351.351077317578

Ti	2.17458202944999	10.29445585159249	8.29335861983690
N	0.23848108236157	9.48177388051732	8.78672815665971
N	3.92323406179488	9.00840684841430	8.67454776924203
N	5.06143971367253	9.73481724399594	8.68346328693852
N	3.24837519476770	10.54110148358064	6.34295078565737
N	4.53448242018370	10.91543840236186	6.54211482516757
N	1.41121781176657	10.36184597749400	10.38675434278415
C	6.09528175753134	8.98901545434322	9.13481224684616
C	5.59815492840147	7.72233120467300	9.40312031845249
H	6.16737469203260	6.87148692898594	9.76880977426942
C	4.22735897591607	7.77261506537484	9.08538175368263
C	7.48813754367766	9.49779570854518	9.27541501892243
H	7.94533830225193	9.72864942295716	8.29746767274969
H	8.10994361705206	8.73541353827373	9.77025878279954
H	7.52699743574834	10.41790320145873	9.88283251330641
C	3.22007919097904	6.64743961584743	9.15945594733176
C	3.95951894611118	5.30633864879661	9.19371433921914
H	4.61100294493981	5.18166713846995	8.31103205086317
H	3.22997523916868	4.47749970914339	9.20278205784395
H	4.58273273431516	5.20972067904262	10.09967688611578
C	2.30232932467622	6.68228112311182	7.93530370873262
H	1.67106369662336	7.58645006989158	7.93460462153666
H	1.61568831323468	5.81693883474849	7.94974592703590

H	2.88467534927164	6.65120539906323	6.99870374456484
C	2.37542993254803	6.78515311138935	10.42853137288570
H	3.01430331100741	6.80351610439707	11.32847884977012
H	1.67759234882300	5.93356082695430	10.51760174941872
H	1.78335109456983	7.71265856315508	10.40120842229242
C	5.18650022881500	11.00505805102459	5.36122264542274
C	4.28046425763883	10.67301541238925	4.36401461782280
H	4.48195452433798	10.63867894554176	3.29625401209083
C	3.07243242793747	10.38539876038035	5.02626365422279
C	1.75159829914497	9.96700556962186	4.41449697351762
C	1.95726655104986	9.63233184021771	2.93446591669691
H	1.00424471038421	9.29960725474631	2.48781941313594
H	2.30434871109585	10.51130275375626	2.36448856766892
C	0.73747221511801	11.11187838644539	4.52879302589944
H	0.51473264848112	11.33392208384760	5.58397066758895
H	-0.21119784597993	10.83867602605353	4.03361620583830
H	1.12320311091298	12.03359974312849	4.05956155739482
C	0.16382350934604	10.07632065486371	9.96631832001539
C	1.99041286396412	10.51891111310486	11.71653949223467
C	1.69405662014442	11.93597665227808	12.25152286964527
H	0.60283472583827	12.05704526721122	12.34919473139129
C	2.38758326888667	12.16206191340170	13.59713459024104
H	2.14014903333497	13.17782041141060	13.95707562315012
C	3.90200434870045	12.03064887615886	13.42129287461316
H	4.41749610026449	12.21705384660967	14.38235338987009
H	4.26531257178975	12.78770961605879	12.70152373754085
C	4.22438815566412	10.62452751576213	12.90877019014576
H	5.31443658784365	10.52245009635951	12.75702162565921
C	3.74292242740222	9.58559014001956	13.92499793550011
H	4.25532695131875	9.72954818880597	14.89498573295205
H	3.99446203120613	8.56798920450172	13.57154671031658
C	3.51671190155277	10.39016241326271	11.57326415962996
H	3.86747906599612	11.11951900773790	10.82537847249210
H	3.76555960726600	9.38648935748135	11.19075415738469
B	5.13475693164772	11.09261833098572	7.95971260351694
H	6.29906588405718	11.40796093953545	7.86123342788079
H	2.06637466062338	12.66807503228902	11.51612924848872
C	1.89305290126289	11.12402777339656	14.60624281847109
H	2.36345613178875	11.29505063871558	15.59329569797607
H	0.80147289494815	11.22296428605307	14.73594044076841
C	2.22780480187209	9.72002348183812	14.09601621397286
H	1.86605481848472	8.96844788929603	14.82120623920299
C	1.52319157855879	9.48569967916463	12.75629722280860
H	0.43375236053966	9.58224456841128	12.87404296515568
H	1.74319643113038	8.46998553580766	12.39067201314336
N	3.04559087209537	12.32268589486084	8.57203716251895
N	4.38736391259191	12.24133679589949	8.68071669286540
C	4.89531089920084	13.42657410082444	9.09114282492054
C	3.82904729854577	14.30442372439085	9.23016065690040
H	3.88902928180922	15.34905794666869	9.52588644595269
C	2.68135492704573	13.57513769947254	8.85977057014678
C	6.34946667540404	13.66936245446958	9.29823793299561
H	6.90746092645757	13.65580868999095	8.34575462195532
H	6.49838601988209	14.65425099067730	9.76762692192433
H	6.79892302436710	12.90512225429891	9.95408147725295

C	1.27742267288405	14.09082370510612	8.61230039700290
C	1.33591925500125	15.60322901692195	8.37135242879640
H	2.01495491737800	15.85501996106597	7.53849892366116
H	0.32853439601373	15.98172026486109	8.12414849851504
H	1.68040179038383	16.14087172772226	9.27146604103330
C	0.73668149817011	13.40789476550985	7.35435355509894
H	0.61148682716797	12.32440967724366	7.51775458159370
H	-0.25928267063646	13.80448699530596	7.09164384114174
H	1.41512162850959	13.54429816076363	6.49612532918229
C	0.32293852716355	13.82037172459574	9.77613769370898
H	0.65273712422161	14.33042478048264	10.69662939201245
H	-0.68518896392455	14.18933886329119	9.52142391361153
H	0.24938121613199	12.74523083620057	9.99710872346729
H	2.69693075786489	8.82313707797221	2.80442633463225
C	1.20539678797448	8.72898850926069	5.13206636236748
H	0.95397874352638	8.94816244366518	6.18504465912650
H	0.27845257310875	8.38093724618673	4.64368561462949
H	1.93733231364312	7.90403974048832	5.11576204563191
C	6.62321230570272	11.37773155954732	5.23209182786163
H	6.82921522920954	12.37571498137625	5.65593831455745
H	7.28100858741864	10.65868547183688	5.75045903021226
H	6.90524727284567	11.39321354330920	4.16777114713480
Ti	-3.03376076788517	10.42393204860992	12.08412331962204
N	-1.20211352482121	10.51662749040945	11.99105636359509
N	-3.95474352859987	8.44275115717395	11.83750523174562
N	-5.26604982224420	8.70277069571850	11.60574215598928
N	-4.12592597135367	10.25097356521234	14.00188466188579
N	-5.41969367531927	9.91842559138230	13.80337806608179
N	-2.33723716458852	10.51456424344232	10.05261096389679
C	-5.83426035941334	7.66098757590231	10.95234867203749
C	-4.85796903988769	6.69115657267373	10.79464494329583
H	-4.98869508579117	5.71829447597431	10.32666555596156
C	-3.69054063357804	7.21238104211050	11.38956937134795
C	-7.26217029511352	7.63277059648958	10.53334610536083
H	-7.94616729548585	7.59125619580175	11.39885987543602
H	-7.44626017126904	6.74560923064388	9.90791038204305
H	-7.52662292655450	8.52651282907364	9.94319230460881
C	-2.37718567011930	6.48780095566240	11.60691866405305
C	-2.69724405900916	5.02327686807326	11.94569369392288
H	-3.36669870587009	4.95053803170220	12.82064292666222
H	-1.76296781474731	4.48362410690701	12.18010747237266
H	-3.17824463399401	4.50129241195375	11.10049410127925
C	-1.61508826875774	7.10360690006360	12.77970819452065
H	-1.32130228016762	8.14460976367404	12.56881612808996
H	-0.69071886670732	6.53061046585450	12.96974419801619
H	-2.22663922048515	7.09459220329766	13.69809961836626
C	-1.50104351863500	6.52158776534378	10.34902021410551
H	-2.04180756500511	6.12845690186044	9.47087369522707
H	-0.60447116278113	5.89732704990762	10.50700314180605
H	-1.16176637881573	7.54130830980312	10.10954448042976
C	-6.04909010039657	9.75104737134493	14.98743686851788
C	-5.11176738200746	9.98125080647250	15.98518742966502
H	-5.28658664307787	9.93416087970983	17.05718502645404
C	-3.91116187403250	10.29510321601669	15.31971679549667
C	-2.56095806147097	10.62313160344188	15.91922578724945

C	-2.70768036229692	10.81283770929258	17.43093004092629
H	-1.73023413441902	11.07214436218400	17.87291650886794
H	-3.41711962896083	11.62456291347454	17.66914223357636
C	-2.01083898831071	11.91414155564703	15.30145322330394
H	-1.75784314444796	11.77354853961405	14.23674735686280
H	-1.08169297237888	12.21549587322124	15.81580731299980
H	-2.73928738958355	12.73942694449516	15.38865498880218
C	-1.15761974271588	10.37880847601231	10.65134147903342
C	-2.82663069487419	10.34849120147553	8.69974746189276
C	-2.07515676335596	11.22592257734031	7.68934296612647
H	-1.02112422371769	10.90582964010348	7.65390608916108
C	-2.69018280698254	11.10271937822384	6.29361407587514
H	-2.12240274046621	11.74016130821374	5.59225159797910
C	-4.15403730202185	11.54794843706807	6.32743366674776
H	-4.59607603907864	11.47773326928899	5.31602198854030
H	-4.22323229310116	12.60710879919658	6.63869342253525
C	-4.92660883056076	10.66214351709506	7.30860884463893
H	-5.98045589129371	10.99168063170094	7.35772034138449
C	-4.85771521485350	9.20302359349223	6.85037001410063
H	-5.31767476930492	9.09179675613231	5.85026623301074
H	-5.42940963585643	8.56203439625640	7.54789914415474
C	-4.30253543097568	10.78803239714521	8.69778138840919
H	-4.37489070426363	11.82937242498119	9.05235533830342
H	-4.85553228656070	10.16236232426300	9.42051724261903
B	-6.00613348273011	9.82996984423835	12.38092463502150
H	-7.18221926652410	9.54654089033736	12.43634061192698
H	-2.09353802316688	12.27511522233318	8.03644064458489
C	-2.61082242993530	9.64183396916628	5.84128744417318
H	-3.01844282975335	9.53737433101221	4.81784717441294
H	-1.55541833791258	9.31951205872932	5.81022831499674
C	-3.39344673495265	8.75941512316793	6.81826674725744
H	-3.33088945519265	7.70453103581330	6.49461226096434
C	-2.78448342299280	8.88765240145784	8.21731286588651
H	-1.73638686821370	8.54972793142579	8.21086117074282
H	-3.34401723187965	8.26204916271953	8.93374013346418
N	-4.68959572092006	11.86875692794504	11.74896629098885
N	-5.87071332728253	11.21748801364360	11.71644935319859
C	-6.83845188871217	12.02750256110822	11.23266946131922
C	-6.24761343144648	13.25355204607899	10.95776376221702
H	-6.74606290395536	14.13791216491262	10.56508213481188
C	-4.89233519264461	13.11138959514837	11.30728019392202
C	-8.26047071151685	11.61472652415623	11.07087796764881
H	-8.73570068113624	11.39213667573850	12.04168037355669
H	-8.82790998638346	12.42721550534276	10.59124612033170
H	-8.35654877255455	10.71323418516776	10.44281749091599
C	-3.81101095749793	14.16428760046118	11.27468348032611
C	-4.26446961975706	15.34932872554042	12.14042915392548
H	-4.44263286751241	15.03042490110048	13.18218094057998
H	-3.48650392700465	16.13407137986761	12.14702486606893
H	-5.19773175654553	15.79692487509254	11.75665003669235
C	-2.51451141412924	13.59323838846285	11.83756685567808
H	-2.14840921827440	12.75816711909118	11.21700922137787
H	-1.72148715348485	14.35923955327773	11.85851027305716
H	-2.65895585316326	13.23804886145800	12.87124382258813
C	-3.59219218069183	14.64417623433630	9.83376393415284

H	-4.52650204969062	15.03307841842289	9.39277285040654
H	-2.84128401563016	15.45427137061317	9.81123901917166
H	-3.22900943196676	13.82175099153230	9.19567745420118
H	-3.06175222153264	9.88988303171097	17.92171720026890
C	-1.58356813968052	9.47029855714745	15.64934498294210
H	-1.39995063051745	9.34988570063989	14.56942666254095
H	-0.61093330546154	9.66931800826854	16.13363151144587
H	-1.97820751056169	8.51739449512579	16.04289230597904
C	-7.49106202792756	9.39999647554933	15.11303702183679
H	-8.13914882526795	10.16220741676780	14.64697373703558
H	-7.72080313251105	8.43383644813873	14.63157377683023
H	-7.76180603542174	9.32520799559398	16.17757251374039

3(κ^2) (S = 0)

Eh = -5351.396932286954

Ti	2.03251757932919	10.75193830008470	8.46087085064153
N	0.30202355822893	10.72705292366039	8.41954508753516
N	3.49453748872302	9.11245153874234	8.83204955327282
N	4.70437002496320	9.71715373294910	8.91765348155537
N	3.14841105881197	10.57765363480484	6.46499460704508
N	4.47361429522477	10.74540061368460	6.66162529080632
N	1.46140489034757	10.84907686863578	10.30783742283135
C	5.58134328237856	8.90826902143301	9.55763533626245
C	4.90805733757716	7.73790291985996	9.87431753096909
H	5.32661425271203	6.86948959128711	10.37911035124948
C	3.59918409164381	7.90158788164769	9.37956010020520
C	6.99824939927927	9.27565364696083	9.82971609665904
H	7.58759359697426	9.36230149062006	8.90058226665822
H	7.46439895855758	8.50295830735666	10.46035778200219
H	7.07281131143022	10.23993962017176	10.36022076983746
C	2.51560287220028	6.85260674333768	9.28108002943590
C	2.97755908555947	5.84477620373887	8.21321215112891
H	3.12461207468158	6.34817937491690	7.24154860290541
H	2.21622067092196	5.05450058353066	8.08268129376679
H	3.93015214801729	5.36396444635141	8.49670995050149
C	1.19333002677230	7.46530639588747	8.83628297846242
H	0.79651656967326	8.17890532758891	9.57767976214297
H	0.43738152084582	6.67607992807816	8.69227390049746
H	1.30691848519607	7.98602640327960	7.87315648384443
C	2.33835186979165	6.12204443602550	10.61638112119666
H	3.29322073620422	5.70720069979430	10.98385913357889
H	1.63470985800941	5.28033852789818	10.49221124779742
H	1.93281979087015	6.79428517473033	11.39020919255645
C	5.14848721476052	10.65382764244016	5.49839463638462
C	4.21071486403109	10.41386838562682	4.50388870225803
H	4.41327021889745	10.28401390332708	3.44410660119574
C	2.96373495390736	10.37202501189006	5.15438060235456
C	1.60870783689346	10.14259101799727	4.52489323440207
C	1.78567343636154	9.78214550585021	3.04730432667926
H	0.79983791132136	9.58626194583414	2.59189502183781
H	2.25844028723384	10.60406344350957	2.48215587797909
C	0.77187413334980	11.42435994405164	4.62700660516596
H	0.52457229863309	11.65294802748295	5.67559802638675
H	-0.17567531897351	11.30756397051301	4.07306093093061

H	1.31696384809664	12.28552037336065	4.20263213618175
C	0.16285995837569	10.60687172978832	9.82622086092979
C	1.99620291905470	10.92775944934639	11.64707197040549
C	1.24710660860817	11.93211722388259	12.54075014126736
H	0.19553423684364	11.61863656489298	12.61998785330325
C	1.89907577134075	12.00161006086434	13.92574025504159
H	1.32977046529722	12.71037077092271	14.55450258736771
C	3.34704918818819	12.48012955783243	13.79717864989803
H	3.81849149068246	12.55331579128004	14.79534034053781
H	3.37550084535895	13.49006788593645	13.34719059162609
C	4.11739539921650	11.49604893872931	12.91440362777966
H	5.15963842510019	11.84427000004263	12.78835370578091
C	4.11012506332905	10.10572431330302	13.55695200855453
H	4.60625306185014	10.13836834797630	14.54527425976328
H	4.67891120931593	9.39701060655730	12.92543368431627
C	3.44924264180041	11.42065879348529	11.54192352133203
H	3.45532828276354	12.41604427123374	11.06369888818297
H	4.01789289730745	10.73586434800913	10.89409663676827
B	5.02970035362529	10.97539190517560	8.07294418847582
H	6.23270084866825	11.09589677046078	8.03479327947576
H	1.27243876905885	12.92486449525983	12.06084998117531
C	1.87944178041111	10.61356579627168	14.57274236477412
H	2.31528325197880	10.65919490481577	15.58914869389436
H	0.83833327729052	10.26624185601549	14.67294403583159
C	2.66293509921232	9.62691676259404	13.70345380401095
H	2.64788965513112	8.62649245899680	14.17332364482345
C	2.00639220175596	9.54613132799407	12.32370531876503
H	0.96718243518145	9.18428489479951	12.40370448082115
H	2.56963922693010	8.85420559602370	11.67459963629224
N	3.12696394871832	12.61555851798182	8.40472391773310
N	4.43314891913836	12.30780377558520	8.59629576646863
C	5.11179863454558	13.40720081332273	8.98748843165581
C	4.20720710739498	14.46139933379522	9.02457773523171
H	4.43141217484821	15.49303377722790	9.28444994158258
C	2.96856098306322	13.92184561179903	8.63766407298198
C	6.56775803749547	13.41396931614614	9.29873849445534
H	7.18125343248214	13.17835818841556	8.41253763056992
H	6.86181607146908	14.41002232365245	9.66426692012431
H	6.81686146146852	12.67550570167874	10.07961256056416
C	1.64585238896490	14.63117103190055	8.46823148440287
C	1.88673157527868	16.13780957947421	8.34693313765984
H	2.54600629825437	16.37534771076936	7.49360342197453
H	0.92497619571603	16.65731555492946	8.19328257324148
H	2.34320753001632	16.55164115366473	9.26281290526586
C	0.93683096182808	14.12373270101664	7.20848513060142
H	0.65990632305627	13.06261434626388	7.32121866997405
H	0.00595985549293	14.69545142169802	7.04593396976638
H	1.57460329398386	14.23211113065042	6.31400442368649
C	0.75960702927943	14.35436416420418	9.68409086645611
H	1.22158362211490	14.74145673847260	10.60915400493710
H	-0.21902145716209	14.84569507118595	9.55219288583249
H	0.59081876734610	13.27336610100859	9.81118492170019
H	2.40436421573715	8.87618409293521	2.92330014427046
C	0.89483851766693	8.99050148881979	5.23863818334835
H	0.66516309181560	9.25457952530009	6.28326846908932

H	-0.06238413464211	8.76299330806295	4.73844022807509
H	1.51602565787744	8.07753372412444	5.23295750747616
C	6.62731541890923	10.79506906569523	5.38984676139489
H	6.97088051479544	11.77952193371850	5.75191126553425
H	7.15594226711039	10.02481593790454	5.97736939623350
H	6.93241907615264	10.69310493265009	4.33703226016849
Ti	-2.85361663852983	10.04366252097635	11.85294939265904
N	-1.12249725433966	10.06362242139299	11.89317076139642
N	-3.95511627316567	8.18417288915241	11.89895333555867
N	-5.25879986991179	8.49992021558895	11.70325971677027
N	-3.96965156155687	10.21054111239092	13.84999605793660
N	-5.29493988001690	10.04265087036383	13.65448480003856
N	-2.28337015790236	9.95187938023250	10.00540506352246
C	-5.93830329919246	7.40986617162317	11.28813749520845
C	-5.03729448605814	6.35298570677987	11.24030687425572
H	-5.26337828286994	5.32670735991044	10.96154130926825
C	-3.79960454795447	6.88148166924255	11.64532300243643
C	-7.39116765156360	7.41505641460643	10.96300107402627
H	-8.01220646412300	7.64295018285826	11.84593289691996
H	-7.68713345085571	6.42558738384296	10.58146883518283
H	-7.62792662091273	8.16477780793523	10.18891544961659
C	-2.48044525437051	6.16494516042502	11.81214591882366
C	-2.72959056686630	4.65891119851897	11.92562420739250
H	-3.39239047588568	4.42092389527268	12.77607995640496
H	-1.77099951202483	4.13356219237214	12.07924818841769
H	-3.18592796922782	4.25181318703960	11.00665475693440
C	-1.76923473917903	6.66192867714391	13.07479182130228
H	-1.48960533753873	7.72313374151195	12.96938419567530
H	-0.83969996724525	6.08664611647972	13.23240458606852
H	-2.40663169920219	6.54895167899199	13.96899572537851
C	-1.59203737010446	6.44285305283009	10.59793519371695
H	-2.05801579642978	6.06746417584272	9.67007948737836
H	-0.61839728741382	5.94071925410214	10.72570040872455
H	-1.41232142528822	7.52304930678889	10.47899070380139
C	-5.96788841401239	10.12631783127141	14.81947869928674
C	-5.02862357015687	10.36092735993881	15.81391242678940
H	-5.22941557515234	10.48392132304841	16.87486159741545
C	-3.78293664234711	10.40836562435020	15.16135103562012
C	-2.42671312070149	10.63727909804105	15.78814927343477
C	-2.60001861482840	10.98479028653838	17.26920196591290
H	-1.61334896084491	11.17895153802823	17.72353634818177
H	-3.22037157342266	11.88825448336687	17.40268400741617
C	-1.72042478944756	11.79840821646637	15.08112997660947
H	-1.48970736414701	11.54179905757446	14.03488220605442
H	-0.76430176907603	12.02896426902729	15.58192647749592
H	-2.34725360365435	12.70744537246786	15.09248644132091
C	-0.98391735799642	10.18878429257715	10.48694080261019
C	-2.82124923863962	9.88911252827553	8.66649115815886
C	-2.82265588458727	11.27686514141658	8.00203109777206
H	-1.78124516972104	11.63260670973214	7.92388783971989
C	-3.48126624351362	11.21223968491401	6.62234536478430
H	-3.45955135999980	12.21643402253082	6.16083727094509
C	-4.93172919407398	10.74284889001496	6.76673789212938
H	-5.42943799945334	10.72241208472607	5.77887644255904
H	-5.49437364080313	11.45025638258710	7.40518155230209

C	-4.94825184783215	9.34714523404362	7.39733711706359
H	-5.99283351547068	9.00547897637292	7.52207091374696
C	-4.18660156036972	8.36517319614310	6.50479128908972
H	-4.66000064534684	8.30452375009650	5.50670960413300
H	-4.22203463420235	7.35142353579563	6.94559582212657
C	-4.27769875559532	9.40598929953241	8.76946910138841
H	-4.84096874544148	10.08910788685417	9.42380990538513
H	-4.28999601247596	8.40639351420124	9.23860970514576
B	-5.85377702045698	9.82666738255155	12.24196801133769
H	-7.05684474017982	9.70677829920620	12.28127558594386
H	-3.38084334885777	11.96744548462399	8.65724413275148
C	-2.70598719646333	10.22735426711604	5.74368126733978
H	-3.14339270129247	10.19387304122402	4.72748784669959
H	-1.66239091240894	10.56778423488236	5.64530237604031
C	-2.73530233067881	8.83390409665706	6.37852512152620
H	-2.17218530942081	8.12647364580590	5.74279094718937
C	-2.08095738045831	8.88667835139662	7.76305663507531
H	-1.02728691447073	9.19310699193408	7.68469406017457
H	-2.11305947682575	7.89017113289357	8.23479131895618
N	-4.31281499512265	11.68640396885972	11.49090639057948
N	-5.52872150765111	11.09366309211451	11.41041946831270
C	-6.40757349700380	11.92233703371344	10.79910888814402
C	-5.72842319439295	13.09262151160151	10.49477548348496
H	-6.14633260032152	13.97381550762648	10.01214639006401
C	-4.41446409136404	12.90855117240582	10.96836922877221
C	-7.83278105526862	11.57473427696039	10.54449711706421
H	-8.40924555935542	11.48804982564984	11.48173928763536
H	-8.29842444206167	12.35874632609943	9.92758086159287
H	-7.92812157683773	10.61593257578284	10.00777035154883
C	-3.32113262173069	13.94650789005209	11.07275053991946
C	-3.74541925840083	14.91976892041240	12.18707967652048
H	-3.86372065915822	14.38380618481607	13.14521180086695
H	-2.97730447093844	15.70299325073483	12.32019334393156
H	-4.70460993114030	15.41212965848846	11.94957604529490
C	-1.99082537283015	13.31276964825296	11.45878528335702
H	-1.61567368688959	12.62556806979630	10.68232091180453
H	-1.22872455047113	14.09389587798153	11.61339113379633
H	-2.07988847645534	12.75638407769058	12.40442936216306
C	-3.17651772989055	14.71911891469728	9.75754996681972
H	-4.13530263029688	15.16346782991199	9.43797192113187
H	-2.45305742704528	15.54334766110020	9.88445112114810
H	-2.81125040893688	14.06565992092555	8.94816192589228
H	-3.06950707527610	10.15703129878159	17.82852624589476
C	-1.58566525335083	9.35937876521023	15.67219697391164
H	-1.34139665864949	9.14026947690888	14.62076623382100
H	-0.63650827102549	9.47469866509078	16.22368403905145
H	-2.12627618842427	8.49291623225158	16.09150839487425
C	-7.44645453270833	9.98351363191542	14.92965398222187
H	-7.97663513556046	10.75789715422954	14.34895003215227
H	-7.79021243452445	9.00168379632227	14.56077625955407
H	-7.74966920368954	10.07745807967432	15.98375972645689

3(κ^2) (S = 1)

Eh = -5351.413311770028

Ti	2.11201090703632	10.21576904094008	8.39050625917541
N	0.30580974866005	9.94683090114107	8.54063142452475
N	3.81568390157059	8.86821524304179	8.76443800630015
N	4.93866763166247	9.61651279922866	8.81331871614058
N	3.17474571846350	10.30951258110700	6.42463005495352
N	4.46384955350362	10.67575558784975	6.60256907016031
N	1.46450832113645	10.15205112876735	10.45709368906467
C	5.91996496326049	8.94260267944709	9.45215539057194
C	5.40461912510899	7.70135351375739	9.80271282661720
H	5.93736469229000	6.90506392385271	10.31802904187070
C	4.07536278019186	7.69319358332825	9.34150486599679
C	7.27796453206321	9.49977175909987	9.70195721393652
H	7.80838697285712	9.73624611037602	8.76407127402991
H	7.87982952887639	8.76773757363649	10.26233244222673
H	7.22856508829961	10.42692011385022	10.29866453969491
C	3.06050416576096	6.57782724838633	9.43490521443264
C	3.77425225851842	5.23800256785894	9.21683189898826
H	4.28026448927423	5.20855836317966	8.23601480100164
H	3.04140724736771	4.41237883113388	9.25262760096920
H	4.52982388561898	5.04650300586200	9.99820425676652
C	1.98368772277984	6.75224948099371	8.36486698286161
H	1.38124156313135	7.66502606463833	8.52904192235542
H	1.28002180266591	5.90217011266309	8.38993125033907
H	2.42913320910856	6.80284887919153	7.35766899425124
C	2.41454416038262	6.59127748531804	10.82547622457396
H	3.17849586713701	6.53986745585116	11.62114676771532
H	1.73698847046756	5.72638272610323	10.94329197420547
H	1.83170463996955	7.51541170032499	10.96578461882635
C	5.11729666352356	10.69744590692085	5.42019879814851
C	4.20254808464841	10.33663677097108	4.44182835898538
H	4.39886953031452	10.25286738574355	3.37604260695315
C	2.99001286827447	10.09864417600157	5.11709078478115
C	1.65835508287662	9.70544350898782	4.51413957350206
C	1.86611750711934	9.27984278402983	3.05721521647416
H	0.90512576480559	8.95542024583261	2.62216423309772
H	2.24650682639519	10.11239678378900	2.44045680718683
C	0.70989324881113	10.91174079565047	4.54450273918575
H	0.44039627439486	11.17874827603301	5.57778674236800
H	-0.22373656875230	10.68147627310705	4.00226214190406
H	1.17641735800739	11.79361899895064	4.07214741681568
C	0.27405196140491	10.17999256792819	9.87055496342130
C	2.00646551835611	10.57057467970368	11.74270826351610
C	1.65960628984141	12.02955445970558	12.07937940402722
H	0.56560824014539	12.12514723119760	12.17091151468756
C	2.34650421890392	12.47122424786264	13.37396157710660
H	2.06170337405578	13.51676759229579	13.59303408438601
C	3.86523310447557	12.37628471694936	13.20625975118538
H	4.37652944908033	12.71977583419177	14.12546600432074
H	4.19412166016737	13.03619475561818	12.38132512484166
C	4.24341420783231	10.92508456260988	12.89829743025957
H	5.33704895042250	10.84624996945716	12.75253947932399
C	3.80831856944785	10.02188465653233	14.05426153819425
H	4.31443255580753	10.32306127010478	14.99108624982958
H	4.10266173466584	8.97613893978511	13.84649811247295
C	3.53825863551271	10.47911802299195	11.61727868430711

H	3.86588775461328	11.11333144740915	10.77806034540532
H	3.81575528763253	9.43831785037863	11.37592317057623
B	5.03852586169317	10.93195276882924	8.00869878691731
H	6.20671495723438	11.23590368288489	7.92600080883306
H	1.99876481148069	12.66523530181185	11.24426562642136
C	1.89867205080259	11.56470758391429	14.52308266152455
H	2.36580349611511	11.89122564086494	15.47213746092401
H	0.80481644451009	11.63697501781202	14.65007409634278
C	2.28887991477386	10.11711040324116	14.21185080386117
H	1.96048817175479	9.46024044695004	15.03796506967517
C	1.59742371928724	9.66716480181661	12.92059799461607
H	0.50433693966163	9.71460998965784	13.02308568749549
H	1.87642818772617	8.62491637887818	12.69322797671701
N	2.93687850184487	12.26069606718450	8.47547792193974
N	4.27488726705307	12.12669217958957	8.64555882978073
C	4.80996202942440	13.29910620926800	9.05905718809562
C	3.77766733383784	14.22234982056192	9.12498796694623
H	3.86860036530637	15.26999376376745	9.40030090510650
C	2.61542738019967	13.53624549175163	8.72176697606854
C	6.25739697756164	13.49042797246142	9.34916231359043
H	6.87648992235594	13.40141773443347	8.43983981990180
H	6.41805321682560	14.49245667873428	9.77631858386778
H	6.62651464594434	12.74665212959653	10.07506252029922
C	1.24967570798433	14.12734704832822	8.42522955771152
C	1.38058523847197	15.64818086359690	8.29106545357160
H	2.11799290164936	15.92633114973742	7.51838333655892
H	0.40510293302747	16.07963542117458	8.00663738013970
H	1.68356699851515	16.11468460195311	9.24442026683116
C	0.75634113263214	13.54621307424955	7.09765252742416
H	0.62506105234013	12.45733133647407	7.18266723043572
H	-0.22209515005640	13.97899320731819	6.82588463017014
H	1.47255448427842	13.74285085189417	6.28160375607029
C	0.22248682495148	13.83098433142403	9.51970571868018
H	0.53461330647387	14.25033434083242	10.49008219457196
H	-0.74812000547090	14.28070299334514	9.24838961398977
H	0.06260325616849	12.75056466949303	9.64795145811393
H	2.57785271823396	8.43925665259059	2.97991776332372
C	1.03936780559151	8.53736062448610	5.28790031703992
H	0.81624810230594	8.80576929410616	6.33469365092992
H	0.08534127478913	8.23707034737177	4.82004649947762
H	1.71363717745389	7.66376084097021	5.28512923485172
C	6.56003762016667	11.04036227878995	5.28005999510415
H	6.77967769462844	12.05210969361868	5.66230977638720
H	7.20373555876921	10.33277775903396	5.83086098242990
H	6.84579393555197	11.00673556170709	4.21731794710991
Ti	-2.93775291868103	10.41397338777803	12.00669957800667
N	-1.10997212574341	10.49831279700094	11.90570057414177
N	-3.83126309950385	8.44248695471983	11.64864685528329
N	-5.15130677157540	8.68108076419496	11.44518217935481
N	-4.02926829091413	10.10693252161374	13.92445020024910
N	-5.31361061962907	9.75119021729995	13.70766509833977
N	-2.24306124363091	10.67492138429725	9.97685991507513
C	-5.70719336862998	7.64886978411612	10.76682673423487
C	-4.71253863528542	6.70710154875165	10.56143112015344
H	-4.83007127660326	5.74817533781501	10.06261685140717

C	-3.54731243707122	7.23390721914017	11.15556314576668
C	-7.13953150106284	7.60415747906084	10.36484901377518
H	-7.81220537147616	7.52753734054119	11.23674635738589
H	-7.31455364653234	6.73024129970012	9.71854376082060
H	-7.42764160820975	8.50760185931415	9.80097743682122
C	-2.21147277666644	6.53639393331988	11.31781849091658
C	-2.48019283662593	5.04033913795057	11.53919272927934
H	-3.14286888038547	4.87544026337395	12.40662369646640
H	-1.52678737109327	4.51630191670866	11.72770009411759
H	-2.94652876014945	4.57118962208662	10.65574600855043
C	-1.46177470770805	7.08370166940451	12.53344342498639
H	-1.18301718227509	8.14090917017547	12.39382515603530
H	-0.52889163925772	6.51300836633737	12.68599404626268
H	-2.07442536241341	7.00217799065475	13.44772942591395
C	-1.34138251440767	6.69568389919453	10.06621893983008
H	-1.86633127726186	6.34600460012020	9.16072642559465
H	-0.41990679714403	6.10105607926317	10.18089603906140
H	-1.04874909529649	7.74349846564363	9.90083199499695
C	-5.93232180162443	9.47807201067736	14.87707899303082
C	-4.99857547161489	9.66657772214014	15.88694943628471
H	-5.16680120168876	9.53826473870863	16.95338295931268
C	-3.81234791270226	10.06662882697590	15.24209646157253
C	-2.47679580203753	10.41572362700624	15.85989187661939
C	-2.62382797956233	10.50159923788515	17.38077214648167
H	-1.65592707603061	10.77308562565769	17.83634340781082
H	-3.36570533249494	11.26520684038957	17.67275340475942
C	-1.99613731033838	11.76888603253330	15.32273604655579
H	-1.72615915554985	11.69966872130394	14.25546688417128
H	-1.09112105614227	12.09387639637341	15.86444936661614
H	-2.77158924513108	12.54572805518207	15.44215937038438
C	-1.06669942287098	10.47099551327358	10.56081946934555
C	-2.79650026861692	10.55610238340402	8.63899486749309
C	-2.17619842665526	11.53460232637585	7.63076773468255
H	-1.10660493329503	11.30064444918684	7.54407335785601
C	-2.86549261465309	11.41535070272624	6.26822232394560
H	-2.38284808759088	12.11409695663530	5.56020694292096
C	-4.35155803182444	11.75653898787085	6.40256297928784
H	-4.85177665414787	11.69065489740065	5.41806380060810
H	-4.47291935358021	12.79648820285550	6.75980245478012
C	-4.99907779612868	10.78494918171220	7.39337793436939
H	-6.06638892318090	11.04202618696544	7.52452945772641
C	-4.86864225691084	9.35182464039063	6.87128372580803
H	-5.38529167387207	9.24831218667050	5.89832993267209
H	-5.35051078683120	8.64739175418424	7.57576116902428
C	-4.29472250271604	10.89592156231449	8.74497967403623
H	-4.40921998401049	11.91671653726263	9.14619114901201
H	-4.76488831447768	10.20701540082362	9.46690241679317
B	-5.90185149720293	9.75366365294067	12.28451890833460
H	-7.07509525096216	9.45653428682046	12.31933881150202
H	-2.27144797408521	12.56524866441798	8.02007351724029
C	-2.72289135040794	9.98148142625485	5.74755974600306
H	-3.18522311250032	9.89368442982639	4.74585127665236
H	-1.65485990137784	9.72714431993491	5.64346259342796
C	-3.38434196396633	9.01000950468266	6.72747163102340
H	-3.27496621020610	7.97521826167228	6.35393605297225

C	-2.68977413118907	9.12508801712962	8.08567732550381
H	-1.62316558414667	8.86744057244751	7.99394193817583
H	-3.15772929726752	8.43535346108830	8.80815695626070
N	-4.60909202787435	11.85122447315624	11.79225261578206
N	-5.78043968827687	11.18581321303497	11.71793851716143
C	-6.75982451809226	12.01258525899278	11.28896369110930
C	-6.18717026143722	13.26338440731228	11.09730332834907
H	-6.69814668617659	14.16412526888815	10.76226048412233
C	-4.82900838052776	13.11693287054079	11.43436316232362
C	-8.17309922109050	11.58588784854978	11.08977410220578
H	-8.64698436605390	11.27813912775974	12.03763936260162
H	-8.75550871627461	12.42124960784031	10.67123800282169
H	-8.24728935132720	10.73407892826338	10.39280404872200
C	-3.75536541825400	14.17772665833115	11.46369065586272
C	-4.14214881961600	15.22874058177413	12.51444890571669
H	-4.22275333675856	14.76987247628746	13.51544732280206
H	-3.37470976037787	16.02258381757299	12.55859492865297
H	-5.11130646913263	15.70117041185771	12.27695250076552
C	-2.41561666990572	13.55427287702428	11.83962861631550
H	-2.11162909608930	12.80137556373607	11.09315918485473
H	-1.62400675206188	14.32094995216036	11.88458596243538
H	-2.46770030120159	13.08093307847807	12.83432765178256
C	-3.65025759650183	14.84566248410309	10.08639460035286
H	-4.60915527465623	15.30239306848318	9.78526105213678
H	-2.88538406481158	15.64236431228424	10.10691746925765
H	-3.36311745703036	14.11261263151466	9.31365996951153
H	-2.93577617948365	9.53442159481946	17.81126044402354
C	-1.44468040023706	9.33398990274480	15.51378264258004
H	-1.28491502332103	9.27168350777767	14.42547754794106
H	-0.47159872239790	9.56732349170369	15.98173242203467
H	-1.77401532867491	8.34340497869039	15.87278445761493
C	-7.36121856660715	9.06980115189116	14.97703306132005
H	-8.03694255475622	9.84726034858166	14.58037589605048
H	-7.56160427842576	8.14162672834458	14.41448794766486
H	-7.62347661953862	8.89564345164146	16.03208433257007

3(κ[1]) (S = 0, broken symmetry 1,1)

Eh = -5351.405120392019

Ti	-0.18933431579749	7.73818371927114	22.03812686384273
N	0.64212947332588	8.13456189241434	23.52401956722118
N	-2.00129102099661	8.83320317697001	22.46293245340315
N	-3.08705655911609	8.06560448713706	22.66733854709856
N	-1.46052094269267	7.35560472912023	20.14003573883363
N	-2.71880778336818	6.96976872639389	20.45431431337499
N	1.40938091503835	8.70805321215353	25.69581448908164
C	-4.06200861708667	8.78889496129094	23.26227889279292
C	-3.57302329394933	10.07895458792331	23.42794443491277
H	-4.10423851144843	10.91875847858171	23.87118414846733
C	-2.26987359700597	10.06556449330453	22.89040157843832
C	-5.38935105340383	8.22138223635522	23.62833218307298
H	-5.96335403203772	7.91186686012619	22.73760303980150
H	-5.98008003892127	8.97478393454113	24.17217774151027
H	-5.28287840863463	7.33507487728955	24.27743575464677
C	-1.30368719906638	11.21350708078037	22.71269247028184

C	-2.07947538814061	12.40902016358740	22.14005870185779
H	-2.55446140849527	12.15115606684833	21.17715336502222
H	-1.39241156959978	13.25734482791212	21.97039949029966
H	-2.87102646421464	12.74914926288515	22.83005148691290
C	-0.19761302087937	10.83292573559274	21.72396491551856
H	0.49310619171407	10.07393604353062	22.14510690264594
H	0.43412542407292	11.70884099357721	21.49619759940143
H	-0.62994018187627	10.47472747121708	20.77211140118953
C	-0.69166866910061	11.60953642836412	24.06235885810498
H	-1.48100103180540	11.83098777409709	24.80177884257600
H	-0.06935728463983	12.51298109277145	23.94393202521389
H	-0.05672879294304	10.80704153924169	24.46862616497845
C	-3.48574398597074	6.93356834981877	19.34193369611383
C	-2.68346292287567	7.30059287591345	18.26990295487878
H	-2.98773592697052	7.36780340291671	17.22707961096695
C	-1.41526302245594	7.55683015779708	18.82297099943332
C	-0.16737115323462	8.01806482317645	18.10733176885000
C	-0.35965980373146	9.47401388811832	17.65618257431545
H	0.53954717328155	9.83649276767703	17.12601134130720
H	-1.22278112418561	9.56732286608107	16.97464678794218
C	0.06789649753825	7.13027055806805	16.87792834778144
H	0.20043500032562	6.07456106628190	17.17213844330033
H	0.97561109585563	7.45624612359824	16.33909103824722
H	-0.77989847923112	7.18256669460882	16.17365340860872
C	1.49326831931563	8.67065271531841	24.40163168133296
C	0.31137994315646	8.04182308896989	26.37233764533739
C	0.49194219859135	8.23222643395967	27.88672058159799
H	0.49244926576927	9.31576749184207	28.10662758203328
C	-0.61568163749077	7.52289821287873	28.67306008367613
H	-0.45744776957075	7.67993275741914	29.75601685334507
C	-0.58511166778558	6.02264885229443	28.36222894526144
H	-1.37324222232503	5.49973989535112	28.93668698732060
H	0.38429915504109	5.59018278642119	28.67392931489670
C	-0.78924786643302	5.81217445763705	26.85933926972025
H	-0.75935317324136	4.73258715966563	26.62253183921203
C	-2.14198620870001	6.39068252566484	26.43230633966763
H	-2.96499928194672	5.85989663951827	26.94868098714371
H	-2.28072634491421	6.24360025280982	25.34485554996930
C	0.32124516085371	6.52703399612098	26.09099681596456
H	1.31116531989948	6.12856424044723	26.38367274129176
H	0.19940776560077	6.37089456027267	25.00808464062358
B	-3.16583019266797	6.71816527758464	21.92263077788459
H	-4.31172424033421	6.32351607289713	21.93609886785210
H	1.48491107785679	7.84341117370246	28.17946622049607
C	-1.98093637994922	8.08783668735916	28.26710014118261
H	-2.78451655375402	7.58465679917973	28.83805186105685
H	-2.03376201939859	9.16539076868544	28.51193759742683
C	-2.18401272192688	7.88595196341361	26.76325999691016
H	-3.16031524693030	8.30580165024008	26.45675499122893
C	-1.07279760465387	8.60755065766960	25.99911371626895
H	-1.09080913341716	9.68559690314173	26.23950699250901
H	-1.22205507377394	8.50483747164097	24.91434782743813
N	-0.92041400794653	5.73508472085946	22.41663155989062
N	-2.25953186673020	5.63692007773335	22.53956633835382
C	-2.59527334679653	4.41930684097876	23.01956996107704

C	-1.41666754620922	3.70763280247276	23.19855064737533
H	-1.32823554826550	2.68661491170365	23.56435945430829
C	-0.38283509176910	4.56995494551123	22.78557490590398
C	-3.99960777206599	3.99697992406491	23.27774320168204
H	-4.59160390365006	3.94910457095527	22.34775603518872
H	-4.00725109653028	2.99843070131080	23.74142122972740
H	-4.51130136060400	4.69626811178229	23.96065982283767
C	1.08604640565275	4.24154764409548	22.63834176542696
C	1.19837336283302	3.05077646373364	21.67130452588920
H	0.75393236720878	3.29528254520652	20.69052133340416
H	2.26017598711721	2.78777719676633	21.51516655396397
H	0.67946589008559	2.15981067750368	22.06508143841221
C	1.86269744950048	5.42219911718043	22.05556873161147
H	1.82409290206031	6.31765364993158	22.70433744282042
H	2.92751936110047	5.16191339235257	21.92983161137534
H	1.47781831535217	5.67987135054125	21.05091150526401
C	1.67793033764420	3.84164209570343	23.99587062041211
H	1.12033057947510	2.99934626300593	24.44188695629545
H	2.72992999505295	3.52704929873429	23.87482190252825
H	1.64784655180611	4.68512120299529	24.70204880018302
H	-0.53752519197736	10.13544890122177	18.52141103735845
C	1.05427494649136	7.92354465397885	19.01647713147323
H	1.21845401749534	6.89034579561456	19.36574144545836
H	1.96108971233873	8.23764172937773	18.47283522995511
H	0.98667546928935	8.59088544330822	19.89627960113323
C	-4.92838096645332	6.56243221841916	19.34512030186720
H	-5.08761746268881	5.54018233189016	19.72890162989945
H	-5.52281135279203	7.24665734625829	19.97457447690270
H	-5.32400991696678	6.60862203095261	18.31870508020769
Ti	4.54581363774181	10.35467403690510	26.01467035163036
N	3.59574132574112	9.85287375620937	24.63445814322527
N	5.40640496705010	12.18563032527128	25.25357023731380
N	6.74778775346449	12.21753456575662	25.14824943808440
N	5.81579845176231	11.01057073426165	27.78052601108263
N	7.09267419809276	11.30537069999249	27.45645391979331
N	2.87270765786532	9.12268308914268	22.48011262230463
C	7.13482929339019	13.29521262137428	24.43271995047523
C	5.98345722761634	13.98860119701383	24.07943067571122
H	5.93849468426098	14.91263186881746	23.50740887065990
C	4.91414641952816	13.26032278408564	24.63585870249929
C	8.55866781105026	13.61306231863963	24.13304756417331
H	9.13015757348023	13.84117571167337	25.04948925750288
H	8.61246575565256	14.48906123547993	23.46819538447057
H	9.06402633603548	12.77036461732439	23.63105479169123
C	3.43900912157768	13.58708548990306	24.60839832446685
C	3.26814041268727	15.10668473096928	24.72889754482573
H	3.72400646068367	15.49009809851355	25.65866761728121
H	2.19474975913832	15.36641276807916	24.73619773170423
H	3.73280311643059	15.63485538850573	23.87881958596804
C	2.71025491403510	12.90931281925993	25.77424835350480
H	2.65537179018783	11.81057281466640	25.62592033915838
H	1.66290966330004	13.25431925899728	25.82199162656027
H	3.19013748065052	13.14983298838525	26.74003903524180
C	2.83764697826929	13.10744332590740	23.28218493498850
H	3.39482094892296	13.52387611262021	22.42517496509884

H	1.78522401256004	13.43069623241323	23.19623399731069
H	2.87009642264626	12.00807641167092	23.22321673318137
C	7.77117054736392	11.69105225491868	28.56062072350834
C	6.88753347511843	11.64195761898420	29.63245774078536
H	7.11214940689222	11.88901398453391	30.66861920955508
C	5.66447862937169	11.20680352778030	29.08839749949016
C	4.33962654865167	10.99512747480079	29.78164613236842
C	3.82399537968575	12.35069312273288	30.28686746517739
H	2.85667476101776	12.22684883595353	30.80625380887385
H	4.53620810985985	12.81172327597862	30.99258306270860
C	4.53501586179649	10.03818218690972	30.96504667907721
H	4.91154589076236	9.05897061592004	30.62146667039814
H	3.57783298273183	9.87436378454515	31.49149352278934
H	5.25906269146977	10.44377303253457	31.69227159499374
C	2.76168195266316	9.28296922499218	23.76182069514644
C	4.01908210299209	9.47951822549113	21.66985951885341
C	3.56191123837234	10.51117514992107	20.61816218357724
H	3.26141768771152	11.43899722754253	21.13217434681204
C	4.66982718418971	10.80444153973766	19.60342107138276
H	4.30555449440039	11.54860238214152	18.87168433247886
C	5.05104966520142	9.51221547662417	18.87507203697362
H	5.83884148238463	9.71454926661733	18.12524363971180
H	4.17646910963098	9.11666299264817	18.32487195917918
C	5.54159459940613	8.47883232696550	19.89373616440805
H	5.80476144477872	7.54030194840794	19.37258179393446
C	6.77228239474055	9.02870831017189	20.62109112670627
H	7.59056208692101	9.21735483612725	19.90004377787628
H	7.14361638394666	8.28718635052835	21.35297864680620
C	4.43280544526641	8.19920568457897	20.91072217529782
H	3.53882527655017	7.78397956879581	20.41277215708162
H	4.76804594158332	7.44726064093445	21.64617499987579
B	7.58981041944084	11.23543792623062	25.98536943376868
H	8.75675375179208	11.56162270883614	25.93473798226950
H	2.66322350961728	10.12186515148190	20.10903427174712
C	5.89823540472992	11.35691330474405	20.33321150945504
H	6.69725515223515	11.59839385309975	19.60661060048845
H	5.63854400829152	12.29578756473374	20.85707442935529
C	6.38911474106437	10.32035299918736	21.34594327282713
H	7.26852166758041	10.71782641100355	21.88626058316334
C	5.28057202469821	10.03486381382992	22.36409265622198
H	5.03467923053512	10.95550281664815	22.91310373574768
H	5.64136959743489	9.31322225327527	23.11434242304924
N	6.29652294413490	9.12391072397418	25.72456364766673
N	7.43935977767066	9.78991259245608	25.47619784747285
C	8.36534237138080	8.95151782240850	24.96295402320991
C	7.78643672962063	7.68902999037459	24.90058039608811
H	8.26498102558065	6.77757281947512	24.54900733567779
C	6.48316449230793	7.83986073943520	25.41350139289323
C	9.73610785275670	9.38502682567759	24.57397839503201
H	10.31458604135149	9.74486435111491	25.44227319308909
H	10.28044863218868	8.53970248065687	24.12530136287642
H	9.70661863801629	10.20426778282518	23.83494046812263
C	5.44039386997886	6.77823385639244	25.68102668729303
C	6.14740410299351	5.53559108074451	26.23991822593212
H	6.70031001832360	5.77289962337575	27.16580603602134

H	5.40367094571129	4.75287607812218	26.47181220922477
H	6.86302510818747	5.11215949197221	25.51426555010803
C	4.43162673714282	7.27432046424745	26.72133213147315
H	3.74564072071007	8.04243172054874	26.30684180502378
H	3.77242505500848	6.44563562435125	27.03371388890767
H	4.94810657714705	7.65276742358122	27.62166148134914
C	4.70991973330358	6.40268063615502	24.38643085020380
H	5.41950755407078	6.02365965037461	23.62984156158416
H	3.96300200341268	5.61459571758769	24.58519067603091
H	4.18196495283969	7.26841365716154	23.96023986209317
H	3.67807515541828	13.05148961820000	29.44603014139262
C	3.30981173529104	10.39785561209540	28.82452945116659
H	3.63546344751375	9.42375399082394	28.41951478858654
H	2.35567107045404	10.21409150054386	29.34459482835836
H	3.06939411332359	11.08441177942835	27.99116156511091
C	9.20899863413281	12.08110061646563	28.54873983241553
H	9.84719497167780	11.26932685511795	28.16031842897971
H	9.38616752110483	12.96898965220143	27.91758376247893
H	9.53803475733505	12.31584167379005	29.57284876120021

3(κ[1]) (S = 2)

Eh = -5351.344471676837

Ti	-0.21183629846401	7.93082442320506	21.79699161258340
N	0.78957868662333	8.07350101506707	23.23689162802307
N	-2.01511598625111	8.79045989854206	22.57938602772423
N	-3.03606909424863	7.92822968944259	22.74698234119148
N	-1.67465714883961	7.76672906572785	20.01257725626031
N	-2.85249123475509	7.20389692983759	20.36676031088963
N	1.28560464189900	8.19881551915367	25.58660889712087
C	-4.00065929191399	8.49824015599069	23.50377764552808
C	-3.57400319482746	9.78297366256201	23.81814343435104
H	-4.11369241795682	10.51433943447720	24.41637109834964
C	-2.31807387969938	9.93138796661574	23.19718147527680
C	-5.25664982569368	7.79372199034161	23.88084803574195
H	-5.89455021970940	7.58979252747941	23.00303923844465
H	-5.83230146466888	8.40962599392565	24.58883305511285
H	-5.03946517972698	6.82634388715777	24.36509614604115
C	-1.41742402784430	11.14220545796051	23.13051519552891
C	-2.28662515033476	12.37890580081166	22.86501418866132
H	-2.85079259095535	12.27876711668362	21.92101441481249
H	-1.64925571142426	13.27847455432953	22.79483840918223
H	-3.01252302472272	12.54507819775811	23.67957368829606
C	-0.41719706484042	10.98866301843089	21.98013631534709
H	0.35336048008663	10.22026485998602	22.20381787646878
H	0.15591181881591	11.92073499875968	21.83913220765209
H	-0.94311651902317	10.76609369727648	21.03440806237305
C	-0.66967456294108	11.32945095751518	24.45699540014418
H	-1.38063815888423	11.45039678858314	25.29299754487392
H	-0.03636312194163	12.23216342719911	24.40753482212948
H	-0.01941428010679	10.47118611220510	24.68487065965215
C	-3.71859433773492	7.24262688225566	19.33027526814080
C	-3.06669995122804	7.85004005834587	18.26553780948963
H	-3.47854647422279	8.03635592597090	17.27541471011138
C	-1.78138500643392	8.16497259315724	18.74422908211750

C	-0.66799351084111	8.86481810478774	18.00041068454069
C	-1.10995664392507	10.30267894366731	17.68810115542096
H	-0.31688670696820	10.84034867007949	17.13769228044180
H	-2.02387491389428	10.31224364359774	17.06964816810513
C	-0.39141577557564	8.11598367417381	16.68907563722448
H	-0.07641864838577	7.07646538350254	16.88686931333208
H	0.41201319797760	8.61900120297982	16.12174276229632
H	-1.28886431201183	8.08066192717720	16.04834270270194
C	1.47198732400456	8.47099222163010	24.33595702976296
C	0.22237427226058	7.36366351424966	26.09261528112241
C	0.42959449962631	7.22193214608435	27.61134389361269
H	0.46116921699114	8.23334555953821	28.05593217498621
C	-0.67273286870014	6.37525292880696	28.25220503668239
H	-0.49285512546236	6.29561575514881	29.33988186840217
C	-0.66486183280275	4.97657454756509	27.62663457926375
H	-1.44790707248274	4.34638333021334	28.08939229472376
H	0.30544207695998	4.48169593594189	27.81931114088438
C	-0.89933154094624	5.09558436557567	26.11848453347363
H	-0.88806489056341	4.09198252469765	25.65527415292304
C	-2.25562035855866	5.76029846566238	25.86204684802588
H	-3.07181314527897	5.14022501336095	26.28084657785298
H	-2.42472203538259	5.84615558818051	24.77404125952976
C	0.20579666483214	5.94740129264730	25.49021858696968
H	1.19164343739322	5.47926971311674	25.66374130935921
H	0.06009063994974	6.02275095042975	24.40213093038427
B	-3.11724205228914	6.70142627468095	21.81399984947368
H	-4.22675824835008	6.21980678780551	21.88367082481189
H	1.41823755558675	6.76330699785740	27.79130368275743
C	-2.03317703879937	7.03361669386930	28.00323336381944
H	-2.83836951772213	6.43591998452752	28.47143292883994
H	-2.05876542722848	8.03597151207169	28.46957340055324
C	-2.26795509302087	7.15396866675129	26.49550722956557
H	-3.24321809231494	7.64013455909603	26.30818036666586
C	-1.15993452157867	8.00277057208923	25.86819198079936
H	-1.15845755200035	9.01482085860973	26.30979763094829
H	-1.32416771931702	8.11401965122179	24.78683290988871
N	-0.77392456090305	5.86356182249535	21.91372253761701
N	-2.07553699524668	5.61747185999370	22.15001333520059
C	-2.25464225709900	4.30973994899096	22.43985791242032
C	-1.00707235601460	3.69936638937590	22.37675636951796
H	-0.79810902368782	2.64550931159142	22.54635900512136
C	-0.10068831909756	4.71645825386349	22.02322119380215
C	-3.58199843141891	3.71231352570307	22.75319435367032
H	-4.28135235770243	3.79561684728577	21.90372990720344
H	-3.46043457153104	2.64525315221625	22.99526980528568
H	-4.05065781034092	4.20922180799401	23.62012793471808
C	1.37493586454569	4.61548611127729	21.70639203026282
C	1.62364029698492	3.29832343463167	20.96137324175905
H	1.01142255160457	3.23379129673304	20.04484404032575
H	2.68751287520360	3.22046764982118	20.67622012821869
H	1.38350579306266	2.42744745557257	21.59499933404081
C	1.78391027797057	5.78237352289286	20.80515550181827
H	1.76184027892492	6.73454817106105	21.37565019117923
H	2.82926382621683	5.66610806610215	20.47102030529169
H	1.14062333156333	5.84526902892239	19.91033697538026

C	2.22723450075836	4.65185061576516	22.98135392295128
H	1.91809242653835	3.86524311209938	23.69055233759379
H	3.29132981640761	4.49238076395370	22.72939765780038
H	2.13389912489955	5.62528110919606	23.48627516730664
H	-1.32399430347263	10.86002841370698	18.61643250410323
C	0.61495973149190	8.89652134859255	18.82653239418607
H	0.95724201593175	7.87790802170438	19.07406513167394
H	1.42825097277932	9.38439315075571	18.26307829125078
H	0.49868431531327	9.47821268323794	19.75925814824211
C	-5.11005659131928	6.71546061894338	19.39758691988785
H	-5.12873409279456	5.63897032472046	19.63827424276169
H	-5.70529050625798	7.23605200374022	20.16725805330252
H	-5.60701527041992	6.85847506422800	18.42556405120618
Ti	4.48089090565312	10.69305758998719	26.23159577932801
N	3.35686402205231	9.95711696356853	24.90084015866942
N	5.33164233231291	12.33543721244045	25.09616442139360
N	6.64311776632764	12.21251164971995	24.81760458708693
N	5.98243755628817	11.50202765966315	27.73027282876025
N	7.21302411109783	11.68788168244582	27.20349925197934
N	2.78058022372854	9.76319192510561	22.67997057241357
C	6.99983036481131	13.09829166277703	23.86074254780395
C	5.86136330350952	13.82124798354423	23.52343583060690
H	5.79728365601490	14.61824108267601	22.78483838661151
C	4.83263733960238	13.31079162779377	24.33862545910509
C	8.38609318863456	13.21561857362962	23.32868023889888
H	9.09139581982142	13.57329620315331	24.09928357575599
H	8.40352048540074	13.92939588499219	22.49042854603822
H	8.76372365772395	12.24550291541736	22.96276676835058
C	3.39014057097985	13.74814119268184	24.43643147471401
C	3.34962500444761	15.27851438656616	24.54402196225361
H	3.90190623310391	15.63067245444148	25.43289959501864
H	2.30430717189670	15.62727460176915	24.62382380145349
H	3.79692749733266	15.75673860037208	23.65562615757239
C	2.73988752371779	13.14535031596286	25.68477667258519
H	2.62512076935924	12.04584358576772	25.58238356219568
H	1.72274278167019	13.55030585919859	25.82397057775452
H	3.32732901377431	13.39099597778479	26.58688408507579
C	2.62232323075845	13.30060269020851	23.18599580590047
H	3.08067012891608	13.72249886476027	22.27370906484953
H	1.57701704391424	13.65748944790330	23.23543031348533
H	2.61404194110195	12.20055672295054	23.09145211400498
C	8.05361227464909	12.18087121339176	28.14160389181614
C	7.33100271240802	12.30860876362770	29.32014398402504
H	7.70870745671797	12.67552504293062	30.27275221978008
C	6.02877847827792	11.86838188226783	29.01140762062100
C	4.84162664054644	11.75469695852281	29.94080372398835
C	4.69037210760943	13.06179494086581	30.72930400692314
H	3.83671505470276	12.99429149212330	31.42734135835548
H	5.59465398933592	13.27990681108042	31.32244888260308
C	5.08670082280818	10.59314025391151	30.91588035695327
H	5.19837982756792	9.64005928246415	30.37020182450005
H	4.24043347149215	10.49203713489579	31.61953368503598
H	6.00636439570711	10.75582322050631	31.50372023739077
C	2.62033736923633	9.40262865581778	24.02639124061448
C	4.00186508221188	9.43209728843884	21.98854191385569

C	3.65500025409477	9.21776106260237	20.50684884321074
H	3.13596362752157	10.11856020825773	20.13344302423566
C	4.91447738080220	8.93175525705661	19.68646605216157
H	4.63140165867618	8.77014526996773	18.63014104540749
C	5.60628820460220	7.67735453585761	20.23252142463661
H	6.50771458982916	7.44987606562093	19.63317388890576
H	4.93143283336925	6.80481698069031	20.14424531744519
C	5.98470859863091	7.90139738790228	21.69947015619255
H	6.48233776509942	7.00099430360446	22.10284463188325
C	6.93342475412741	9.09920221914441	21.80699484859339
H	7.86669469180455	8.89900088707927	21.24698077034342
H	7.20664060078080	9.25795523592427	22.86455292649540
C	4.72528656532090	8.18541927052684	22.52086035221355
H	4.03458253648612	7.32270528757941	22.48817690434470
H	4.99437634536007	8.35067907499963	23.57564041045169
B	7.52682967116550	11.32932857277464	25.72488365416004
H	8.69265109096838	11.56534073644252	25.48717932661247
H	2.94771499704591	8.37565662813341	20.42465849478167
C	5.86859409156114	10.12573922758216	19.78868623867609
H	6.77741248515956	9.93914702054570	19.18633992257949
H	5.38536713686117	11.03153390019595	19.37726611878243
C	6.24017468903102	10.34877220307369	21.25711636611197
H	6.91532613834634	11.21953031929371	21.34573733340259
C	4.97708581981031	10.62861919547778	22.07222385814814
H	4.46795195743833	11.53402011284715	21.70078038274945
H	5.21989474638488	10.79895464660962	23.13138670921668
N	6.10425864076861	9.30431468425955	25.97520686090528
N	7.26831686209281	9.82023856106158	25.53593513696797
C	8.15465429156091	8.82362150823826	25.29217857823152
C	7.52088817392539	7.62679086778320	25.59636289710309
H	7.95391612305412	6.63130112638631	25.52196508816492
C	6.23245889877861	7.98016829654478	26.05170189051096
C	9.54091944220912	9.06280375285189	24.80380077648197
H	10.15481355784193	9.59383431897976	25.55255028758783
H	10.02797124519604	8.10010578871016	24.58425327760467
H	9.54859016945908	9.66946297877227	23.88163467242232
C	5.15947154746951	7.10649230566221	26.66369205252152
C	5.84509189070581	6.07255334787878	27.56845462187530
H	6.44779744427898	6.56316334409355	28.35286961206565
H	5.08489622849933	5.43873817422048	28.05933521705251
H	6.51184564944295	5.40874596673060	26.99102886322792
C	4.21844559362165	7.96688163129904	27.51611492681687
H	3.52474832627493	8.55841199978574	26.87812202921876
H	3.56164128824799	7.32613664252895	28.12779998175215
H	4.79772158554885	8.60863301566333	28.20511361259263
C	4.34917395456806	6.37431267663059	25.58691215233832
H	5.00953186679722	5.79405766581491	24.91785660340085
H	3.63505017702959	5.67625078581706	26.05974537372881
H	3.76623126322693	7.08566434721270	24.98492943377497
H	4.51658623795566	13.91474737788005	30.05017256255542
C	3.54805255223737	11.49437463029947	29.16864447918989
H	3.56678409100084	10.52290511943902	28.63965767969872
H	2.69080779990283	11.44590940653397	29.86236031824509
H	3.34240712990874	12.29782861818520	28.44118658717615
C	9.48238097651080	12.50910436496492	27.87533799764909

H	10.05310536516509	11.62166747251674	27.55179471475755
H	9.58551071056512	13.27122373264471	27.08368067187605
H	9.94978515654947	12.90128716737301	28.79205180176131

3(κ[1]) (S = 0)

Eh = -5351.322607965408

Ti	-0.31050154149232	7.86088060012437	21.91499734789046
N	0.63464951822894	8.07761648911936	23.35679373384991
N	-2.08542388975402	8.84035717828253	22.53579754457992
N	-3.18289931962763	8.08712952265747	22.72578422395745
N	-1.71343191026848	7.57398249995199	20.08303439219951
N	-2.91893526676984	7.07785549320363	20.45726796968848
N	1.48441871697375	8.44105478503504	25.54754717000337
C	-4.13940928318996	8.81558654786309	23.34373621561021
C	-3.62388867501068	10.09230388892929	23.54143089642437
H	-4.13722800829774	10.92981817737448	24.00949359534825
C	-2.32644181051788	10.06825512535468	22.99278610791315
C	-5.47649013060705	8.26560495214113	23.69969532235390
H	-6.05649674519865	7.98632729844033	22.80295641922303
H	-6.05261685110022	9.01773381481163	24.26062713912454
H	-5.38541579867734	7.36323689788812	24.32867023260706
C	-1.32917660343399	11.18610801386539	22.80515204099160
C	-2.08078191722344	12.42132809408184	22.28979857013682
H	-2.60540829090068	12.20185759058042	21.34332780543253
H	-1.37111997343621	13.24852601511272	22.10933969291240
H	-2.82953330082320	12.77276862074895	23.02051216333397
C	-0.28016023762144	10.77834732473334	21.76217413733950
H	0.42504157130472	10.01325587598887	22.15693920532251
H	0.35914260464799	11.63775466451946	21.49625721657057
H	-0.76477856282660	10.42710290525049	20.83393704332047
C	-0.64055341069906	11.52610602993377	24.13142735683309
H	-1.38481838553850	11.73336466356628	24.91975404296068
H	-0.01022114508950	12.42137074776883	24.00897671359851
H	0.00041686675187	10.69865650866885	24.47307823464818
C	-3.73292009486867	6.97523327623303	19.38384153122867
C	-3.02239170236146	7.41786532943961	18.27693692769648
H	-3.38585551094175	7.46164141092114	17.25241126556480
C	-1.75544051119546	7.78728483492387	18.76571185258459
C	-0.61847958172072	8.40584215014100	17.98057033637772
C	-0.91446079329019	9.90164925047873	17.78549447601037
H	-0.11731674496294	10.37609846626474	17.18530859269018
H	-1.87553940928984	10.04973282969293	17.26367872187245
C	-0.52481543273585	7.73314845379634	16.60490800457188
H	-0.36269665568616	6.64585805429345	16.70508438520348
H	0.31887523298025	8.15733358243442	16.03223435101530
H	-1.44186966333400	7.88797964000106	16.01145653876484
C	1.54459683819060	8.47625848049814	24.25506838923101
C	0.36360252446737	7.83915809138211	26.24574949125499
C	0.62569227779270	7.96717647995424	27.75549956403110
H	0.72314348286971	9.03986140815378	28.00409463526125
C	-0.49899452237432	7.32435983204260	28.57267872652365
H	-0.27841630277515	7.43179134151381	29.65066668369015
C	-0.60471925444970	5.83806976267515	28.21523153677302
H	-1.40285276210658	5.35860897846687	28.81312510478633

H	0.34117051845610	5.32095275357257	28.46324467665882
C	-0.89866738591233	5.69550261362312	26.71897929357963
H	-0.96857588962863	4.62553687817666	26.44986759568680
C	-2.21887546278604	6.39492267199876	26.38060665373055
H	-3.05608854896423	5.91990461101001	26.92744936946803
H	-2.42606592368647	6.29224248543287	25.29888897894689
C	0.22875913766722	6.34126106810438	25.91252159769481
H	1.19182572109371	5.84389130245966	26.13096058030837
H	0.03220764499026	6.23404759651243	24.83407458997480
B	-3.27589518224123	6.76246334735899	21.94285833753220
H	-4.40699977225404	6.33174667253548	22.01131772731462
H	1.59651695915689	7.49499801659641	27.99261875754210
C	-1.82836323952931	8.01396378950518	28.24917655776649
H	-2.64569399645004	7.56266349027106	28.84324382280829
H	-1.77802759233058	9.08367777415571	28.52628896019161
C	-2.11656680387983	7.87732948303564	26.75167515837760
H	-3.06555944668245	8.38817928024870	26.50429215847748
C	-0.98351498929532	8.52619596742988	25.95348544808271
H	-0.90229353620326	9.59355066215916	26.22360555699268
H	-1.19029634680259	8.46656708732445	24.87482346610334
N	-0.98000500390779	5.87810805078668	22.22043391099309
N	-2.29120576612768	5.70251990949138	22.46621770134843
C	-2.50889026914704	4.45094601472435	22.92940486118694
C	-1.27809915831054	3.80460926757954	22.96736937585991
H	-1.09984504112052	2.77778049650174	23.27981585151043
C	-0.33997960613292	4.73848665852683	22.48425000775726
C	-3.85936862555681	3.94242947985193	23.29595816655485
H	-4.53426537742884	3.90456479298531	22.42327853865045
H	-3.77559127105734	2.92559782002630	23.70954855576655
H	-4.33700377483185	4.58354869117358	24.05688832235120
C	1.12199655905513	4.55763575282049	22.14760237372364
C	1.28105544743734	3.19835274229255	21.45151083021402
H	0.65566065112950	3.13857968197511	20.54348572695786
H	2.33499089841927	3.04391221093320	21.15982708656505
H	0.99276181635081	2.36822585620807	22.11931289148737
C	1.57623883601158	5.66388899801870	21.18848499203474
H	1.68009411841949	6.64916810958634	21.69782000412184
H	2.58951880130580	5.44665294779502	20.80714061091758
H	0.89896913819826	5.73218724025635	20.31979185812596
C	1.99745419656430	4.59104120949351	23.40266638297162
H	1.65565641259681	3.85560047438854	24.15180235267492
H	3.04297818148106	4.35559374236929	23.13668659274891
H	1.97873165459079	5.58892416608140	23.86418554813601
H	-0.97112204091463	10.42589457637615	18.75400103607736
C	0.72435822200057	8.23862180107017	18.68744935331177
H	1.00929685029866	7.17705077535238	18.76456868782361
H	1.51846381787335	8.75913651439673	18.12574581065225
H	0.73833187924376	8.66748522282577	19.71033638064087
C	-5.13276261155712	6.47019288408648	19.45203302040460
H	-5.17687633447089	5.43874509856865	19.84128400864684
H	-5.76105676156704	7.09640332209461	20.10856320418601
H	-5.57663010944721	6.47631155522886	18.44452640330083
Ti	4.54310693934263	10.22123979206129	25.89095165385088
N	3.67529337817901	9.62031695304960	24.50264211882499
N	5.31610612544291	12.05867134639764	25.14056394449668

N	6.65148945784699	12.14418098768704	25.00018548207500
N	5.77105585635078	10.99658996902943	27.68376642519406
N	7.04538604912845	11.28657561398211	27.32439598644148
N	2.98324458085104	8.86330639883766	22.35763714585472
C	6.97303606247519	13.26201080259679	24.31299850555720
C	5.78546329087462	13.92311233770989	24.01980914253316
H	5.68679236739394	14.86442974802961	23.48285609639692
C	4.76302141329070	13.13825944896047	24.58720269001891
C	8.37434052550629	13.63818045769829	23.97934562293667
H	8.96748194808088	13.85514201724398	24.88451344865897
H	8.37816995384327	14.53596442022027	23.34209284554810
H	8.88746889004345	12.82779997440344	23.43385288360937
C	3.28983398841528	13.44125771983077	24.72371203890551
C	3.15126159292136	14.87820547303065	25.25002157799441
H	3.67339921784943	15.00075573039021	26.21493194031660
H	2.08494445433696	15.12540701110796	25.39766874534241
H	3.57282167190149	15.61023885445238	24.53982222611309
C	2.64849581175344	12.48079956853801	25.73225377701673
H	2.60369572787341	11.44167679419805	25.34123216624512
H	1.60484854973338	12.77079537619636	25.94046866777062
H	3.19030661759512	12.50853008451540	26.69578702105665
C	2.58475928456648	13.33474028073972	23.36642468707461
H	3.12483666792421	13.90986540082030	22.59455594126212
H	1.56060905978264	13.74070407849060	23.43727173220515
H	2.52427853294041	12.28732298019449	23.03395007445937
C	7.77285459833902	11.61327548800417	28.41467798953498
C	6.92958498897730	11.54125403303534	29.51565198731717
H	7.20039723000140	11.74514564987730	30.54930359358718
C	5.67657505463250	11.15273934079173	29.00681770616495
C	4.37131410115362	11.02556796162824	29.76398494207400
C	3.70086619694132	12.40883187860906	29.80100118505649
H	2.75955353204235	12.36677562183544	30.37826293112267
H	4.36387018028386	13.15613513803163	30.27000255372310
C	4.65026160578323	10.56974634183474	31.20075435223751
H	5.19112090717852	9.60760459198302	31.21701351438389
H	3.69950404972999	10.43993455819108	31.74673018629794
H	5.25232156687925	11.31148036742582	31.75301107515021
C	2.83880733885490	9.03954302483373	23.63476906358985
C	4.19992914276070	9.25640704481857	21.67291591794944
C	4.04226012675343	8.91519524344556	20.18265543627290
H	3.16943222910747	9.46457002237793	19.78674163940757
C	5.30520779961419	9.27610690250834	19.39358843348370
H	5.15936485943341	9.01715263332630	18.32876942949841
C	6.50189621953737	8.49862283606015	19.95239103327195
H	7.41584305645898	8.74497284079660	19.37918604254488
H	6.33276267684207	7.41100598409478	19.84286940944251
C	6.68673125340260	8.84885340436782	21.43110915675695
H	7.53914162515543	8.28267645924386	21.84995114115613
C	6.94481278254083	10.35101540928206	21.58040852589983
H	7.87943647589394	10.63335750229049	21.05791531146059
H	7.07111047023577	10.60135784540247	22.64985184412366
C	5.41979466925248	8.47853523448977	22.20124716716039
H	5.21563176435916	7.39751238375960	22.09816422672110
H	5.54510361341489	8.69483285695535	23.27247200733338
B	7.53726183669628	11.20962970340584	25.84646414627568

H	8.68935962019962	11.58169653752111	25.77889647904130
H	3.81534279452188	7.83723723463804	20.08501552687151
C	5.58161072018506	10.77807161947653	19.52421573182847
H	6.48750179103295	11.04869806398329	18.94913385846756
H	4.74164555188789	11.35744737709059	19.09727443003846
C	5.76022586956812	11.13243396770072	21.00302666460465
H	5.94572466365335	12.21692826081293	21.11191236154824
C	4.48892460778333	10.76531324843928	21.77063851214387
H	3.62472111875868	11.31267807944582	21.35154939010692
H	4.58448911612695	11.04463250520401	22.83003376410331
N	6.32070588513165	9.06546614270504	25.70458125504996
N	7.44106094486657	9.74430348201545	25.39221073815286
C	8.40382174049839	8.88696889150165	24.98356400151572
C	7.87165393761379	7.60544366139654	25.05557967197205
H	8.38476316472197	6.67912718038868	24.80591315320713
C	6.55798328178365	7.76264663497505	25.54163566287581
C	9.75915282452893	9.33255500341316	24.55833122641673
H	10.31818931199491	9.79745183217165	25.38917986067233
H	10.33862337020945	8.46996043282620	24.19486154791839
H	9.69981176913631	10.07514488353717	23.74351781685411
C	5.55757322309409	6.71501432321677	25.96505894499413
C	6.31222978219763	5.62809071190128	26.74526584893543
H	6.83627043149854	6.05384210547266	27.61876496045194
H	5.60383779944081	4.86148372857326	27.10618555352353
H	7.06111443167546	5.12142637682767	26.11208907160345
C	4.50699317939427	7.34681040294317	26.88299275849839
H	3.79091425883774	7.99237505930257	26.32632988467664
H	3.87356512908187	6.56246482625718	27.33346085938349
H	4.99135923394820	7.90128859075541	27.70467404898704
C	4.86225354668155	6.07647040800207	24.76020554536063
H	5.59583750808496	5.65001267731011	24.05372824787564
H	4.20029764063963	5.26359235742563	25.10480348902029
H	4.24756799324296	6.81054457513147	24.21770802902285
H	3.46373562622840	12.75915913913472	28.78242717342035
C	3.42560438882511	10.01890991286410	29.10959223952759
H	3.83412114805777	8.99669060090364	29.14658164692333
H	2.45279202601535	10.01234852967233	29.63014883856854
H	3.20706099387243	10.25444485967860	28.04631606527672
C	9.21992076816486	11.96606623685906	28.36989727008610
H	9.82578856969375	11.14739593057812	27.94547707115833
H	9.40464608042324	12.86374436639339	27.75528939020354
H	9.58261896007020	12.16657272375105	29.38995548809821

3(κ^1) (S = 1)

Eh = -5351.405039828277

Ti	-0.18913403391409	7.73814679236954	22.03868810900880
N	0.64272058450455	8.13409651089608	23.52408340750861
N	-2.00105213993406	8.83337956939347	22.46415393082607
N	-3.08679193791800	8.06562737471715	22.66825418177275
N	-1.46050975473214	7.35647488755875	20.14067478093318
N	-2.71877047151951	6.97046789414610	20.45481589415235
N	1.40927062957524	8.70786928524967	25.69592947939677
C	-4.06182151857243	8.78865162752868	23.26340843726914
C	-3.57296510776955	10.07870075343029	23.42943890527779

H	-4.10427396788559	10.91834631716574	23.87285899867792
C	-2.26986891076838	10.06564866734521	22.89174026140430
C	-5.38918209280473	8.22100138672567	23.62919654875567
H	-5.96327121168459	7.91199473201317	22.73834798196130
H	-5.97982674220393	8.97415238816179	24.17348116294460
H	-5.28271925841707	7.33436069665670	24.27783878309285
C	-1.30412499790725	11.21395288935957	22.71388345430583
C	-2.08037381289950	12.40913034148573	22.14119601105157
H	-2.55552378934632	12.15095840367381	21.17845422395183
H	-1.39356358530406	13.25759797967778	21.97120840415521
H	-2.87183271647570	12.74921305193526	22.83131239612655
C	-0.19798150488207	10.83371809049923	21.72513813106018
H	0.49344288887681	10.07554890409173	22.14658106852660
H	0.43297907416893	11.71001215864048	21.49670714424677
H	-0.63021134984176	10.47449312257825	20.77363881116099
C	-0.69220781540218	11.61037869699242	24.06348402259011
H	-1.48161627471306	11.83163619881212	24.80287946580965
H	-0.07021231432960	12.51401769066324	23.94493488679154
H	-0.05697098046145	10.80819834991526	24.46988645127899
C	-3.48556733131406	6.93421536981068	19.34234285958094
C	-2.68312824027569	7.30106946713570	18.27037157270937
H	-2.98725289414266	7.36809069031457	17.22749417171557
C	-1.41494296151140	7.55719571501638	18.82353931241329
C	-0.16672947778518	8.01756395189294	18.10788229240201
C	-0.35820826113485	9.47353768227254	17.65645625767336
H	0.54122900983323	9.83544097786690	17.12628188027798
H	-1.22124431577117	9.56718261363352	16.97485852020305
C	0.06807824088973	7.12945788384608	16.87862879029381
H	0.19990565635501	6.07369778219494	17.17297562053740
H	0.97603902821800	7.45478382198974	16.33981380740607
H	-0.77962404356115	7.18224314662234	16.17427893620168
C	1.49380550914779	8.67060056195274	24.40183067947277
C	0.31112176351477	8.04137188004108	26.37185009807885
C	0.49088125440621	8.23162212357680	27.88635067311849
H	0.49108389348056	9.31513996858146	28.10637091970249
C	-0.61702922950208	7.52199721841660	28.67201852684391
H	-0.45940238835843	7.67891256568501	29.75508197670783
C	-0.58603438007691	6.02179153149589	28.36100934718610
H	-1.37436561837397	5.49867427101218	28.93500692927328
H	0.38329374600369	5.58945169700476	28.67314338446252
C	-0.78937996102809	5.81147084358275	26.85798867635642
H	-0.75918021466896	4.73191693006877	26.62105724753594
C	-2.14201246185709	6.38978392036786	26.43036284178915
H	-2.96518875498419	5.85880982311161	26.94628429232433
H	-2.28020302612871	6.24277946619644	25.34283222298095
C	0.32136130313761	6.52663313235208	26.09028048592329
H	1.31120237255215	6.12827732833327	26.38335692745322
H	0.20004950740020	6.37069338217376	25.00727700038565
B	-3.16576735346098	6.71848532418351	21.92307141957508
H	-4.31171845232619	6.32398966125768	21.93644765903885
H	1.48376744775473	7.84295623599048	28.17958011433420
C	-1.98216955764627	8.08675338039594	28.26541025771380
H	-2.78595714861197	7.58336997723861	28.83589355963753
H	-2.03530398884161	9.16427047800906	28.51034487886142
C	-2.18444339297937	7.88500507235723	26.76144211275998

H	-3.16066057581958	8.30472719036551	26.45448947839391
C	-1.07296352356533	8.60687474819451	25.99794546463898
H	-1.09133818474735	9.68488473992534	26.23844521847461
H	-1.22158273272254	8.50424960496045	24.91307485377229
N	-0.92049315654057	5.73494179116893	22.41649475792891
N	-2.25959886579131	5.63693936937466	22.53969144357265
C	-2.59540836037086	4.41937291338222	23.01974095378986
C	-1.41679999246210	3.70784172570152	23.19930345255752
H	-1.32845751843648	2.68685328861503	23.56521516586097
C	-0.38294881283812	4.56991968002357	22.78591376028647
C	-3.99982151980648	3.99684420779764	23.27718647323520
H	-4.59157641422111	3.94970159547911	22.34701099148672
H	-4.00760254193136	2.99795912014446	23.74013445225544
H	-4.51169585206680	4.69562960570926	23.96049129959999
C	1.08591857474624	4.24138932506750	22.63885139700684
C	1.19813273314216	3.05054222614851	21.67187402694024
H	0.75369631618217	3.29501886872602	20.69108090692550
H	2.25990891363938	2.78743092573798	21.51573130778076
H	0.67915203798170	2.15965367436110	22.06571842976245
C	1.86272240454276	5.42190832960377	22.05601690687421
H	1.82431258768107	6.31734968967713	22.70480773282058
H	2.92748443165234	5.16140106121795	21.93022999674343
H	1.47778053583919	5.67960494536502	21.05138908841084
C	1.67773138911864	3.84145095420666	23.99640688676748
H	1.11986321365165	2.99940905552872	24.44256511445703
H	2.72960171790468	3.52645923766589	23.87530818874585
H	1.64803356167157	4.68501526355532	24.70248974926199
H	-0.53576798746001	10.13520350562229	18.52156008390864
C	1.05482923923673	7.92258914873679	19.01710148680262
H	1.21840975313534	6.88943777214252	19.36679607245191
H	1.96186185647576	8.23592555690808	18.47337588421543
H	0.98764024473982	8.59036047298112	19.89660949641221
C	-4.92817628301653	6.56296422977222	19.34538516908811
H	-5.08738091909053	5.54071496873246	19.72918298350621
H	-5.52270662597372	7.24716724135654	19.97476836852680
H	-5.32371415646291	6.60909971215429	18.31893231469497
Ti	4.54575451194437	10.35412078435349	26.01605798944849
N	3.59601046207001	9.85312240592418	24.63573624877858
N	5.40661774048503	12.18573740147884	25.25549617932641
N	6.74788940865677	12.21590338413994	25.14822659186253
N	5.81634405161211	11.00950239980882	27.78137750023455
N	7.09286746347025	11.30543534894916	27.45713547050203
N	2.87350254855919	9.12367731082925	22.48081211269044
C	7.13499677085999	13.29046009276174	24.42805526291219
C	5.98381521439097	13.98366346585142	24.07372230993581
H	5.93903592052556	14.90569858229139	23.49844432899706
C	4.91459949226640	13.25860112873234	24.63451935003357
C	8.55871880925834	13.60574927834075	24.12514771767919
H	9.13206576616626	13.83545586990204	25.04002142742577
H	8.61249031190721	14.47994256558795	23.45792374123409
H	9.06211887988409	12.76112109214390	23.62441590161801
C	3.43983587522786	13.58704184376282	24.60846967362673
C	3.27101987630612	15.10681636914655	24.73006502226997
H	3.72779778443597	15.48889921072689	25.65993390373239
H	2.19797401481204	15.36798572230971	24.73799285969314

H	3.73613121544460	15.63498777310833	23.88023301173631
C	2.71123122989788	12.90951371902288	25.77452216136833
H	2.65651762176619	11.81073744234651	25.62649154411401
H	1.66393554854177	13.25466330618240	25.82236015039921
H	3.19126359222565	13.15031345815021	26.74017527738482
C	2.83682556742481	13.10893602121676	23.28247047020964
H	3.39352631104877	13.52550804836413	22.42521705765564
H	1.78458696161300	13.43310045104301	23.19770609535398
H	2.86841645644292	12.00957378486975	23.22262842619716
C	7.77046534546281	11.69419320361550	28.56079592253716
C	6.88658372016998	11.64578302053783	29.63246258668599
H	7.11058935837935	11.89496723574254	30.66824826692478
C	5.66427235447517	11.20799098974130	29.08881406666279
C	4.33956652442037	10.99519141855019	29.78201104871164
C	3.82317569116882	12.35007209203602	30.28823181749581
H	2.85599960153795	12.22527292387294	30.80764970040547
H	4.53517147762211	12.81102322598529	30.99421717680711
C	4.53560841729052	10.03751967726550	30.96473285416751
H	4.91265222196788	9.05876579957086	30.62041447170296
H	3.57858703214842	9.87279463621827	31.49119691663387
H	5.25955840088310	10.44303927040267	31.69209464047981
C	2.76208771258170	9.28332021479954	23.76254635071234
C	4.01976750678855	9.48114252347639	21.67065036616976
C	3.56189616556410	10.51215836567717	20.61862780706445
H	3.26077796515893	11.43989577577699	21.13239852244916
C	4.66957985876234	10.80595202384442	19.60380608625967
H	4.30474626051839	11.54959041891555	18.87181711404106
C	5.05180346639150	9.51378661157512	18.87587740658300
H	5.83940489929909	9.71648342086259	18.12594936650456
H	4.17750214417844	9.11735657662117	18.32585773621602
C	5.54315461069306	8.48113061198925	19.89487899621925
H	5.80708431378792	7.54264030712408	19.37403679686485
C	6.77338622246233	9.03219983250850	20.62210690616683
H	7.59154409201187	9.22125453021725	19.90102731342974
H	7.14524022144312	8.29117866056037	21.35423526089077
C	4.43452914766927	8.20094281655769	20.91188576660466
H	3.54092255645424	7.78492322367040	20.41391788783508
H	4.77030336107163	7.44945864441435	21.64756085436518
B	7.59003181212004	11.23450088553712	25.98617476435535
H	8.75697840726540	11.56074256520204	25.93525798625102
H	2.66349408931194	10.12203997143351	20.10963344357684
C	5.89752560808575	11.35962381379440	20.33345552447321
H	6.69638759225282	11.60148836447962	19.60680933884416
H	5.63706991683020	12.29843560031603	20.85704666670651
C	6.38916596540697	10.32377613354149	21.34652610157095
H	7.26823389789416	10.72217008277865	21.88673260808601
C	5.28082636933536	10.03770308627914	22.36473413476126
H	5.03418007498950	10.95831735746467	22.91343950177538
H	5.64216858399695	9.31650729114499	23.11513354745159
N	6.29662549183536	9.12293043399676	25.72596251599377
N	7.43962266230896	9.78871783674825	25.47771045691388
C	8.36515003011133	8.95037979263363	24.96357882501448
C	7.78571160519738	7.68817949271663	24.90025306552974
H	8.26388023871308	6.77678351299887	24.54800194521842
C	6.48270829725069	7.83905201042749	25.41381958942274

```
C     9.73606668320518    9.38354618717790   24.57476438996338
H    10.31410015100285    9.74443800795503   25.44290026847637
H    10.28065396727167    8.53778039578908   24.12723001524927
H     9.70685028156963   10.20197110690338   23.83479220719739
C     5.43979810215063    6.77746075776469   25.68094814076755
C     6.14658784512663    5.53475026948624   26.23996135794599
H     6.69907101760994    5.77196072001342   27.16613071777837
H     5.40276099095291    4.75198724037769   26.47142958501780
H     6.86255115879815    5.11143120536464   25.51457982789772
C     4.43065928146787    7.27338243493060   26.72094908320010
H     3.74487119895851    8.04156861613112   26.30628204243979
H     3.77135983661650    6.44462849209760   27.03292535319917
H     4.94681881220637    7.65164831653020   27.62153990977998
C     4.70981251421185    6.40217442161021   24.38600231396043
H     5.41967098106766    6.02338468316787   23.62954895595711
H     3.96285546810785    5.61399998479578   24.58422106457865
H     4.18200878676550    7.26802256088561   23.95987438080875
H     3.67673680900364   13.05139977378314   29.44792689337338
C     3.31001088764595   10.39799345695677   28.82457396662562
H     3.63606890563787    9.42422703193401   28.41904946032333
H     2.35595684893944   10.21337202096452   29.34447805401584
H     3.06912648079373   11.08503864510903   27.99172158975608
C     9.20769386293020   12.08641166856998   28.54854144094193
H     9.84716783788986   11.27498340510871   28.16150611705112
H     9.38360997126838   12.97357498312321   27.91601135532105
H     9.53621296447800   12.32327968471983   29.57232775347231
```

TS$^{I(\eta3)\rightarrow3(\kappa2)}$ (S = 0, broken symmetry singlet 1,1)

```
Eh = -5351.338611781184
Ti    2.36837234936298   10.17633346239056    8.13057102011988
N     0.37163048222357   10.05002412513476    8.32611753942114
N     4.11233569836531    8.90959460326597    8.55118482425507
N     5.24554892161501    9.63809754399421    8.57912267805523
N     3.46468751659533   10.37930782523219    6.22592397063314
N     4.72913403701444   10.81183061485116    6.43224171827163
N     1.78429412845202   10.04323535103920   10.20898050077761
C     6.26510906675462    8.89478980476590    9.06755139357100
C     5.75541746440378    7.63551738985967    9.35317069334637
H     6.30907434840259    6.79023223262854    9.75786947124208
C     4.39260514293575    7.68657261692300    8.99953497108410
C     7.65376146888442    9.40790720544365    9.23059078349921
H     8.11746283856971    9.65411826379433    8.25973870944143
H     8.27537766078011    8.64346490204276    9.72199671038658
H     7.68079076000319   10.32082456111725    9.84951087139322
C     3.37602937299265    6.57027592892459    9.02214421332410
C     3.81649793558669    5.49695232748355    8.01562870307242
H     3.86523812882593    5.91480586093503    6.99478614120208
H     3.09705093540593    4.65811271531555    8.01298075050652
H     4.81332243876691    5.09404199738008    8.26609160398158
C     2.00152763374389    7.10278509578817    8.62782777873287
H     1.64722852454818    7.83981115326904    9.36582120643717
H     1.25918141034323    6.28764759881480    8.58361966935345
H     2.02947864348747    7.56433264956288    7.62398965442335
C     3.30561112535896    5.95995590940191   10.42730874864412
```

H	4.28172182262309	5.54950375058176	10.73921891575339
H	2.56742257265710	5.13832528148841	10.45181024426463
H	3.00121292772317	6.71850174196801	11.16811590368629
C	5.37341421377863	10.95321835164106	5.25245236740554
C	4.47794272228395	10.60527280939788	4.25051868128570
H	4.67626003908138	10.60430718235089	3.18149969879055
C	3.28508563094910	10.24813440728429	4.90811535471890
C	1.97067433228302	9.81302709085484	4.29408357973535
C	2.20721318064966	9.37097433053772	2.84663812485548
H	1.26049979475008	9.01984064013914	2.40082639724326
H	2.57902472911939	10.20274479721292	2.22387400900247
C	0.98421253213697	10.99041320315441	4.30272899599247
H	0.75302140102083	11.30544624846487	5.33255847210398
H	0.03491410096809	10.70019342076038	3.81791335441152
H	1.39960585112742	11.85920328015900	3.76317307712367
C	0.68422127667325	10.12573897466427	9.53946581402682
C	2.19159341005946	10.27644196254378	11.57511203534252
C	1.85245558245127	11.69553104903250	12.05368289869164
H	0.76136134847473	11.83351826265063	12.00601513858238
C	2.34613813491319	11.90803952127967	13.48669285680623
H	2.07664646322685	12.92906108272095	13.81373519811851
C	3.86596929706292	11.73550608081501	13.53330028813937
H	4.24251146635040	11.90488496395671	14.55997294435235
H	4.34978945595881	12.48495490986049	12.87918373775810
C	4.22476751715590	10.32187693269221	13.06582609043962
H	5.32221738267343	10.19203452813884	13.07971997646217
C	3.57227387691935	9.29715147730463	14.00074156867408
H	3.94353155965181	9.43454759053188	15.03374928397550
H	3.84947095789453	8.27176976469127	13.69125911161524
C	3.71810237839449	10.10890970861543	11.63679405351859
H	4.18617460772022	10.83464486393365	10.94853913239319
H	3.99375795544067	9.10096184219421	11.28047139133990
B	5.29195551586335	11.00457462956227	7.86142079983004
H	6.44613303799511	11.36535612976286	7.79814186922991
H	2.33106033080544	12.42028744173840	11.37148878438411
C	1.67781289887720	10.88255014305332	14.40789453194692
H	1.99931075708302	11.04260986355852	15.45490939952092
H	0.58205857660521	11.00860490323400	14.37170754499556
C	2.05062842755940	9.46727241908120	13.95440080635010
H	1.56859551500919	8.72777819423501	14.62000746487099
C	1.55396881192250	9.25049672629455	12.52320407534469
H	0.46080140168195	9.36502715029486	12.47467799436114
H	1.81131483474237	8.23320895253140	12.17506967454879
N	3.13351789876051	12.20593763728450	8.38724891415352
N	4.47079490067969	12.11459476131503	8.57167500457114
C	4.94395152552251	13.26608498698319	9.10357357437313
C	3.86769290246718	14.13088613035407	9.23686457035437
H	3.90597193734115	15.15095353546768	9.61087144239543
C	2.74485880164650	13.43325262832547	8.74709030638826
C	6.37951739940557	13.49538408061048	9.42575812046988
H	6.99996318901718	13.56098264891053	8.51487768031211
H	6.48771421374036	14.43981377914142	9.98151842224397
H	6.79148506714131	12.68320719640063	10.04790825055644
C	1.34946644635676	13.97507812777195	8.49446270287471
C	1.43458954765607	15.49982799988721	8.35223702203085

H	2.14191113082949	15.79428619933346	7.55761027519915
H	0.43969692881461	15.90447728127779	8.09660533202808
H	1.75377533748991	15.97822812974770	9.29427391657155
C	0.81910978394774	13.38432739961440	7.18450497236937
H	0.68086263492304	12.29531529101541	7.27417056078927
H	-0.16636209661398	13.81884164127002	6.94132332376259
H	1.51023755996925	13.58329142684907	6.34749703669031
C	0.37180080531761	13.65415884717810	9.63022791090485
H	0.71457111097630	14.07849722551553	10.58895228333539
H	-0.61728512900951	14.08647888043509	9.39681119663418
H	0.23751983878042	12.57045439751385	9.76683306354788
H	2.93957083599244	8.54636462247077	2.79265710619251
C	1.36778417455929	8.64565437193181	5.08048627351475
H	1.08467680850272	8.94976223312747	6.10387112355632
H	0.44377397634232	8.29369128348197	4.58850238100150
H	2.07317200010904	7.79840723700638	5.14314600574718
C	6.79234698697957	11.39030911471702	5.13403443200160
H	6.95047753768475	12.39175299577861	5.57012988350148
H	7.47831157870561	10.69541600812238	5.64882893538144
H	7.07920959147469	11.43045003051767	4.07176752115994
Ti	-3.18615286995221	10.60370598454134	12.17936742213564
N	-1.24024765852779	10.69944389018332	12.00642997260270
N	-3.96540371616173	8.58680611278916	11.91800702666704
N	-5.29997003271843	8.70311287439556	11.72399909319961
N	-4.27566650329618	10.39341148529273	14.08835050472981
N	-5.54339704204885	9.97125464295333	13.88318686201212
N	-2.63160694880232	10.75118848372954	10.12437792490000
C	-5.78424486622953	7.57163939882657	11.16017411353893
C	-4.71951412344227	6.69454456401142	11.01613454064343
H	-4.76904523690681	5.68475155741873	10.61660292939510
C	-3.59063495383880	7.36354356632904	11.53098450588691
C	-7.21927451196064	7.37237704141077	10.81687123915800
H	-7.85152516643998	7.29569583694020	11.71869338195885
H	-7.33552187719477	6.44300851164052	10.23792478129928
H	-7.61043182782156	8.20518299909817	10.20861492352484
C	-2.20264822997728	6.79817375219025	11.77082899861822
C	-2.30799557238813	5.27196660113437	11.87969204529909
H	-3.01793535399590	4.96961345602187	12.66903621585718
H	-1.31826263986397	4.84877329775391	12.12485582491509
H	-2.63453068570592	4.81829047475469	10.92800293169247
C	-1.66010855715521	7.35358334889503	13.09114209708994
H	-1.50776834835024	8.44202576603127	13.02270277292199
H	-0.68011369597705	6.89983451048566	13.32084485559496
H	-2.35069138126395	7.14652596414673	13.92674317292369
C	-1.22453082522679	7.13317532771962	10.63930598304298
H	-1.57524107672792	6.73701393862243	9.67154228994066
H	-0.24043594186480	6.68292912379477	10.85946028272924
H	-1.07692328456579	8.21814869000269	10.52887107455962
C	-6.18056409921738	9.81061705011601	15.06393488467281
C	-5.27678565774865	10.13621945947287	16.06591026853546
H	-5.46784524785997	10.11896612080054	17.13606847570926
C	-4.08668432672871	10.50020727148522	15.40728701719121
C	-2.76787869411416	10.92033280139131	16.02187806412272
C	-2.99342995229154	11.32626184209404	17.48158351920594
H	-2.04306984536385	11.66578606787329	17.92853140341454

H	-3.72469412512721	12.14967491878564	17.56193051616073
C	-2.17328247027076	12.10788095160920	15.25977364434372
H	-1.89653368176399	11.82912016072455	14.22795377443545
H	-1.24703392660793	12.45060019000360	15.75371447350076
H	-2.88152120772429	12.95425606319102	15.22210026197740
C	-1.50823772071293	10.64247084037972	10.76511271124185
C	-3.04387481506292	10.55265599827155	8.75333264952223
C	-2.37708521623089	11.57662567869053	7.82392220499479
H	-1.28764755335593	11.43468493019582	7.87553776852767
C	-2.87275590707655	11.39536790045305	6.38764678687934
H	-2.36889588069535	12.13282565259743	5.73602187330496
C	-4.38910131839046	11.60652415150351	6.33897116161828
H	-4.76039736522369	11.49469734726896	5.30293172825791
H	-4.64002335233544	12.63410162351366	6.66317713057815
C	-5.07190023050151	10.58515071141574	7.25569126055928
H	-6.16548059629358	10.74452411585878	7.24029185801194
C	-4.74890193833848	9.17002473190008	6.76656738947550
H	-5.12598201962907	9.02741495999187	5.73606817859836
H	-5.25545087248925	8.42333394263702	7.40665971821001
C	-4.56501864403405	10.76094733012527	8.68968022216324
H	-4.81598425934632	11.76967732963727	9.06159155380438
H	-5.05493038510059	10.03536380065154	9.36304065507877
B	-6.11201889670931	9.80783204420253	12.45437495677769
H	-7.26923724334639	9.45632445150492	12.51215590067667
H	-2.61089083731137	12.59464000676118	8.18642559125365
C	-2.53584877643618	9.97811248962610	5.91200255322772
H	-2.85923059367523	9.84383937077795	4.86198469206742
H	-1.44358709198441	9.82236674363158	5.94737810547418
C	-3.23419836767247	8.95609261959427	6.81518214841136
H	-2.99131265730503	7.93356311970744	6.47258861209164
C	-2.73930547128012	9.13270244135472	8.25280452481633
H	-1.65201405389330	8.96730679373910	8.30167997543783
H	-3.23747476461605	8.40880467961422	8.92214292700904
N	-4.91329284489464	11.90534089000123	11.80130190077982
N	-6.05280815437015	11.18680729285642	11.76061636895177
C	-7.06303861332955	11.94431260583091	11.27578415342491
C	-6.54102416252179	13.20265065500638	11.00725293144811
H	-7.08550236739864	14.05727749157668	10.60989669121279
C	-5.18033034508229	13.13568471147859	11.36460205519889
C	-8.45482999552163	11.44470137185821	11.09828851928246
H	-8.92528780134067	11.18762661005231	12.06296781696229
H	-9.06790423089720	12.22114935484661	10.61512430761158
H	-8.48542904946784	10.54146466199085	10.46551561413730
C	-4.15212489411196	14.24147134955841	11.35415619992139
C	-4.57494644578115	15.30306315460390	12.38039288585221
H	-4.62165119428702	14.86938297408630	13.39473756687429
H	-3.84625972475645	16.13375888265363	12.39185925698467
H	-5.56876213439360	15.72093315367720	12.14281218118769
C	-2.78126558424294	13.68799500156218	11.73144532192409
H	-2.44099433251878	12.95500102152219	10.98288170539876
H	-2.02837216450570	14.49322971598177	11.77833393866082
H	-2.80668585066577	13.21534777726001	12.72984736536875
C	-4.08381150752204	14.87269403603993	9.95820219687642
H	-5.05755261464093	15.29713874326999	9.65790816279002
H	-3.33809420784749	15.68757483369619	9.94262047214907

H	-3.79026545649884	14.12284303474850	9.20425917936249
H	-3.36066862691421	10.47936512491727	18.08635946248458
C	-1.78167695591661	9.74370460606127	15.97692107837096
H	-1.55851466645262	9.45282918346813	14.93835977156284
H	-0.82865396285345	10.02217358669910	16.46113668619492
H	-2.19313208000244	8.86241862574546	16.49898626674572
C	-7.60048698748819	9.37716657572072	15.18287672909250
H	-8.28640022491456	10.08417584468616	14.68489182238034
H	-7.76492548526976	8.38452342652034	14.72940382697285
H	-7.88137623057141	9.31878732406554	16.24584314386905

TS$^{I(\eta3)\rightarrow3(\kappa2)}$ (S = 1)

Eh = -5351.335990221753

Ti	2.28598238645266	10.14341534620465	8.09554118839149
N	0.24754783995410	10.08014868094268	8.30734378513052
N	3.99403847328398	8.87715491907964	8.64307501431359
N	5.14179859232551	9.57096787384607	8.74492401296952
N	3.56683413398484	10.29324666248518	6.26452013579383
N	4.81173874434473	10.72824545699146	6.56085503848429
N	1.63993643092743	10.03728973473203	10.19811063364382
C	6.10382149664323	8.80117157757275	9.30495935425348
C	5.53781115214818	7.55912074628090	9.56140156028888
H	6.03691171079221	6.70030478016308	10.00684107604435
C	4.20417243281627	7.65023225570068	9.11371784086981
C	7.49164220924430	9.27708794085538	9.56149624760219
H	8.02418726670144	9.51568725498558	8.62468291694592
H	8.06020074588383	8.49520869117977	10.08874002136476
H	7.50084384598871	10.18675246251503	10.18600916234765
C	3.14486365616376	6.57587502879624	9.06402086153430
C	3.63457281173025	5.45588493915547	8.13447136830628
H	3.80067936139670	5.83911898103167	7.11239900507442
H	2.88498457675110	4.64523404535384	8.08345500479357
H	4.58461617604348	5.02294594517656	8.49336753758852
C	1.83890048105320	7.15584666750237	8.52461851604688
H	1.42755426971532	7.89594401284860	9.22875631284906
H	1.08112577011010	6.36643937578461	8.38500272510107
H	1.99291791444711	7.62963004897137	7.53568037500204
C	2.90818232663173	6.01355441214409	10.47097087818384
H	3.83195273791811	5.57929764234676	10.89177794548184
H	2.14001391010233	5.21944939830123	10.44404693661826
H	2.56077624025689	6.80835196409945	11.15251172791033
C	5.53845073761306	10.88306201979276	5.43198760714918
C	4.72063012328970	10.53504191948615	4.36599177017937
H	4.99137018212292	10.55151318604980	3.31299892426040
C	3.48797663874973	10.16528483752326	4.93716247935502
C	2.25403386045489	9.63378612619012	4.24315758513374
C	2.33084919160672	9.94226070145974	2.74555105447504
H	1.42172492140942	9.57490926007654	2.23818590926759
H	2.40939212780555	11.02839852549638	2.56440083635961
C	0.98965425004463	10.27539058189667	4.81866092104488
H	0.83747324394961	10.02475462975047	5.88425353832131
H	0.09985419626980	9.91155505325374	4.27558863810548
H	1.02228692381633	11.37421759685683	4.72838696780728
C	0.54863250921462	10.17809149615750	9.51650555538843

C	2.08418025460333	10.37599365085279	11.53188936287122
C	1.74888287199850	11.82390508571918	11.91560455560361
H	0.65563462079271	11.95086664984068	11.89745901683973
C	2.29149858631225	12.15124084871562	13.30824103011481
H	2.02064769322451	13.19159128167181	13.56656770759068
C	3.81375212365287	11.99772382152424	13.31238663425196
H	4.22575333742041	12.25519802380855	14.30683146033141
H	4.26314291714519	12.69452124902333	12.57982953109839
C	4.17098299987923	10.55416702510336	12.94802508390906
H	5.27006418377518	10.43573478698027	12.92878046362103
C	3.56671558754712	9.59825275299684	13.98116180254551
H	3.96974243927038	9.81628006753209	14.98836397859920
H	3.84582196411641	8.55562244635661	13.73875161266222
C	3.61380960173780	10.22359241898362	11.56218459570558
H	4.05320370289303	10.89311365750824	10.80541385523166
H	3.88502631154028	9.19113477145413	11.28141730814452
B	5.26626032994847	10.93005171515395	8.02495917899870
H	6.42828775323641	11.27212636459732	8.04866190408590
H	2.19636228815993	12.49804563822785	11.16526101308200
C	1.66944048752321	11.19280907371071	14.32721238990290
H	2.02110727526632	11.43675650323432	15.34840604256513
H	0.57257419260680	11.30534808399512	14.31307710083476
C	2.04315310008216	9.74992609886617	13.97202669053281
H	1.59362799769476	9.06023964288536	14.71019512643475
C	1.49732909966733	9.41848742266710	12.58063553720608
H	0.40225935791625	9.52071411993202	12.56845260396303
H	1.75472439767132	8.38013038743173	12.30157475548648
N	3.09304821977480	12.17050075351699	8.35702640071426
N	4.40977410112826	12.06466904337728	8.64915531942899
C	4.85868477733743	13.21862841606704	9.19639936870090
C	3.79065160660347	14.10273549256875	9.22611905988556
H	3.81545357884315	15.12811823342705	9.58694667874730
C	2.69902372617919	13.41082834774786	8.66084262828314
C	6.26456383731811	13.42489532681984	9.64214193310039
H	6.96625604145870	13.46727525007200	8.79054544744417
H	6.34182500016346	14.37342114060025	10.19629690525481
H	6.60114130548572	12.61208871345601	10.30769418314932
C	1.32961259336143	13.96358479114438	8.30905023162428
C	1.46097576726230	15.47349913234964	8.07013286452800
H	2.20952634448768	15.69617148258692	7.28993944725603
H	0.48924336728474	15.88443753849374	7.74418362225340
H	1.75328151424644	16.00730788848317	8.99084100730668
C	0.83033552851979	13.30296105177284	7.02032927381811
H	0.65622078605277	12.22503038707491	7.16706359101308
H	-0.13291490226331	13.74912365280799	6.71515811257848
H	1.55663307118346	13.43543089615627	6.19963594342558
C	0.30626869769568	13.74230045980451	9.42935141238831
H	0.63380390863080	14.21650153300510	10.37030082986786
H	-0.66182907335655	14.18806782548788	9.13794142621288
H	0.13762261790617	12.67266559180283	9.62875599200853
H	3.19809217994418	9.44984559251146	2.27301898429744
C	2.19306512701362	8.11092684238761	4.43629855539911
H	2.13702358836062	7.85262809242751	5.50697160647888
H	1.30189159257389	7.69173169573640	3.93523195128985
H	3.08997637829052	7.62353309494461	4.01628542157325

C	6.95407035049926	11.34568514456864	5.41979303265662
H	7.06183339548141	12.34505120175226	5.87573139702996
H	7.61406873136742	10.65809197473566	5.97661659936324
H	7.31562154825487	11.40182023651365	4.38127215636141
Ti	-3.16155940940242	10.51884130108099	12.04845155291665
N	-1.30039617165404	10.73917892588796	12.03983887268413
N	-3.94368473919292	8.51670978197947	11.89433564168666
N	-5.27785785383723	8.65866927014935	11.71703711526577
N	-4.14914127864446	10.29176752340999	14.03726195050699
N	-5.42984293416295	9.88568605692497	13.89256907808811
N	-2.69241289219490	10.59791105790698	10.18764692565266
C	-5.78236220209019	7.54730941645311	11.13784901872810
C	-4.72959434272046	6.65840974176198	10.95985586773218
H	-4.79753265192122	5.65859763678555	10.53882990565921
C	-3.58599904925490	7.29909908532655	11.47192419324088
C	-7.22401417427352	7.37562724590175	10.80958507177507
H	-7.84430442723142	7.29147256358113	11.71877906055697
H	-7.36248689257785	6.46003168697925	10.21422108164194
H	-7.60867720090597	8.22687527120823	10.22316590763780
C	-2.19516501308440	6.72625848168946	11.66067924299087
C	-2.29759261364294	5.19790165210647	11.72443894927687
H	-2.98120737573867	4.86928143984623	12.52651344476101
H	-1.30032149520154	4.76876662785318	11.92377858776124
H	-2.65396679002160	4.77427625178168	10.76955267776003
C	-1.61656649094903	7.24714797221151	12.97918480207859
H	-1.46358321568470	8.33633301254622	12.93339022580345
H	-0.63138820404481	6.78715702642582	13.16890227838530
H	-2.28294172676817	7.02259826788385	13.82976372606750
C	-1.25121451403305	7.10295720647518	10.51390502367881
H	-1.61972913382537	6.72587381106594	9.54534393770806
H	-0.25713796537828	6.66160050071938	10.70068022526466
H	-1.11978242298728	8.19221610159343	10.42458496744698
C	-6.01357706950326	9.72699635427324	15.09732250854024
C	-5.06254219980659	10.04858315752984	16.05781164596827
H	-5.20461541563694	10.03271785687118	17.13534678102282
C	-3.90110423469581	10.40350257129629	15.34807012385310
C	-2.56794545218961	10.84952059859843	15.90916993494896
C	-2.71948753569603	11.13759047378698	17.40541432589108
H	-1.75833301766312	11.49156707421725	17.81604000253139
H	-3.47886948153463	11.91683714903803	17.59268838138333
C	-2.09935959322498	12.12439650008333	15.19799065318417
H	-1.86128104503404	11.92421511793939	14.14007784733449
H	-1.17576662501036	12.50422537361819	15.66845447840493
H	-2.86732034354161	12.91658009640650	15.25179907909244
C	-1.45500489352149	10.63581552492082	10.74086196545140
C	-3.13611959102955	10.52296290384700	8.81144333204892
C	-2.49371098079565	11.61824642675134	7.95078161197672
H	-1.40201136212976	11.47793047799215	7.96345251195634
C	-3.02806086791458	11.54190892516429	6.51848923740581
H	-2.54181054742290	12.32746232578129	5.91200617518313
C	-4.54504209314044	11.75081718944793	6.52156402067035
H	-4.94048480166805	11.71911117501936	5.48904209318020
H	-4.79009118060862	12.74863453773417	6.93247180244486
C	-5.20329037252136	10.65939709537528	7.37200320614725
H	-6.29714228817995	10.81645796328550	7.39771175533338

```
C    -4.89030880004734    9.28568777216342    6.77261774000475
H    -5.29178979421352    9.21736138608262    5.74398579399736
H    -5.38100582920053    8.49307127776505    7.36875856265878
C    -4.65885897117180   10.72948039912460    8.80034787681353
H    -4.90132129689787   11.70876727543769    9.24691625385750
H    -5.13093780654646    9.95433657197508    9.42921615145572
B    -6.05183048402981    9.76518592647885   12.48793709759888
H    -7.21227404335643    9.43109135789014   12.57076783976281
H    -2.72557081490942   12.60525494130434    8.39229034824520
C    -2.70094699428107   10.16581486265820    5.92999745131711
H    -3.04661977069649   10.10983467371715    4.88025829273465
H    -1.60910525706808   10.01276974557978    5.93334667060692
C    -3.37436667275919    9.07511642281380    6.76864067958669
H    -3.13757828792927    8.08289690628822    6.34337245030086
C    -2.83932228677757    9.14460283118211    8.20105104208824
H    -1.74992784349926    8.98419778446595    8.20879670630784
H    -3.31866963297353    8.37097797804958    8.82734341473800
N    -4.85090807019825   11.89385576806421   11.86139434215005
N    -5.98907446378546   11.16481279456064   11.83206803380462
C    -7.01375340988601   11.92431462266905   11.38337270127642
C    -6.50747775328798   13.19029171486681   11.12641303178213
H    -7.06692095172232   14.04689536727651   10.75602298498730
C    -5.13856477979670   13.12976273075019   11.44939569032701
C    -8.40635719302656   11.42156137439886   11.22284738105574
H    -8.85956155503676   11.14848968011258   12.19121168505337
H    -9.02963911853460   12.20330934696017   10.76206530382208
H    -8.44412739888028   10.52840425562530   10.57649396167666
C    -4.13624800783321   14.25950716726125   11.41643425998682
C    -4.48210964645735   15.23336671141534   12.55377053124827
H    -4.41566014114322   14.72734124788191   13.53296630193701
H    -3.77660116799188   16.08358707393526   12.55529717258473
H    -5.50500389328612   15.63357750384289   12.44494496613257
C    -2.71649211735556   13.74045351252149   11.61235102377932
H    -2.42991121050765   13.04593586310325   10.80787118046507
H    -1.99446127116442   14.57455918245351   11.60246797578043
H    -2.60103239185851   13.23104106734482   12.58384124177481
C    -4.22809208973251   14.98560607593500   10.06838847236920
H    -5.23191804973277   15.41320118864107    9.90271480746687
H    -3.49776245897660   15.81311578477289   10.03262720639908
H    -4.00784210024527   14.29633305615659    9.23502300532134
H    -3.00742415810803   10.23176900336471   17.96636339447458
C    -1.52611801742965    9.73920286874018   15.71711213122331
H    -1.32966126876624    9.56300647683878   14.64843749467166
H    -0.57092868262026   10.02594830028812   16.19086647272334
H    -1.86814995326971    8.79245858338517   16.17055310120010
C    -7.42830249452873    9.29804383195735   15.27740052311719
H    -8.13226473649207   10.01383673175652   14.81898835221777
H    -7.61629265023951    8.31142216915862   14.82029170551649
H    -7.66103742078424    9.22840585245073   16.35110568944609
```

TS$^{I(\eta 1)\rightarrow 3(\kappa 1)}$ (S = 0, broken symmetry 1,1)

```
Eh = -5351.3517814873
Ti   -0.07813269933793    7.81220721085174   21.95390686600748
N     0.58140751305394    8.07218999286707   23.69471964104244
```

N	-1.93576620885204	8.86499655462868	22.41344942897866
N	-3.01175508363740	8.06570840583973	22.52101648779537
N	-1.25853308833169	7.51721690008753	19.99984844032412
N	-2.53804503977497	7.14455021772323	20.24138044157024
N	1.32624467492449	8.65458119155774	25.93190764512473
C	-4.00059840703169	8.69657282000007	23.19147142021412
C	-3.53345892766059	9.96543040960120	23.51089849627921
H	-4.08139080572457	10.74092447340921	24.04280189074490
C	-2.23006513343882	10.03564296776460	22.97904427448614
C	-5.32102050944244	8.06972208784535	23.47691609216574
H	-5.87918891753697	7.84812218009117	22.55067004405380
H	-5.93171163357079	8.74912064954519	24.09153406235011
H	-5.20627052302035	7.12024454472242	24.02788255513024
C	-1.29858104799614	11.22336450003491	22.92546571588726
C	-2.10470439539287	12.44016511745753	22.44659770380941
H	-2.55127389392948	12.25593711164846	21.45380737622345
H	-1.44475830904900	13.32331751432947	22.37106745844213
H	-2.92064768273883	12.68769533917829	23.14726331821658
C	-0.17024577508506	10.96289231017906	21.92526581663517
H	0.54907198627231	10.20712071577234	22.29492016405464
H	0.41897981494717	11.87964250299354	21.75592603024198
H	-0.58225347161565	10.64698618922061	20.95109036790716
C	-0.72485035394679	11.51807820553600	24.31665017216638
H	-1.53480938403034	11.63953151015675	25.05691198306589
H	-0.13952724551895	12.45275315208596	24.29415227507954
H	-0.06246701010816	10.70908806208959	24.66255781415251
C	-3.24401300622276	7.11653067528581	19.09163213288810
C	-2.37997569191179	7.47263326491639	18.06380355619149
H	-2.62653316505471	7.53707697516153	17.00656727199888
C	-1.14116365639894	7.71070793524078	18.68173271457461
C	0.15788751998758	8.12888762845373	18.03104486716359
C	0.29330171664262	9.65721614896342	18.08667438136236
H	1.23868417512811	9.98160153890198	17.61570015413136
H	-0.54361107669298	10.14435383608453	17.55696018545198
C	0.16972369213699	7.68243215248403	16.56585805454528
H	0.04638653141281	6.58904127563165	16.47927945026685
H	1.12967531649103	7.95945329806356	16.09567172541852
H	-0.63479532477307	8.16765905184797	15.98749682862570
C	1.20712365613163	8.50031546734757	24.67556702773178
C	0.26676541926745	8.01446122770152	26.72204602964685
C	0.52892120366393	8.27017030398668	28.20851349025136
H	0.54537830962574	9.36181037407850	28.38024653084906
C	-0.54568556237308	7.60299546872951	29.07418402408537
H	-0.33534678789765	7.80262698450280	30.14093660223261
C	-0.53914303137171	6.09121795212534	28.82418394910226
H	-1.29851934907377	5.59830505301553	29.46008700190925
H	0.44278133635426	5.66487111512205	29.10321582842669
C	-0.82435507841119	5.81764378639414	27.34413489742353
H	-0.81161304510898	4.72844312271926	27.15682321451848
C	-2.19552482235827	6.38791317273862	26.96754861152449
H	-2.99070543512647	5.89209754242935	27.55605033201426
H	-2.40269378175338	6.18883003585633	25.89902533232772
C	0.24852993041668	6.49350721631703	26.48910919058246
H	1.24918213637169	6.08655337054907	26.72728335153527
H	0.05824379522896	6.30351682011878	25.41847398528783

B	-3.05396867288900	6.79048345439987	21.66335278772829
H	-4.19993717924350	6.40211357753997	21.58804134744296
H	1.53228972134335	7.88471379203935	28.46846545332995
C	-1.92432347083649	8.16466682494912	28.70958915953756
H	-2.70283059499683	7.69618541960528	29.34098805664591
H	-1.95538497198882	9.25235662631124	28.90877717686021
C	-2.20715788644053	7.89757212047093	27.22819873234547
H	-3.19433894019254	8.31368654834034	26.95463894762617
C	-1.12648262123246	8.57118199790331	26.38024458208256
H	-1.12640719391155	9.66088400405661	26.56027062753092
H	-1.32962905609305	8.41667047391889	25.30609077034159
N	-0.84303283517067	5.78972053990414	22.23883019841917
N	-2.18401906678402	5.66399284698350	22.24068952974596
C	-2.54001475154733	4.41245319342466	22.60441208904865
C	-1.36899735550717	3.70489939216426	22.83722393826806
H	-1.29252638131549	2.66239542752099	23.14037089028913
C	-0.32007545637108	4.60815258031377	22.57533483367386
C	-3.95392007782763	3.95288113496627	22.69290763899051
H	-4.44723521244781	3.95599713454183	21.70557965940060
H	-3.98576621155176	2.92560265476759	23.08789774869384
H	-4.55132221286814	4.59695879767581	23.36017714889468
C	1.16144969251920	4.31210245321887	22.56774085492563
C	1.40399532565633	3.13345156095196	21.61103175276832
H	1.06447601082298	3.37816307008358	20.58921769456945
H	2.48187628724851	2.89405157934355	21.56933588816920
H	0.86522995346751	2.22754397059179	21.93836487025262
C	1.96471453851690	5.51608497559738	22.07675633630853
H	1.87439145414362	6.38571713016829	22.75215249119187
H	3.03865417926019	5.27031422479047	22.01986943846182
H	1.64998655725694	5.80395037660733	21.05647849889368
C	1.61301598650945	3.91656515447835	23.97929149958736
H	1.03437411479403	3.05399319651581	24.35383270432362
H	2.68110702263570	3.63477268162824	23.97567240176508
H	1.47580060945303	4.75250028640038	24.68329572192533
H	0.29302377005373	10.01922369753178	19.12719247835137
C	1.33480474733258	7.47351904049031	18.75194244135881
H	1.22955139199452	6.37548479891726	18.77460253985225
H	2.28356327666512	7.72270900666844	18.24791702995320
H	1.42769989327525	7.83627559722910	19.79185871478500
C	-4.68802330035561	6.75836083027436	19.01028214372666
H	-4.87594373446874	5.72779062183307	19.35719330943294
H	-5.30968514326848	7.43119135423681	19.62521785765299
H	-5.02775267662408	6.83517010595853	17.96567150689824
Ti	4.45888963143135	10.28985910690934	26.11345334747556
N	3.50836143391927	9.79648145948945	24.57347322743381
N	5.27398100603678	12.10338392175820	25.21658591978785
N	6.60730235675021	12.12138141163290	25.03161274602013
N	5.79821532372256	11.01296162082261	27.75127800456882
N	7.05586130830296	11.31465032559159	27.36092923855023
N	2.76753095633008	9.06908107997821	22.37253255702242
C	6.95552586528681	13.13596206975660	24.20976089980826
C	5.78788701988971	13.80627831800695	23.87043315518393
H	5.71115093283546	14.68365083820929	23.23150690305533
C	4.75173684432040	13.13145982365848	24.54668145751437
C	8.36017687871587	13.41943766812444	23.80367715368915

H	8.99159445429630	13.69024409575715	24.66763146883332
H	8.37850482685285	14.25700691966417	23.08918359302038
H	8.82592843165105	12.54521241593776	23.31663529305287
C	3.28506031116263	13.49080545177456	24.59390907987855
C	3.16517183690134	15.01240984170938	24.76136352659846
H	3.67144219568711	15.35455170051406	25.68100159492927
H	2.10143848789225	15.30392908003284	24.82113847907365
H	3.61198218848462	15.55009039340515	23.90786097256212
C	2.59353348034131	12.81091930124946	25.78035385609822
H	2.49400772130648	11.71866822116965	25.62075159222584
H	1.56518172556067	13.19417362782374	25.89461736811603
H	3.13415245134988	13.01785119400357	26.72183456931785
C	2.60611526939143	13.07182467136697	23.28398562987098
H	3.14497886117881	13.48265843180239	22.41225875691254
H	1.56922093271583	13.44970136962976	23.25287734356681
H	2.57841391968867	11.97479809196317	23.19051427059721
C	7.76925759664057	11.77026608221486	28.41411118712481
C	6.93073458036232	11.76077444057045	29.52290610723380
H	7.19198328062533	12.06651017580530	30.53445038688709
C	5.69620883007308	11.27601022437548	29.05373159989772
C	4.41140245456026	11.07928003011467	29.82230022575225
C	3.92879982629885	12.44482452246944	30.33271552759367
H	2.99452651975318	12.33265892590621	30.91188927054373
H	4.68248333295913	12.91715060404593	30.98575833578374
C	4.67557071288713	10.14327815876547	31.00979554332647
H	5.03348197281457	9.15857419958420	30.66185774031463
H	3.75070898256009	9.98789159179860	31.59369486611032
H	5.44024932529297	10.56167991867999	31.68637258700350
C	2.87593488073878	9.32416656773726	23.61699540882096
C	3.90197435782679	9.40150781117011	21.49724687211915
C	3.39677864245978	10.29601634536618	20.35547673559464
H	3.01363991303979	11.23980332414652	20.78355000090380
C	4.50996329307706	10.58290297213387	19.34447896157349
H	4.11128629814079	11.21950879219592	18.53322387859230
C	5.02405748551679	9.26370840431288	18.75843638859429
H	5.82109429536686	9.46312421684022	18.01780813796410
H	4.20915323211104	8.74456551850326	18.22094944480880
C	5.56140283286857	8.37415619932139	19.88485735169449
H	5.92155092926288	7.41837889992288	19.46287217649961
C	6.71395917477999	9.09454446733324	20.59119177911698
H	7.53983378093999	9.28503041836351	19.87987543366991
H	7.11759565910149	8.46024022180155	21.40256062150495
C	4.44486801661986	8.09384747283654	20.89470742052961
H	3.61702063820357	7.54183073355960	20.41832690811383
H	4.82036765129246	7.45860645955600	21.71643251338335
B	7.49426507043226	11.18926840896471	25.87463171034957
H	8.65535859078480	11.52332501069428	25.76462023469260
H	2.54539129146557	9.80020925908244	19.86086695572682
C	5.66287397206301	11.30229869470165	20.05057299167708
H	6.46739638532942	11.53466219650669	19.32742634547217
H	5.31214759318514	12.26402684723602	20.46885145546510
C	6.19830159540688	10.41210870053571	21.17495964882226
H	7.01896964456001	10.93286855919639	21.70201657607057
C	5.07995567678641	10.12259006123404	22.17930130190505
H	4.73301258901635	11.06509872437971	22.63312655420749

```
H     5.47040546480652    9.50475124966174   23.00479004808223
N     6.19824843195072    9.07875038582686   25.72472063195028
N     7.33967393634836    9.72442924756385   25.42994626887894
C     8.24429602492241    8.86320207473108   24.91854452642158
C     7.65152951219160    7.60439104715075   24.90554832518042
H     8.11283458810793    6.67821390410742   24.56955330568416
C     6.36379166175464    7.78462744066415   25.44612391495703
C     9.60940109538930    9.27312562497042   24.48625736581801
H    10.21016926035322    9.65115365517404   25.33137529877819
H    10.13654025809131    8.41128648422309   24.04864778953656
H     9.57024793900558   10.07308589775760   23.72699110594482
C     5.30985677436623    6.75396514248938   25.77461303920596
C     6.00562802440032    5.51139548127955   26.34613097505888
H     6.58540469889781    5.75944414898955   27.25259216781022
H     5.25317314415415    4.74851471243159   26.61329484249588
H     6.69551007899313    5.05849796050197   25.61340416062298
C     4.34452563151908    7.30249621856673   26.83129842578548
H     3.62813028342540    8.04158332750191   26.41193082373100
H     3.70032554093093    6.49071489728710   27.21167539486024
H     4.89723620651108    7.72287840613958   27.69089788240059
C     4.52979964036955    6.36442962744794   24.51471936090151
H     5.20375862750343    5.94331709187831   23.74814560364351
H     3.76765906682117    5.60589442828379   24.75902290357157
H     4.01325816546491    7.23315985619641   24.08103908423504
H     3.73376695398381   13.13083552698260   29.48968427107342
C     3.32153278930125   10.46654074786107   28.94444309367525
H     3.60970063216667    9.47504828800056   28.55418060439645
H     2.39940458472420   10.31443985438333   29.52917390373839
H     3.04088284874843   11.12905576540000   28.10376575068279
C     9.19625426506467   12.18891191875047   28.32406695802946
H     9.83676985941752   11.37238401104200   27.94987548488418
H     9.32740698151100   13.04812322639131   27.64385584879087
H     9.56048557581554   12.48124584932512   29.32112745260503
```

TS$^{I(\eta1)\rightarrow3(\kappa1)}$ (S = 1)

```
Eh = -5351.344183904466
Ti   -0.08195597025857    7.91246057838318   21.89646971770235
N     0.70897271920800    8.35323681066640   23.51424462028641
N    -1.90370816661758    9.00020147696221   22.28134612192602
N    -2.97130493919740    8.25437385588203   22.61915882435581
N    -1.42346080525157    7.20682102644189   20.17848542240283
N    -2.63312452460495    6.78707822860125   20.62234233988145
N     1.54497087190808    8.93445280360643   25.71597343246172
C    -3.94908055252291    9.04028937862678   23.12218797098392
C    -3.48432809304386   10.34904707765080   23.08446179619705
H    -4.02415277302926   11.23631414775592   23.40964941424385
C    -2.19323242439908   10.27756037785462   22.52588684336567
C    -5.25410471189495    8.51201832140583   23.60692869167531
H    -5.83470815171246    8.04215522666104   22.79437639482179
H    -5.85454144708084    9.33299834687969   24.02833750821145
H    -5.11116026938896    7.75179244857296   24.39428730774279
C    -1.27637573364040   11.40836878494658   22.12947696557474
C    -2.03279268230890   12.29364321727905   21.12599129600857
H    -2.32579912019391   11.71646024396672   20.23154266770362
```

H	-1.39337266297164	13.13443338627394	20.80191162650642
H	-2.95018164204219	12.71463032754784	21.57233233239845
C	-0.00843130231120	10.87351052358931	21.46291876417179
H	0.62632178164793	10.29620159342135	22.16117585008691
H	0.61500106933589	11.70229406674500	21.08768634240457
H	-0.26411209170197	10.25106049586614	20.58273525667988
C	-0.90797822314973	12.24022038039585	23.36216791869933
H	-1.81217631972921	12.57221956325970	23.90147898308958
H	-0.34249131533131	13.13977255496948	23.06247659121174
H	-0.28820895690094	11.65350947564776	24.05812160131486
C	-3.40243282825758	6.39600388006039	19.58470242410120
C	-2.65761787631531	6.56983543619136	18.42495574569010
H	-2.98111873983352	6.34547204584876	17.41123001830242
C	-1.41920651097767	7.08220440906439	18.84707030549451
C	-0.24518631582385	7.50003692331653	17.98970142062007
C	-0.33497697550862	9.00776755022426	17.70988505208458
H	0.50626587779319	9.33445884981745	17.07236279896081
H	-1.27852857348284	9.25758973329702	17.19482994640288
C	-0.28132968310372	6.74486441408604	16.65768658106371
H	-0.26622906197074	5.65264602435893	16.81610474672302
H	0.59603062969179	7.01785749769414	16.04562542940659
H	-1.18348893752419	6.99569719860066	16.07430050620596
C	1.42096741430963	8.71616716668403	24.46402362008431
C	0.30050444502423	8.67033227597403	26.47026809262748
C	0.49594852125902	9.06040614162654	27.93370530669212
H	0.75845239196546	10.13196777092700	27.98603496753056
C	-0.77091614813482	8.78138425655986	28.74988975470976
H	-0.59330211337712	9.06522915814393	29.80366715294125
C	-1.12054728287051	7.29090070178297	28.67025616267368
H	-2.02640316991511	7.08226540265994	29.27015304030738
H	-0.30134675630238	6.68433936816774	29.09955758171128
C	-1.34509884632824	6.89910614826519	27.20665793715988
H	-1.58730776847285	5.82276007692499	27.13812197391786
C	-2.49545022784830	7.72322753656500	26.61956786653413
H	-3.43764661260986	7.50584432711853	27.15854149280579
H	-2.64600025002284	7.44802405763858	25.55881019858597
C	-0.07008022754764	7.17891611912631	26.41056989835247
H	0.77289756771294	6.58875689382949	26.81387529964935
H	-0.20788100957791	6.88060942907151	25.35733477925382
B	-3.02310721474539	6.79608760292193	22.12796986773849
H	-4.15200320620292	6.37255668623222	22.25876725176633
H	1.34749559525309	8.49562706715111	28.34353375010046
C	-1.93817404427090	9.59782721986733	28.18597302657050
H	-2.85436653298206	9.41038227544013	28.77719366254683
H	-1.71760594549470	10.67896162496467	28.26239483614266
C	-2.15857713873164	9.21402297488190	26.72035228079586
H	-2.98503154103369	9.81078504815826	26.29295087101691
C	-0.88086976879603	9.49408611960911	25.92856064130413
H	-0.62128123151165	10.56614367309604	25.99910542983283
H	-1.03756482411821	9.25785324459420	24.86376820600970
N	-0.73220414171557	5.99507143382003	22.64061707956374
N	-2.04888433576187	5.88796279853789	22.89290232331136
C	-2.28627562318588	4.82682907209507	23.69489205806393
C	-1.06041904714172	4.22431662431916	23.95072376420093
H	-0.89155367783953	3.33624040966003	24.55645006606173

C	-0.10628278988652	4.98750163810302	23.24916522373129
C	-3.64715696530882	4.44597331790575	24.16405330475233
H	-4.31082892620305	4.17619673231439	23.32444171029365
H	-3.57912826510539	3.58042520188952	24.84103960894561
H	-4.12914592086854	5.27261733365815	24.71424961254316
C	1.37743920326833	4.74455227148632	23.10738176104678
C	1.59160974847654	3.26009394708317	22.77668095561337
H	1.07513914688857	2.98031912473998	21.84173507797692
H	2.66955366701663	3.05210918778629	22.65396987538551
H	1.21299635672305	2.60787425676544	23.58237843871279
C	1.95971980396209	5.59260852438895	21.97382776487723
H	1.98820951996797	6.67274158238969	22.22610566377159
H	3.01188879158773	5.31507318927931	21.78974724797168
H	1.40337701287481	5.42986360886616	21.03461456477326
C	2.09644474353116	5.07573352095289	24.41968885646100
H	1.65731413116915	4.51945685443678	25.26619845339710
H	3.16127855863953	4.79810345273856	24.34193158470386
H	2.03711646702752	6.15164736265553	24.64613833572805
H	-0.29787264186050	9.58897762488635	18.64625074314671
C	1.07426415702084	7.18790345317718	18.69325742093294
H	1.16992943814797	6.11140135011109	18.91123318786175
H	1.92645311574860	7.49081418807272	18.06109655489080
H	1.17796668992444	7.74231893579849	19.64568565374457
C	-4.79275784132101	5.87976409332523	19.72841903699381
H	-4.82988877287047	4.96942254398632	20.35091099939681
H	-5.45815005798718	6.62546408701118	20.19598397824749
H	-5.19896544097017	5.63462185113958	18.73487840862413
Ti	4.56291884754871	9.93907658131621	26.04205942987651
N	3.88897157468870	9.56849942651188	24.29938903266966
N	5.16672253535968	12.00895124057414	25.71418747446498
N	6.47387657194729	12.28918809829620	25.88432803378116
N	5.64232575627695	10.29002918337114	28.03029854304708
N	6.88663205232624	10.79580658590219	27.86167387168057
N	2.96496589752385	9.02552247816429	22.10070434856854
C	6.72483835630123	13.58130054558356	25.58978769688432
C	5.51796776820721	14.15740519312957	25.21585543017120
H	5.36171790877950	15.19178647891290	24.91931438125232
C	4.55920168015090	13.13281688110233	25.31469525873147
C	8.07527703879032	14.20262583998008	25.68661664029788
H	8.45818436501333	14.19077899408939	26.72166549545220
H	8.02547342265238	15.25045067886159	25.35185903364177
H	8.81313635258675	13.67558948270322	25.05797084347788
C	3.08404533501818	13.21278410776968	25.00132814764047
C	2.59973017180061	14.65415061090200	25.19276005709805
H	2.77561361919086	15.00462047436760	26.22502934321223
H	1.51593575729660	14.71366356155867	24.98967165533669
H	3.10556182021312	15.35004331837370	24.50150566131784
C	2.28058442639279	12.29789387344710	25.93113641668914
H	2.42917951761818	11.22279454091818	25.71418894404611
H	1.19895757415203	12.46861903289418	25.79051347743478
H	2.51715098407181	12.49967328307176	26.99110643239958
C	2.87406240472838	12.79800773183090	23.53746189401011
H	3.44752130918470	13.45418510621067	22.85926170200284
H	1.80745267327467	12.86258716501477	23.26667352637142
H	3.20569905916891	11.76035318315351	23.38503452418741

C	7.54797718746071	10.82585259027827	29.03593844240863
C	6.69011873532640	10.32490794621215	30.00814559459296
H	6.90835875549826	10.21331969190311	31.06720820672246
C	5.50355327662166	10.00414980404593	29.33057287499158
C	4.21932562005672	9.45255257127756	29.90454505036144
C	3.11115332882329	10.49744452145677	29.72475654626166
H	2.15802039213862	10.13814422366774	30.15039448143357
H	3.38497232599736	11.44234057600123	30.22545570992662
C	4.39817333272836	9.16465282162753	31.39680581813776
H	5.19544994025293	8.42119602163975	31.57054053660528
H	3.46041279085728	8.76202158275840	31.81745006671104
H	4.65249672168844	10.08067473114903	31.95690483188369
C	3.20946329126924	9.22385627180954	23.32937662088012
C	3.97578056960970	9.37535706186551	21.09985286062271
C	3.34433774207346	10.36948020287493	20.11093982757765
H	3.06129180467342	11.28342483892888	20.66370905060526
C	4.31160295723120	10.70950518304246	18.97433519452525
H	3.82809026618365	11.42111018910844	18.28033194788992
C	4.68523044590010	9.42910934309377	18.22084002685552
H	5.37225558011329	9.66440375637112	17.38660610840506
H	3.77982373830805	8.97533961322211	17.77533986399963
C	5.34501034426459	8.44112581455622	19.18796223631330
H	5.60840964484422	7.51352736290326	18.64830043733942
C	6.61015898258400	9.07339394940998	19.77669949681572
H	7.33435575835537	9.30110011901689	18.97167490133389
H	7.10289377468901	8.36290186183986	20.46652293313236
C	4.37148701944518	8.10881490879645	20.32227685902258
H	3.45855212059965	7.63322215904845	19.92624862703375
H	4.82931938797950	7.38774225378841	21.02011647468524
B	7.42033997547881	11.23821737779525	26.47645878999369
H	8.51914424886839	11.73129289236893	26.61219133561608
H	2.41066887943100	9.93147524418051	19.71222027553802
C	5.57643080914070	11.34115820914300	19.56375822205384
H	6.28070880143723	11.61360261958815	18.75519165401264
H	5.31730341747706	12.27406468674686	20.09762576189782
C	6.23441236346655	10.35224917961885	20.53063265629530
H	7.14306597225611	10.80641548863914	20.96500487934147
C	5.25900934362661	10.01275412278895	21.66183533022262
H	5.00403186888080	10.92465672771810	22.22824961713756
H	5.73718942638519	9.32765844238074	22.38275243623505
N	6.53246391327168	9.09732381830159	25.59667731389813
N	7.53481464438128	9.99352055590511	25.58196304243386
C	8.61223641514842	9.48599099255975	24.94389876847188
C	8.28336992774066	8.19547242859966	24.55024961319055
H	8.93038263286888	7.49667697150737	24.02346746623581
C	6.96614293961042	7.98323576936502	25.00583314464812
C	9.88440769269413	10.23892229529439	24.76317005931957
H	10.37931358664944	10.44463563991729	25.72850259131139
H	10.57934697858135	9.65438163944770	24.14057281223500
H	9.71388934530328	11.21022018324715	24.26782256286674
C	6.15270937314745	6.71031301533669	24.94992619016654
C	7.06539393154756	5.53899334948580	25.34074389208438
H	7.48560000048357	5.68105435800476	26.35175264771970
H	6.49061746371176	4.59552081533589	25.33329842286521
H	7.90599990574801	5.42535067694508	24.63481008059159

C	4.99029300531079	6.77230427692927	25.94463233524199
H	4.20418801773442	7.47901232815916	25.61543473435042
H	4.49981885689390	5.78777946986435	26.02552953163540
H	5.35158463670214	7.05101890400886	26.94855944744008
C	5.62458805363452	6.48500919245138	23.52919132796366
H	6.44878191472056	6.50333611550394	22.79451839716445
H	5.12430980653096	5.50332323783733	23.45322037282903
H	4.89620953367845	7.26329799959688	23.25657500172147
H	2.94123269075378	10.71518722318913	28.65717558378517
C	3.85110648342644	8.14580068647704	29.19398566040217
H	4.68023482187189	7.41999595852344	29.25298523424219
H	2.95819147998329	7.69034989149155	29.65664541311411
H	3.61024289548933	8.31048083392865	28.12882529017275
C	8.94770117393533	11.31271483178775	29.19142843801508
H	9.65384788290364	10.72877526333940	28.57654419830501
H	9.04952334266116	12.37061606837717	28.89466492719425
H	9.25381618450538	11.22183594479077	30.24538377487590

I(η[1])-P (S = 0)

Eh = -2962.066692490796

N	1.53336116878336	13.92750151470417	5.28164235877863
N	0.78797046709306	13.06224524668406	6.00891022204537
N	3.67438116834782	12.69594055448782	6.94125597388719
N	2.59494378009293	12.03116513219620	7.41854820502638
N	2.74747941099733	11.26327566671362	4.43583658024268
N	1.81594410322105	10.85408404776864	5.33328466572082
N	4.51146125628415	15.41770644370526	1.26465332297986
C	-0.44814059886101	13.56449253762898	6.21441382002537
C	-0.50042518902358	14.80228640022408	5.58479870693051
H	-1.35331552382505	15.47590885007992	5.55475429314299
C	0.76780724261811	14.99933056896914	5.01620960710451
C	-1.50686318789793	12.86190545549502	6.99122928765688
H	-1.75195908001620	11.87887753483538	6.55287042544587
H	-2.42341646866987	13.47181763745439	6.99943433363966
H	-1.20167287541983	12.68785721490914	8.03744023095322
C	1.26735190863788	16.16266446586734	4.19562047710620
C	0.20402018257657	17.26142488901210	4.14734223900356
H	-0.73083404767855	16.89926245433529	3.68527748517977
H	0.57001699067515	18.11075213426166	3.54497987079280
H	-0.03211968769103	17.63834212823165	5.15787963942338
C	1.54256412711493	15.67110932208682	2.76857076182844
H	2.27736101274976	14.84797872895976	2.76280424645713
H	1.96358339579870	16.47968998399429	2.14712099906861
H	0.61590270153066	15.30160889954327	2.29515835529897
C	2.54723942221574	16.72916035070777	4.82269443753725
H	2.36296311399149	17.06407744429519	5.85824193975635
H	2.91735877596314	17.58727050244222	4.23473675077356
H	3.35851163825996	15.97774149045725	4.83240484642678
C	2.77144647679061	11.71576120701107	8.71367578121927
C	4.02298301416984	12.19442653817624	9.08955616021731
H	4.47743585176507	12.10907582192498	10.07309082372431
C	4.55803389079256	12.80826979142790	7.94924150853126
C	1.76593560496587	10.98394779416430	9.53297888728534
H	0.80813981470907	11.52857487085513	9.58736104497843

H	2.14709073401071	10.85633368535263	10.55776491738325
H	1.55401672296116	9.98373430256581	9.11768656821970
C	5.89572483819212	13.49119731515237	7.79774208772842
C	6.62995694858265	13.49302697298848	9.14088683318487
H	6.05559123684164	14.03217186166655	9.91425028723444
H	7.60533091564790	13.99743936192161	9.03104625862721
H	6.81957265508485	12.46688782871843	9.50078941865417
C	5.68048851987094	14.94213550276215	7.34958706782099
H	5.15897512349416	14.98723649752892	6.37812177600327
H	6.64970196438894	15.45460711701877	7.22269043708837
H	5.08416604574531	15.49654641105324	8.09517792590980
C	6.74197341381044	12.71926335593206	6.77576577413148
H	6.89902180591391	11.67822537062873	7.10798285408248
H	7.72732136632660	13.20053200216360	6.64806543308709
H	6.25850707901071	12.69365717106753	5.78461747876982
C	1.37179430904372	9.60897906500579	5.02388696742345
C	2.04242006334430	9.20866175746617	3.88227805556670
H	1.92433014339196	8.26039857555528	3.36435033605169
C	2.89998963827092	10.27183767369088	3.54067204792331
C	0.34852384832751	8.87526402697796	5.81760974414692
H	0.66879666317044	8.71678044713981	6.86249965685947
H	0.17209817908550	7.88811997226415	5.36262412213865
H	-0.61544515889508	9.41359334570238	5.84937588668966
C	3.84616344593687	10.36150379502660	2.36680675600310
C	3.74570017267240	9.08823859954913	1.52332694127194
H	4.02604193927564	8.19543249249776	2.10884034531065
H	4.43199789272422	9.15572345232473	0.66125539216586
H	2.72366966108714	8.93975326056779	1.13334004936489
C	5.29010264726277	10.51017886110280	2.86337039475790
H	5.98915231854231	10.56924506863822	2.00986816375063
H	5.57617788374179	9.64921284105123	3.49208707896383
H	5.42451949732517	11.43484519046130	3.45156189033794
C	3.45578814665188	11.55909126092090	1.49321631027225
H	2.41722974274247	11.46111422878246	1.13194791338447
H	4.12621934365569	11.63130322946442	0.61828532206634
H	3.53988678451352	12.51079766256321	2.04616834740775
C	4.89419382068168	14.88337696542297	2.27794120675044
C	4.52312614866818	15.26954285780941	-0.16153295324330
C	5.63456621150882	14.30958107962852	-0.61284762412605
H	6.60842010835001	14.69404421276446	-0.26027169362745
H	5.48266767864742	13.32753866880015	-0.13177073574335
C	5.62389011032124	14.17640131968152	-2.13796479550771
H	6.42555085266689	13.48218893488638	-2.44564273560548
C	4.26563689368799	13.63064867854720	-2.59239191725434
H	4.09263811177021	12.63109141713934	-2.15207574295180
H	4.25391064293382	13.50684481370459	-3.69093438766470
C	3.15473341823399	14.59366645446540	-2.16043776361262
H	2.17483499490046	14.19907007047659	-2.48292204730551
C	3.39292956319250	15.96733239777696	-2.79508372912661
H	3.36987219975537	15.88575651785935	-3.89754596455129
H	2.58513270882723	16.66452558496351	-2.50575113706198
C	4.74925636361473	16.51331018044585	-2.33593472994616
H	4.92116848530970	17.50739278019389	-2.78453548042417
C	4.75605688148784	16.64326169776293	-0.81032627619543
H	5.72115038670335	17.04758126880970	-0.45623467981105

H	3.96684316880776	17.33713761145881	-0.47014788964888
C	5.86063602011525	15.55168059639364	-2.77072094237898
H	5.87856604636988	15.46445495181446	-3.87265933526920
H	6.84595774246013	15.94646657453384	-2.46092605793286
C	3.16265797944115	14.72786911359780	-0.63541493257413
H	2.36498201899844	15.41289426904974	-0.29772484720215
H	2.97767763108471	13.74981477723049	-0.15847554265657
B	1.38051365464948	11.73236435422051	6.51203150989281
H	0.54360169270333	11.14749531444999	7.16041032820966
P	5.47339802805473	14.30427158630650	3.72808903606390
Ti	3.51301404323803	13.22279876291881	4.85819773253280

I(η^1)-P (S = 1)

Eh = -2962.116305321644

N	1.44279295420957	13.86066404554283	5.01304526321132
N	0.71527523620496	12.99407233843172	5.75569191460032
N	3.58865927636591	12.79326419088075	6.89246578922451
N	2.48632437017857	12.15304618891582	7.34691439270369
N	2.86152100792913	11.12399506601842	4.46404126580583
N	1.88440148498833	10.76592914193309	5.32917915880643
N	5.19001140842643	13.89445473487937	0.87173015651479
C	-0.54315041079740	13.45757003723995	5.91541893368817
C	-0.62349399076515	14.67316027371870	5.24803788036277
H	-1.49901207976113	15.31343753191369	5.17641959264515
C	0.64966102970494	14.89265889659568	4.69621541347196
C	-1.59978980663465	12.74382255729125	6.68586478953833
H	-1.78316273285485	11.73093836273299	6.28885911185383
H	-2.54189310522473	13.31064960331909	6.62896453514397
H	-1.32968004609528	12.63612723611210	7.75038950026013
C	1.13152745450368	16.07124831022880	3.88199809284147
C	-0.01468652309987	17.06533478796463	3.68456644683500
H	-0.86141735895356	16.60459316472827	3.14654643616400
H	0.33522601619076	17.92663666942624	3.08986764050465
H	-0.38523915633927	17.45053747744519	4.65048646594738
C	1.61216153507497	15.58375308873648	2.51078132711622
H	2.49520729665626	14.92851271224577	2.60869380989273
H	1.91342201890088	16.44077153167274	1.88278819548494
H	0.81502103529206	15.03093239408431	1.98377472632376
C	2.28134060758447	16.77027682095158	4.61990616705156
H	1.96483377642564	17.09571911206783	5.62608571695396
H	2.61780222061801	17.65690261156851	4.05384446281946
H	3.15696567159793	16.10496091789771	4.72096546059870
C	2.58182864953124	11.93882178348531	8.67689910122152
C	3.79738029485420	12.46577715506613	9.09241321242132
H	4.18919618617987	12.46415220631917	10.10634853483686
C	4.40091299096150	12.99288192507231	7.93812430299981
C	1.53544450654921	11.25120259639019	9.48373364016999
H	0.56564480979520	11.77505442073392	9.43163096395289
H	1.84807214446590	11.21625307619683	10.53866059822671
H	1.36936731128107	10.21492815955158	9.14243016269824
C	5.74043317391051	13.67809109049764	7.80446131336143
C	6.43642467455918	13.72126230442945	9.16673995558284
H	5.84032140760980	14.28295187796989	9.90693623825688
H	7.41534509677750	14.22179008265520	9.07150345162095

H	6.61212745950578	12.70578718294648	9.56204490870557
C	5.53098258331984	15.11534908395949	7.30997487944409
H	5.02307363692223	15.13840205167804	6.33106929465449
H	6.50057193530046	15.62797245781611	7.18647581256166
H	4.91828636176941	15.68979605108908	8.02655833443928
C	6.61761557091593	12.88911983668684	6.82275706728586
H	6.75137347583963	11.84914910456055	7.16814526402049
H	7.61190446084700	13.35986626954502	6.73032934431080
H	6.17761800138480	12.86510894448575	5.81056622118471
C	1.57432472389107	9.46052935764889	5.16931373608696
C	2.38560754331486	8.96452392589167	4.15719824657243
H	2.39376302410058	7.94671095688855	3.77605581267441
C	3.18206486935011	10.04404235734468	3.74017550625448
C	0.54386229407661	8.74694862960436	5.97433314636435
H	0.79725983631353	8.73544882033145	7.04829161444251
H	0.46699003548867	7.70366001596676	5.63126387423438
H	-0.45079512609659	9.21422702911098	5.87623728984605
C	4.22124130316548	10.08459925495571	2.64463667323860
C	4.46926094977185	8.67269524820170	2.10889378978012
H	4.83645036190772	7.99898584554536	2.90285123638257
H	5.23124194676190	8.70486002730797	1.31096551512313
H	3.55155788458420	8.23457923032714	1.67914391539276
C	5.53748757234268	10.65089250395097	3.18878361983639
H	6.30919595287434	10.65370977492649	2.39922446080332
H	5.90547588213769	10.05190342634140	4.03987191629821
H	5.42952693351256	11.70091971270144	3.51821724508623
C	3.69625074931687	10.96876169007800	1.50693149002516
H	2.76240285676377	10.55425345974117	1.08742422589419
H	4.44555064491108	11.05284456267128	0.70137041798728
H	3.49085411630678	11.99226962901421	1.86092425568131
C	5.21121881111113	14.19700611677544	2.05085940673216
C	4.63362101574055	14.49707447946080	-0.31529877461689
C	3.18992759036653	14.00953586948630	-0.52747365807030
H	3.18714748259868	12.90763522951938	-0.59914983948728
H	2.58470625139516	14.28203846384204	0.35344418828759
C	2.60364099959842	14.63328815512913	-1.79701326215109
H	1.56600780479851	14.27807984838875	-1.92835376981855
C	2.61301754688999	16.16006912816824	-1.66777982977816
H	1.99251768801815	16.47008732455684	-0.80673483254678
H	2.16792896513979	16.61985318772341	-2.56945450834004
C	4.05308102865391	16.64959680781225	-1.48008072308205
H	4.06336984913420	17.75008428459531	-1.38577675666749
C	4.90075980144756	16.22759830704544	-2.68500194694802
H	4.50384591335005	16.68642326196777	-3.60953365859997
H	5.93757968491183	16.59120894634127	-2.56345595576813
C	4.89032999914374	14.69981778705392	-2.80912181761580
H	5.50542222759927	14.39390395650023	-3.67369108120817
C	5.46874202781888	14.07969263820605	-1.53340590275840
H	5.47545427742933	12.97660479868027	-1.59944854433167
H	6.51403447644594	14.40185965526243	-1.37933119218676
C	3.44953328403435	14.21559581887816	-3.00418014460460
H	3.02646505268312	14.64392451397409	-3.93199566359618
H	3.43109750429578	13.11577535066863	-3.11686518269508
C	4.63598322030286	16.02923696632849	-0.20854522751153
H	5.67262203264745	16.37453812495280	-0.04483462487095

H	4.05248283477148	16.32989633237056	0.68030342968128
B	1.34347292295581	11.73835544647257	6.39754453908540
H	0.49132263196757	11.17019114929106	7.03898842490775
P	5.39900766743809	14.56581357981185	3.65254820843240
Ti	3.49757512990875	13.19542554650545	4.74162046380044

I(η^3)-P (S = 0)

Eh = -2962.138780651032

Ti	5.17907553714929	11.92150572195344	9.70256059065428
P	7.46111159719046	11.92154941099689	10.07045695219029
N	4.01293269257115	10.12667697127399	9.49251111099659
N	2.77180296642090	10.60474388790988	9.23389094823004
N	4.04073611654178	11.92151673647743	11.66730054962635
N	2.72322691418012	11.92142881683633	11.35489271835589
N	5.68291265140692	11.92163550449763	7.83253512146341
C	2.02859723003979	9.64373844089923	8.63891294427141
C	2.82597525613141	8.51239126301636	8.52715472721833
H	2.52803791139858	7.55519715475982	8.10710911734409
C	4.07146721292964	8.85572097274268	9.08523899922323
C	0.60995347059973	9.83175777697678	8.22842829719060
H	-0.05544252332299	9.96699144712020	9.09850567876403
H	0.26788141862516	8.94680485075854	7.67004725056237
H	0.48756942988846	10.71436932056862	7.57836480033305
C	5.28806319111262	7.97428143718566	9.28560460243051
C	4.87270721163702	6.50732627061780	9.13890376542252
H	4.07950640696511	6.23760762842246	9.85766149288156
H	5.74190725718836	5.85409850547509	9.32793147445291
H	4.50814536958280	6.28799424656362	8.12019830349217
C	5.84982636722345	8.20331866025257	10.69245391392937
H	6.21864360022182	9.23572394382403	10.80156087228928
H	6.69914223307626	7.52210842483955	10.87550200098997
H	5.08247181648194	8.02537591777331	11.46557194462985
C	6.38033216217035	8.28470019598635	8.25279299544555
H	6.03003662875469	8.07824110876494	7.22710338284930
H	7.26339979748227	7.65000853210159	8.44476005428482
H	6.70441053191932	9.33621328774506	8.30842270231094
C	1.96289765545730	11.92135672201943	12.46894937284522
C	2.83187643565967	11.92150331510391	13.54938718843329
H	2.55196945779778	11.92156523915435	14.59934747442083
C	4.13021218048992	11.92173167926240	13.00383815195093
C	5.43479286453739	11.92219057108500	13.77290031281982
C	5.14125896669596	11.92226825306940	15.27561428000154
H	6.09024427740262	11.92261866548299	15.83896717068711
H	4.56964374846188	12.81710153925653	15.57797069356659
C	6.24484016813547	13.17621205299082	13.42685104821151
H	6.50122504161456	13.18757386714443	12.35588338414762
H	7.18421022502379	13.19595744417734	14.00742466487312
H	5.67284492915721	14.09230832453730	13.65686507368950
C	6.80283182578914	11.92157355591231	8.48669776389482
C	5.37918184254083	11.92181436235376	6.41344487901629
C	6.64515040514203	11.92102580138616	5.55592945190890
H	7.25455249663008	11.03421518248781	5.80607059770784
C	6.26542022889678	11.92122506662529	4.07114431156810
H	7.18639766604366	11.92063467767635	3.46244831609524

C	5.43815047904379	13.17200914813878	3.75205070288780
H	5.18158972547010	13.19388165836910	2.67669190178221
H	6.03114335451601	14.08142095711411	3.96175315378842
C	4.16174092099208	13.17136740253098	4.59982645457929
H	3.56921071729496	14.07784790987885	4.38193978349537
C	3.33396088929549	11.92305883072202	4.28101767382847
H	3.03495114721392	11.92322758264603	3.21639488988770
H	2.40355657165319	11.92365415947922	4.87938330900030
C	4.54191281801343	13.16549853290173	6.08142909117076
H	5.12330332335845	14.06744888000788	6.33013635489258
H	3.64048523555493	13.17441168277875	6.71815069183291
B	2.26281420404963	11.92139843385151	9.89358687144360
H	1.05544607903873	11.92137195769471	9.82963244047734
H	7.25565141918064	12.80708874780055	5.80603450838509
C	5.43659197689106	10.67145608416807	3.75211567314957
H	5.18001818137676	10.64983680198503	2.67675498103754
H	6.02844160490532	9.76131112594139	3.96189417781741
C	4.16017611377601	10.67373028226629	4.59987319167360
H	3.56651595863875	9.76798140984731	4.38202173209187
C	4.54036328909753	10.67917277428869	6.08146111205307
H	5.12062118946797	9.77650509395215	6.33017946008548
H	3.63893452028691	10.67140687595435	6.71819484255741
N	4.01282715051427	13.71625255903330	9.49261110564473
N	2.77175157444830	13.23808900380573	9.23389874024696
C	2.02850041607382	14.19906020987312	8.63891547440591
C	2.82580003683200	15.33047505193391	8.52723707679317
H	2.52783278881565	16.28764579662103	8.10715925556855
C	4.07129499482882	14.98720200035451	9.08534681994695
C	0.60990003822369	14.01093424874892	8.22833177628761
H	-0.05554711394071	13.87565680367039	9.09836344051894
H	0.26780039806498	14.89585733046412	7.66992053384696
H	0.48762972383349	13.12830773582922	7.57826631839660
C	5.28790011034625	15.86861818523356	9.28570163932539
C	4.87262523359539	17.33557801685215	9.13881233924110
H	4.07940532761227	17.60542462022686	9.85750094797680
H	5.74184971597750	17.98878313928754	9.32780682667475
H	4.50812527016959	17.55480643919971	8.12006289402391
C	5.84953625098997	15.63966963465484	10.69262679922459
H	6.21826333092161	14.60724386940961	10.80186638106827
H	6.69888139961807	16.32083998069352	10.87568675062831
H	5.08212387816305	15.81773318820415	11.46565977682775
C	6.38021971204857	15.55801471054280	8.25301161006599
H	6.03001462579832	15.76441179106903	7.22728458504430
H	7.26332315525962	16.19265282165045	8.44499016082922
H	6.70421690431673	14.50648065788562	8.30876310225323
H	4.57018559817920	11.02715744745613	15.57817324905283
C	6.24557379044820	10.66857542175082	13.42712274861769
H	6.50199997403822	10.65716554641098	12.35616778444478
H	7.18493875250772	10.64949231026987	14.00772679258135
H	5.67410481807897	9.75219159464205	13.65730070782550
C	0.47338203623179	11.92120727081928	12.45404618053368
H	0.06887887582317	12.81066274083547	11.94081906101775
H	0.06905965703101	11.03159925618612	11.94094084182322
H	0.09376122499196	11.92123751800104	13.48730516367359

I(η^3)-P (S = 1)

Eh = -5351.3517814873			
Ti	-0.07813269933793	7.81220721085174	21.95390686600748
N	0.58140751305394	8.07218999286707	23.69471964104244
N	-1.93576620885204	8.86499655462868	22.41344942897866
N	-3.01175508363740	8.06570840583973	22.52101648779537
N	-1.25853308833169	7.51721690008753	19.99984844032412
N	-2.53804503977497	7.14455021772323	20.24138044157024
N	1.32624467492449	8.65458119155774	25.93190764512473
C	-4.00059840703169	8.69657282000007	23.19147142021412
C	-3.53345892766059	9.96543040960120	23.51089849627921
H	-4.08139080572457	10.74092447340921	24.04280189074490
C	-2.23006513343882	10.03564296776460	22.97904427448614
C	-5.32102050944244	8.06972208784535	23.47691609216574
H	-5.87918891753697	7.84812218009117	22.55067004405380
H	-5.93171163357079	8.74912064954519	24.09153406235011
H	-5.20627052302035	7.12024454472242	24.02788255513024
C	-1.29858104799614	11.22336450003491	22.92546571588726
C	-2.10470439539287	12.44016511745753	22.44659770380941
H	-2.55127389392948	12.25593711164846	21.45380737622345
H	-1.44475830904900	13.32331751432947	22.37106745844213
H	-2.92064768273883	12.68769533917829	23.14726331821658
C	-0.17024577508506	10.96289231017906	21.92526581663517
H	0.54907198627231	10.20712071577234	22.29492016405464
H	0.41897981494717	11.87964250299354	21.75592603024198
H	-0.58225347161565	10.64698618922061	20.95109036790716
C	-0.72485035394679	11.51807820553600	24.31665017216638
H	-1.53480938403034	11.63953151015675	25.05691198306589
H	-0.13952724551895	12.45275315208596	24.29415227507954
H	-0.06246701010816	10.70908806208959	24.66255781415251
C	-3.24401300622276	7.11653067528581	19.09163213288810
C	-2.37997569191179	7.47263326491639	18.06380355619149
H	-2.62653316505471	7.53707697516153	17.00656727199888
C	-1.14116365639894	7.71070793524078	18.68173271457461
C	0.15788751998758	8.12888762845373	18.03104486716359
C	0.29330171664262	9.65721614896342	18.08667438136236
H	1.23868417512811	9.98160153890198	17.61570015413136
H	-0.54361107669298	10.14435383608453	17.55696018545198
C	0.16972369213699	7.68243215248403	16.56585805454528
H	0.04638653141281	6.58904127563165	16.47927945026685
H	1.12967531649103	7.95945329806356	16.09567172541852
H	-0.63479532477307	8.16765905184797	15.98749682862570
C	1.20712365613163	8.50031546734757	24.67556702773178
C	0.26676541926745	8.01446122770152	26.72204602964685
C	0.52892120366393	8.27017030398668	28.20851349025136
H	0.54537830962574	9.36181037407850	28.38024653084906
C	-0.54568556237308	7.60299546872951	29.07418402408537
H	-0.33534678789765	7.80262698450280	30.14093660223261
C	-0.53914303137171	6.09121795212534	28.82418394910226
H	-1.29851934907377	5.59830505301553	29.46008700190925
H	0.44278133635426	5.66487111512205	29.10321582842669
C	-0.82435507841119	5.81764378639414	27.34413489742353
H	-0.81161304510898	4.72844312271926	27.15682321451848
C	-2.19552482235827	6.38791317273862	26.96754861152449
H	-2.99070543512647	5.89209754242935	27.55605033201426

H	-2.40269378175338	6.18883003585633	25.89902533232772
C	0.24852993041668	6.49350721631703	26.48910919058246
H	1.24918213637169	6.08655337054907	26.72728335153527
H	0.05824379522896	6.30351682011878	25.41847398528783
B	-3.05396867288900	6.79048345439987	21.66335278772829
H	-4.19993717924350	6.40211357753997	21.58804134744296
H	1.53228972134335	7.88471379203935	28.46846545332995
C	-1.92432347083649	8.16466682494912	28.70958915953756
H	-2.70283059499683	7.69618541960528	29.34098805664591
H	-1.95538497198882	9.25235662631124	28.90877717686021
C	-2.20715788644053	7.89757212047093	27.22819873234547
H	-3.19433894019254	8.31368654834034	26.95463894762617
C	-1.12648262123246	8.57118199790331	26.38024458208256
H	-1.12640719391155	9.66088400405661	26.56027062753092
H	-1.32962905609305	8.41667047391889	25.30609077034159
N	-0.84303283517067	5.78972053990414	22.23883019841917
N	-2.18401906678402	5.66399284698350	22.24068952974596
C	-2.54001475154733	4.41245319342466	22.60441208904865
C	-1.36899735550717	3.70489939216426	22.83722393826806
H	-1.29252638131549	2.66239542752099	23.14037089028913
C	-0.32007545637108	4.60815258031377	22.57533483367386
C	-3.95392007782763	3.95288113496627	22.69290763899051
H	-4.44723521244781	3.95599713454183	21.70557965940060
H	-3.98576621155176	2.92560265476759	23.08789774869384
H	-4.55132221286814	4.59695879767581	23.36017714889468
C	1.16144969251920	4.31210245321887	22.56774085492563
C	1.40399532565633	3.13345156095196	21.61103175276832
H	1.06447601082298	3.37816307008358	20.58921769456945
H	2.48187628724851	2.89405157934355	21.56933588816920
H	0.86522995346751	2.22754397059179	21.93836487025262
C	1.96471453851690	5.51608497559738	22.07675633630853
H	1.87439145414362	6.38571713016829	22.75215249119187
H	3.03865417926019	5.27031422479047	22.01986943846182
H	1.64998655725694	5.80395037660733	21.05647849889368
C	1.61301598650945	3.91656515447835	23.97929149958736
H	1.03437411479403	3.05399319651581	24.35383270432362
H	2.68110702263570	3.63477268162824	23.97567240176508
H	1.47580060945303	4.75250028640038	24.68329572192533
H	0.29302377005373	10.01922369753178	19.12719247835137
C	1.33480474733258	7.47351904049031	18.75194244135881
H	1.22955139199452	6.37548479891726	18.77460253985225
H	2.28356327666512	7.72270900666844	18.24791702995320
H	1.42769989327525	7.83627559722910	19.79185871478500
C	-4.68802330035561	6.75836083027436	19.01028214372666
H	-4.87594373446874	5.72779062183307	19.35719330943294
H	-5.30968514326848	7.43119135423681	19.62521785765299
H	-5.02775267662408	6.83517010595853	17.96567150689824
Ti	4.45888963143135	10.28985910690934	26.11345334747556
N	3.50836143391927	9.79648145948945	24.57347322743381
N	5.27398100603678	12.10338392175820	25.21658591978785
N	6.60730235675021	12.12138141163290	25.03161274602013
N	5.79821532372256	11.01296162082261	27.75127800456882
N	7.05586130830296	11.31465032559159	27.36092923855023
N	2.76753095633008	9.06908107997821	22.37253255702242
C	6.95552586528681	13.13596206975660	24.20976089980826

C	5.78788701988971	13.80627831800695	23.87043315518393
H	5.71115093283546	14.68365083820929	23.23150690305533
C	4.75173684432040	13.13145982365848	24.54668145751437
C	8.36017687871587	13.41943766812444	23.80367715368915
H	8.99159445429630	13.69024409575715	24.66763146883332
H	8.37850482685285	14.25700691966417	23.08918359302038
H	8.82592843165105	12.54521241593776	23.31663529305287
C	3.28506031116263	13.49080545177456	24.59390907987855
C	3.16517183690134	15.01240984170938	24.76136352659846
H	3.67144219568711	15.35455170051406	25.68100159492927
H	2.10143848789225	15.30392908003284	24.82113847907365
H	3.61198218848462	15.55009039340515	23.90786097256212
C	2.59353348034131	12.81091930124946	25.78035385609822
H	2.49400772130648	11.71866822116965	25.62075159222584
H	1.56518172556067	13.19417362782374	25.89461736811603
H	3.13415245134988	13.01785119400357	26.72183456931785
C	2.60611526939143	13.07182467136697	23.28398562987098
H	3.14497886117881	13.48265843180239	22.41225875691254
H	1.56922093271583	13.44970136962976	23.25287734356681
H	2.57841391968867	11.97479809196317	23.19051427059721
C	7.76925759664057	11.77026608221486	28.41411118712481
C	6.93073458036232	11.76077444057045	29.52290610723380
H	7.19198328062533	12.06651017580530	30.53445038688709
C	5.69620883007308	11.27601022437548	29.05373159989772
C	4.41140245456026	11.07928003011467	29.82230022575225
C	3.92879982629885	12.44482452246944	30.33271552759367
H	2.99452651975318	12.33265892590621	30.91188927054373
H	4.68248333295913	12.91715060404593	30.98575833578374
C	4.67557071288713	10.14327815876547	31.00979554332647
H	5.03348197281457	9.15857419958420	30.66185774031463
H	3.75070898256009	9.98789159179860	31.59369486611032
H	5.44024932529297	10.56167991867999	31.68637258700350
C	2.87593488073878	9.32416656773726	23.61699540882096
C	3.90197435782679	9.40150781117011	21.49724687211915
C	3.39677864245978	10.29601634536618	20.35547673559464
H	3.01363991303979	11.23980332414652	20.78355000090380
C	4.50996329307706	10.58290297213387	19.34447896157349
H	4.11128629814079	11.21950879219592	18.53322387859230
C	5.02405748551679	9.26370840431288	18.75843638859429
H	5.82109429536686	9.46312421684022	18.01780813796410
H	4.20915323211104	8.74456551850326	18.22094944480880
C	5.56140283286857	8.37415619932139	19.88485735169449
H	5.92155092926288	7.41837889992288	19.46287217649961
C	6.71395917477999	9.09454446733324	20.59119177911698
H	7.53983378093999	9.28503041836351	19.87987543366991
H	7.11759565910149	8.46024022180155	21.40256062150495
C	4.44486801661986	8.09384747283654	20.89470742052961
H	3.61702063820357	7.54183073355960	20.41832690811383
H	4.82036765129246	7.45860645955600	21.71643251338335
B	7.49426507043226	11.18926840896471	25.87463171034957
H	8.65535859078480	11.52332501069428	25.76462023469260
H	2.54539129146557	9.80020925908244	19.86086695572682
C	5.66287397206301	11.30229869470165	20.05057299167708
H	6.46739638532942	11.53466219650669	19.32742634547217
H	5.31214759318514	12.26402684723602	20.46885145546510

```
C     6.19830159540688   10.41210870053571   21.17495964882226
H     7.01896964456001   10.93286855919639   21.70201657607057
C     5.07995567678641   10.12259006123404   22.17930130190505
H     4.73301258901635   11.06509872437971   22.63312655420749
H     5.47040546480652    9.50475124966174   23.00479004808223
N     6.19824843195072    9.07875038582686   25.72472063195028
N     7.33967393634836    9.72442924756385   25.42994626887894
C     8.24429602492241    8.86320207473108   24.91854452642158
C     7.65152951219160    7.60439104715075   24.90554832518042
H     8.11283458810793    6.67821390410742   24.56955330568416
C     6.36379166175464    7.78462744066415   25.44612391495703
C     9.60940109538930    9.27312562497042   24.48625736581801
H    10.21016926035322    9.65115365517404   25.33137529877819
H    10.13654025809131    8.41128648422309   24.04864778953656
H     9.57024793900558   10.07308589775760   23.72699110594482
C     5.30985677436623    6.75396514248938   25.77461303920596
C     6.00562802440032    5.51139548127955   26.34613097505888
H     6.58540469889781    5.75944414898955   27.25259216781022
H     5.25317314415415    4.74851471243159   26.61329484249588
H     6.69551007899313    5.05849796050197   25.61340416062298
C     4.34452563151908    7.30249621856673   26.83129842578548
H     3.62813028342540    8.04158332750191   26.41193082373100
H     3.70032554093093    6.49071489728710   27.21167539486024
H     4.89723620651108    7.72287840613958   27.69089788240059
C     4.52979964036955    6.36442962744794   24.51471936090151
H     5.20375862750343    5.94331709187831   23.74814560364351
H     3.76765906682117    5.60589442828379   24.75902290357157
H     4.01325816546491    7.23315985619641   24.08103908423504
H     3.73376695398381   13.13083552698260   29.48968427107342
C     3.32153278930125   10.46654074786107   28.94444309367525
H     3.60970063216667    9.47504828800056   28.55418060439645
H     2.39940458472420   10.31443985438333   29.52917390373839
H     3.04088284874843   11.12905576540000   28.10376575068279
C     9.19625426506467   12.18891191875047   28.32406695802946
H     9.83676985941752   11.37238401104200   27.94987548488418
H     9.32740698151100   13.04812322639131   27.64385584879087
H     9.56048557581554   12.48124584932512   29.32112745260503
```

[3]$^{2+}$ (S = 0)

```
Eh = -5351.063194478126
Ti  4.21797613223742      5.77949364597396      5.04270075054013
N   5.26485344761178      4.34773132172178      3.91423636824450
N   4.41483477084322      3.95599895655346      2.92047998273839
N   2.18622073837713      5.85287105344758      4.39441460256336
N   2.10499365564198      5.03807022121846      3.30436081808552
N   4.46719882299219      6.94404752815603      3.24054447218774
N   3.92773369749939      6.27869393419520      2.18461194416336
N   4.93630099324033      6.62683637471354      6.39491293184105
N   5.47601780326836      6.70194415306473      8.77485033119320
C   4.84088674281015      2.80145999616696      2.37553634450739
C   6.02134604352977      2.44884571984872      3.01971760737973
H   6.63744660162953      1.57948251604369      2.80065780782654
C   6.27352923228542      3.45017383810070      3.96116515768189
C   4.13973807520726      2.09430543873290      1.27175530309537
```

H	3.07829756628195	1.91875433352359	1.51121607610694
H	4.61923679404919	1.12008372182265	1.09526785591644
H	4.18103444843498	2.66935329612793	0.33121252709922
C	7.52368061385909	3.55994295831362	4.79473316380942
C	7.72864941962511	2.24424386283654	5.55711246536411
H	6.86030708878029	2.01728216985755	6.19856130729947
H	8.62661833950796	2.31070143458634	6.19482517024577
H	7.87348214068859	1.39508440775826	4.86884697148389
C	7.45916341975408	4.72974933990357	5.76162918145079
H	7.29220653061971	5.68144205154537	5.23221010362658
H	8.41176475437777	4.81644191229784	6.30640201660778
H	6.65910444787032	4.59159756520693	6.50363892325177
C	8.70101793005779	3.78212314012025	3.82844290804496
H	8.79728723680108	2.95264578264857	3.10858541533542
H	9.64461337826600	3.85129009869208	4.39744779391425
H	8.57358316266086	4.71784498060231	3.25711996533566
C	0.81430384538611	4.76097517819160	3.02812279328900
C	0.03616308109092	5.44520483687837	3.95423160275402
H	-1.05110026318880	5.46101024384301	3.99439958813513
C	0.92718344224199	6.14798195079975	4.77312870333018
C	0.37863906293410	3.88791065608485	1.90615672886172
H	0.59183721822600	4.34792989508057	0.92614667640629
H	-0.70563783526413	3.71483222687777	1.97084716875974
H	0.88553587206103	2.90932750802204	1.93337312201257
C	0.56655303442616	7.20488232573450	5.78216886757371
C	-0.42220603202783	6.64315081126185	6.80933763545504
H	-1.33027099052262	6.25333498182048	6.31951916357486
H	-0.73867506820563	7.44058691579638	7.50339649902588
H	0.02396717772345	5.82605771435687	7.39828878731185
C	-0.10887926375048	8.35404364543778	5.01028937383732
H	0.56419723901287	8.76438704488693	4.23804801260750
H	-0.37209850530187	9.16792779361113	5.70836518508461
H	-1.03480389325723	8.01924940326977	4.51414599513545
C	1.81842329033601	7.74090814940960	6.45539493104768
H	2.34579912024802	6.94896922695221	7.00749322013193
H	1.55834515286805	8.53300690135590	7.17243795558105
H	2.50931093295482	8.17869005792454	5.71670414706702
C	4.00758790484950	7.01444806287618	1.06222414748348
C	4.63295964288632	8.20874186937878	1.40238333059553
H	4.85984170287691	9.02781885709777	0.72630823141635
C	4.91479797596237	8.13338408093535	2.76929740032480
C	3.49877346572151	6.56728781455907	-0.26186758032497
H	3.98873640574130	5.63593525865816	-0.59217273855106
H	3.69663166147698	7.34308249466479	-1.01620047642496
H	2.41118247347600	6.38380853898203	-0.23672607694272
C	5.64735592115744	9.17076409852324	3.58444527520574
C	5.97588952338196	10.38142257120908	2.70517352421073
H	6.52106668075272	11.13150372575731	3.30219808942515
H	5.06232265047139	10.86292053544165	2.31681692762829
H	6.61759778106646	10.10609567988825	1.85173290057639
C	6.96412973586471	8.55222764176913	4.07201359376295
H	7.53914618344249	9.28154248997622	4.66734505083370
H	7.58696041683180	8.24655824606590	3.21383012874878
H	6.78036399256124	7.66427242511156	4.69540048632625
C	4.76862311514616	9.64534197467737	4.74808715055504

H	4.60843663966591	8.84690056108666	5.48696152823890
H	3.78746055217256	9.98926749656290	4.37726237723606
H	5.24668091016372	10.49568811513348	5.26238858755327
C	4.93717278616069	6.23928435808680	7.67290841135745
B	3.31987156487253	4.88910753995617	2.36270950295029
H	2.93996501262006	4.44771763457824	1.30806072297169
N	4.06252808986136	4.62448111662508	9.17415148296046
N	3.75460992586679	4.40591680636777	6.76369603328780
C	4.15908868141443	4.95391883312509	7.88354174572075
Ti	4.77825633818170	5.47200802313548	10.52664479502023
N	3.96747827050001	7.15889657496467	11.53007242889171
N	4.87176207211815	7.47305457169888	12.50104781474320
N	6.70168240050913	5.04813227005097	11.27606255006704
N	6.87156650693709	5.84528076687127	12.37102139249244
N	4.17550794469485	4.52422822535194	12.37352465979367
N	4.72726750705914	5.18228501536777	13.42731714940723
C	4.64519867994658	8.72178000053714	12.95722857361845
C	3.53608098786728	9.21556864248994	12.28028625753126
H	3.06975127179413	10.18693279180199	12.43088257242353
C	3.11512872346600	8.19603348436129	11.41822982856826
C	5.46768739772916	9.37251927475532	14.01084796388013
H	6.54385218633645	9.30550846866412	13.78268047619081
H	5.19746656230027	10.43600133017193	14.08965964604059
H	5.30562850435675	8.90615727633392	14.99767589189378
C	1.83009776087469	8.16407865654814	10.63439858933192
C	1.72659645982371	9.40437541323367	9.74028803542651
H	2.52469154502052	9.42223731572824	8.98072721037699
H	0.75211183990730	9.41632529684097	9.22237011544558
H	1.79739468742454	10.33322054601185	10.33093787010373
C	1.73623101458580	6.88664836422900	9.81740352121070
H	1.77420584398070	5.99845097361922	10.46835367110856
H	0.78520290646439	6.85334909749875	9.26586764741437
H	2.55200894026213	6.82122158458487	9.08288800019868
C	0.68112317297398	8.17553669426283	11.66015212667898
H	0.69080353339386	9.09343563865165	12.27106580310295
H	-0.28918138410733	8.12434184730306	11.13572359845182
H	0.75093662355574	7.30915942327462	12.34039659338626
C	8.16928621603360	5.87327270894539	12.72709895260176
C	8.85846358050816	5.04489450943801	11.84940177502149
H	9.92443724158266	4.82860899435774	11.87309229806386
C	7.90866564476925	4.52713047007192	10.96517672951402
C	8.70227975749757	6.65354709813540	13.87494068034558
H	8.33776922935271	6.25813620387365	14.83837614051750
H	9.80100613847615	6.59962198544825	13.87932240038197
H	8.40919632948042	7.71448083222645	13.81609117925860
C	8.19116611061516	3.50416285254208	9.89644672926918
C	9.25781250749673	4.07557855836813	8.95269174018416
H	10.19623059642778	4.29294312116094	9.48916931838396
H	9.48888845953583	3.35010202733902	8.15433974333989
H	8.90887792192504	5.01249794979373	8.48661469059800
C	8.73529906840193	2.24001800042853	10.58306810251091
H	7.99186321526090	1.81461042833400	11.27879006257023
H	8.97314145994811	1.47554482259240	9.82309111702681
H	9.65587164100818	2.44903934901005	11.15237485788488
C	6.93724279489890	3.13689846144581	9.12146234612942

H	6.54590593191718	3.99863454349301	8.56130780145645
H	7.16536126332896	2.34182592610650	8.39549946497629
H	6.14614875839545	2.76052525171725	9.78993877096282
C	4.30992528451996	4.65858468614766	14.59304516485654
C	3.45257296164077	3.60861697007437	14.28301922881563
H	2.94166193762968	2.96679519647458	14.99484919342540
C	3.39117566670752	3.54569243687005	12.88806138796407
C	4.73387938033221	5.16294544668987	15.92638005911719
H	4.44789396429547	6.21883290523107	16.06942869242608
H	4.25297153477769	4.56872338467000	16.71745928000999
H	5.82665581426100	5.09092620557396	16.05931740449078
C	2.61992900274052	2.53402434433881	12.07669329270380
C	1.84016228356836	1.60384070483150	13.01198963903077
H	1.28748308676133	0.86039542754264	12.41323170885544
H	2.50888470712822	1.05046102688857	13.69235689941787
H	1.10408074703863	2.15869526700846	13.61831634758178
C	1.61616627434813	3.25204827336218	11.16669798908255
H	0.96312225718301	2.51869654560800	10.66532572712779
H	0.97019205487186	3.92434078116191	11.75798904268246
H	2.12528521742453	3.84112319384319	10.39031829473146
C	3.62489252100939	1.69368300903213	11.27788424874293
H	4.22081300214948	2.32273677411481	10.59988554374836
H	4.31817358229675	1.17195573384346	11.95995376292512
H	3.10366433928055	0.93107532809501	10.67501411726760
B	5.67806604761634	6.35545466835481	13.20261527175375
H	6.09323877040543	6.78084373978060	14.25036977849650
C	6.32751846749912	7.90703600143064	8.78259890631480
C	5.45042969697479	9.16330819656370	8.65949310915890
C	7.36844091401399	7.89800118703749	7.65585920610261
C	7.09282267760193	7.96502483161166	10.10689250919947
H	4.71422113699639	9.16788007782789	9.48185078050850
C	6.32278231800437	10.42122332472944	8.72993693173271
H	4.89076489122544	9.13069430413798	7.70838963048332
C	8.23961070216179	9.15814206143407	7.72958272197705
H	6.86439288213365	7.85040661393791	6.68113448589746
H	7.99322866455945	6.99096426224347	7.75046343801438
C	7.94995481776558	9.22739432029667	10.19785043967480
H	6.38065579082068	7.95064823601813	10.94344320461223
H	7.73817736724238	7.07451815297150	10.18921759112195
H	5.67501757660241	11.30918191194212	8.62883239340109
C	7.35330135919948	10.40011505154124	7.59825562944944
C	7.05028189954906	10.45959521221718	10.07703421478019
H	8.96760327691042	9.13109140146953	6.90022731126669
C	8.97959820723227	9.20792964617074	9.06761671879599
H	8.46683418176771	9.23475401601521	11.17401087637504
H	7.97425284433034	11.31253423617246	7.63660295699278
H	6.84233391808672	10.40062685662800	6.62087635818358
H	7.65219502567295	11.38200350607548	10.15818193740171
H	6.31586773084297	10.47706145594314	10.90443515114728
H	9.62183945831521	10.10507866344100	9.11474477444278
H	9.64241546457279	8.32959383816721	9.17474623303952
C	3.02899232032696	3.12172287477847	6.74392917964323
C	3.69808703531891	2.05299264111984	7.61890650677396
C	3.00668057876331	2.58723295447697	5.31085339253276
C	1.58191508796357	3.34030792225538	7.21306239016183

H	3.74431799174793	2.39574747075214	8.66127079014553
C	2.91233177878434	0.73777873217394	7.53701550774420
H	4.73770805792506	1.90424767296287	7.27261235342998
C	2.21124206361586	1.28547245916370	5.21475224851480
H	4.04229843332453	2.41085693394713	4.97723769857270
H	2.55172186292714	3.33616709308065	4.64872266541412
C	0.80105834548622	2.02450825985644	7.13523794542460
H	1.58270200143894	3.73540936091018	8.24372239753641
H	1.10957177273274	4.09704846694982	6.56397852316852
H	3.40515026048784	-0.00695736142627	8.18587755719956
C	2.88436055764989	0.23070898119981	6.09410648878897
C	1.47598440101258	0.96888102108545	8.01501319736543
H	2.20827554055895	0.94943941216272	4.16251531906807
C	0.77598205881100	1.53360520661558	5.68459921142799
H	-0.22952151175216	2.20178052724700	7.48821377615506
H	2.33646330592854	-0.72630421938651	6.03690553162220
H	3.91183796568795	0.03868607785959	5.73449112921989
H	0.90799141992970	0.02305028245964	7.97134677483711
H	1.47408222194544	1.29562118064430	9.06801322218851
H	0.18160974101286	0.60624090075345	5.60741652603940
H	0.28918894221755	2.28669399114955	5.03650823684506

4 (S = 1)

Eh = -5351.072638908102

Ti	3.89853525945205	5.51127930771788	5.16882656126971
N	5.12171186987452	4.45658915778179	3.82159573062137
N	4.34493082970324	4.12446269755954	2.75418982558689
N	1.88689150873924	5.31283787595633	4.56477764376123
N	1.90559352307424	4.73000428409482	3.33420708815308
N	3.87562304571080	7.01275485095068	3.57616957359693
N	3.46311368864550	6.42938462547779	2.41873796286887
N	4.70615656882978	6.61773965040557	6.67144631962808
N	5.83575768075978	6.98765640875775	8.82376067624878
C	4.95355131058135	3.17753017477157	2.01471608013605
C	6.17786482228003	2.90551925337157	2.61603415028451
H	6.92899565800142	2.20482316606022	2.25736154096450
C	6.26035755301161	3.73525864339972	3.73760263664658
C	4.37511580048783	2.59140057427201	0.77592413127854
H	3.37784687614105	2.15817186415477	0.95862700799781
H	5.03267861399396	1.79182877567755	0.40368962850027
H	4.27034182037530	3.34675588498195	-0.02111843994240
C	7.44846115071509	3.86221715034725	4.65637353857343
C	7.76458392459677	2.48999173336189	5.26587441471664
H	6.90647176649805	2.10369518929985	5.84158472695525
H	8.63278333577683	2.56710609916282	5.94327021608338
H	8.01106789144469	1.74823012123030	4.48798744102840
C	7.17589711679741	4.87320466214661	5.75578187270225
H	6.98817185739876	5.87571657073570	5.33670664459965
H	8.04504879490433	4.95146125104065	6.42874601712421
H	6.31228961431432	4.56990720595274	6.36840761054498
C	8.64818247619888	4.34372124141927	3.82419506897593
H	8.90114069822468	3.63014850985280	3.02278094825573
H	9.53449237679519	4.45322379097901	4.47360012078227
H	8.43902056606502	5.32158373729903	3.35736029259248

C	0.68244618060421	4.25612091658938	3.02898011762459
C	-0.16158680198610	4.57240457452844	4.08999019137673
H	-1.22402576856454	4.35195188615069	4.15219979487488
C	0.61858356718937	5.25850547074526	5.02551305204068
C	0.36088905704330	3.55365197617367	1.75838323240472
H	0.42131230564450	4.23509539678147	0.89265694496141
H	-0.66297683698541	3.15397179288777	1.80477230985304
H	1.05125539749455	2.71495258290812	1.57221160176932
C	0.12707988032197	5.99937920708021	6.24416227393865
C	-1.22899007025388	5.44392404414206	6.68849311437975
H	-2.00522595831905	5.58976117533532	5.91934688939167
H	-1.56629672974368	5.97013281741554	7.59741496531582
H	-1.16677100017827	4.36650151590572	6.92161770367888
C	-0.02943746596502	7.47659583213445	5.83868823357493
H	0.93729493553252	7.90801304818971	5.52708167775984
H	-0.41582948762650	8.06355274931336	6.69101975496772
H	-0.73749911852331	7.58603719322874	5.00005659427685
C	1.10819256367232	5.89059669133940	7.40048439386663
H	1.23195011371846	4.84987282033110	7.72730889311745
H	0.74031538718125	6.47301649678693	8.26032310543742
H	2.09864586778193	6.29921128720776	7.13357673043740
C	3.39843844487287	7.32763510493170	1.41932406054187
C	3.78554071400798	8.55204106429487	1.94947689719966
H	3.84837022774812	9.49307285301963	1.41065539321000
C	4.07678071521117	8.32282151992218	3.29764877962614
C	2.98232059138442	6.99743090859817	0.02989620374783
H	3.63282761352761	6.22665757223085	-0.41675728484404
H	3.03972994743891	7.89862782935476	-0.59826523993745
H	1.94525693068374	6.62270757362366	-0.00571790035861
C	4.57529915981359	9.35317424567938	4.28162985023158
C	4.64807975585838	10.72916123312513	3.61065139602730
H	5.00972144768468	11.47348981633597	4.34043328934512
H	3.65927298174566	11.06378177972210	3.25430309316693
H	5.34751511434473	10.73259349422720	2.75791130254985
C	5.99359618212755	8.96305205830649	4.71329566548338
H	6.38922357630361	9.69427588205931	5.43800091463214
H	6.67000336800918	8.95109522852611	3.84141188145826
H	6.01696896261518	7.96675132835570	5.17714172696328
C	3.61085409660310	9.45395548783513	5.46968240479383
H	3.48465824223998	8.48956283611995	5.98082865497472
H	2.61962072996245	9.80042953169136	5.12992448435803
H	3.98822968924795	10.18393741553271	6.20731060165998
C	5.27637018454919	6.82522673007835	7.69079224380070
B	3.09774523861595	4.94870408507694	2.37176598430713
H	2.75470227203052	4.62632537966071	1.26291593594217
N	3.94302681563053	4.80151064664674	8.95538911384578
N	3.66937552446964	3.92853728192416	6.67205082441437
C	3.80240676671485	4.34321838311619	7.87023830046433
Ti	4.86803526533315	5.85383060817060	10.43466220654833
N	4.29431052403966	7.63103558535905	11.41838248238329
N	5.07362208140699	7.73174932706054	12.53056710227246
N	6.62086673662736	5.11512039550488	11.33842033624898
N	6.80075382957171	5.81964958628811	12.48892406317210
N	3.88255383214254	4.98068185492452	12.17521125127942
N	4.47395284028012	5.45456208782070	13.30538793663632

C	5.02415514667742	8.98653545423154	13.01664990726957
C	4.15658653961100	9.71238757412002	12.20533700702505
H	3.87769902447542	10.75470505635418	12.33588874037057
C	3.69511560338313	8.82595624035555	11.22761279882398
C	5.77390158236881	9.43950116556368	14.21800529523199
H	6.84968428584215	9.21399052561253	14.13452076469486
H	5.65929540394808	10.52715868432162	14.33703407207959
H	5.40113840591762	8.95408759720059	15.13609328255177
C	2.57183219299175	9.06183325258717	10.24886309379660
C	2.82566030911223	8.36321431011468	8.92302429151033
H	2.95672100730481	7.27402746745242	9.05252219608235
H	1.96206881399366	8.50357447007912	8.25342382443092
H	3.71213639684060	8.76800564115451	8.41734018832695
C	1.29281558139193	8.49260880584947	10.88945245279832
H	1.07702384311214	8.98412181396245	11.85286925983770
H	0.43006225700228	8.65840015609834	10.22019382877138
H	1.38964627547328	7.40876953755122	11.07269139944394
C	2.39586312459627	10.56126406198294	9.99332231873707
H	3.32040407619781	11.01420943785007	9.59494584298817
H	1.59337528459908	10.72076925424612	9.25343603762749
H	2.10923481588762	11.10507090191443	10.90849683636169
C	8.06214623856114	5.66664109829585	12.93446116052298
C	8.71221335797568	4.80948418270496	12.05320236969480
H	9.73803065991305	4.45677357146043	12.13711718596124
C	7.77746137332542	4.46984374875225	11.07164279968427
C	8.59286463952702	6.31210898261784	14.16409263450095
H	8.10272559223156	5.92116642742325	15.07192347935436
H	9.67195612985017	6.11459886183716	14.24782656015930
H	8.44242784605277	7.40384705712028	14.14638555401037
C	7.99142232025004	3.47828130128263	9.95745979092171
C	9.22827677395308	3.88173519021565	9.14457412026274
H	10.13682247277822	3.89829502530078	9.76928418236696
H	9.39712297572590	3.15712413904874	8.32929133042012
H	9.10396068458135	4.88357685982291	8.69922118702264
C	8.22433162969178	2.09669858649687	10.59220500901344
H	7.35617863256504	1.78853660583806	11.19985044906977
H	8.37887026876783	1.34151799922881	9.80163088059053
H	9.11449489236810	2.09422259597121	11.24296270970725
C	6.77287431076595	3.40955324923639	9.05479471164017
H	6.55943665012286	4.39125539745931	8.60320838114916
H	6.94616811141593	2.69663391358295	8.23296651664638
H	5.88505830230469	3.06627636453109	9.61127513655783
C	3.86020130882884	4.98513112799090	14.40654780910526
C	2.82196356284281	4.16897831172915	13.97461091630511
H	2.12738614527688	3.62655884006095	14.60945451518467
C	2.85975926958419	4.18779115686813	12.57658099099972
C	4.27356036523223	5.32202116992240	15.79543527942968
H	4.19013925124151	6.40403437816259	15.99427410998228
H	3.62877501649163	4.79273305037235	16.51251382482748
H	5.31794749183616	5.02580408078783	15.99104655626085
C	1.93560324604938	3.42181984421156	11.65859712573802
C	0.86628674818558	2.69496182275910	12.48149412739717
H	0.18848788619777	2.15031176109811	11.80239882982839
H	1.30975752837088	1.95668717108715	13.17040957312319
H	0.25471437422730	3.39993876579903	13.06961718927433

C	1.22010415899571	4.38461803203797	10.70352992964901
H	0.56987440605047	3.82094212923666	10.01121481597095
H	0.58141870916204	5.08313066995551	11.27144089274215
H	1.92750792613785	4.97057345849176	10.10059477358497
C	2.75448272205448	2.36313690612662	10.90924145243788
H	3.55031417139792	2.81787784537837	10.30105093060343
H	3.22454349113711	1.66552062370262	11.62357928654767
H	2.10008738208697	1.77469516347652	10.24360264051517
B	5.61620767457116	6.46458465076875	13.23592823552544
H	5.98543778991090	6.77273522632003	14.34059238599233
C	6.90852088738833	8.03818870207303	8.91850129261860
C	6.30831504719026	9.43673990973610	8.73240135421947
C	7.97371961746338	7.80090548496780	7.84162629645780
C	7.58101258392923	7.96458717468398	10.28619181757137
H	5.54293767140399	9.61182459280647	9.50744309426386
C	7.40790031601868	10.49991471453818	8.83507690774021
H	5.81061506196620	9.49747406450851	7.74726031954344
C	9.07557266875173	8.86161808243512	7.94480270179089
H	7.50942076490925	7.83385770451792	6.84070332593973
H	8.39970010887456	6.79088846150020	7.97336929315712
C	8.67531942245560	9.02927170855449	10.40146136299490
H	6.83072510185107	8.12277160276416	11.07493745118327
H	8.02469125614545	6.96561420940501	10.42491272389276
H	6.95415972344856	11.49593171103271	8.69381394274295
C	8.46509957286083	10.25349570299021	7.75498948210305
C	8.05750412010092	10.41765918811798	10.21899438171124
H	9.82663154103791	8.67199445474046	7.15921211866867
C	9.73405121208521	8.77891173780366	9.32466876250962
H	9.13624378260826	8.95190220531543	11.40179281047958
H	9.25454349599972	11.02312575337412	7.81464899505761
H	8.01361686386534	10.33992394914425	6.75040353417997
H	8.83283381353803	11.19726509739946	10.32147702624174
H	7.30221859095097	10.60565874793759	11.00500137130645
H	10.54473095246180	9.52429264393609	9.40346762851646
H	10.19580539362163	7.78483054864486	9.47063898804917
C	3.21996521021471	2.50473524039085	6.48760168461848
C	4.12728570263369	1.56188821779804	7.28657178699503
C	3.30523779377347	2.12347765169052	5.01250121417349
C	1.77313746419362	2.33087462550276	6.96509992596480
H	4.09083660830671	1.82507574055092	8.35852267401957
C	3.68560270129310	0.10747415904893	7.09157483225217
H	5.16986178098717	1.69563727270681	6.94964575409199
C	2.85243907120756	0.67407690908579	4.80987428615652
H	4.34414613955161	2.23651104010922	4.66215003718160
H	2.66482132416464	2.79411197287636	4.41868108245622
C	1.32712405029646	0.87617254471190	6.77419875999070
H	1.69928840447829	2.61443116745595	8.03114094987688
H	1.11418749091987	3.00443204498422	6.39081902477961
H	4.35210690577507	-0.54875121060198	7.67660232872148
C	3.77107089801461	-0.25492418127942	5.60693394353193
C	2.24182653084059	-0.05427744825431	7.57578028151423
H	2.92076057937515	0.43337813724808	3.73454864870049
C	1.40642932586304	0.51957765694326	5.28716536878936
H	0.28694407975976	0.77410071149313	7.12829961036866
H	3.47606720941735	-1.30808828564451	5.45596369573136

```
H   4.81250713439424     -0.15871903103970      5.24842904381214
H   1.91699978534620     -1.10236840144719      7.45279690266161
H   2.17306191779230      0.17473075211209      8.65434513237086
H   1.05962358700142     -0.51610678357415      5.12442461653338
H   0.73932685387325      1.18051294251066      4.70269009099394
```

4 (S = 0, broken symmetry 1,1)

```
Eh = -5351.072722300391
Ti  3.89733878310764      5.51400592629601      5.17216706864412
N   5.12056360919909      4.45765176213438      3.82431636831157
N   4.34220471228286      4.12298417989644      2.75878991897567
N   1.88564185671894      5.31306690929376      4.56931545974178
N   1.90297656164321      4.72842923950848      3.33962672191706
N   3.87180270596799      7.01172081204234      3.57565433608866
N   3.45885549009955      6.42635298915376      2.41949908082746
N   4.71059238435581      6.62103631083369      6.67054003160964
N   5.84456673899225      6.99742162232717      8.81950004772540
C   4.94985772911042      3.17459909883718      2.02029114939529
C   6.17522261131696      2.90440914506086      2.62000346005958
H   6.92609349972680      2.20336869181349      2.26147502596872
C   6.25926672296707      3.73660476007856      3.73971990090909
C   4.36922308009665      2.58498852088156      0.78419655190650
H   3.37285982473159      2.15110569006367      0.97034807126049
H   5.02675497142259      1.78515977798994      0.41245375180889
H   4.26179927984521      3.33818739006465     -0.01451655733581
C   7.44970834734290      3.86598201531549      4.65513317175290
C   7.76790263137772      2.49497996224283      5.26631245727674
H   6.91102099572686      2.10891664133976      5.84399497397402
H   8.63721861967160      2.57365621457089      5.94208273985078
H   8.01356475159913      1.75219792846945      4.48914345558054
C   7.17988412004747      4.87917137284814      5.75313276395121
H   6.99061996497360      5.88070871767975      5.33248451799809
H   8.05082511996240      4.95901317643844      6.42352259002667
H   6.31798612078479      4.57686704992328      6.36851456436624
C   8.64705942475438      4.34621114210877      3.81880916835461
H   8.89834371022573      3.63098192241955      3.01835159075471
H   9.53494481157932      4.45764461679419      4.46573549063235
H   8.43620301758630      5.32291399922767      3.35031080126338
C   0.67940814640961      4.25461705967019      3.03621686841010
C  -0.16353371756158      4.57257913857225      4.09766147537135
H  -1.22592962147264      4.35235453023359      4.16132577044773
C   0.61776743828793      5.25953712478657      5.03148477838507
C   0.35646382058090      3.55025280230827      1.76703008644708
H   0.41647091300063      4.23023737389784      0.90013577480016
H  -0.66752678549173      3.15107918779895      1.81494569118652
H   1.04634184970889      2.71097210104799      1.58166873127745
C   0.12843468468721      6.00073567073108      6.25072369244732
C  -1.22834509849581      5.44754723711512      6.69562459338445
H  -2.00479529306291      5.59541208539870      5.92708280968834
H  -1.56403532364821      5.97372212809920      7.60516041049439
H  -1.16794024621649      4.36984881723764      6.92791870767483
C  -0.02541215606252      7.47859222400671      5.84678807336162
H   0.94189254692014      7.90844475663166      5.53483693775630
H  -0.41002502234332      8.06552353224316      6.69993311529103
```

H	-0.73387996090315	7.59022116916672	5.00878862771671
C	1.11047204929129	5.88828009370462	7.40588781898313
H	1.23239217179422	4.84671844657120	7.73080336697398
H	0.74514750505119	6.47022706596331	8.26714826887749
H	2.10153222507593	6.29508753769599	7.13833503439197
C	3.39302749691575	7.32283300733354	1.41862966686995
C	3.77987918445591	8.54831158974908	1.94649108543009
H	3.84196443987519	9.48847212560252	1.40606352688342
C	4.07207199815642	8.32152448290388	3.29485651128508
C	2.97604228847362	6.98995231853710	0.03010657002426
H	3.62624843306816	6.21827317635862	-0.41541472496187
H	3.03306148286884	7.88990669063601	-0.59986923839728
H	1.93894746317940	6.61517333233498	-0.00410646282624
C	4.57132934428854	9.35375737400371	4.27645540196997
C	4.64146717696163	10.72893062866331	3.60353014418459
H	5.00367264291978	11.47456955638162	4.33169575354354
H	3.65167082846613	11.06203808644552	3.24850597813460
H	5.33937798736408	10.73199560138426	2.74955107354359
C	5.99089232836640	8.96553488894311	4.70570129934031
H	6.38666825570404	9.69700165191328	5.43008430977992
H	6.66583468393773	8.95487262346413	3.83267799592875
H	6.01650078537174	7.96901272521544	5.16894354260106
C	3.60923310111624	9.45525958750138	5.46626181355127
H	3.48464634367729	8.49137375517168	5.97871576525217
H	2.61709695541086	9.80076918437161	5.12816436431552
H	3.98765617337521	10.18623891608602	6.20235595858519
C	5.28309218812441	6.83166088955613	7.68808798342778
B	3.09428315690289	4.94549153993955	2.37578648458431
H	2.75035877944239	4.62066513034129	1.26793918978179
N	3.94776769391391	4.80282708522101	8.94971337765683
N	3.66920494658685	3.92583341446951	6.66843360482016
C	3.80464798527154	4.34260555099223	7.86562745417861
Ti	4.87596040018757	5.85784513573839	10.42497316398191
N	4.30284015061277	7.63474430046269	11.41064897687371
N	5.08451396714523	7.73788213978136	12.52087291550706
N	6.63304729413995	5.12448413418966	11.32655453805203
N	6.81459519477380	5.82886435854616	12.47674394146523
N	3.89725551119145	4.98527348864136	12.16968348372196
N	4.49047147001021	5.46044900557207	13.29830218461996
C	5.03281121209190	8.99259014035384	13.00684206648658
C	4.16121673309010	9.71588383862585	12.19753650563418
H	3.87991712518747	10.75750869040184	12.32836555608782
C	3.70001608630380	8.82805278555904	11.22101290768925
C	5.78490085329642	9.44787769699376	14.20585191026491
H	6.86099553642484	9.22503133902139	14.11918375784349
H	5.66791681596619	10.53528826160666	14.32481774167365
H	5.41614548136047	8.96187196973819	15.12523473374359
C	2.57498441170093	9.06105282810628	10.24368669020471
C	2.83170234658203	8.36587811889338	8.91661195293496
H	2.96609645260009	7.27687392141379	9.04419579487431
H	1.96820848914356	8.50465725612959	8.24655551721282
H	3.71741149942733	8.77438573042392	8.41258989182169
C	1.29867033329825	8.48540002825078	10.88380931048837
H	1.08091976905222	8.97485295972153	11.84783663271043
H	0.43504121013399	8.64811940931142	10.21492521863493

H	1.40030050838913	7.40181838702186	11.06579213056000
C	2.39269574786419	10.56018376343857	9.99090912410874
H	3.31510227445667	11.01767665755068	9.59279664651840
H	1.58910960526398	10.71768062797491	9.25178142248262
H	2.10434814793972	11.10112982316080	10.90723346911674
C	8.07682265516966	5.67654515494735	12.92019024092192
C	8.72568993304180	4.81948992202500	12.03802977119342
H	9.75186272485381	4.46736272060152	12.12004919560215
C	7.78937629518755	4.47953678566714	11.05802058382037
C	8.60942399065332	6.32342933787865	14.14828303277595
H	8.12131599663682	5.93301233773676	15.05741510188754
H	9.68877162647465	6.12678553353747	14.23014473789589
H	8.45821006233558	7.41504229083902	14.12980117441882
C	8.00180765461645	3.48787627336540	9.94368121951267
C	9.23355132543634	3.89531754808243	9.12499790532139
H	10.14478808236635	3.91432971478014	9.74570879856115
H	9.40088921817232	3.17160388412610	8.30862214751537
H	9.10434180041595	4.89703076799198	8.68081205188717
C	8.24268694442729	2.10749098677364	10.57797207676078
H	7.37837694426421	1.79625725633474	11.18950720213597
H	8.39670747753983	1.35257246084720	9.78705119221465
H	9.13565992064618	2.10862975907337	11.22485740811715
C	6.77901666356246	3.41421209121201	9.04721707434643
H	6.56092263780111	4.39452088531809	8.59487164795388
H	6.94978552602819	2.70033150275825	8.22573888909741
H	5.89492821285746	3.06960367468407	9.60885294715981
C	3.87979930319772	4.99080982075219	14.40102956715841
C	2.84169779051031	4.17303832037432	13.97181870831957
H	2.14929309271291	3.63000115805372	14.60849286304032
C	2.87608813276784	4.19144081069193	12.57372621558012
C	4.29548120942499	5.32922439892390	15.78884255040394
H	4.20970161194903	6.41110552795915	15.98744811381756
H	3.65360415882145	4.79859702062240	16.50754648104177
H	5.34104261778160	5.03575410800151	15.98223014167128
C	1.95024407922291	3.42404332550630	11.65858816085007
C	0.88347075661744	2.69693575185117	12.48466576711510
H	0.20448767298351	2.15125693386775	11.80757841494440
H	1.32912019243979	1.95948028857599	13.17304535427000
H	0.27271763102936	3.40184899669214	13.07371317203797
C	1.23147699563218	4.38553342052440	10.70469788768573
H	0.58037041193007	3.82073407316967	10.01413588507151
H	0.59318889732390	5.08345958978736	11.27376155345990
H	1.93672833030193	4.97210864739402	10.09985650379320
C	2.76790361409236	2.36523559229592	10.90805396869029
H	3.56241382927370	2.81972462532345	10.29797005326407
H	3.23955117267200	1.66811124467577	11.62181787465552
H	2.11232310842053	1.77630717722755	10.24400348686711
B	5.63093921684655	6.47234289560027	13.22584500735321
H	6.00182552064024	6.78175846070395	14.32958578642743
C	6.91485765147603	8.05000346250399	8.91202510483132
C	6.31066629939432	9.44750456377491	8.73068642958878
C	7.97618643780087	7.81714842996145	7.83031914751663
C	7.59294720706281	7.97528071248206	10.27684120885085
H	5.54767976281055	9.61937374247636	9.50879677593998
C	7.40819642968629	10.51298120890041	8.83119335095400

H	5.80932787595761	9.50894479549958	7.74741377880630
C	9.07599930144288	8.88018987501314	7.93102464413544
H	7.50756302246272	7.85093947375393	6.83145747246738
H	8.40498430861489	6.80784962264452	7.95830295025144
C	8.68529828308521	9.04219330199223	10.38965253826025
H	6.84540445923489	8.13017878238194	11.06878082990647
H	8.03921775199093	6.97693814928068	10.41183250223833
H	6.95165615110235	11.50824629200470	8.69369680523709
C	8.46162726866831	10.27105901109117	7.74641243766714
C	8.06352863932530	10.42948940593674	10.21233712949224
H	9.82430634258218	8.69380842814344	7.14203656361749
C	9.74022746114504	8.79633630060751	9.30808333656937
H	9.15053846919961	8.96405020053722	11.38792754738181
H	9.24956098422746	11.04236352203784	7.80440778205036
H	8.00591539089279	10.35848962394941	6.74380665998856
H	8.83742678626393	11.21072199173147	10.31324990082999
H	7.31093793730885	10.61420185629290	11.00171674573359
H	10.54952640445272	9.54340578681857	9.38503911534448
H	10.20477927092314	7.80301151934672	9.45028858933495
C	3.22043002733535	2.50217298406801	6.48556235504618
C	4.12962195346378	1.56044307891674	7.28379738309139
C	3.30382694579466	2.12024136483988	5.01060416933378
C	1.77442495267241	2.32768967085639	6.96539011807315
H	4.09463225017974	1.82432779258790	8.35562983477426
C	3.68858980879611	0.10564253279565	7.09035547268207
H	5.17157881660485	1.69472231498018	6.94512650988818
C	2.85180601874809	0.67040306702798	4.80948959793478
H	4.34214867306206	2.23398425218378	4.65874351707606
H	2.66214947440807	2.79027673789195	4.41750974179285
C	1.32907711481806	0.87260666934770	6.77587954238834
H	1.70212644005156	2.61163973336115	8.03143339310861
H	1.11416531352318	3.00068789824912	6.39189277599015
H	4.35633226636099	-0.54983450466723	7.67480877674536
C	3.77219457622277	-0.25753497910751	5.60579492525684
C	2.24557392800209	-0.05676086653726	7.57669002507390
H	2.91873656033308	0.42904751357873	3.73421880227884
C	1.40656801474252	0.51517927176234	5.28893001298897
H	0.28948076890242	0.76997106215827	7.13152260293500
H	3.47773062727293	-1.31099309252200	5.45583082569719
H	4.81305999582230	-0.16078070441300	5.24578869872115
H	1.92128255349489	-1.10514134469569	7.45476047872574
H	2.17812722900547	0.17282067764695	8.65523272311231
H	1.06023958997084	-0.52083876452502	5.12731247450149
H	0.73818306962323	1.17528311920984	4.70497605740360

12 References

1. Reinholdt, A.; Pividori, D.; Laughlin, A. L.; DiMucci, I. M.; MacMillan, S. N.; Jafari, M. G.; Gau, M. R.; Carroll, P. J.; Krzystek, J.; Ozarowski, A.; Telser, J.; Lancaster, K. M.; Meyer, K.; Mindiola, D. J., A Mononuclear and High-Spin Tetrahedral Ti^{II} Complex. *Inorg. Chem.*, 2020, **59**, 17834–17850.
2. G. Jafari, M.; Fehn, D.; Reinholdt, A.; Hernández-Prieto, C.; Patel, P.; Gau, M. R.; Carroll, P. J.; Krzystek, J.; Liu, C.; Ozarowski, A.; Telser, J.; Delferro, M.; Meyer, K.; Mindiola, D. J., Tale of Three Molecular Nitrides: Mononuclear Vanadium (V) and (IV) Nitrides As Well As a Mixed-Valence Trivanadium Nitride Having a V_3N_4 Double-Diamond Core. *J. Am. Chem. Soc.*, 2022**, 144**, 10201-10219.
3. Spinney, H. A.; Clough, C. R.; Cummins, C. C., The titanium tris-anilide cation $[Ti(N[^tBu]Ar)_3]^+$ stabilized as its perfluoro-tetra-phenylborate salt: structural characterization and synthesis in connection with redox activity of 4,4′-bipyridine dititanium complexes. *Dalton Trans.*, 2015, **44**, 6784-6796.
4. Evans, D. F., The determination of the paramagnetic susceptibility of substances in solution by nuclear magnetic resonance. *J. Chem. Soc.*, 1959, 2003-2005.
5. Bain, G. A.; Berry, J. F., Diamagnetic Corrections and Pascal's Constants. *J. Chem. Educ.*, 2008, **85**, 532.
6. Gaffney, B. J.; Silverstone, H. J., Simulation of the EMR Spectra of High-Spin Iron in Proteins. In *EMR of Paramagnetic Molecules*, Berliner, L. J.; Reuben, J., Eds. Springer US: Boston, MA, 1993; pp 1-57.
7. CrysAlisPro 1.171.40.61a: Rigaku Oxford Diffraction, R. C., Oxford, UK, 2019.
8. SCALE3 ABSPACK v1.0.7: an Oxford Diffraction program; Oxford Diffraction Ltd: Abingdon, U., 2005.
9. CrysAlisPro 1.171.40.53: Rigaku Oxford Diffraction, R. C., Oxford, UK, 2019.
10. Sheldrick, G., A short history of SHELX. *Acta Crystallogr., Sect. A*, 2008, **64**, 112-122.
11. Sheldrick, G., SHELXT - Integrated space-group and crystal-structure determination. *Acta Crystallogr., Sect. A*, 2015, **71**, 3-8.
12. Dolomanov, O. V.; Bourhis, L. J.; Gildea, R. J.; Howard, J. A. K.; Puschmann, H., OLEX2: a complete structure solution, refinement and analysis program. *J. Appl. Crystallogr.*, 2009, **42**, 339-341.
13. Neese, F. The ORCA Program System. *Wiley Interdiscip. Rev. Comput. Mol. Sci.*, 2012, **2**, 73-78.

14. Adamo, C.; Barone, V., Toward Reliable Density Functional Methods without Adjustable Parameters: The PBE0 Model. *J. Chem. Phys.*, 1999, **110**, 6158-6170.

15. Weigend, F.; Ahlrichs, R., Balanced Basis Sets of Split Valence, Triple Zeta Valence and Quadruple Zeta Valence Quality for H to Rn: Design and Assessment of Accuracy. *Phys. Chem. Chem. Phys.*, 2005, **7**, 3297-3305.

16. Weigend, F., Accurate Coulomb-Fitting Basis Sets for H to Rn. *Phys. Chem. Chem. Phys.*, 2006, **8**, 1057.

17. Neese, F.; Wennmohs, F.; Hansen, A.; Becker, U., Efficient, Approximate and Parallel Hartree–Fock and Hybrid DFT Calculations. A 'Chain-of-Spheres' Algorithm for the Hartree–Fock Exchange. *Chem. Phys.*, 2009, **356**, 98-109.

18. Grimme, S.; Antony, J.; Ehrlich, S.; Krieg, H., A Consistent and Accurate Ab Initio Parametrization of Density Functional Dispersion Correction (DFT-D) for the 94 Elements H-Pu. *J. Chem. Phys*., 2010, **132**, 154104.

19. Tao, J.; Perdew, J. P.; Staroverov, V. N.; Scuseria, G. E., Climbing the Density Functional Ladder: Nonempirical Meta–Generalized Gradient Approximation Designed for Molecules and Solids. *Phys. Rev. Lett.*, 2003, **91**, 146401.

20. Staroverov, V. N.; Scuseria, G. E.; Tao, J.; Perdew, J. P., Comparative Assessment of a New Nonempirical Density Functional: Molecules and Hydrogen-Bonded Complexes. *J. Chem. Phys.*, 2003, **119**, 12129-12137.

21. Staroverov, V. N.; Scuseria, G. E.; Tao, J.; Perdew, J. P., Erratum: "Comparative Assessment of a New Nonempirical Density Functional: Molecules and Hydrogen-Bonded Complexes" [J. Chem. Phys. 119, 12129 (2003)]. *J. Chem. Phys.*, 2004, **121**, 11507.

22. Marenich, A. V.; Cramer, C. J.; Truhlar, D. G., Universal Solvation Model Based on Solute Electron Density and on a Continuum Model of the Solvent Defined by the Bulk Dielectric Constant and Atomic Surface Tensions. *J. Phys. Chem. B*, 2009, **113**, 6378-6396.

23. Pascual-Ahuir, J. L.; Silla, E., GEPOL: An Improved Description of Molecular Surfaces. I. Building the Spherical Surface Set. J. *Comput. Chem.*, 1990, **11**, 1047-1060.

24. Silla, E.; Tuñón, I.; Pascual-Ahuir, J. L., GEPOL: An Improved Description of Molecular Surfaces II. Computing the Molecular Area and Volume. *J. Comput. Chem.*, 1991, **12**, 1077-1088.

25. Pascual-ahuir, J. L.; Silla, E.; Tuñon, I., GEPOL: An Improved Description of Molecular Surfaces. III. A New Algorithm for the Computation of a Solvent-Excluding Surface. *J. Comput. Chem.*, 1994, **15**, 1127-1138.
